# Research on enhancing road apparent crack detection based on the improved YOLOv8n model

**DOI:** 10.1371/journal.pone.0330218

**Published:** 2025-09-04

**Authors:** Wenyuan Xu, Jianbo Xu, Yongcheng Ji, Guodong Li, Hao Li, Zhen Zang

**Affiliations:** School of Civil Engineering and Transportation, Northeast Forestry University, Harbin, Heilongjiang, China; Maulana Abul Kalam Azad University of Technology West Bengal, INDIA

## Abstract

The improved YOLOv8n algorithm is proposed for the existing target detection algorithms to solve the issues of insufficient detection accuracy and leakage due to the target scale variability and complex background interference during road surface crack detection. This algorithm introduces the convolutional block attention module (CBAM) attention mechanism and integrates it with the cross-stage partial-feature fusion (C2f) module in the backbone network. The spatial pyramid pooling faster cross-stage partial channel (SPPFCSPC) module is introduced by integrating the spatial pyramid pooling (SPP) module with the Fully Cross-Stage Partial Convolution (FCSPC) module, which efficiently extracts multi-scale features. Then, the fine Slim-Neck paradigm is adopted to enhance the learning capability of the model while effectively reducing the number of model parameters. Ultimately, to mitigate the detrimental gradients produced by low-quality pictures, the weighted intersection over union (WIOU) loss function is employed instead of the complete intersection over union (CIOU), hence augmenting the bounding box regression efficacy of the network. After the aforementioned enhancements, the experimental outcomes on the road apparent crack dataset indicate that in comparison to the benchmark model YOLOv8n, the average precision (mAP@50), mean average precision (mAP@50–95), and recall of the enhanced algorithm have risen by 1.8%, 1.7%, and 1.8%, respectively. This indicates that the detection accuracy of road fractures is significantly enhanced by the enhanced YOLOv8n, which can more effectively accommodate the requirements of road maintenance.

## Introduction

Road cracks signify the first stages of deterioration and aging; prompt identification and remediation of these fissures are crucial for maintaining safety and prolonging the lifespan of the roadway. The conventional approach to identifying apparent cracks in pavements is physical examination, which depends on visual checks by hand and simple instrumental tests, both of which are subjective and affected by several variables [1]. To tackle these issues, digital image processing techniques [2] have been suggested, wherein appropriate image preprocessing is followed by the use of quantitative statistics and machine learning methods to extract image attributes. Threshold segmentation and edge detection are two of the more popular methods for road crack detection. Chen et al. [3] suggested an automated crack detection approach that employs the extended Otsu algorithm to amalgamate 3D point clouds with 2D images, thereby enhancing the precision of detection. Edge detection is often used for defect detection, which uses local grayscale and gradient data to identify edges [4]. Zhang et al. [5] proposed an improved Canny edge detection algorithm using switched median filtering instead of Gaussian filtering, which removes the noise while retaining the same grayscale value of the non-noise pixel points, thus improving the edge localization accuracy. Despite the efficacy of standard image processing, it requires additional refinement. These methods depend on manual parameter adjustment and extensive preprocessing, lacking the robustness necessary for thorough end-to-end defect recognition. The ongoing advancement of computer vision and machine learning has established the collection of road surface crack datasets via UAV imagery as a prevalent method, subsequently employing deep learning for target detection, significantly enhancing both detection speed and precision.

Beyond traditional image processing methods, deep learning [6], which is based on multi-layer neural network training, offers notable resilience. Deep learning detection methods may be classified into two categories: two-stage and single-stage detection algorithms, depending on the network design. The two-stage algorithm, Faster R-CNN [7], employs a regional convolutional neural network to produce candidate frames, a method that is intricate and computationally demanding. The other category is single-stage detection algorithms, which directly predict the category and location of the target after deep learning; typically represented by the YOLO series [8–10]. The single-stage detection algorithm is more direct and faster than the two-stage detection algorithm. It possesses a more compact model and can fulfill the demands of real-time performance for practical applications. YOLOv3 [11] makes detection faster and more accurate by using multi-scale feature pyramids. However, it has trouble with complex backgrounds and noise, which causes fake detections and missed detections. YOLOv4 [12] enhances the feature representation using CSPDarknet53 and Mish activation function, but still has difficulties in detecting subtle features in complex backgrounds. The rapidity and precision of YOLOv5 [13] are enhanced by an automatic anchor box mechanism; however, it is still insufficient to identify minor fractures in the road surface.YOLOv6 [14] introduces PAFPN and SIOU loss function enhancements but still faces the challenge of multiscale and irregularly shaped defects. YOLOv8 [15] incorporates a sophisticated feature extraction network and an effective training methodology, resulting in notable enhancements in detection accuracy and speed. This study seeks to investigate the improvements of the YOLOv8n algorithm and its relevance to the problem of detecting road surface cracks.

### Related research

In recent years, researchers have conducted many experiments to detect road cracks using deep learning. Oliveira et al. [16] suggested a comprehensive method for the automated identification and characterization of fractures on flexible pavement surfaces, which eliminates the need for manually labeled samples to minimize human subjectivity caused by traditional visual measurements. Xu et al. [17] used a training strategy that combines Faster R-CNN and Mask R-CNN models. Good detection results were achieved using a limited number of crack images for training. Nhat et al. [18] compared the CNN with the traditional edge detection operator crack extraction method; the recognition accuracy was 79.99% and 92.08% respectively, and the deep learning-based method showed significant advantages. Wang et al. [19] proposed an improved algorithm for small-scale target detection based on YOLOv3, which enhances the accuracy of small-scale target detection by adding a branch parallel to the main trunk. YUG et al. [20] used the BoT module in YOLOv5 to improve the model accuracy by capturing long-range dependencies to obtain global information. Xu et al. [21] employed faster R-CNN and YOLO-RDD lightweight models to achieve an 85% reduction in computation while preserving good detection accuracy. Wang et al. [22] developed an improved YOLOv8 network with synthetic data. The augmented YOLOv8 network incorporates the Squeeze and Excite Attention Module and the Swin Transformer Module, which are engineered to precisely distinguish various sorts of road surface ailments.

While the aforementioned research has resulted in numerous enhancements to the road surface fracture target measurement technology, it has also facilitated substantial advancements in this algorithm. Nevertheless, the following challenges remain in place as a result of the variability of the road background:

(1) Complexity: The large-size transformation of road crack targets and the complex and variable road context exacerbate the challenge of detecting model targets.
(2) Robustness: The generalization of the model’s performance is diminished when the training data lacks training samples for various scenarios.

To resolve the above concerns, this article suggests a road surface crack detection algorithm that is based on improved YOLOv8n, which will enhance the efficiency and accuracy of crack detection.

### Introduction to the YOLOv8n algorithm

**Fig 1:**
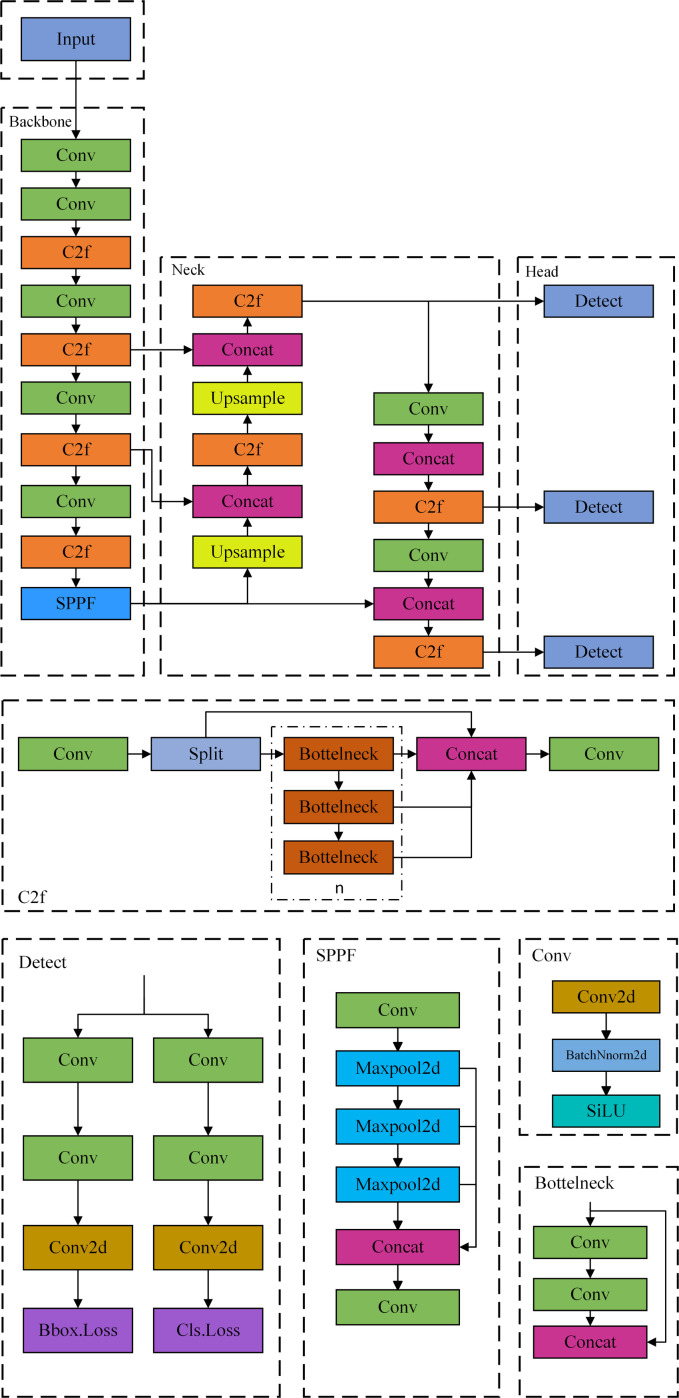
YOLOv8n network architecture diagram.

YOLOv8 is an iteration of the YOLO (You only look once) series of target detection algorithms that exhibits superior performance in segmentation and detection tasks when contrasted with its predecessors. The YOLOv8n model is chosen as the foundational model in this research because it not only retains the real-time efficiency of the YOLO series but also has specific optimizations for target detection in intricate environments. The lightweight architecture facilitates millisecond inference on edge devices, while the optimization of YOLOv10/v11 enhances the efficiency of general-purpose object recognition but diminishes the detection speed of skinny, low-contrast cracks. YOLOv8n’s PANet multi-scale feature fusion and dynamic label assignment mechanism enhance the detection of small cracks and branch breakpoints, while YOLOv10’s SPPF layer and YOLOv11’s dynamic pruning strategy prioritize large targets or computational efficiency, lacking specificity in crack feature extraction. Furthermore, YOLOv8n utilizes the stable codebase and pre-trained weights of the Ultralytics framework, facilitating rapid fine-tuning on the crack dataset. Its engineering maturity significantly surpasses that of YOLOv10/v11, which remains in the experimental phase, thus making it the benchmark model for this study. YOLOv8n is composed of four components: Input, Backbone, Neck, and Head. The network structure of YOLOv8n is illustrated in Fig 1.

The input component employs letterbox scaling and filling mode to continually resize the input image for training, while the Mosaic technique is utilized for data augmentation. Mosaic is disabled during the final 10 epochs of training to enhance the model’s robustness.

The backbone network utilizes a cross-stage localized architecture to improve feature information extraction. It mostly comprises the standard convolution module, C2f module, and SPPF module. The primary function of the standard convolution module is to enhance model generalization, expedite convergence, and mitigate gradient vanishing. The C2f module promotes gradient mobility and feature expression in the network by partitioning and amalgamating feature maps, hence offering superior gradient mobility. The SPPF is a spatial pyramid pooling framework that enhances local feature extraction by broadening the acceptance domain and integrating local features with global characteristics.

Neck primarily integrates multi-scale features derived from the backbone network to produce the feature pyramid. Its architecture is founded on the feature pyramid of YOLOv5, with the addition of a bottom-up path aggregation network layer, thereby enhancing feature fusion and improving the network’s detection performance.

The detection head structure is the component of the target detection network that predicts bounding boxes and category probabilities. In the YOLOv8n model, the head utilizes a Decoupled Head design, which differentiates classification and regression tasks from the conventional coupled head, thereby enhancing the model’s convergence speed and detection efficacy.

## Materials and methods

**Fig 2:**
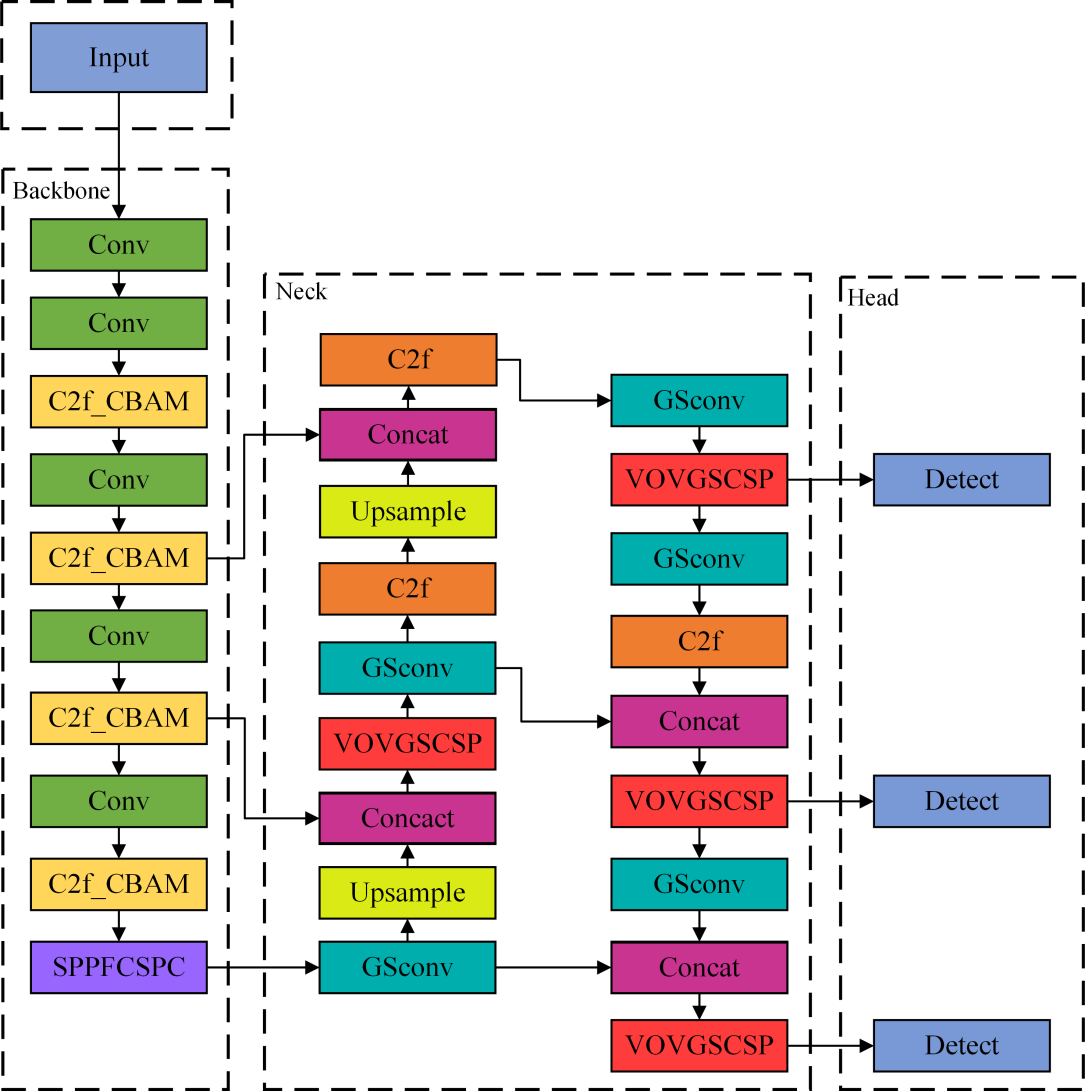
Improved YOLOv8n network structure.

This study addresses the challenges of road crack detection, including weak small-target features, significant background interference, and inconsistent sample quality, by thoroughly optimizing the YOLOv8n model and developing a high-performance detection framework that incorporates CBAM, SPPFCSPC, Slim-Neck, and WIoU modules. C2f_CBAM is implemented in Backbone to substitute all C2f modules, enhancing crack feature representation, while SPPFCSPC augments multi-scale fusion capabilities, and Slim-Neck facilitates a lightweight design, hence decreasing computational demands in the Neck component. Upon comparison and analysis of the three iterations of the WIOU loss function, we have selected the WIOUv3 version as the most appropriate for road surface crack identification. The aforementioned modifications can enhance the model’s efficacy in detecting cracks under intricate road conditions. The improved YOLOv8n structure is illustrated in Fig 2. The specific modifications implemented for the detection of apparent cracks in the roadway are as follows:

(1) The CBAM attention module is included in the C2f structure, resulting in the C2f_CBAM module, which entirely supplants the old C2f module in the Backbone. Initially, the C2f_CBAM module enhances the prominent representation of crack features via the channel attention mechanism, significantly augmenting the capacity to detect slender and low-contrast crack characteristics. Subsequently, the spatial attention mechanism directs the model’s focus toward anomalous areas within the pavement texture while mitigating complex background interference. Furthermore, the module’s cross-phase connectivity structure preserves multi-scale crack feature information, ensuring robust detection of cracks of varying widths. Ultimately, all original C2f modules in Backbone have been substituted with C2f_CBAM modules to create an attention-driven hierarchical feature extraction network, addressing the issue of similarity between cracks and shadows, stains, and other interferences in road scenes.
(2) The SPPF module in Backbone has been supplanted by the SPPFCSPC module, which is specifically tailored to the unique requirements of road crack detection. The SPPFCSPC module markedly decreases computing complexity while preserving the benefits of multiscale feature fields due to its distinctive cross-stage partially-connected architecture, rendering it more appropriate for several pavement crack detecting situations. The enhanced pyramid pooling architecture adeptly captures road damage characteristics across various scales, from minor fissures to broader fractures, with a notable increase in detection sensitivity for elongated cracks. The module’s integrated channel redistribution mechanism effectively mitigates similarity confusion between cracks and interferences, such as pavement joints and ruts, demonstrating superior feature discrimination in complex road conditions, including water stains, oil stains, and shadows.
(3) The Slim-Neck module is presented in the Neck section, specifically tuned for computational efficiency and feature fusion in road inspection jobs. The Slim-Neck module optimizes the feature pyramid architecture via lightweight operations like GSConv, which diminishes the computational load of the Neck section while preserving multi-scale feature fusion capabilities, thereby enhancing the model’s deployment viability on mobile devices. The distinctive feature compression and reorganization mechanism effectively preserves the continuity characteristics of cracks, particularly the prevalent irregular cracks in road environments, facilitating a more precise feature representation. Simultaneously, the module’s cross-layer connection enhances the fusion efficiency of deep and shallow features, thereby improving the model’s consistency in detecting varying degrees of subtle cracks.
(4) The CIOU loss function is substituted with the WIOU function, aimed at enhancing the issue of disparate sample quality in road crack detection. This study thoroughly examines and analyses the three versions of WIOU (v1-v3) to better accommodate the specific requirements of road surface crack detection, ultimately selecting the third version. WIOUv3 mitigates the impact of numerous low-quality samples on model training in intricate road scenarios via a dual adjustment mechanism involving dynamic focusing coefficients and sample quality evaluation. The novel asymmetric gradient assignment strategy enhances the model’s regression capability on challenging samples, including fractured discontinuous cracks and low-contrast edges. Additionally, the implementation of a gradient weighting strategy predicated on the aspect ratio of cracks directs the model’s attention towards the transverse and longitudinal alignment precision of slender cracks.

YOLOv8n innovatively incorporates CBAM, SPPFCSPC, Slim-Neck, and WIoU modules to establish a synergistic optimization of multi-dimensional feature improvement and computing efficiency inside the detection framework, characterized by a distinctive integration technique as follows:

(1) CBAM is integrated into Backbone, utilizing a dual-attention mechanism for both channel and spatial dimensions, which enhances the textural features of superficial cracks while mitigating noise interference from the road surface. This approach amplifies crack texture features at a superficial level while mitigating pavement noise interference.
(2) Its output synergizes with the multi-scale features produced by SPPFCSPC after Backbone via cross-stage local connectivity (CSPC), preserving the local details of minor cracks while simultaneously capturing the overarching morphology of extensive cracks.
(3) Slim-Neck employs depth-separable convolution and dynamic channel clipping to provide efficient reuse and lightweight fusion of cross-scale features in the Neck component, with its feature route selection process predicated on crack depth. The feature path selection algorithm adaptively modifies the fusion weights based on the crack scale.
(4) The WIOU loss function establishes a closed loop with the CBAM-enhanced feature quality via dynamic IOU weight assignment and a crack aspect ratio gradient weighting strategy, which allocates a greater loss gradient to low-quality prediction frames to enhance regression accuracy, while integrating the multi-scale features of SPPFCSPC to accommodate the detection requirements of cracks at various scales.

### Design of the C2f_CBAM module

The C2f module is an essential element in the YOLOv8 detection framework, primarily responsible for facilitating cross-stage aggregation of multi-scale information. Consequently, the C2f module must process and fuse feature maps from various levels, which escalates the model’s computational complexity, resulting in increased training time and the potential for erroneous detections and omissions. This study addresses the aforementioned issues by incorporating the CBAM [23] into the C2f module of the backbone network, thus creating the C2f_CBAM module. By integrating channel attention and spatial attention, it adaptively enhances the input feature map, amplifying the information-dense channels and geographical areas, thus augmenting the feature characterization capability. It is engineered for computational efficiency to guarantee a lightweight execution of the attention mechanisms. The CBAM module comprises two sub-modules: the Channel Attention Module (CAM) [24] and the Spatial Attention Module (SAM) [25]. The computation procedure is delineated in equations (1) and (2).


$$F\prime =M_{C}(F)⊗F$$ (1)

$$F\prime \prime =M_{S}(F\prime )⊗F\prime$$ (2)

**Fig 3:**
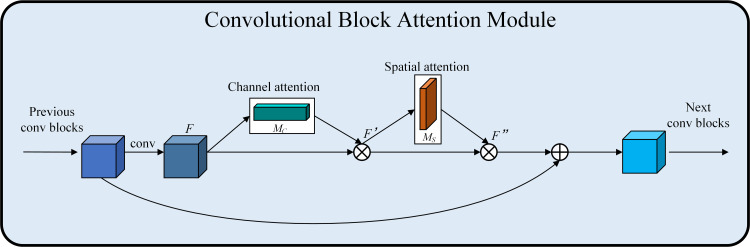
Structure of CBAM.

Where F is the feature map, $M_{C}$ and $M_{S}$ denote the channel attention module and spatial attention module, ⊗ denote the element-by-element multiplication, F′ and F′′ denote the output feature maps after the channel-based and spatial attention, respectively. The structural diagram of the CBAM attention mechanism is illustrated in Fig 3.

The channel attention module serves to augment the feature representation of each channel. It first employs maximum pooling and average pooling on the input feature maps across the spatial dimensions, maintaining the channel dimensions while condensing the other dimensions to one, resulting in a one-dimensional vector. Then pass through an MLP (Multilayer Perceptron) with two convolutional layers sharing weights. The first layer is convolved using the Rectified Linear Unit activation function, while the second layer is convolved using the sigmoid activation function to obtain the output weight. The attention mechanism on the channel is achieved by performing element-by-element multiplication of this weight and the input feature map. Equation (3) illustrates the preceding procedure.


$$\begin{array}{c} M_{C}(F)=\sigma (MLP(AvgPool(F))+MPL(MaxPool(F)))\\ =\sigma (W_{1}(W_{0}(F_{avg}^{C}))+W_{1}(W_{0}(F_{\max}^{C}))) \end{array}$$ (3)

**Fig 4:**
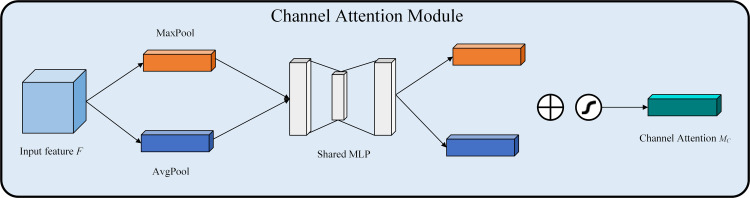
Structure of CAM.

Where σ denotes the sigmoid activation function, $W_{0}$ and $W_{1}$ denote the two convolution operations. Respectively, $F_{avg}^{C}$ denotes average pooling $F_{\max}^{C}$ denotes maximum pooling. The CAM structure diagram is illustrated in Fig 4.

A spatial attention module is utilized to enhance the importance of each spatial location in the feature map, enabling the network to focus more efficiently on vital regions within the image. The feature map F′ produced by the channel attention module serves as the input feature map for this module. Initially, channel-based average pooling and maximum pooling are performed to obtain two H×W×1 feature maps. Subsequently, these two feature maps are concatenated along the channel dimension, and following a 7×7 convolution operation, the channel count is reduced to 1. Ultimately, a spatial attention feature $M_{S}$ is produced using a sigmoid operation. This feature is multiplied by the input feature map to yield the final generated feature. The formula representation is illustrated in equation (4).


$$\begin{array}{c} M_{S}(F)=\sigma (f^{7\times 7}([AvgPool(F);MaxPool(F)]))\\ =\sigma (f^{7\times 7}([F_{avg}^{S};F_{\max}^{S}])) \end{array}$$ (4)

**Fig 5:**
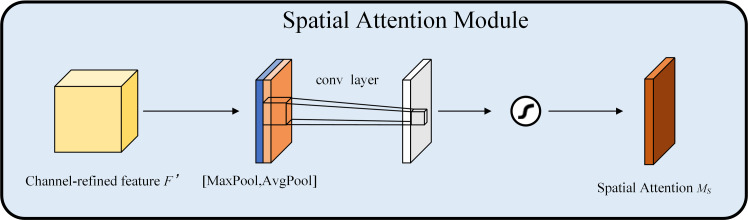
Structure of SAM.

Where σ denotes the sigmoid activation function and $f^{7\times 7}$ denotes a convolution operation with a convolution kernel size of 7×7. The SAM structure is shown in Fig 5.

**Fig 6:**
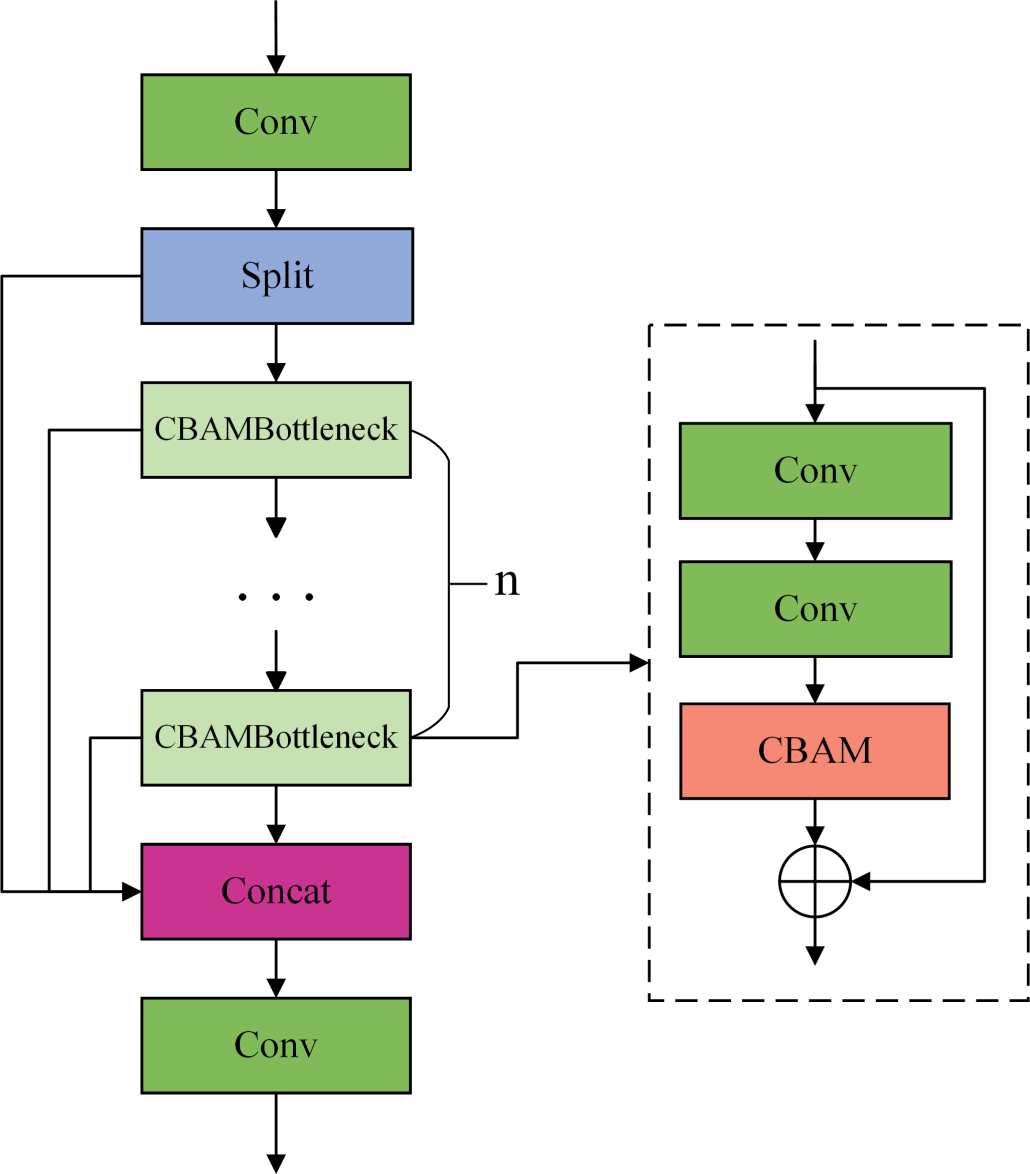
Structure of C2f_CBAM.

CBAM module improves the model’s feature extraction by facilitating adaptive learning of channel and spatial significance while providing plug-and-play functionality without augmenting computing burden or parameter quantity. Consequently, this study integrates the CBAM module into the Bottleneck of the C2f module. After adding the CBAM module to the convolution operation inside the Bottleneck module, the feature map in the Bottleneck module is utilized to weight the features at different scales with attention, thus improving the network’s ability to perceive multi-scale information, which enables the module to take into account richer feature information and further optimize the feature map’s representational ability. Fig 6 illustrates the structure of the C2f_CBAM module.

### SPPF module improvement

YOLOv8n employs the SPPF architecture to enhance the spatial information inside the feature map, primarily extracting input features using three max-pooling procedures and utilizing a concatenation process for feature fusion. This method effectively extracts input feature information; nevertheless, it sacrifices certain global feature information due to its reliance on maximum pooling operations for feature extraction. Consequently, the SPPFCSPC [26] module is proposed to diminish computational complexity and augment the expressive capacity of model features, while concurrently managing detection targets of varying sizes, thereby enhancing adaptability to intricate scenarios.

**Fig 7:**
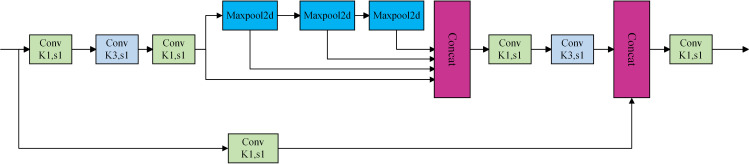
Structure of SPPFCSPC.

The primary objective of SPPFCSPC is to augment the feature extraction proficiency of convolutional neural networks through the integration of multi-scale spatial pyramid pooling and an enhanced cross-stage partial network architecture. SPPFCSPC first does multi-scale pooling with various pooling layer dimensions, often configured to sensory fields of 5×5 9×9, and 13×13 enabling the aggregation of information across diverse spatial scales to capture multi-scale details within the image. The fast spatial pyramid pooling module is employed to enhance feature representation via numerous short pooling operations, hence decreasing computing volume. The module utilizes grouped convolution to partition the feature map into several groups, executing independent convolution operations within each group. This approach distributes the computation across various groups, thereby alleviating the overall computational burden while preserving the model’s expressive capacity. Finally, the feature map is ultimately divided into two segments: one is directly linked to the successive layers by residual connections, while the other is combined following a convolution procedure. This structure can significantly diminish redundant gradient information, enhance gradient flow and feature reuse capability, and hence improve model performance. Fig 7 illustrates the structural diagram of the SPPFCSPC module.

### Combining Slim-Neck lightweight feature fusion module

Presently, high-precision target detection encounters the challenge that real-time performance is sometimes unachievable in complicated scenarios or extensive datasets. Secondly, although the target detection system achieves elevated detection accuracy, such precision typically entails increased algorithmic complexity and substantial processing demands, necessitating hardware devices with robust computational and storage capacities.

Consequently, minimizing computing complexity and volume while satisfying accuracy criteria is the primary emphasis of contemporary research. This study employs the grouped spatial convolution (GSConv) [27] module and the vision-oriented variant of ghost-based cross-stage partial (VOV-GSCSP) [28] module within the Slim-Neck [29] design framework to enhance and reduce the weight of the Neck component of YOLOV8n, hence achieving model efficiency while preserving accuracy.

**Fig 8:**
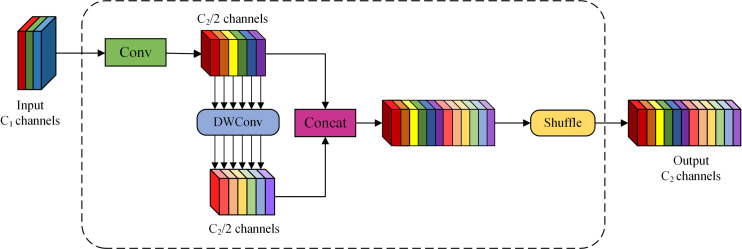
Structure of GSConv.

The implementation of GSConv addresses the challenge of reconciling model speed and accuracy, achieving model efficiency while preserving precision. GSConv is a global sparse convolution technique that facilitates the efficient aggregation of global information by incorporating global sparsity into the feature map. In contrast to standard convolution modules, GSConv executes convolution operations solely at sparse locations while preserving global information, thereby decreasing computational load, enhancing model efficiency, and mitigating the adverse effects of depth-separable convolution deficiencies on the model. The GSConv module employs grouped convolution and selective activation techniques, which can markedly improve the model’s learning capacity by minimizing parameter redundancy and optimizing feature extraction efficiency. It decreases computational demands for each group of convolutions via grouped convolutions and captures inter-group features through spatial convolutions. Additionally, GSConv incorporates a Shuffle operation to facilitate the exchange of information among various convolutional groups, hence enhancing feature variety and the model’s learning capacity. Fig 8 illustrates the structure of the GSConv module.

The input feature map comprises $C_{1}$ channels; following a convolutional layer, the output feature map contains $C_{2}/2$ channels. Thereafter, a depth-wise convolution (DWConv) is executed, conducting convolution operations on each channel of the input feature map to produce a new feature. This grouped convolution reduces the number of parameters and computational cost while preserving the number of channels in the output. The output results of Conv and DWConv are concatenated to produce a feature map with $C_{2}$ channels, which are subsequently rearranged through a Shuffle operation to yield the final feature map with $C_{2}$ channels. Rearranging the sequence of channels facilitates the transfer of information among various channels, thereby enhancing cross-channel information fusion.

In the Neck part, when GSConv replaces the traditional Conv, it handles global information more efficiently and can reduce the consumption of computational resources. It can maximize the generalization ability of the model while reducing the complexity. Subsequently, the VOV-GSCSP module is proposed based on GSConv, and these two modules together form the Slim-Neck architecture.

**Fig 9:**
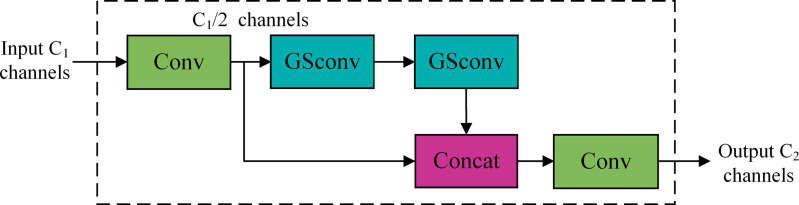
Structure of V0V-GSCSP module.

**Fig 10:**
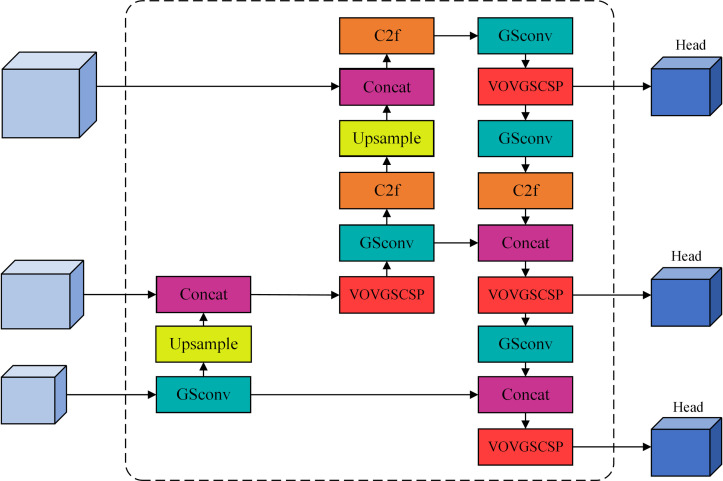
Structure of Slim-Neck module.

The VOV-GCSP module is to reduce the dimensionality of the input feature map by 1×1 ordinary convolution first, and then input the result into two branches at the same time, one of which is a two-layer GSConv, and the other branch is spliced with the result of the two-layer GSConv, and then finally output it by 1×1 convolution. This module can fully extract the feature information of both shallow and deep networks and further fuse them so that the output feature map is rich in both positional and semantic information. The VOV-GSCSP module fuses cross-layer features and pruning strategies, and optimizing the network structure of the model makes the transfer of feature maps more efficient while maintaining rich contextual information. In summary, the Slim-Neck architecture can reduce the computation and complexity of the model while maintaining high accuracy. Figs 9 and 10 show the architecture of the VOV-GSCSP and Slim-Neck modules.

### Improvement of the loss function

E-assessment of target detection efficacy is intricately linked to the formulation of the loss function. YOLOv8 utilizes the CIOU [30] loss function, which calculates the distance between the centroids of the predicted and actual frames, along with the diagonal length of the smallest enclosing rectangle for both frames, resulting in increased computational demands for the model. Furthermore, the aspect ratio penalty component in the CIOU loss function is determined using a static function, which is not suitable for all target types, particularly when there is significant variation in the size of the detection target.

To address the aforementioned issues, the WIOU [31] loss function is employed in place of the CIOU loss function. The WIOU loss function enhances model adaptability and generalizability through its weighting method, hence boosting detection performance across many settings. Simultaneously, varying weights are allocated to the intersection ratios of distinct regions, which aids in mitigating the overfitting of the model’s training data and enhancing the model’s robustness. Currently, there are three versions of the WIOU loss function. For the WIOUv1 [32] loss function, when the anchor frame has a good overlap with the target frame, it can attenuate the penalty of geometric factors. With less training intervention, the model can achieve better generalization. Furthermore, distance attention is formulated, resulting in WIOUv1, which incorporates a dual-layer attention mechanism. The formula for the WIOUv1 loss function is illustrated below.


$$L_{WIOUv1}=R_{WIOU}\times L_{IOU}$$ (5)

$$R_{WIOU}=\exp(\frac{{\left(x-x_{gt}\right)}^{2}+{\left(y-y_{gt}\right)}^{2}}{{\left(W_{g}^{2}+H_{g}^{2}\right)}^{*}})$$ (6)

$$L_{IOU}=1-IOU$$ (7)

Where x, y and $x_{gt}$, $y_{gt}$ denote the coordinates of the centroid in the predicted and real frames, respectively, $R_{IOU}$ denotes the loss of the high-quality anchor frame, $W_{g}$ and $H_{g}$ are the width and height of the real frame, IOU is the intersection and concurrency ratio, and * denotes the separation of the minimum bounding box from the gradient computation, which reduces the detrimental effects produced by the model training.

The WIOUv2 [33] loss function attains these objectives by integrating the distance-attention mechanism with the dynamic non-monotonic focusing mechanism. The computation of loss considers the positional divergence between predicted and actual frames, the size discrepancy, and the characteristics of their minimum enclosing frames. This architecture enables the model to focus more on ordinary quality anchor frames during training, hence minimizing the over-optimization of already well-aligned anchor frames. The WIOUv2 formula is as follows.


$$L_{WIOUv2}=L_{IOU}^{\gamma *}\times L_{WIOUV1},\gamma >0$$ (8)

The gradient of the WIoUv2 backpropagation changes due to the increased focusing factor.


$$\frac{\delta L_{WIOUv2}}{\delta L_{IOU}}=L_{IOU}^{\gamma *}\frac{\delta L_{WIOUv1}}{\delta L_{IOU}},\gamma >0$$ (9)

Since the gradient gain $r=L_{IOU}^{\gamma *}\in [0,1]$, the gradient gain decreases with the decrease $L_{IOU}$ during the model training process, it leads to slower convergence in the late training period. Therefore, the mean value $L_{IOU}$ is introduced as a normalization factor.


$$L_{WIOUv2}=(\frac{L_{IOU}*}{\overset{―}{L_{IOU}}}{)}^{\gamma }L_{WIOUv1}$$ (10)

Where $\overset{―}{L_{IOU}}$ is the exponential moving average with momentum m. Dynamically updating the normalization factor keeps the gradient gain $r=(\frac{L_{IOU}*}{\overset{―}{L_{IOU}}}gamma$ at a high level in general, which solves the problem of slow convergence at the late stage of training.

To avoid large harmful gradients for lower-quality samples, the parameters β and WIOUv1 are introduced to construct WIOUv3 [34]. The loss calculation and dynamic focusing mechanism are illustrated in the following equations, respectively.


$$L_{WIOUv3}=\gamma \times L_{WIOUv1}\times L_{IOU}$$ (11)

$$\gamma =\frac{\beta }{\delta \alpha^{\beta -\delta }}$$ (12)

Where α is a constant, β is the outlier β∈[0,+∞), which is used to define the quality of the anchor frames to enable better regression of the normal quality anchor frames, and a smaller outlier indicates a higher quality of the anchor frames.

Due to the dynamic nature of the quality classification criteria for both $L_{IOU}$ anchor frames, WIoUv3 can implement a gradient gain allocation technique that optimally aligns with the prevailing circumstances at any given instant. In this paper, we will compare and test three versions of the loss function. The version exhibiting the most significant enhancement impact will be chosen as the loss function for the optimized model.

## Results

### Dataset

This study utilized an open-source crack disease database from the industry, which was integrated with self-collected disease data to create a larger and more diversified dataset. The research includes 115 sheets from the Crack Forest dataset, 500 sheets from the CRACK500 dataset, and 3739 sheets from a self-collected dataset.

In the road surface crack dataset constructed in this study, the distribution and causes of various types of crack samples are characterized as follows. Longitudinal cracks have the largest sample size of 1,893, typically resulting from the cumulative fatigue effects of traffic loads and differential settling of the roadbed. Transverse cracks were the second most prevalent, with 1,485 samples, primarily resulting from tensile strains induced by temperature gradients and inadequate treatment of construction joints. The block fractures of 367 are intimately associated with the inadequate load-bearing capability of the pavement construction and the degradation of the base material. The cracking pattern of 298 sheets distinctly delineates the damage pattern of the pavement resulting from prolonged heavy traffic and material strain accumulation. The dataset comprises 233 mixed crack samples (including longitudinal and transverse cross-cracks) and 78 interference samples exhibiting crack textural characteristics, which can offer negative sample support for enhancing model resilience during training.

To enhance the model’s resilience and generalization, the training set photos are subjected to vertical and horizontal flipping, as well as random cropping. In terms of geometric transformation, horizontal flipping is performed with 40% probability to simulate different shooting directions, and vertical flipping is performed with 30% probability to increase morphological diversity; meanwhile, random cropping is implemented, with a scale range of 90%−100% of the original size, to ensure that the effective labeling frames are retained. In terms of color space enhancement, ± 20% brightness adjustment is used, and ±15° random rotation is introduced to enhance the model’s adaptability to complex road conditions. All enhancement operations maintain the geometric consistency of the labeled frames and are normalized using the ImageNet standard. The above preprocessing operations enrich the diversity of the training data and help the model to better cope with various complex scenes in real applications.

### Experimental environment and experimental parameters configuration

All trials in this study were conducted in a consistent environment: Ubuntu 20.04 operating system, Intel(R) Xeon(R) Silver 4216 CPU at 2.10 GHz, and Tesla T4 GPU. The programming language utilized was Python 3.8.10, with CUDA 11.7 enhancing the computational framework; Pytorch 1.13 served as the deep learning framework, and the detailed training parameter configurations are presented in Table 1. In the model training process, a stochastic gradient descent optimizer is employed to minimize the likelihood of the model converging to a local optimum. Ablation and comparative experiments are conducted to demonstrate the scientific efficacy of the enhanced model.

**Table 1:** Configuration of training parameters for the dataset.

Parameters	Value
Learning Rate	0.01
Batch Size	8
Image Size	640×640
Epochs	300
Momentum	0.937

### Evaluation indicators

In this experiment, the computational quantity GFlops, parameter quantity Parameters, precision (P), recall (R), mAP@50 with IoU threshold of 0.5, and mAP@50−95 after a weighted average of IoU from 0.5 to 0.95 are used as the evaluation indices. The smaller values of GFlops, parameters indicate that the algorithm’s complexity is small. The higher value of P represents the high accuracy of the algorithm’s detection results and fewer false detections; the higher value of R represents the algorithm’s ability to detect all targets as much as possible, with fewer missed detections. The higher value of mAP@50 represents the more overlapping part of the predicted frame with the real frame, and the more accurate the matching is; the higher value of mAP@50−95 represents the better the average of the target detection model under different overlapping thresholds (from 0.5 to 0.95). The average of P and R after the weighted average of the overlapping thresholds is used as the evaluation index. The better the detection performance. P, R, mAP@50, and mAP@50−95 formulas are illustrated below.


$$P=\frac{TP}{TP+FP}$$ (13)

$$R=\frac{TP}{TP+FN}$$ (14)

TP denotes the number of road targets detected correctly, FP denotes the number of road targets misdirected, and FN denotes the number of missed detections.


$$mPA=\frac{\sum_{j=1}^{N}P_{j}}{M}$$ (15)

Where M denotes the total number of categories used for detection, and N denotes the number of images to be detected. The mean Average Precision (mAP) includes mAP@50 and mAP@50−95, both of which depend on the predetermined IoU threshold. Its expression is as follows.


$$mAP@50=\frac{\sum_{j}^{N}AP_{j}}{M}$$ (16)

$$mAP@50-95=\frac{\sum_{k=0}^{9}mAP@(0.5+0.05k)}{10}$$ (17)

### Effects of different attention mechanisms on model performance

To assess the efficacy of the C2f_CBAM enhancement module, YOLOv8n serves as the baseline model for comparative analysis with other C2f enhancement modules. The CAM, SAM, and CBAM are employed in conjunction with the C2f module to create the hybrid modules C2f_CAM, C2f_SAM, and C2f_CBAM, with the experimental findings presented in Table 2.

**Table 2:** Performance comparison results of different C2f improvement modules.

Module	P%	R%	mAP@50%	mAP@50–95%	GFLOPs
**C2f**	93.8	91.0	93.7	79.0	8.1
**C2f_CAM**	92.6	91.2	94.0	79.1	9.3
**C2f_SAM**	93.7	90.8	93.1	78.0	11.5
**C2f_CBAM**	94.7	91.5	94.5	79.1	12.7

Experimental findings indicate that the C2f_CBAM module demonstrates considerable benefits in the YOLOv8m-based road fracture detection challenge. C2f_CBAM surpasses other modules on critical measures, including precision (94.7%), recall (91.5%), and mAP@50% (94.5%), by using the synergies of channel attention and spatial attention, achieving enhancements of 0.9%, 0.5%, and 0.8%, respectively, relative to the baseline C2f. Despite its greater processing demands compared to C2f_SAM and C2f_CAM, the enhancement in performance significantly surpasses the rise in computational expense, and it exhibits superior robustness, particularly in addressing intricate pavement backdrops and multi-scale cracks. The single-attention module has notable limitations: the absence of spatial awareness in C2f_CAM results in a 1.2% decline in precision, whereas spatial noise interference in C2f_SAM diminishes recall and mAP. Consequently, the dual-attention mechanism of CBAM is better suited for the dual requirements of global features and local details in crack detection tasks, and its overall performance advantages render it the optimal choice for precision-centric scenarios.

**Fig 11:**
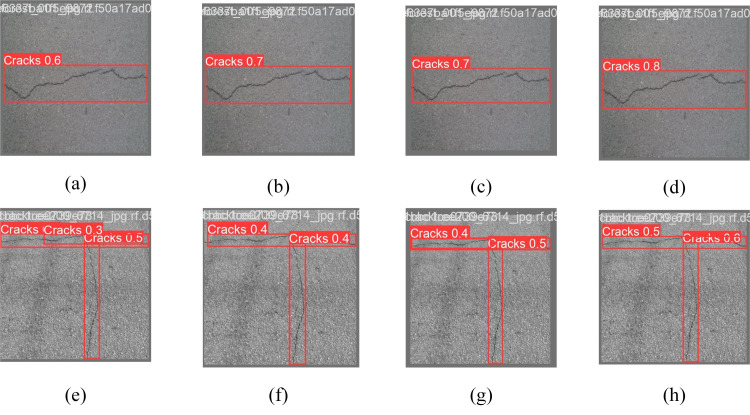
Comparative visualization of different attention mechanisms.

Fig 11 illustrates the comparative feature visualization images of YOLOv8 before and after improvement using the C2f module. Fig 11(a) indicates that YOLOv8n exhibits the lowest confidence regarding the target cracks and encompasses a reduced area. Both C2f_CAM and C2f_SAM detections exhibit lower confidence than the augmented C2f_CBAM attention mechanism depicted in Fig 11(d); the enhanced model demonstrates significant focus on both the center and perimeter of the target crack. Fig 11(e)–11(h)) demonstrate that the enhanced CBAM module effectively identifies both horizontal and vertical cracks with superior confidence compared to the previous model. This indicates that the incorporation of the CBAM attention mechanism facilitates a more complete consideration of the target crack features and spatial information across various scales, hence enhancing detection accuracy.

### Comparison test of different SPPF algorithm improvement modules

To assess the efficacy of the SPPFCSPC module, YOLOv8n serves as the benchmark model for a comparative analysis with various SPPF enhancement modules, including SPPCSPC, SimSPPF, and SPPFCSPC, with the experimental findings presented in Table 3. The experimental results indicate that SPPFCSPC has enhanced the precision rate, recall rate, and average accuracy without significantly increasing the computational volume. Compared with SPPF, SPPFCSPC further optimizes the accuracy of the detection results, reduces the possibilities of misjudgment and omission, and improves the ability to detect multi-scale targets in complex scenes, while improving the computational speed and reducing the computational volume. The introduction of SPPCSPC increases the computational volume of the model due to the inclusion of multiple convolutional and SPP layers, and there is a certain degree of spatial information loss when pooling operations are performed, especially when dealing with smaller input sizes or extreme changes in object scales, which can reduce the accuracy and recall. Compared with SimSPPF, although SPPFCSPC has a 0.3% decrease in mAP@50–95 and an increase in the amount of computation, all the other indicators have increased to varying degrees. Moreover, SPPFCSPC shows stronger adaptability under different data types and scales and can maintain stable and efficient performance when processing large-scale data and dealing with data diversity.

**Table 3:** Performance comparison results of different SPPF improvement modules.

Module	P%	R%	mAP@50%	mAP@50–95%	GFLOPs
**SPPF**	93.9	91.9	93.8	79.0	8.1
**SPPCSPC**	93.6	90.5	93.2	79.1	8.5
**SlimSPPF**	94.3	92.2	94.4	80.1	8.0
**SPPFCSPC**	95.8	92.3	95.8	79.8	8.2

**Fig 12:**
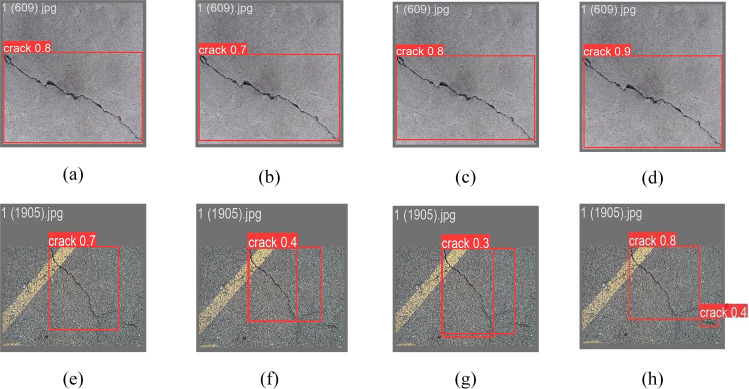
Comparison of model visualization before and after SPPFCSPC improvement.

In addition, to demonstrate the enhancement effect of this improved module more intuitively, the feature visualization comparison images before and after the module improvement are illustrated in Fig 12, which show the detection results using the SPPF, SPPCSPC, SlimSPPF, and SPPFCSPC modules, respectively, from left to right. As can be seen from the images, the confidence level of Fig 12(b) has decreased compared to Fig 12(a), while the confidence level of Fig 12(c) has not changed, but it does not completely describe the crack profile, while Fig 12(d) shows that the bounding box of the SPPFCSPC module is more accurate and has the highest confidence level compared to the other modules. While the bounding box positioning in Fig 12(e) and Fig 12(f) is confusing and intersecting, which cannot pinpoint the crack location, in Fig 12(g), although the confidence level is improved, the tiny crack in the lower right corner of the picture is missed, while Fig 12(h) has a higher detection accuracy and solves the problem of missed detection. It shows that this improvement can pay more attention to the overall characteristics of the input information and improve the overall performance of the model for road crack target detection.

### Comparative testing of lightweight network modules

This experiment aims to integrate the Slim-Neck module into the YOLOv8 model and perform a thorough comparative analysis against the original architecture and prevalent Neck designs, including BiFPN and CSPNeck, with an emphasis on validating the overall benefits of Slim-Neck regarding model lightweighting and detection accuracy. The experimental results are presented in Table 4.

**Table 4:** Comparison results of different lightweight modules.

Module	P%	mAP@50%	Params/10^6^	GFLOPs
**PAN-FPN**	93.8	93.5	3.0	8.1
**BiFPN**	94.0	93.7	3.4	9.3
**CSPNeck**	93.5	93.2	2.8	7.5
**Slim-Neck**	94.2	94.0	2.5	6.8

Experimental findings indicate that the Slim-Neck module displays considerable advantages across various critical criteria. Slim-Neck demonstrates exceptional detection performance, achieving 94.0% mAP@50% and 94.2% precision, surpassing all comparator modules by 0.5% and 0.4% respectively, when compared to the baseline PAN-FPN, particularly excelling in the detection of small objects. In terms of lightweight design, it achieves the minimal parameter count of 2.5M and computational load of 6.8 GFLOPs across all groups, reflecting reductions of 16.7% and 16% respectively compared to PAN-FPN, thus fulfilling the optimization objective of “reducing quantity without sacrificing quality.” Slim-Neck attains an ideal equilibrium between computational efficiency and detection accuracy, providing 0.8% greater detection accuracy while necessitating 9.3% fewer calculations than CSPNeck. This exceptional performance arises from its revolutionary GSConv and VoVGSCSP architectural designs, which guarantee efficient multi-scale feature fusion while markedly diminishing computational redundancy. Slim-Neck exhibits advancements in three areas: precision, lightweight construction, and computational efficiency, positioning itself as the superior alternative for the Neck component in YOLOv8 models.

**Fig 13:**
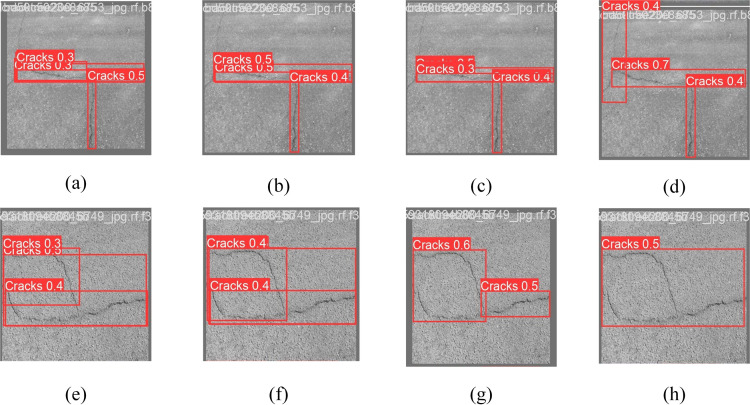
Comparison of different lightweighting models of YOLOv8n.

A graph of the lightweighting test results is shown in Fig 13. From the graph, it can be seen that Fig 13(d) and 13(h) have the best detection result, which fully proves the advantage of Slim-Neck improvement. This comparative experimental result graph illustrates the performance of four distinct neck structures: Primitive Structure, BiFPN, CSPNeck, and Slim-Neck, in the fracture detection test from left to right. The primitive structure depicted in Fig 13(a) serves as a baseline model but exhibits significant fine crack leakage; BiFPN in Fig 13(b) continues to experience some localization bias, despite enhanced detection capabilities through cross-scale feature fusion; CSPNeck in Fig 13(c) demonstrates diminished detection efficacy for fine cracks while preserving high computational efficiency; Slim-Neck in Fig 13(d) exhibits superior overall performance, accurately identifying the smallest cracks and effectively mitigating background interference such as pavement texture, thereby achieving an optimal balance between detection accuracy and computational efficiency, which underscores the advantages of its GSConv and VoVGSCSP modules in feature extraction and computational optimization. Fig 13(e)–13(h)) demonstrate its exceptional efficacy in addressing complex cracks, with only Fig 13(h) accurately identifying the cracks, further highlighting the remarkable performance of Slim-Neck.

### Effect of different loss functions on model performance

Given that various loss functions influence model performance differently, the experimental results illustrating the effects of the three variations of the WIOU loss function on the model, alongside comparative analyses with CIOU and GIOU, are presented in Table 5. As can be seen from the table, the precision rate and recall rate of GIOU, WIOUv1, WIOUv2, and WIOUv3 have different degrees of increase, but due to the GIOU is too concerned about the minimum closed rectangle, resulting in the overlap area between the predicted bounding box and the real bounding box is small resulting in a decrease of 0.2% in its mAP50-95. WIOUv3, although the increase in the regression rate is smaller, the overall WIOUv3 has a stronger performance and the highest precision rate and weighted average accuracy. WIOUv1 primarily centers on the IoU between the predicted bounding box and the ground-truth bounding box. However, it is insufficient in directly optimizing the target positioning accuracy, thereby causing a 0.5% reduction in mAP@50–95. As can be seen from the table, the WIOUv2 loss function exhibits poor performance in terms of recall and average precision improvement. WIOUv3’s P, R, mAP@50, mAP@50–95, and are improved by 2.6%, 0.4%, 0.6%, and 1.6%, respectively. Compared to CIOU, the detection rate is also superior to the other loss functions, indicating that the use of WIOUv3 has the same performance as the other loss functions, and the increase in the regression rate of WIOUv3 is not significant. The application of the WIOUv3 loss function enhances the model’s convergence rate and regression precision.

**Table 5:** Performance comparison of YOLOv8n adding different loss functions.

Models	P%	R%	mAP@50%	mAP@50–95%
**YOLOv8n+CIOU**	93.2	92.0	93.8	78.5
**YOLOv8n+GIOU**	93.7	92.3	94.2	78.3
**YOLOv8n+WIOUv1**	93.5	92.4	93.9	78.0
**YOLOv8n+WIOUv2**	93.9	92.3	94.8	78.5
**YOLOv8n+WIOUv3**	95.8	92.4	95.0	80.1

**Fig 14:**
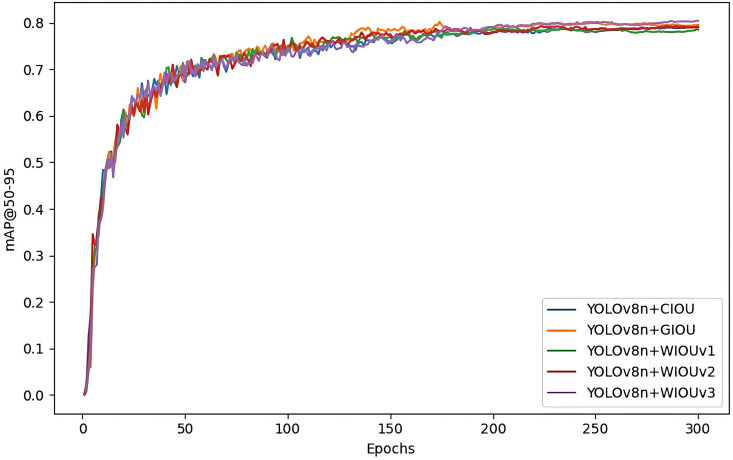
YOLOv8n adds different loss function training process curve change plot.

**Fig 15:**
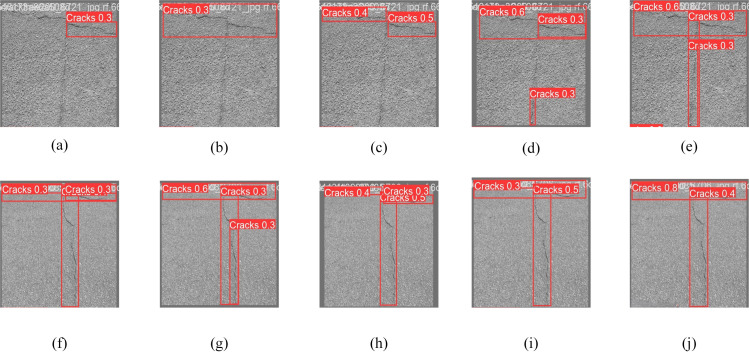
Graph of YOLOv8n detection results with different loss functions.

To further substantiate the detection efficacy of various loss functions in the YOLOv8n model, the training process of the YOLOv8n model with different incorporated loss functions is visualized. The figure demonstrates that the average accuracy of YOLOv8n+WIOUv3 is superior, consequently confirming the efficacy of the WIOUv3 loss function, as shown in Fig 14. Fig 15 displays the graphs of various loss function detection findings, arranged in the sequence of CIOU, GIOU, and WIOU (v1-v3).

### Ablation experiments

This study employs YOLOv8n as the baseline model to systematically assess the specific impact of each enhancement module on algorithm performance, maintaining strict control over experimental variables and utilizing an incremental integration strategy to sequentially introduce each module. The efficacy of the four proposed improvement strategies is thoroughly validated through the creation of 15 sets of controlled trials. Table 6 presents the quantitative analysis results of the ablation studies, wherein each experimental group utilizes the identical training set and employs a standardized evaluation metric to ensure result comparability. This meticulous experimental design quantifies the individual contribution of each module and elucidates the synergistic interactions among them.

**Table 6:** Results of ablation experiments.

Group	C2f_CBAM	SPPFCSPC	WIOUv3	Slim-Neck	mAP@50%	mAP@50–95%	Params/10^6^	GFLOPs
**1**	–	–	–	–	93.8	79.0	3.1	8.3
**2**	**√**				94.5	79.1	3.2	12.7
**3**		√			95.8	79.8	3.8	13.2
**4**			√		95.0	80.1	3.3	11.2
**5**				√	94.0	79.3	2.5	6.8
**6**	√	√			95.1	79.5	3.5	15.2
**7**	√		√		95.5	79.6	3.3	13.5
**8**	√			√	95.7	79.9	2.7	11.0
**9**		√	√		96.0	80.0	4.0	15.8
**10**		√		√	95.2	79.4	2.6	8.8
**11**			√	√	95.4	79.2	2.4	7.5
**12**	√	√	√		95.8	80.2	4.7	16.1
**13**	√	√		√	95.2	79.7	2.8	12.6
**14**	√		√	√	95.0	79.8	2.7	13.4
**15**		√	√	√	94.9	79.6	2.6	10.4
**16**	√	√	√	√	95.6	80.7	2.9	13.7

The systematic examination of the ablation experimental data revealed that the sixteenth group (C2f_CBAM + SPPFCSPC + WIOUv3 + Slim-Neck) had substantial benefits in several important metrics, establishing it as the superior scheme with the most comprehensive performance. Initially, regarding detection accuracy, the combination achieves a mAP@50−95% of 80.7%, which not only surpasses the baseline model by 1.7 percentage points (79.0%) but also exceeds all other combinations, particularly those utilizing only select modules (e.g., 80.0% in Group 9 or 80.2% in Group 12), indicating that multi-module synergy enhances model performance more holistically. Despite its mAP@50% (95.6%) being somewhat inferior to Group 3 (95.8%) and Group 9 (96.0%), its performance exhibits more stability under rigorous IoU criteria (0.5–0.95), rendering it more appropriate for the task of roadway surface disease detection in intricate scenarios.

Secondly, regarding model efficiency, the 16th group contains merely 2.9 M parameters, which is not only below the baseline of 3.1 M but also significantly lower than other high-precision configurations (e.g., 4.0 M in the 9th group and 4.7 M in the 12th group). This reduction is ascribed to the Slim-Neck’s lightweight design, which effectively minimizes redundant parameters. Simultaneously, its computational cost (13.7 GFLOPs) is more efficient than other high-performance combinations (e.g., 15.8 GFLOPs for group 9, 16.1 GFLOPs for group 12), although it is elevated compared to the baseline (8.3 GFLOPs), indicating that the scheme can sustain a high inference speed despite constrained computational resources.

Third, the synergistic impact of the module is significant. The enhancement in mAP@50–95% was minimal when employing C2f_CBAM or SPPFCSPC independently (Groups 2 and 3), yielding results of 79.1% and 79.8%, respectively. Subsequently, the integration of WIOUv3 and Slim-Neck elevated performance to 80.7%, underscoring the important contributions of WIOUv3’s dynamic loss optimization and Slim-Neck’s effective feature fusion. In contrast to Group 12 (lacking Slim-Neck), Group 16 attained a 0.5% superior mAP@50–95% while decreasing the parameter count by 38%, underscoring the optimization potential of Slim-Neck.

Fourth, the combination attains an ideal equilibrium between precision and efficiency. In comparison to Group 15, Group 16 exhibits a 1.1% enhancement in accuracy, accompanied by a mere increase of 3.3 GFLOPs in computational cost. When contrasted with Group 11 (WIOUv3 + Slim-Neck, mAP@50–95% 79.2%), it demonstrates a notable accuracy improvement of 1.5% and a reasonable escalation in GFLOPs (13.7 versus 7.5), indicating that the approach does not excessively augment the computational load while optimizing detection performance.

In conclusion, through the optimized integration of multiple modules, Group 16 achieves the best trade-off across three key dimensions: detection accuracy, parameter efficiency, and computational performance. This makes it the optimal solution for lightweight yet high-performance collaborative optimization in road surface crack detection models.

**Fig 16:**
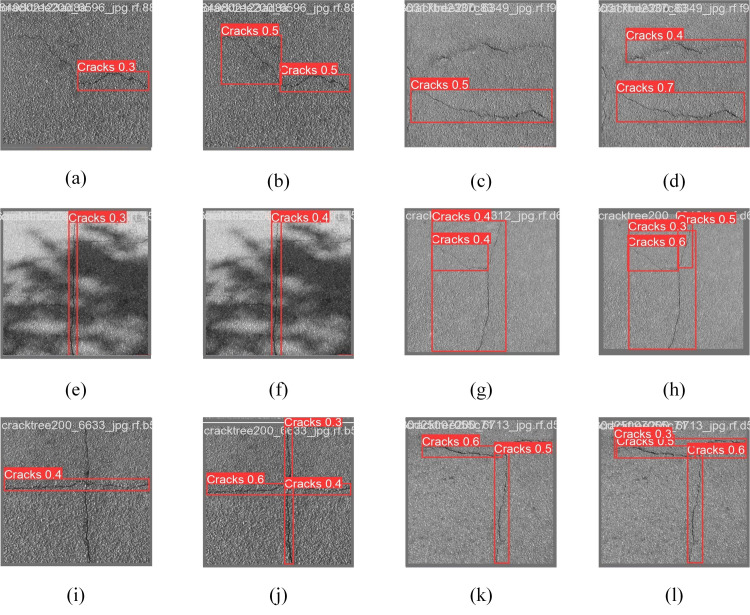
Comparison chart of the ablation test detection.

Fig 16 illustrates a comparison of pavement crack readings to elucidate the detection efficacy of the upgraded model. The figure indicates that the enhanced images typically produce increased detection confidence, for instance, rising from 0.3 to 0.5 for groups (a) and (b), and broaden the effective detection range. The improved model diminishes leakage detection in groups (c) and (i), and the comparative images (i) and (j) demonstrate that the model identifies all the fractures in the image. In the comparison group of (e) and (f), the precision of fracture identification continues to enhance despite increased shadow occlusion. Simultaneously, the enhancement algorithm augments the visual effect, revealing sharper fracture edges, more detailed crack morphology, and enhanced continuity, particularly in groups (g), (h) with (k), (i), where the improvement in crack recognition is most pronounced. The aforementioned comparisons demonstrate that the enhanced model exhibits superior performance in crack identification compared to the original model.

### Comparative experiments

To further validate the superiority of the enhanced method, the refined model is compared with prominent models from previous years. Several more mature models, Fast R-CNN, YOLOv5n, YOLOv6n, YOLOv7-tiny, and YOLOv8n, are selected for this experiment to compare with the improved YOLOv8n model in this paper with the same dataset. In this section, recall, mAP@50, mAP@50–95, the number of floating-point computations, and the number of parameters are used as evaluation metrics to assess the performance of the models. The experimental results are shown in Table 7.

**Table 7:** Performance comparison results of different algorithms.

Module	R%	mAP@50%	mAP@50–95%	Params/10^6^	GFLOPs
**Fast R-CNN**	71.15	76.1	67.4	18.9	29.1
**YOLOv5n**	89.6	91.0	79.3	4.3	14.8
**YOLOv6n**	90.8	93.2	75.6	17.2	15.9
**YOLOv7-tiny**	92.5	93.5	78.3	10.2	14.1
**YOLOv8n**	92.4	93.8	79.0	3.0	8.1
**Improved YOLOv8n**	94.2	95.6	80.7	2.9	7.6

Table 7 demonstrates that the two-stage algorithm Fast R-CNN exhibits significantly poorer detection accuracy compared to single-stage detection. Its mAP@50 is 76.1%, and it also has a large amount of computation, which cannot meet the current detection requirements. In comparison to Fast R-CNN, YOLOv5n exhibits a substantial decrease in both floating-point operations and parameter count, alongside enhancements in metrics such as R, mAP@50, and mAP@50–95; nonetheless, it demonstrates limited robustness in intricate backgrounds. YOLOv6n has superior detection accuracy compared to YOLOv5n; nonetheless, it entails more computational demands and an increased number of parameters, rendering it unsuitable for real-time detection. The overall performance of YOLOv7-tiny is slightly insufficient. Its R is 92.4%, which is higher than that of YOLOv8n, but the values of mAP@50 and mAP@50–95 are smaller. The improved result data in the table shows that, on the premise of a small increase in the amount of computation, this algorithm can significantly improve the average detection accuracy. And YOLOv8n is superior to other models in terms of accuracy, amount of computation, and number of parameters. The enhanced YOLOv8n presented in this study reduces computational demands and parameter count by 9.3% and 7.2%, respectively, while elevating mAP@50 to 95.6% and mAP@50–95 to 80.7%. This performance exceeds that of conventional and other single-stage models, demonstrating the superior overall efficacy of the proposed model in comparison to existing target detection algorithms.

**Fig 17:**
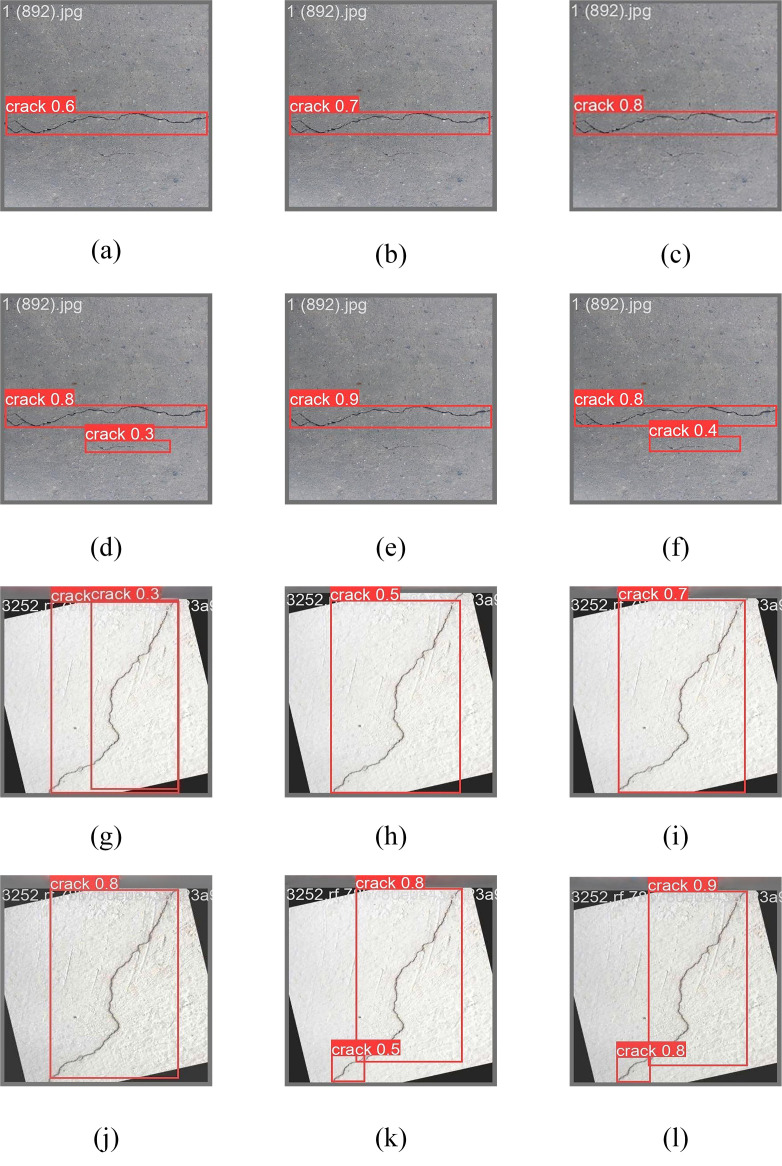
Comparison of the detection effect of different models.

Meanwhile, to verify the actual effect of the improved algorithms in this paper, the above several detection algorithms are visualized and compared, and the comparison of the detection effect is illustrated in Fig 17, which is Fast R-CNN, YOLOv5n, YOLOv6n, YOLOv7-tiny, YOLOv8n, and improved YOLOv8n in that order. It can be seen through the comparison that Fig 17(a)–17(c) and 17(e), the color of the cracks is highly similar to the surrounding environment, and the crack size is small, which causes the model to miss the smaller cracks. In Fig 17(d), although YOLOv7-tiny detects small cracks, the prediction value is lower than that of improved YOLOv8n in Fig 17(f), which indicates that the robustness of the model needs to be improved when dealing with small targets. In Fig 17g, the small spacing between the two cracks leads to low confidence and inaccurate bounding box localization in the detection results of Fast R-CNN. The detection results of the models in Fig 17(h), 17(i) and 17(g) detected two different cracks as continuous cracks. In Fig 17(k), although the bounding box localization of YOLOv8n is accurate, its confidence level is low, indicating that the generalization ability of the model is low. Fig 17(l) indicates that the improved YOLOv8n model successfully discriminates into two single cracks with high confidence, which shows that the improved model has obvious advantages over other models.

**Fig 18:**
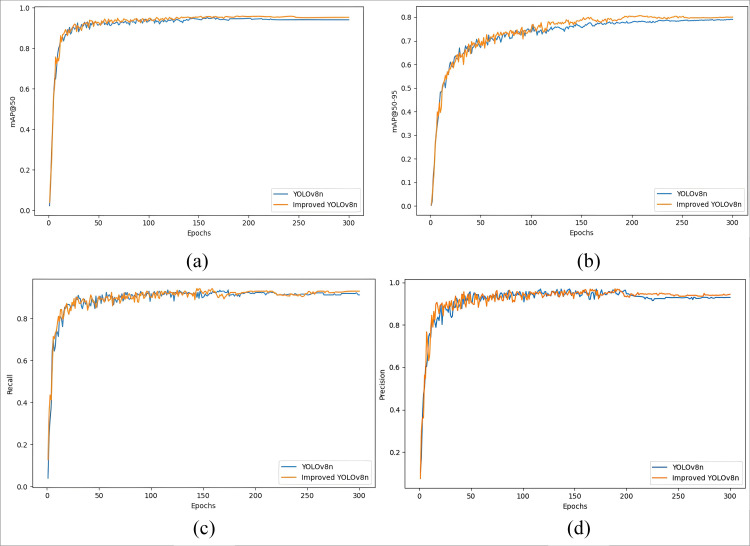
Comparison of mAP@50, mAP@50-95, Recall and Precision training visualizations.

This research illustrates the efficacy of the enhanced model by visualizing and comparing the variations in the metrics of mAP@50, mAP@50–95, P, and R for YOLOv8n and the improved YOLOv8n throughout the training process, with the experimental findings presented in Fig 18. The graphic illustrates that as the iterations increase, the two models converge, with the improved YOLOv8n yielding superior results compared to the benchmark model, indicating that the upgraded method is more effective in road crack identification.

### Effectiveness of crack detection

**Fig 19:**
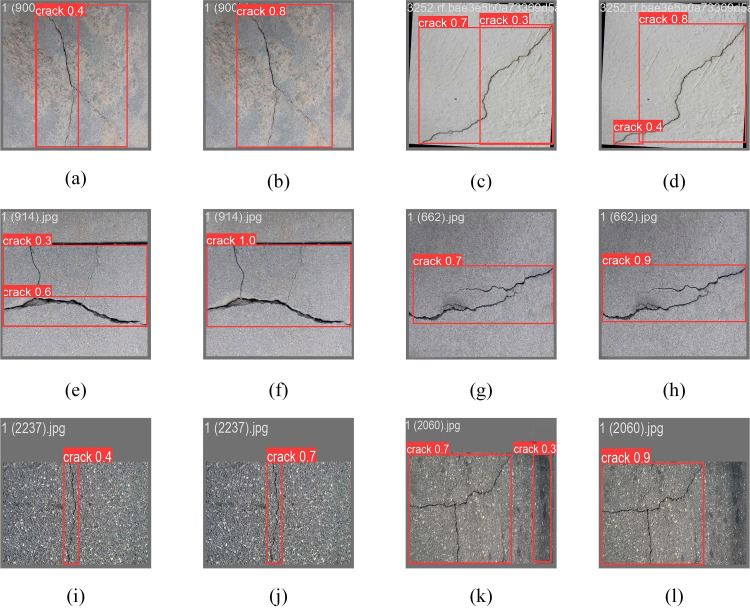
A comparison chart of the detection effect.

This study selects example test photos to illustrate the detection efficacy of the YOLOv8n and improved YOLOv8n models. The results of the visualization on the dataset are presented in Fig 19: (a), (c), (e), (g), (i), and (k) depict the original YOLOv8n model utilized in this work, while (b), (d), (f), (h), (j), and (l) illustrate the detection outcomes of the improved YOLOv8n model. In Fig 19(a), the original model incorrectly detects one continuous crack as two cracks. In contrast, in Fig 19(b), the improved model circumvents this false detection and shows higher accuracy. As can be seen in Fig 19(c), the original model cannot accurately detect two cracks after flipping the original image left and right, and is inaccurate in detecting small target cracks. On the contrary, the improved model detection, as shown in Fig 19(d), accurately locates the position of the two cracks while the accuracy is improved. Fig 19(e) shows that the original model incorrectly detects expansion joints as cracks, whereas in Fig 19(f), all cracks are precisely localized completely with a large improvement in accuracy. In Fig 19(g), the YOLOv8n model could not accurately depict the edge region of the cracks and could not detect the slight crack condition, while in Fig 19(h), the improved YOLOv8n model accurately identifies the edge region of the cracks, and although its detection accuracy is high but also suffers from the interference of the background detecting the two adjacent cracks as a single continuous crack. From Fig 19(i), it can be seen that the detection accuracy of cracks with similar color to the background is low under low light conditions, while Fig 19(j) shows that the enhanced model has improved detection accuracy under complex background conditions, which proves that the improved model can be applied to the detection of cracks with complex backgrounds. In Fig 19(k), the original model incorrectly categorizes the black graffiti in the background as cracks, and in Fig 19(l), the improved model avoids this incorrect detection and shows higher accuracy.

## Conclusion

In this paper, the improved YOLOv8n algorithm is proposed for the detection of apparent cracks on roads in complex scenes. The algorithm solves the problems of low accuracy and slow detection, as well as leakage and false detection of traditional algorithms in the target detection task. Initially, the C2f_CBAM module is constructed, which integrates the CBAM attention mechanism into the C2f module to improve the network’s fusion capabilities for multi-scale features and to more effectively address the obstacles presented by variations in target scales in the road background. Secondly, the SPPF module is supplanted by SPPFCSPC to enhance the fusion of various scale information and mitigate the effect of background interference on target detection. Third, the Slim-Neck paradigm is developed to diminish the computational complexity of the model and the intricacy of the network topology, while enhancing the network’s adaptability through the application of GSConv and VOV-GSCSP. Lastly, the WIOUv3 loss function is implemented to resolve the sample imbalance issue, thereby enhancing the localization accuracy and expediting the algorithm’s convergence. The enhancements result in an increase of 1.8%, 1.7%, and 1.8% in mAP@50, mAP@50–95, and Recall, respectively, when compared to the benchmark model YOLOv8n. The findings indicate that the model presented in this work surpasses the baseline and alternative models regarding average accuracy in road fracture identification, while also exhibiting a quicker detection speed and reduced computational requirements.

Despite the enhanced performance of the YOLOv8n algorithm, there remains significant potential for advancement in computational efficiency and real-time applicability. Future efforts will concentrate on optimizing the model’s lightweight characteristics to address more intricate real-world situations and to investigate additional avenues for practical applications. It is essential to gather data from various contexts and defect types to create a more extensive dataset, hence improving the model’s robustness and generalization capabilities; additionally, the incorporation of multimodal data fusion technology is required to address the demands of road surface disease detection.

## Supporting information

S1 Table Access to open-source datasets. (PDF)

S2 File Dataset. (ZIP)

S3 File Code. (ZIP)
