# Supplementary material for: Research on enhancing road apparent crack detection based on the improved YOLOv8n model
Source: PLoS One. 2025 Sep 4;20(9):e0330218. doi: 10.1371/journal.pone.0330218 (PMC12410743; doi:10.1371/journal.pone.0330218)
Supplement: S1 Table — (PDF) [file pone.0330218.s001.pdf]

S1 Access to open-source datasets

Dataset	Download Link
Crack forest	https://github.com/cuilimeng/CrackForest-dataset
Crack500	https://github.com/TachibanaYoshino/Road-Crack-Segmentation--Keras/tree/master/dataset/CRACK500
