# Supplementary figures and images for: Research on enhancing road apparent crack detection based on the improved YOLOv8n model (part 2 of 2)
Source: PLoS One. 2025 Sep 4;20(9):e0330218. doi: 10.1371/journal.pone.0330218 (PMC12410743; doi:10.1371/journal.pone.0330218)

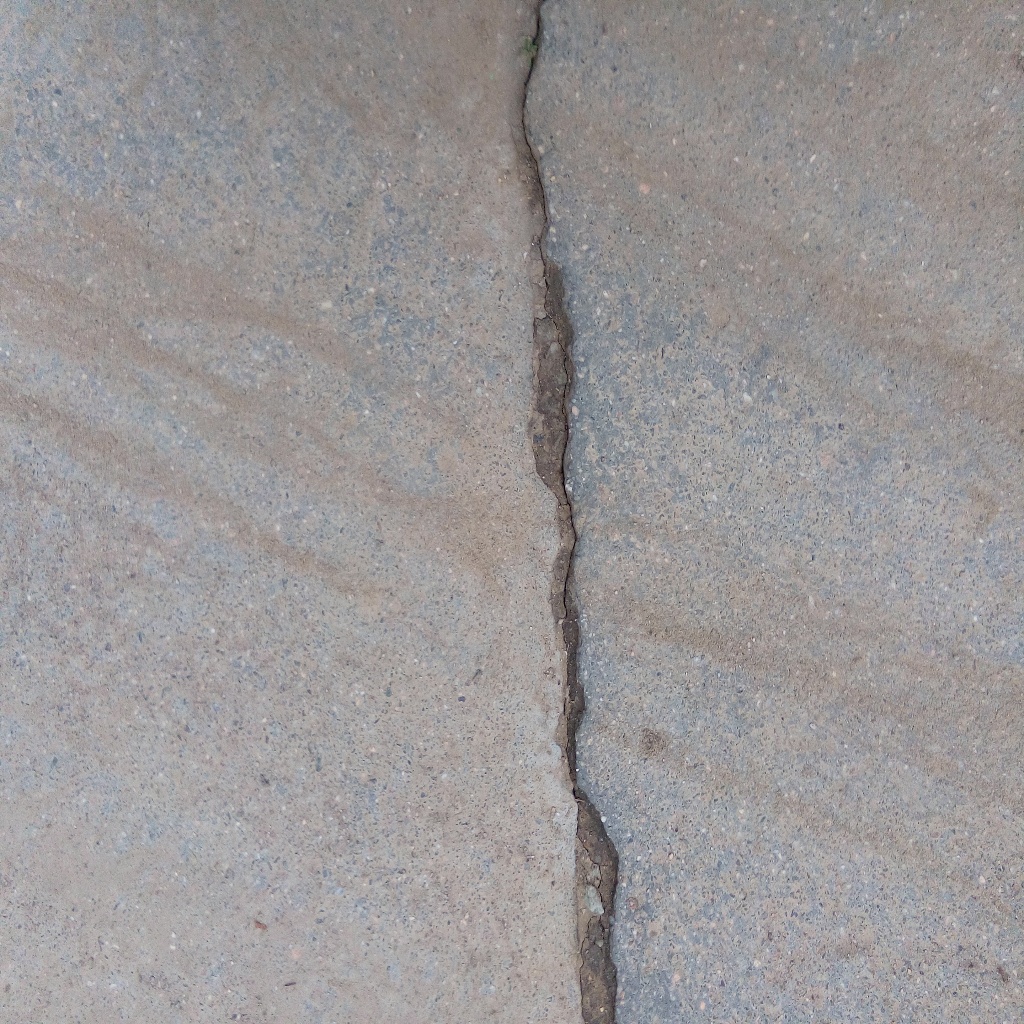

Supplement: S2 File — (ZIP) [file pone.0330218.s002.zip › 1 (299).jpg]

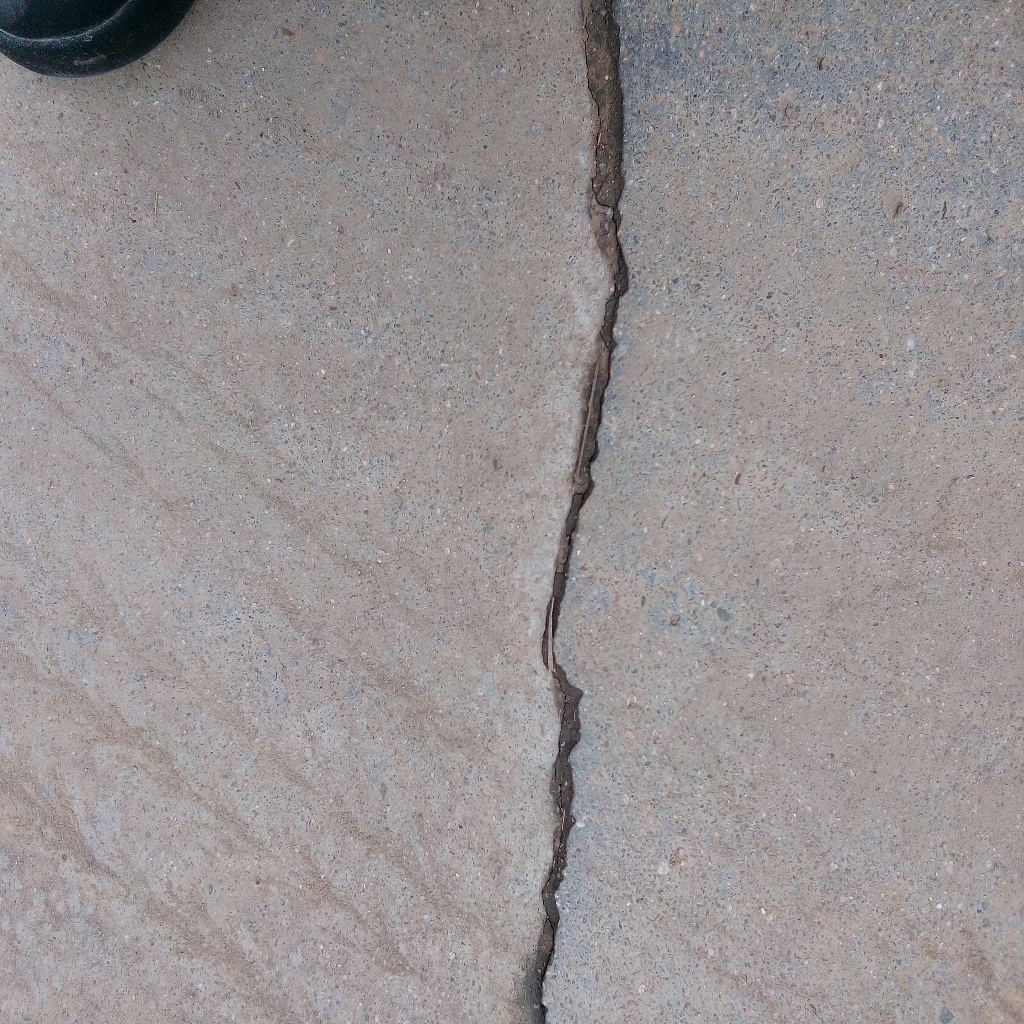

Supplement: S2 File — (ZIP) [file pone.0330218.s002.zip › 1 (300).jpg]

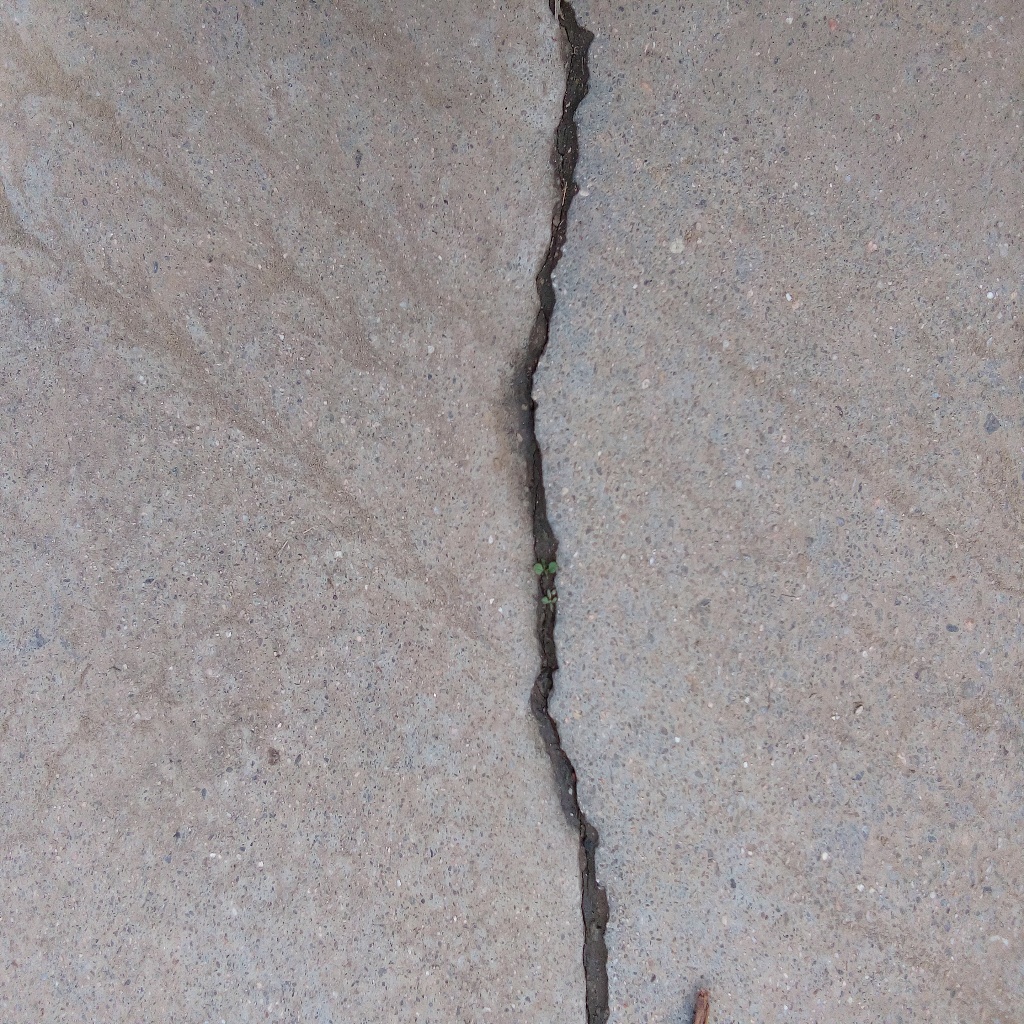

Supplement: S2 File — (ZIP) [file pone.0330218.s002.zip › 1 (301).jpg]

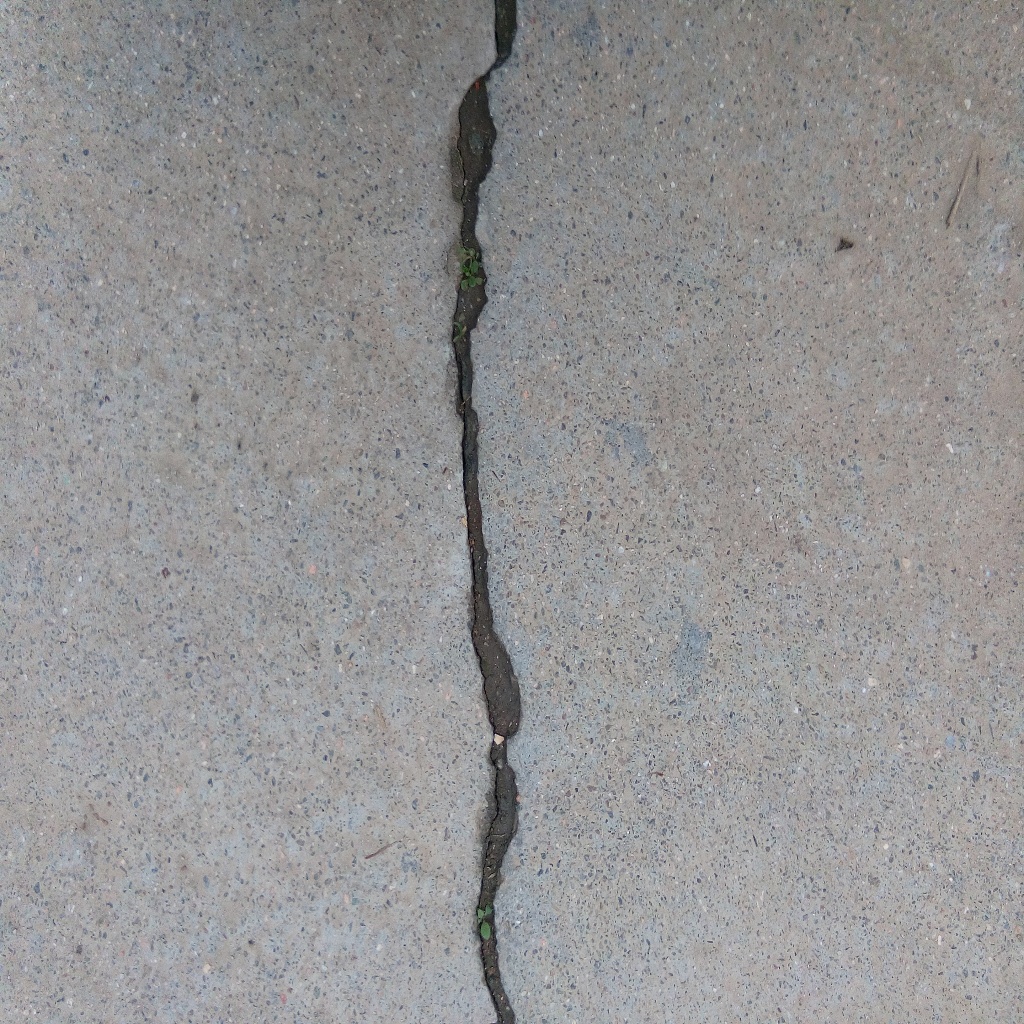

Supplement: S2 File — (ZIP) [file pone.0330218.s002.zip › 1 (302).jpg]

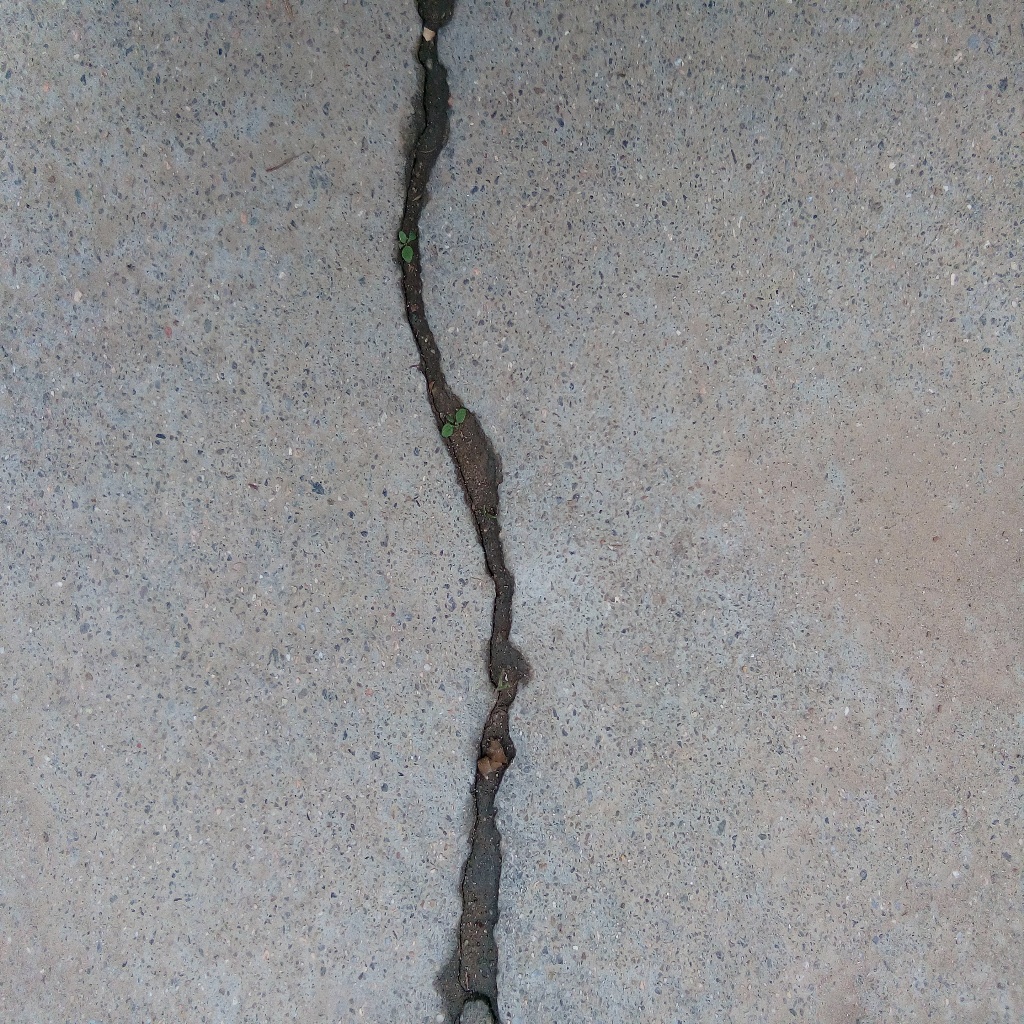

Supplement: S2 File — (ZIP) [file pone.0330218.s002.zip › 1 (303).jpg]

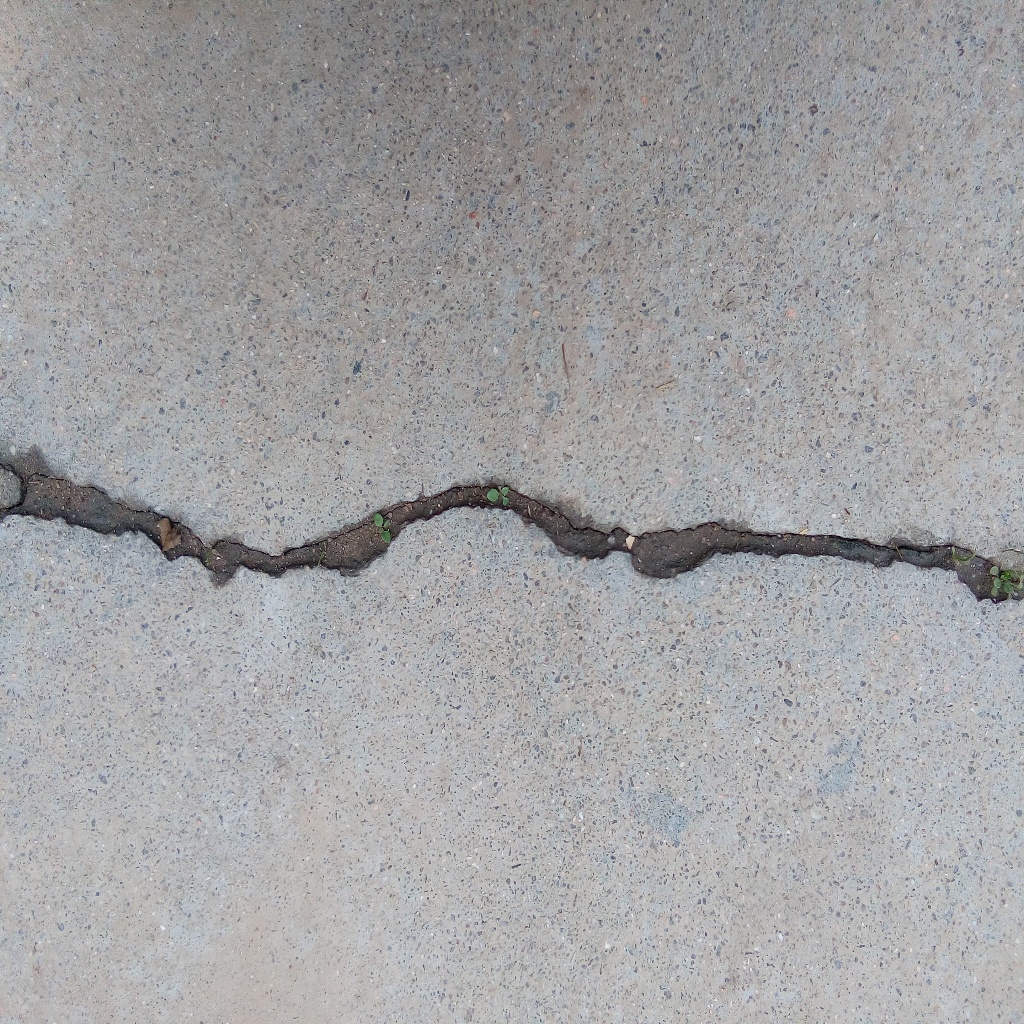

Supplement: S2 File — (ZIP) [file pone.0330218.s002.zip › 1 (304).jpg]

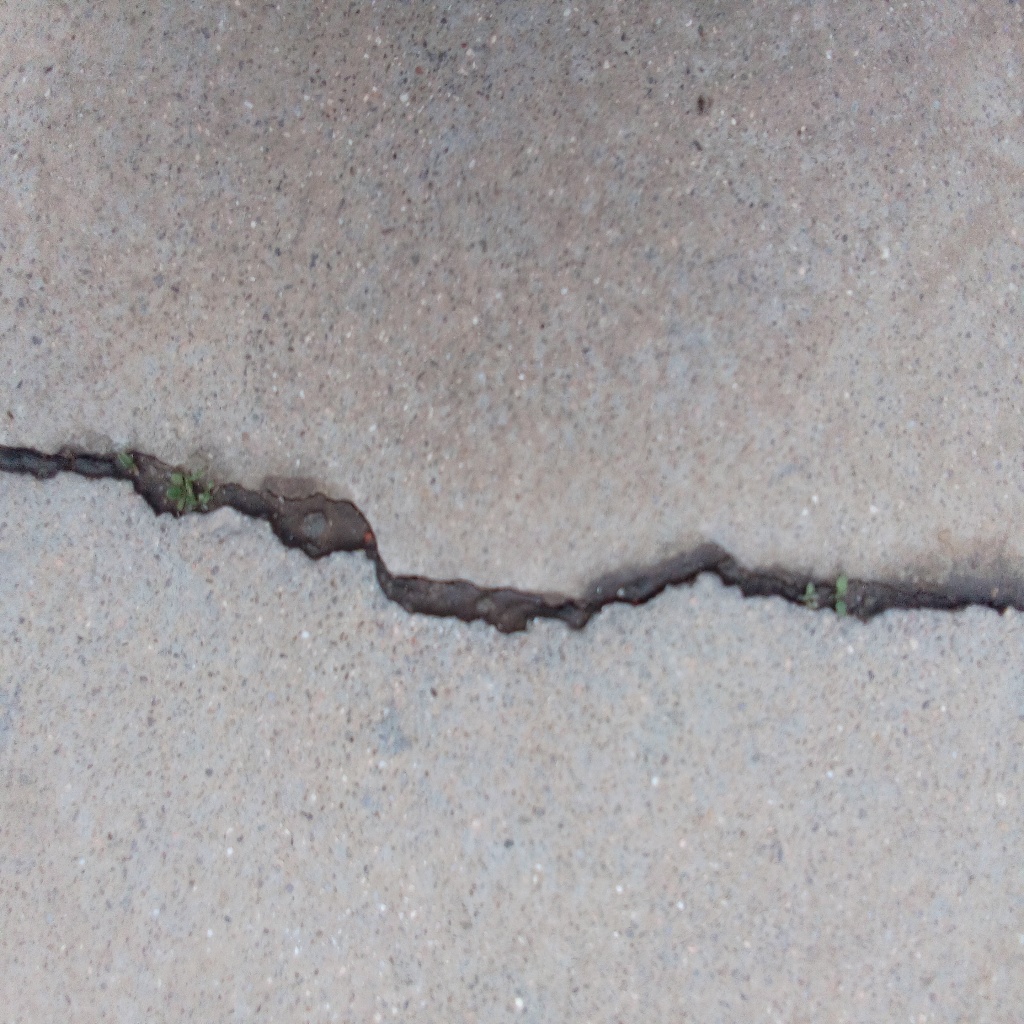

Supplement: S2 File — (ZIP) [file pone.0330218.s002.zip › 1 (305).jpg]

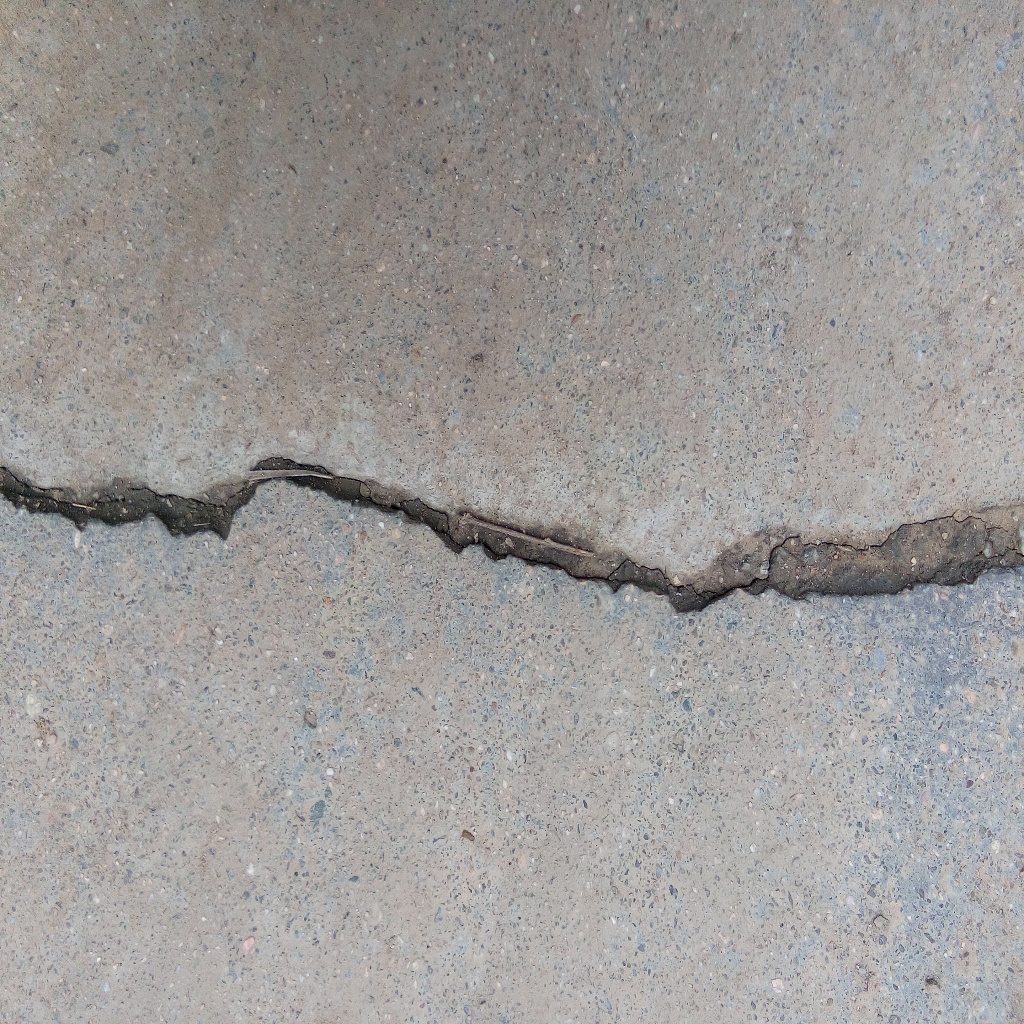

Supplement: S2 File — (ZIP) [file pone.0330218.s002.zip › 1 (306).jpg]

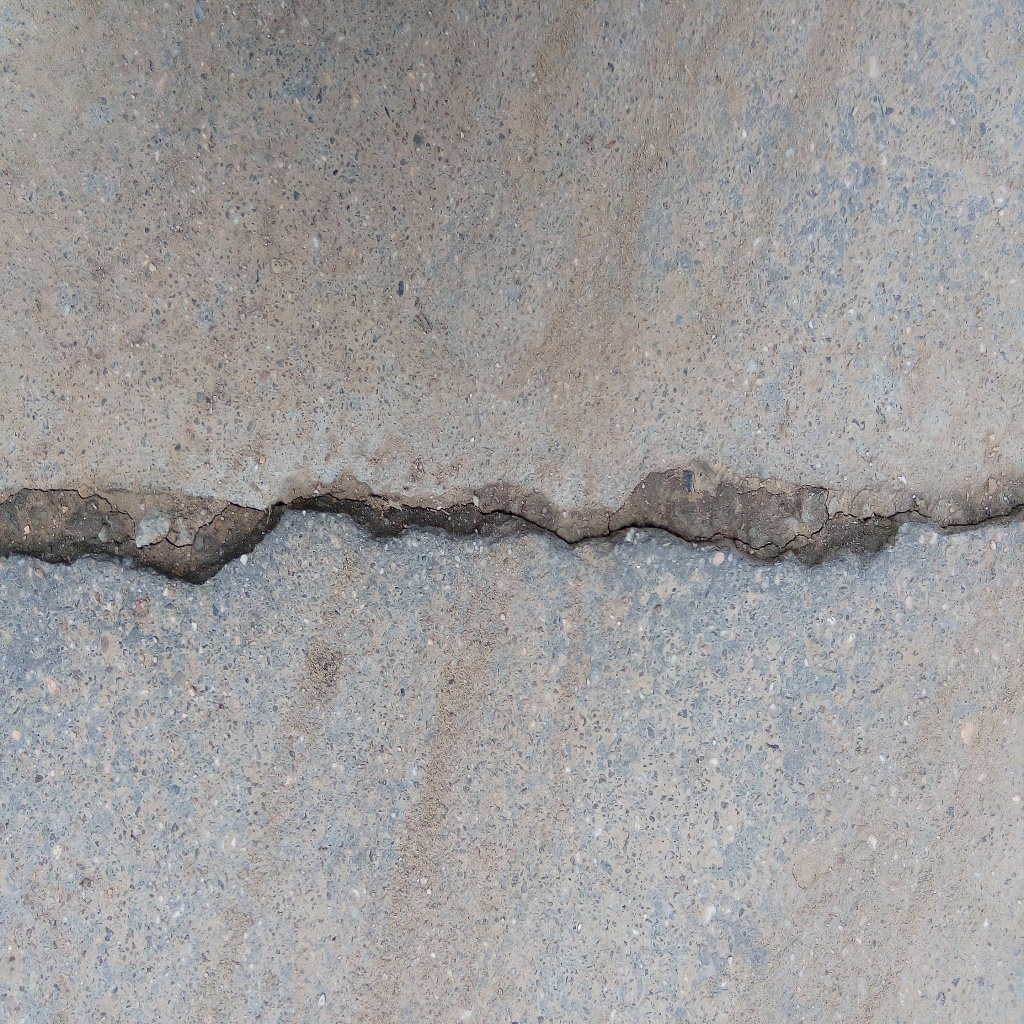

Supplement: S2 File — (ZIP) [file pone.0330218.s002.zip › 1 (307).jpg]

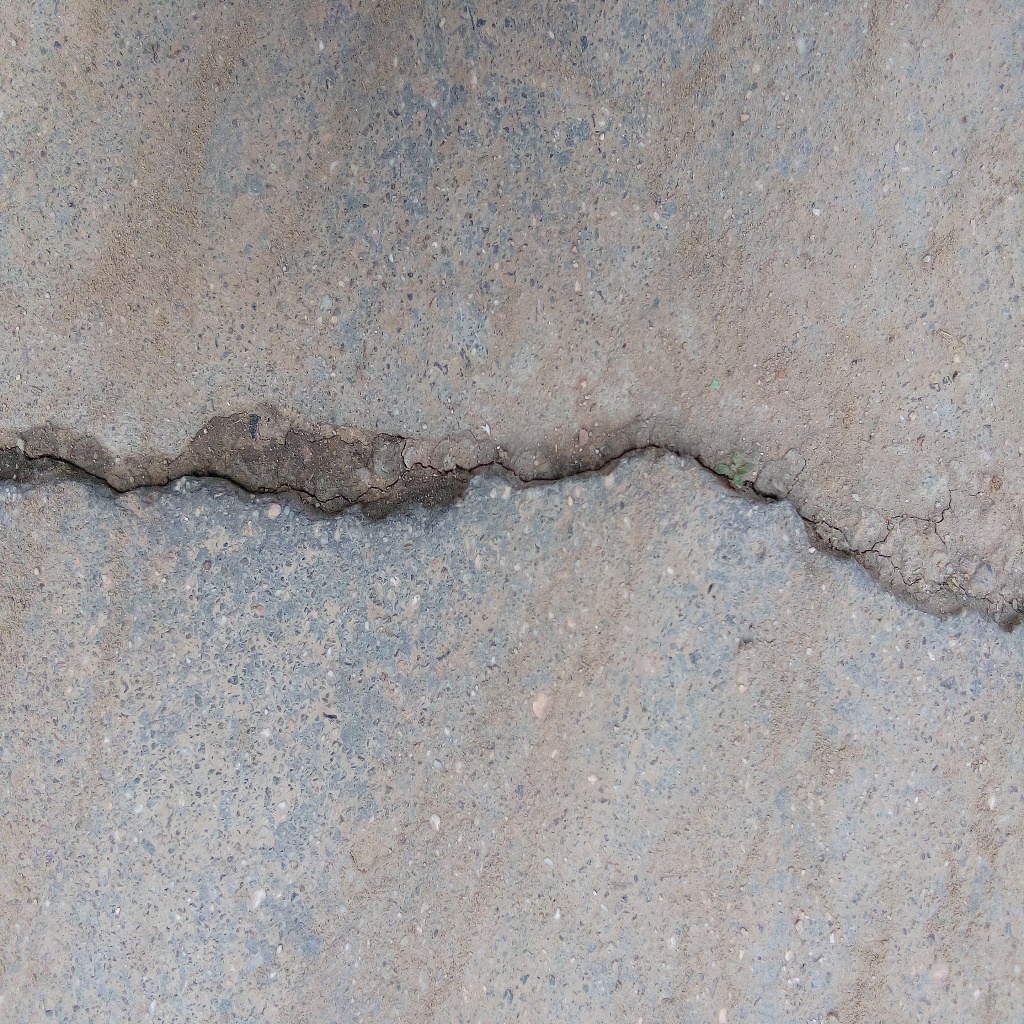

Supplement: S2 File — (ZIP) [file pone.0330218.s002.zip › 1 (308).jpg]

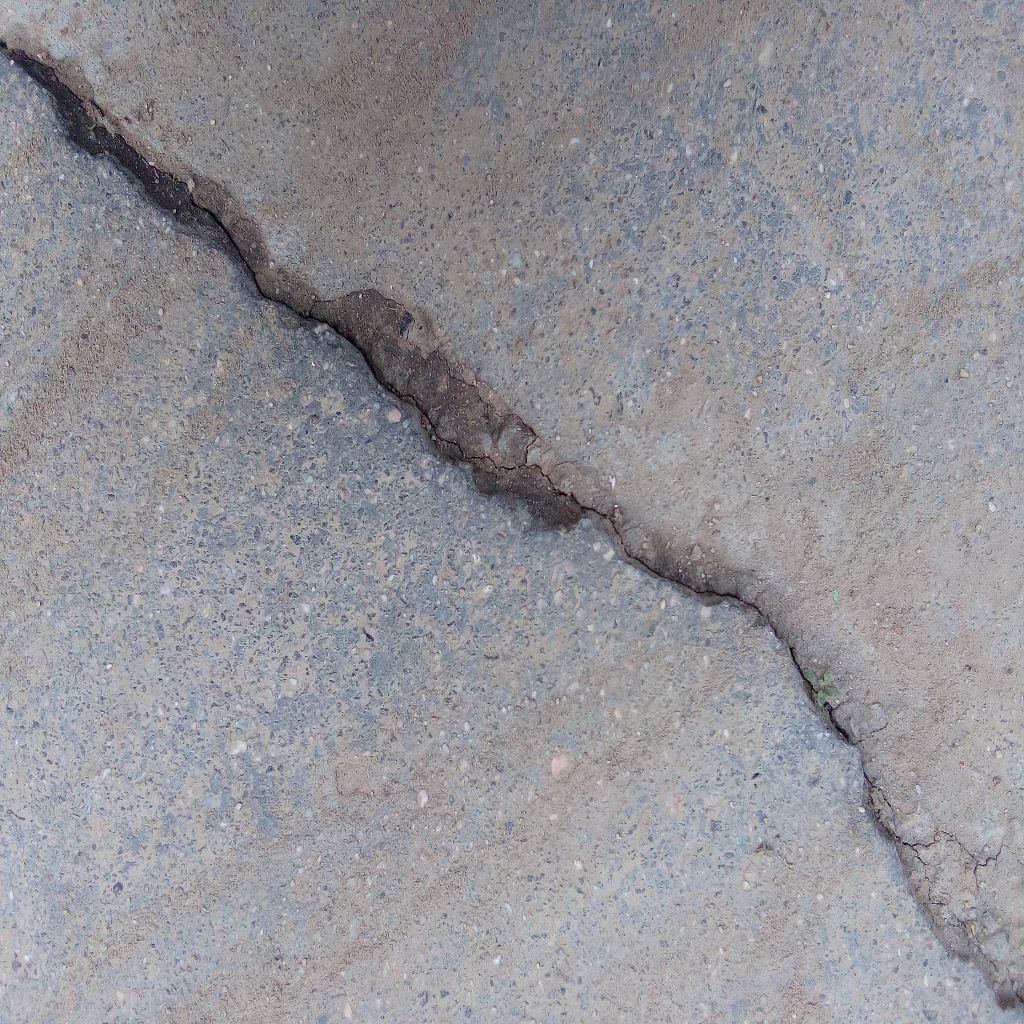

Supplement: S2 File — (ZIP) [file pone.0330218.s002.zip › 1 (309).jpg]

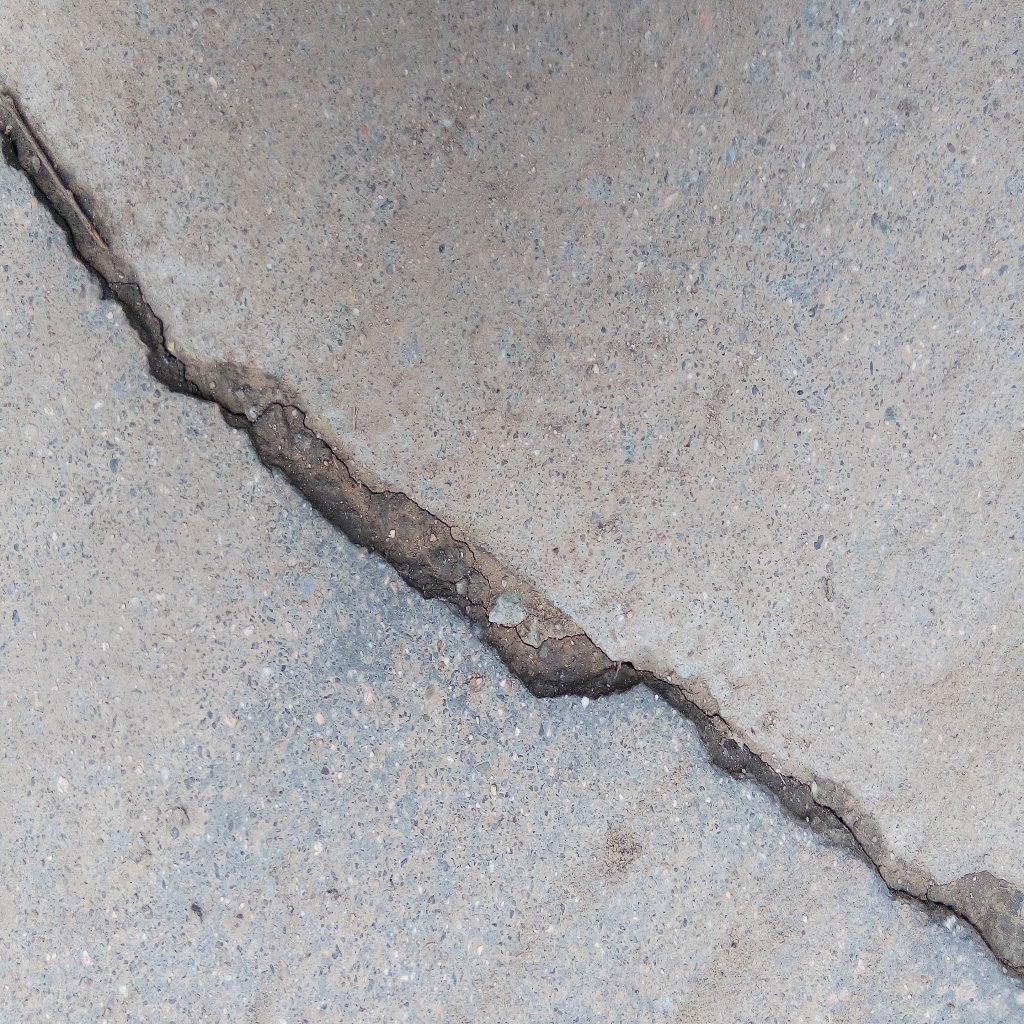

Supplement: S2 File — (ZIP) [file pone.0330218.s002.zip › 1 (310).jpg]

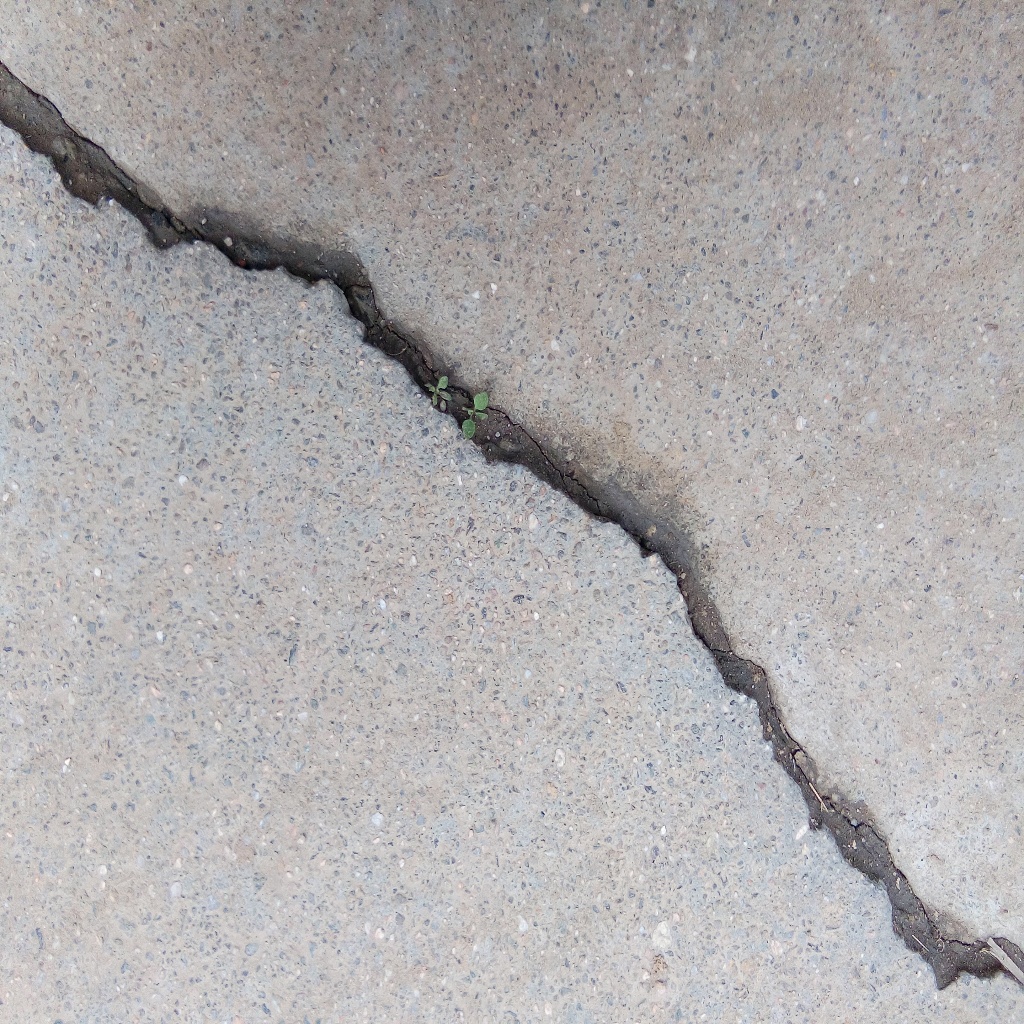

Supplement: S2 File — (ZIP) [file pone.0330218.s002.zip › 1 (312).jpg]

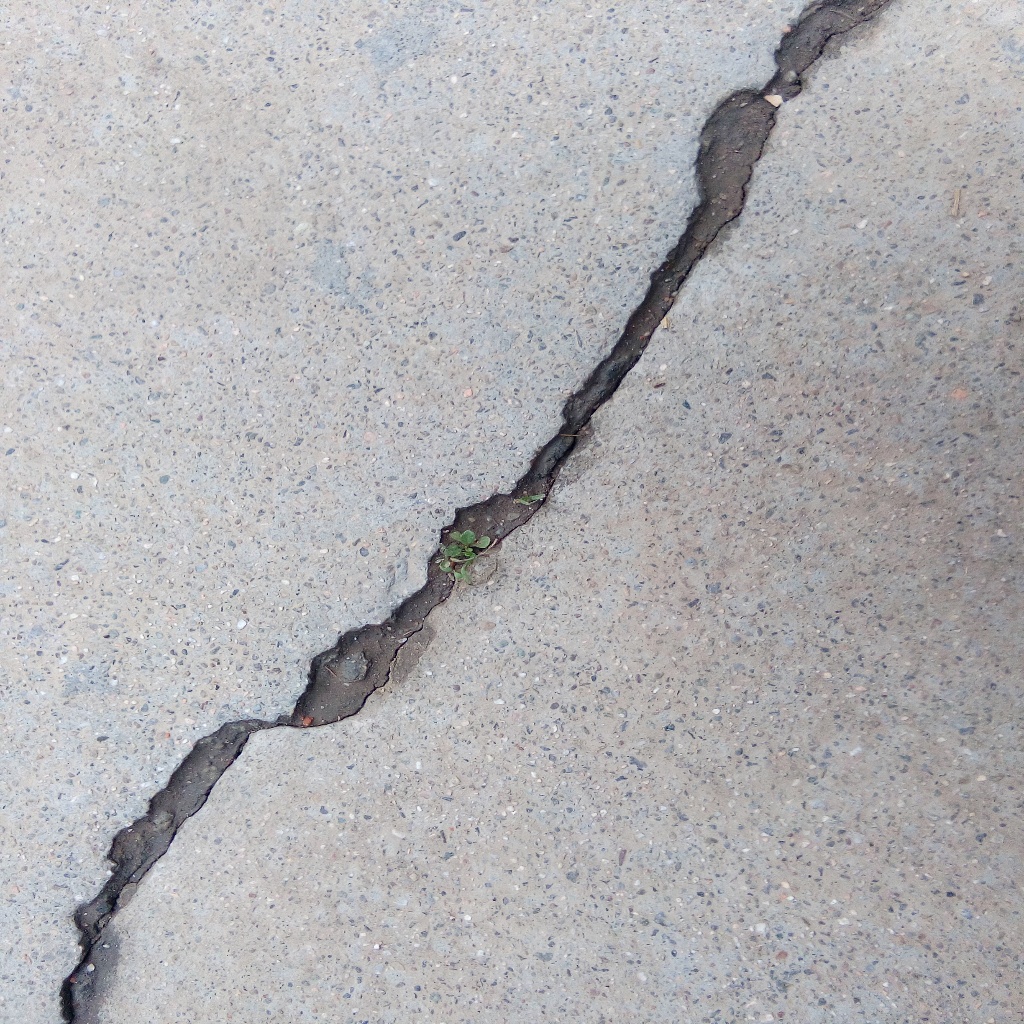

Supplement: S2 File — (ZIP) [file pone.0330218.s002.zip › 1 (313).jpg]

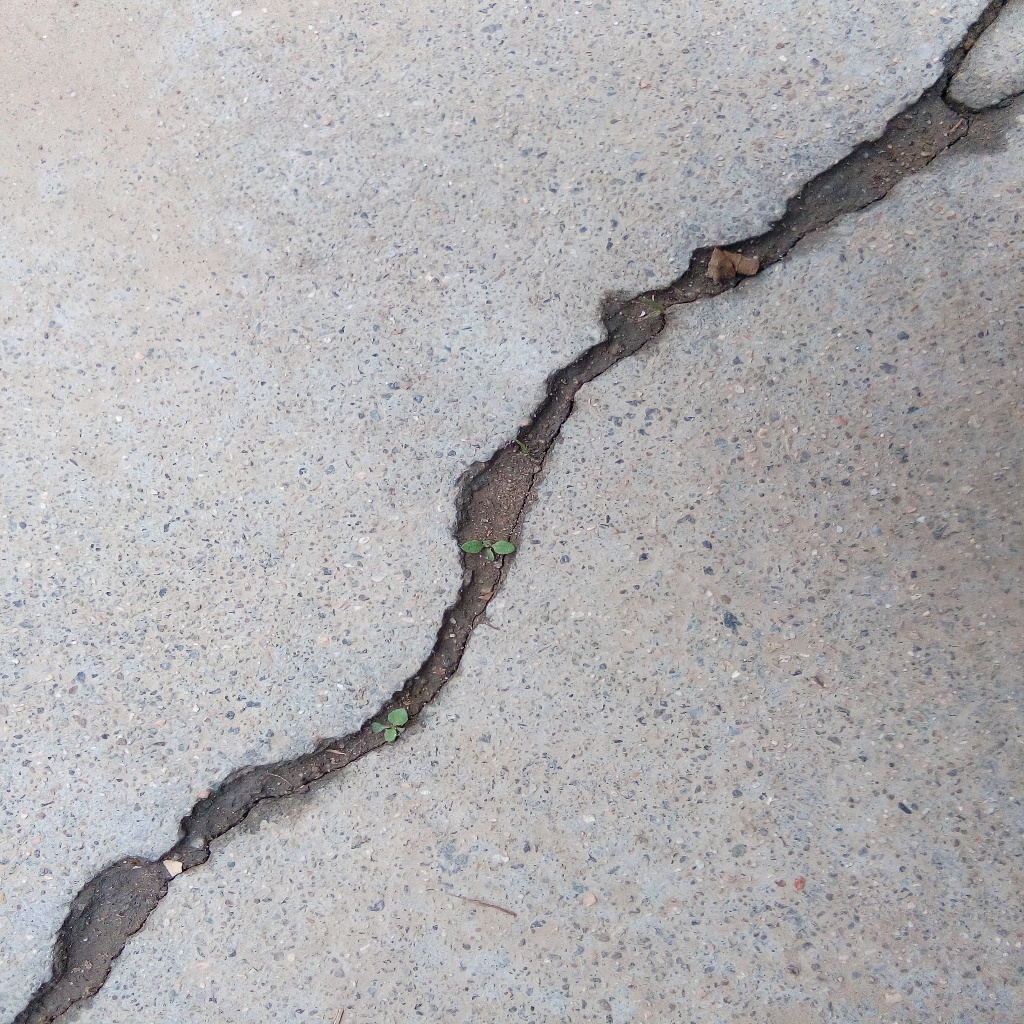

Supplement: S2 File — (ZIP) [file pone.0330218.s002.zip › 1 (314).jpg]

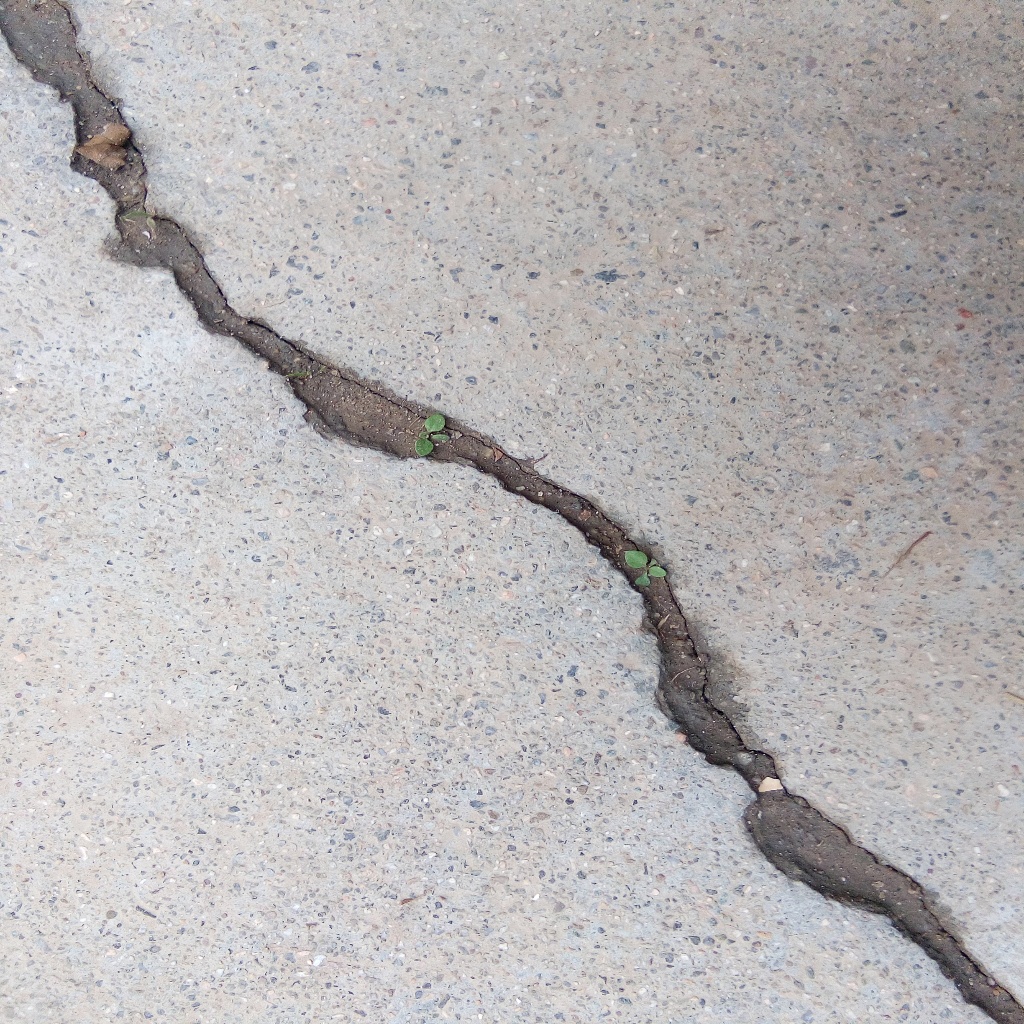

Supplement: S2 File — (ZIP) [file pone.0330218.s002.zip › 1 (315).jpg]

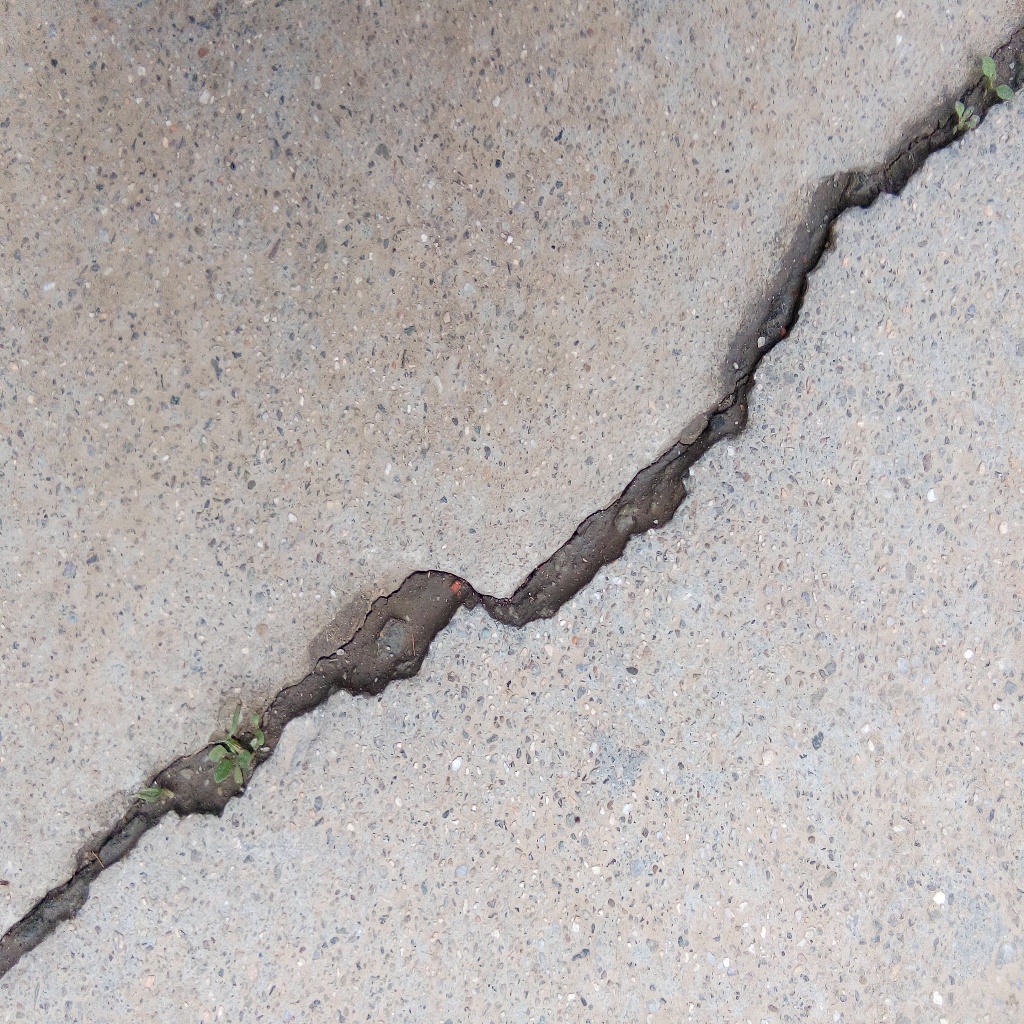

Supplement: S2 File — (ZIP) [file pone.0330218.s002.zip › 1 (316).jpg]

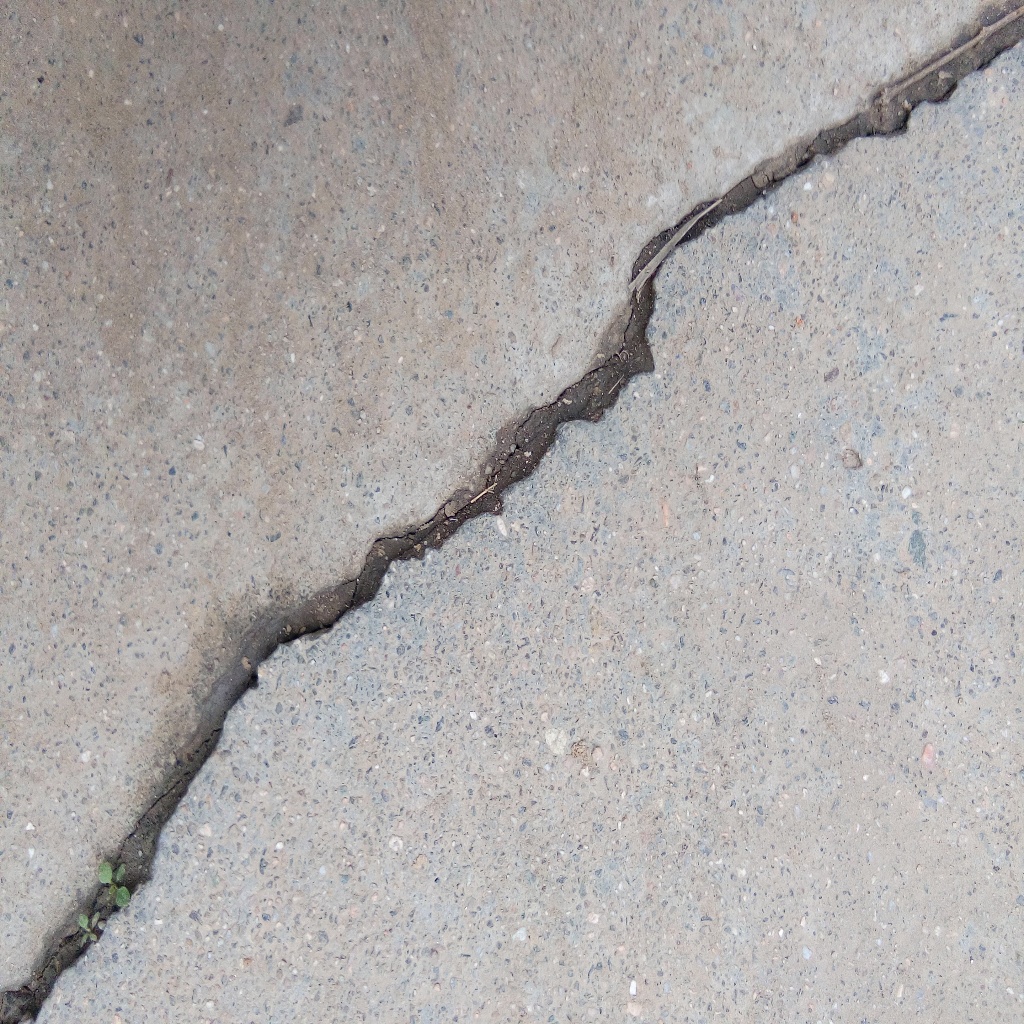

Supplement: S2 File — (ZIP) [file pone.0330218.s002.zip › 1 (317).jpg]

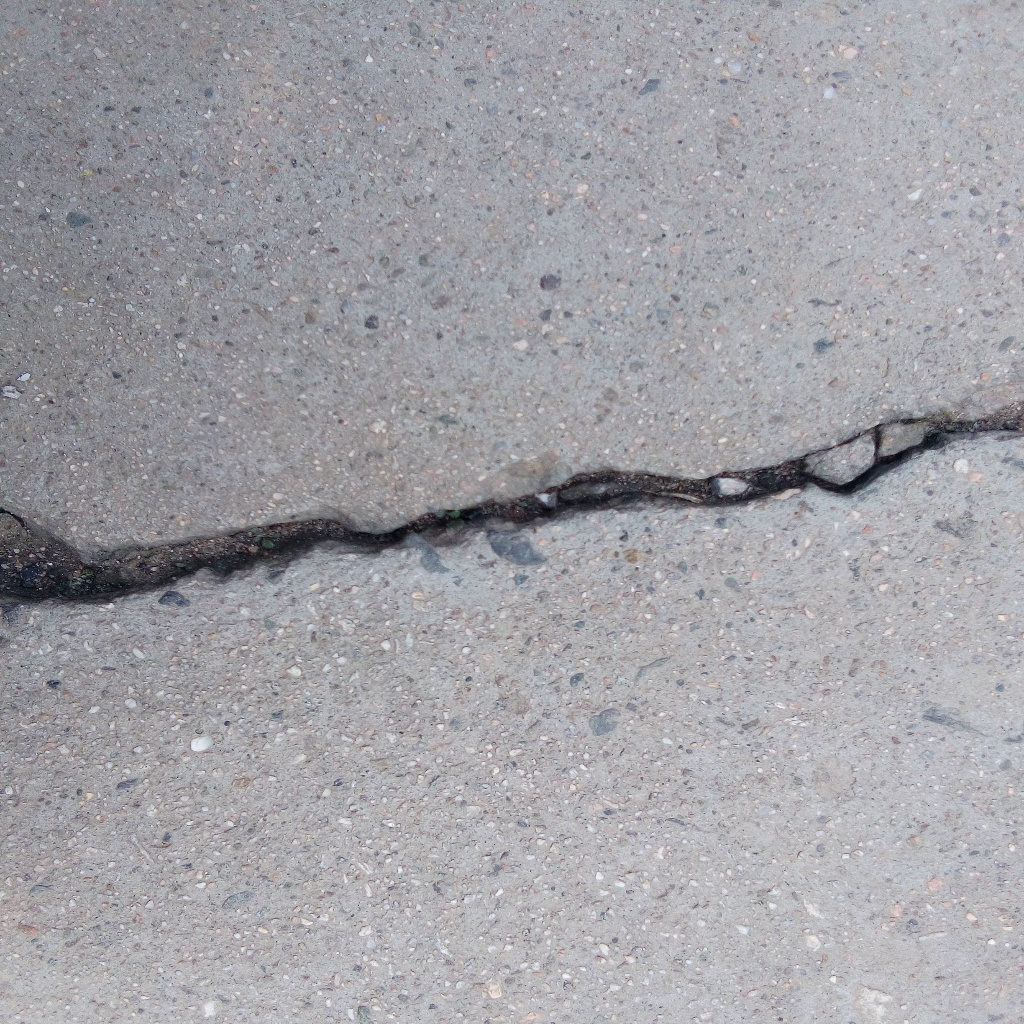

Supplement: S2 File — (ZIP) [file pone.0330218.s002.zip › 1 (319).jpg]

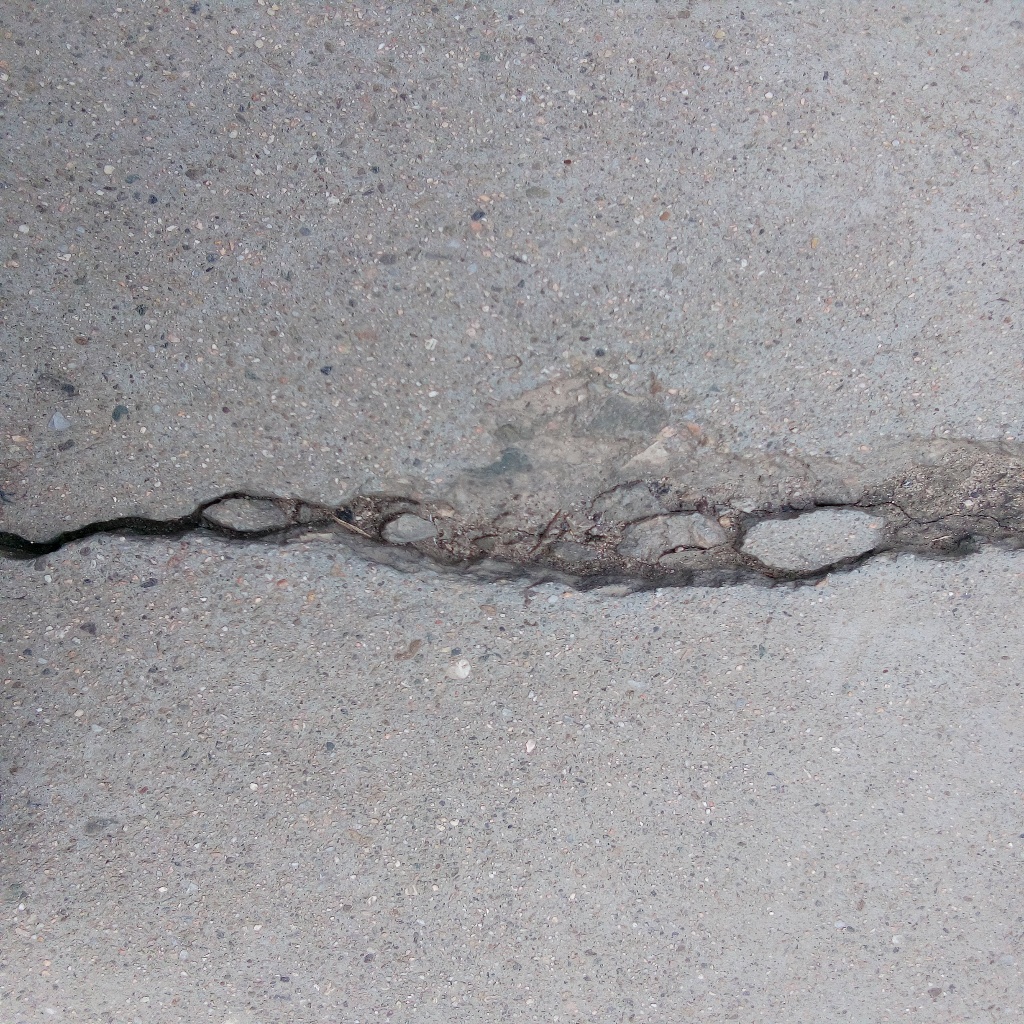

Supplement: S2 File — (ZIP) [file pone.0330218.s002.zip › 1 (320).jpg]

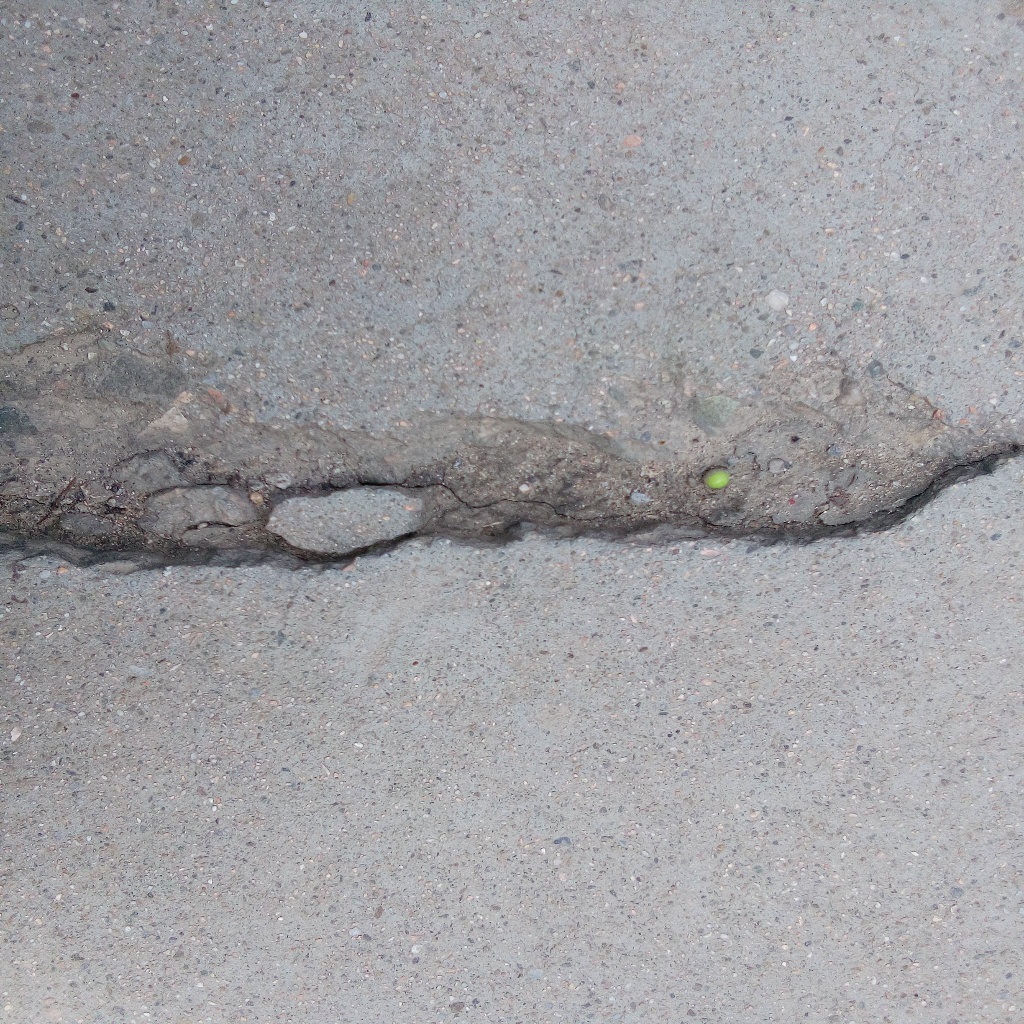

Supplement: S2 File — (ZIP) [file pone.0330218.s002.zip › 1 (321).jpg]

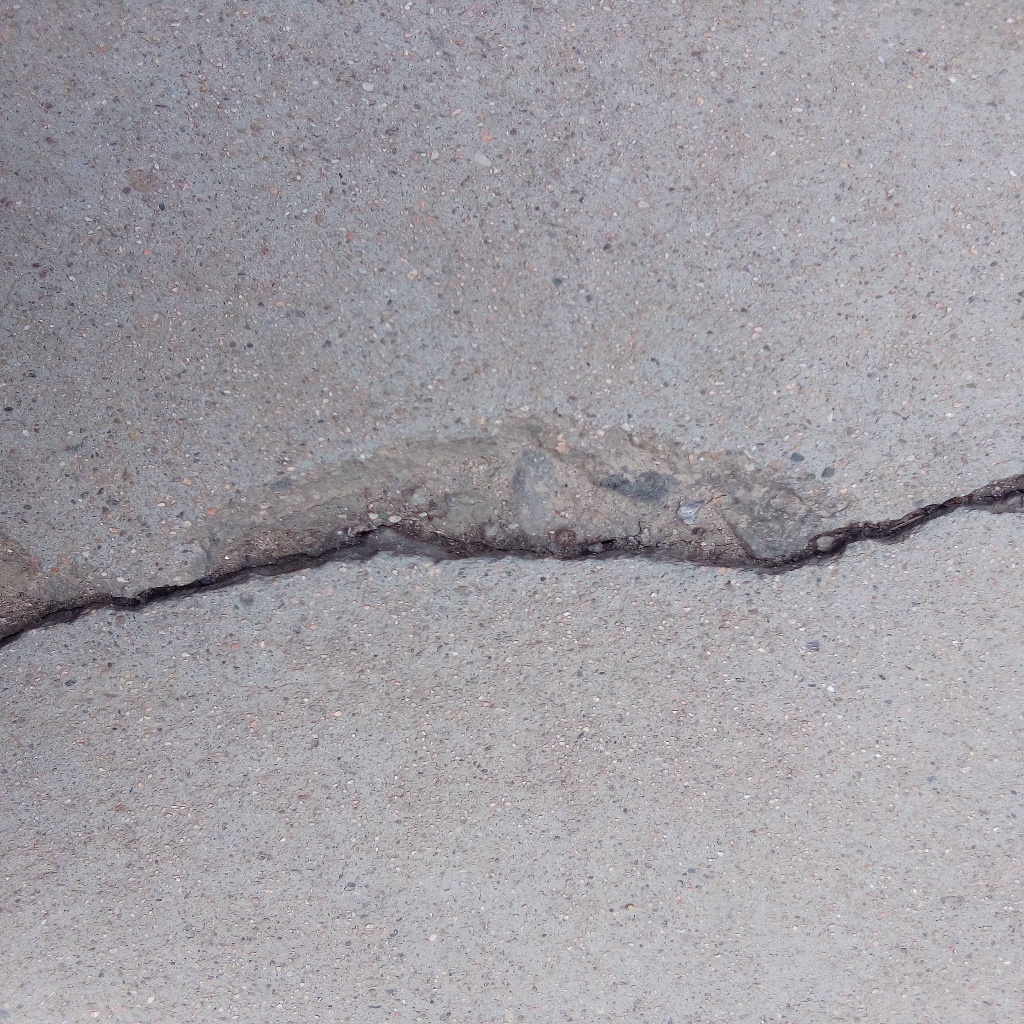

Supplement: S2 File — (ZIP) [file pone.0330218.s002.zip › 1 (322).jpg]

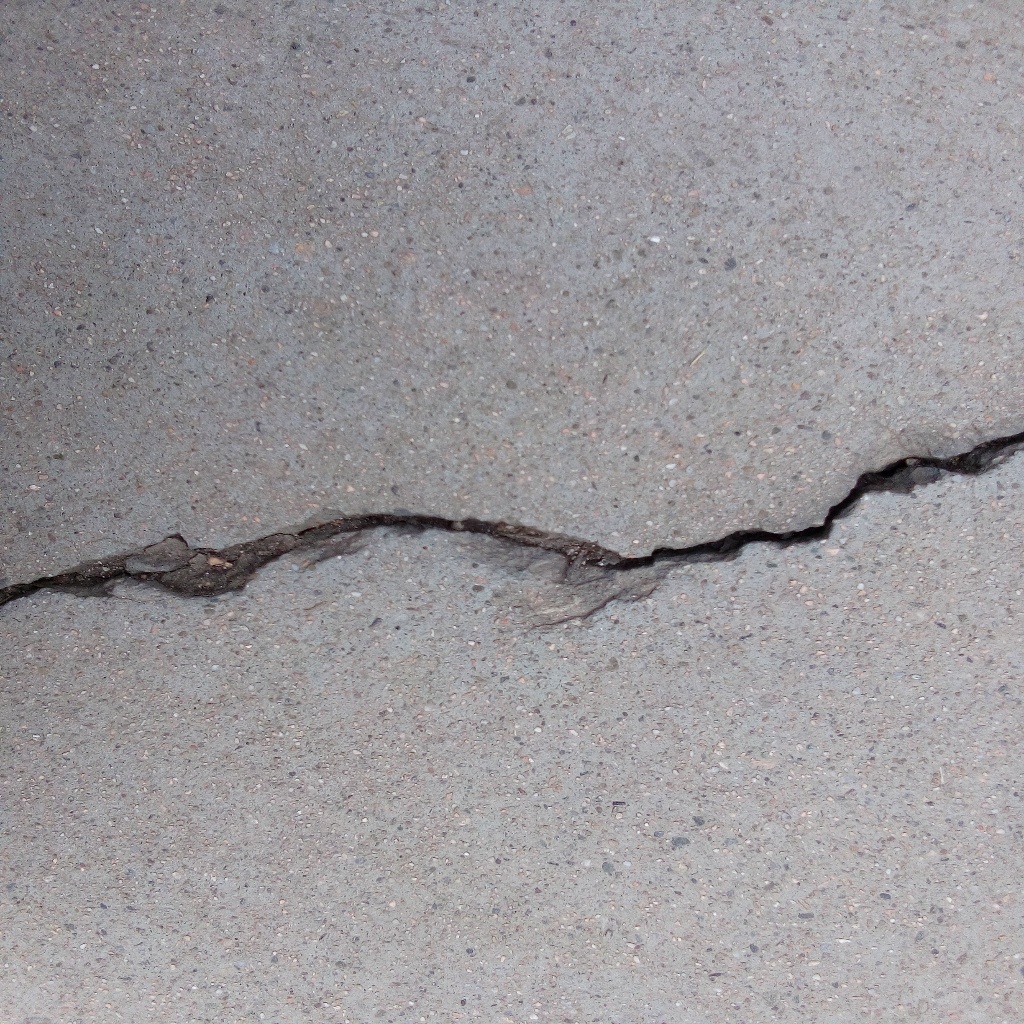

Supplement: S2 File — (ZIP) [file pone.0330218.s002.zip › 1 (323).jpg]

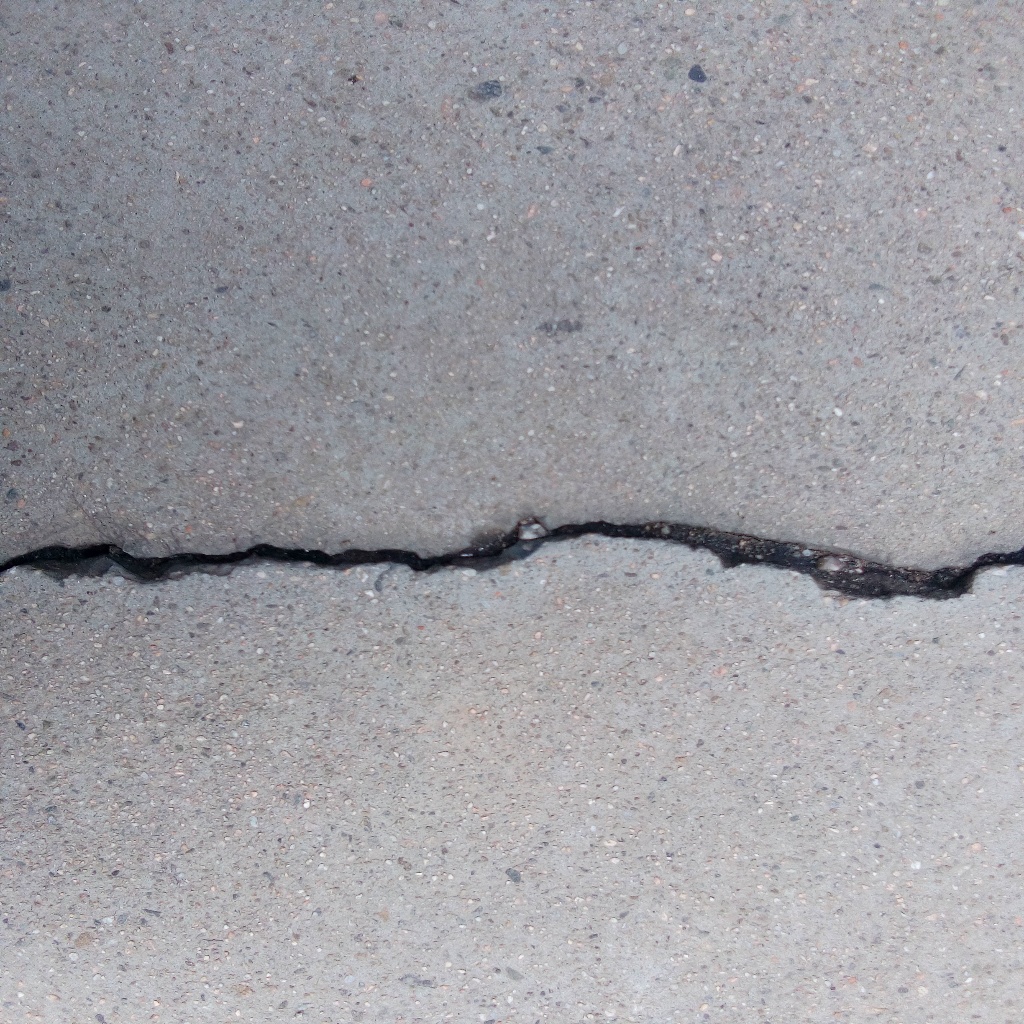

Supplement: S2 File — (ZIP) [file pone.0330218.s002.zip › 1 (324).jpg]

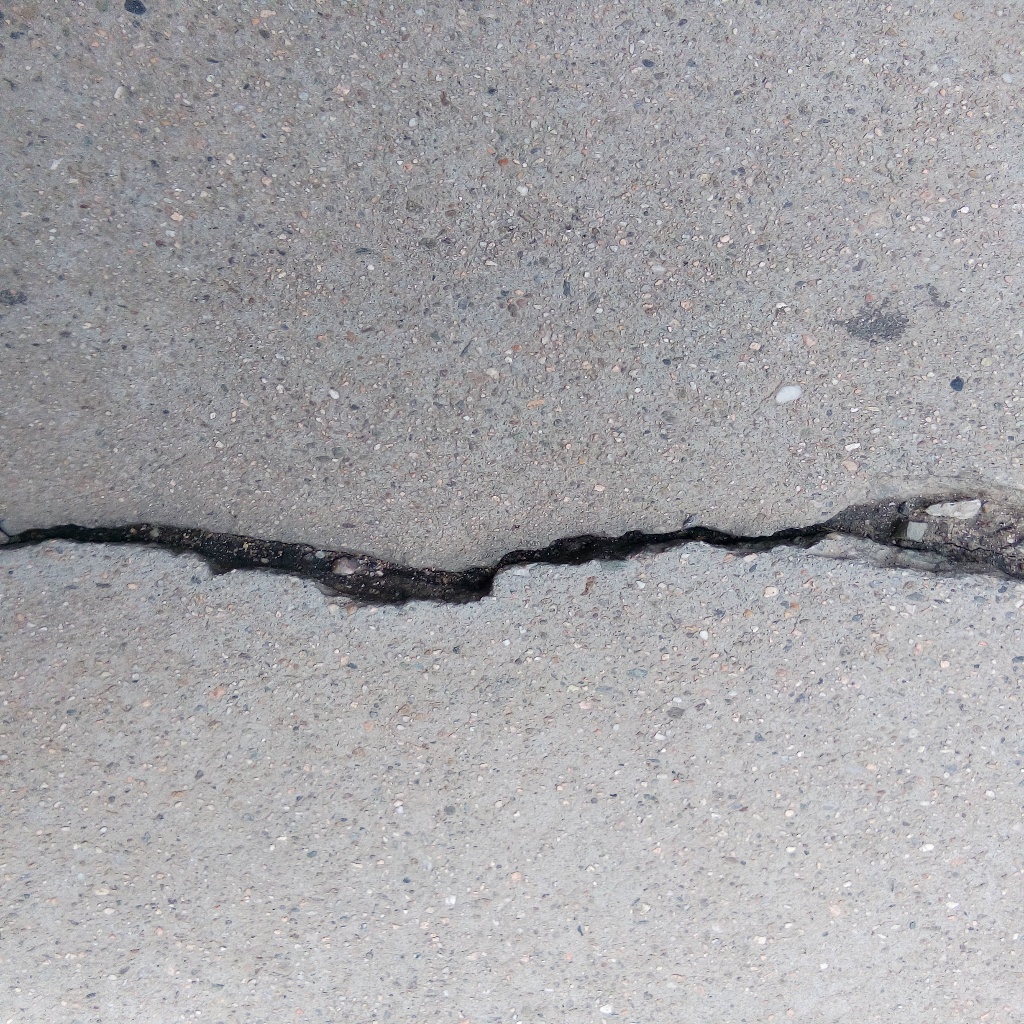

Supplement: S2 File — (ZIP) [file pone.0330218.s002.zip › 1 (325).jpg]

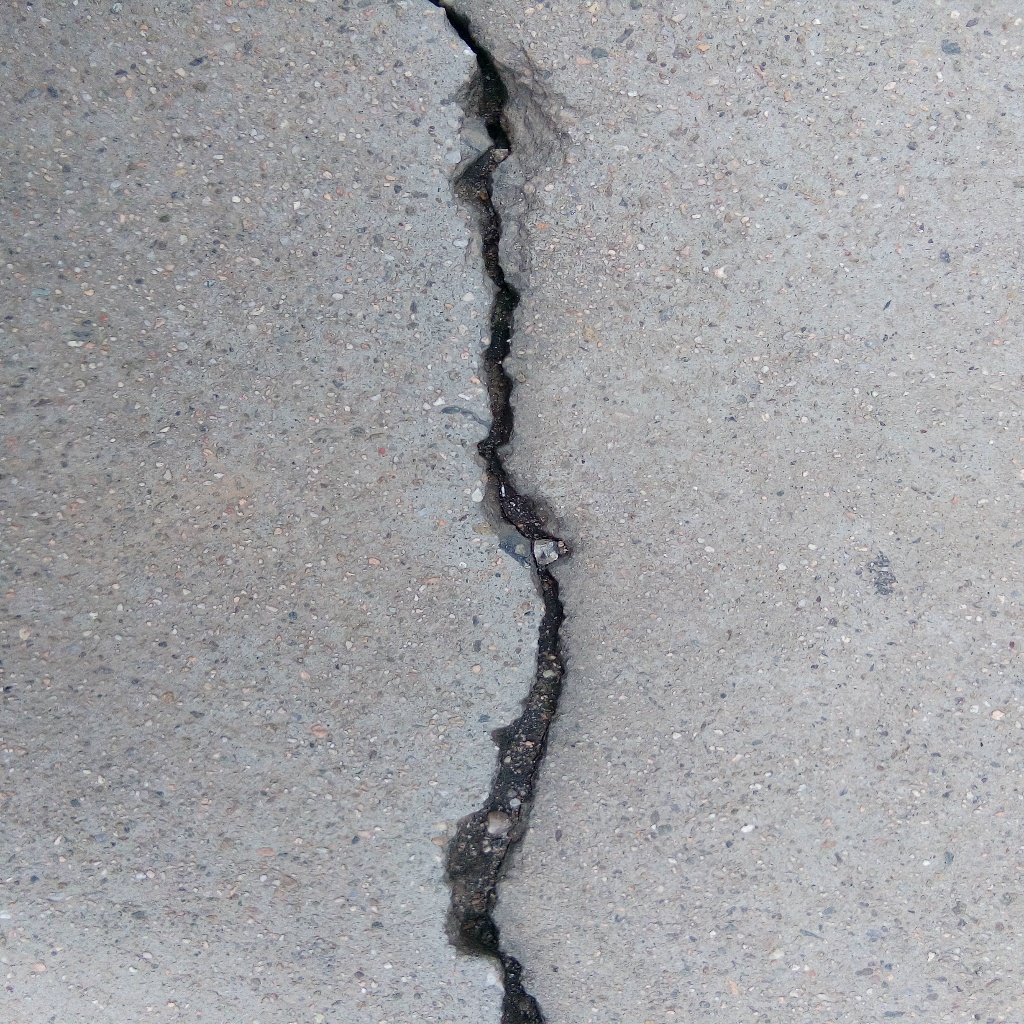

Supplement: S2 File — (ZIP) [file pone.0330218.s002.zip › 1 (326).jpg]

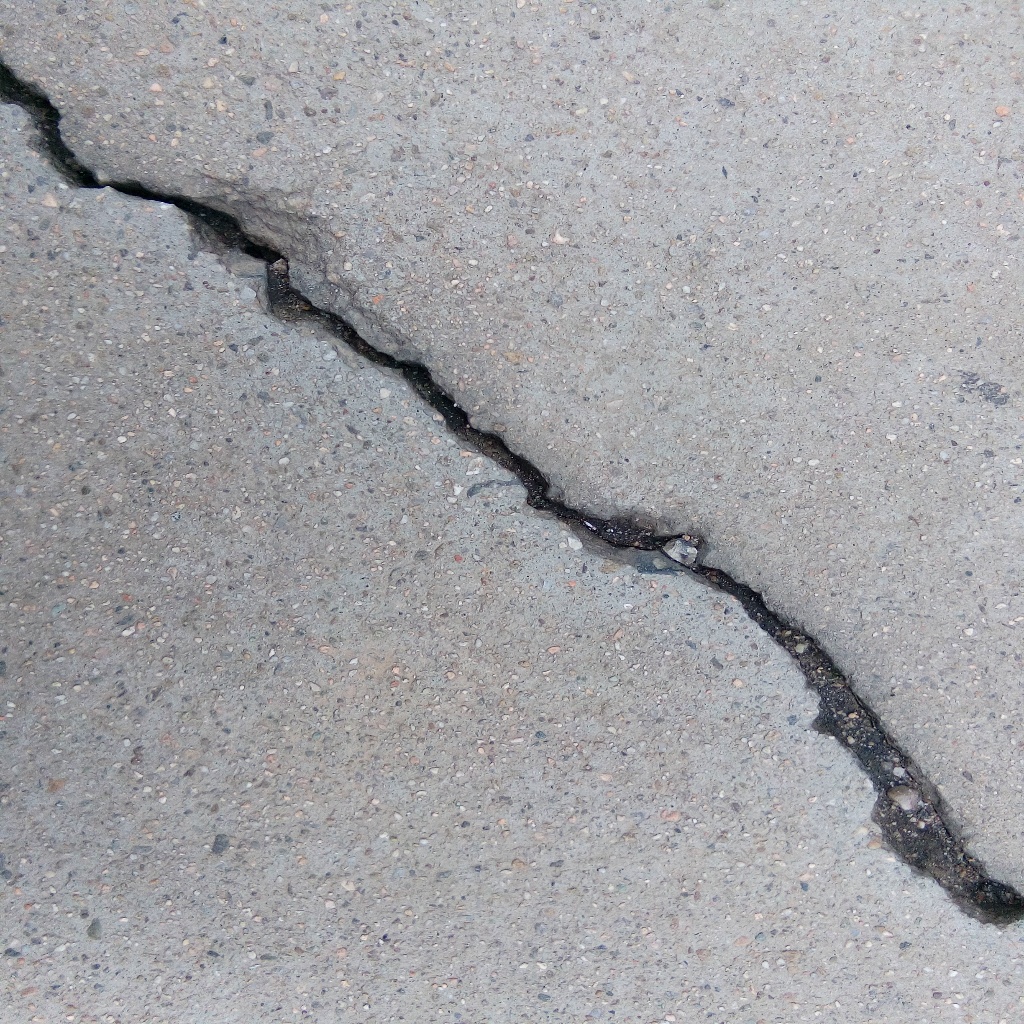

Supplement: S2 File — (ZIP) [file pone.0330218.s002.zip › 1 (327).jpg]

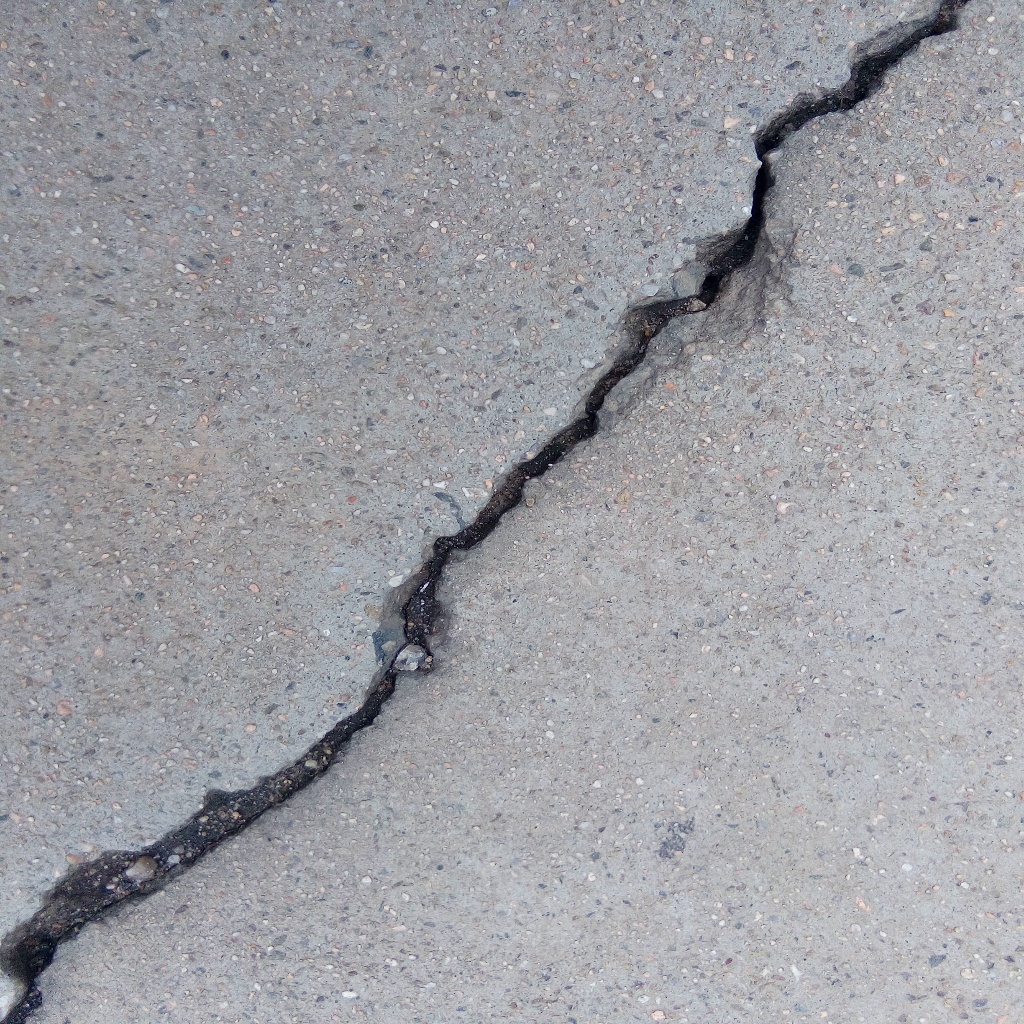

Supplement: S2 File — (ZIP) [file pone.0330218.s002.zip › 1 (328).jpg]

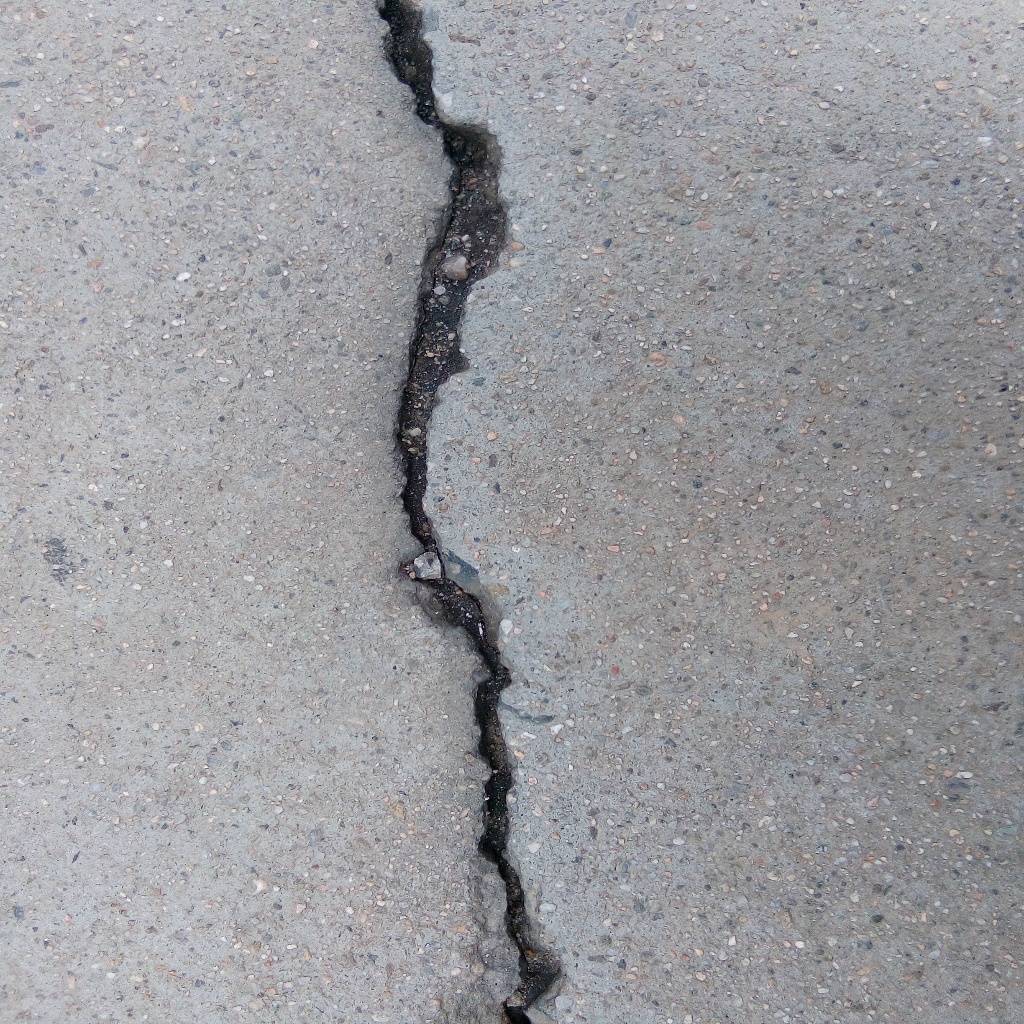

Supplement: S2 File — (ZIP) [file pone.0330218.s002.zip › 1 (329).jpg]

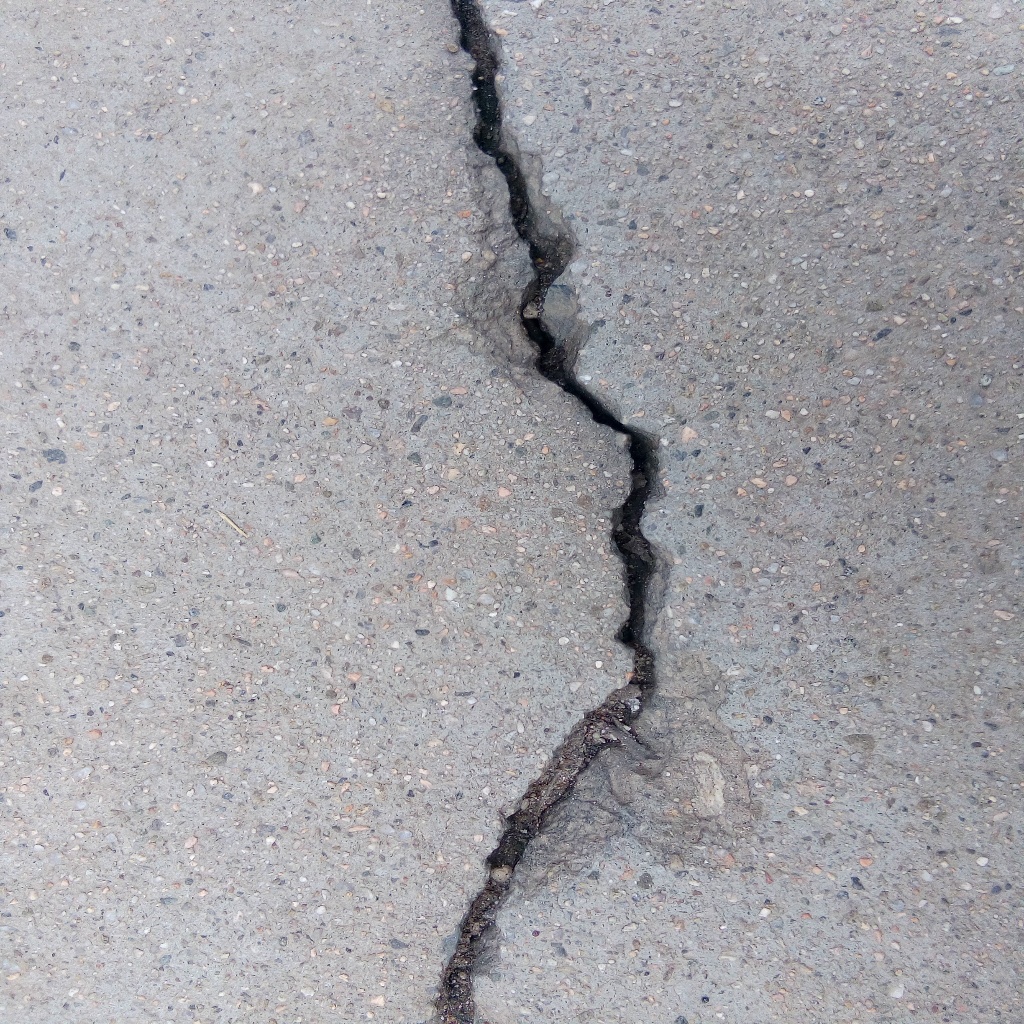

Supplement: S2 File — (ZIP) [file pone.0330218.s002.zip › 1 (330).jpg]

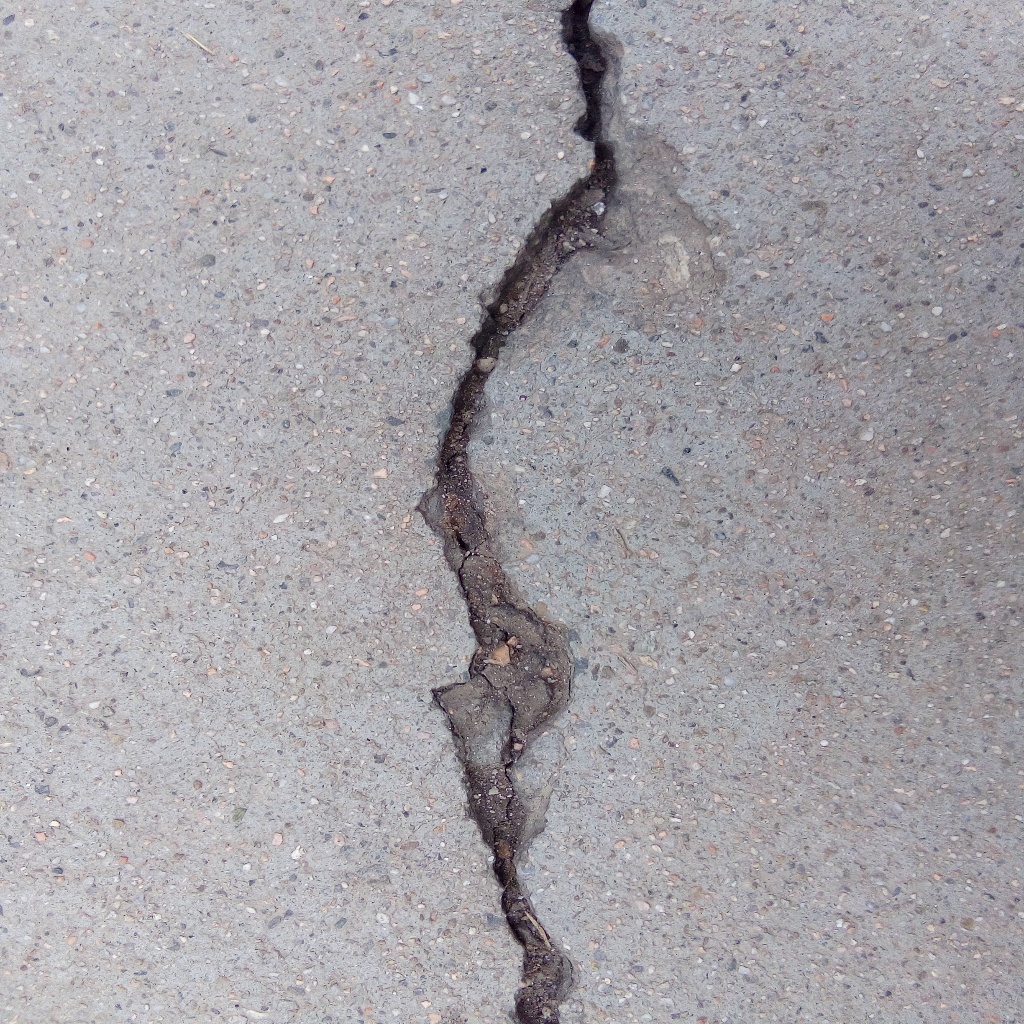

Supplement: S2 File — (ZIP) [file pone.0330218.s002.zip › 1 (331).jpg]

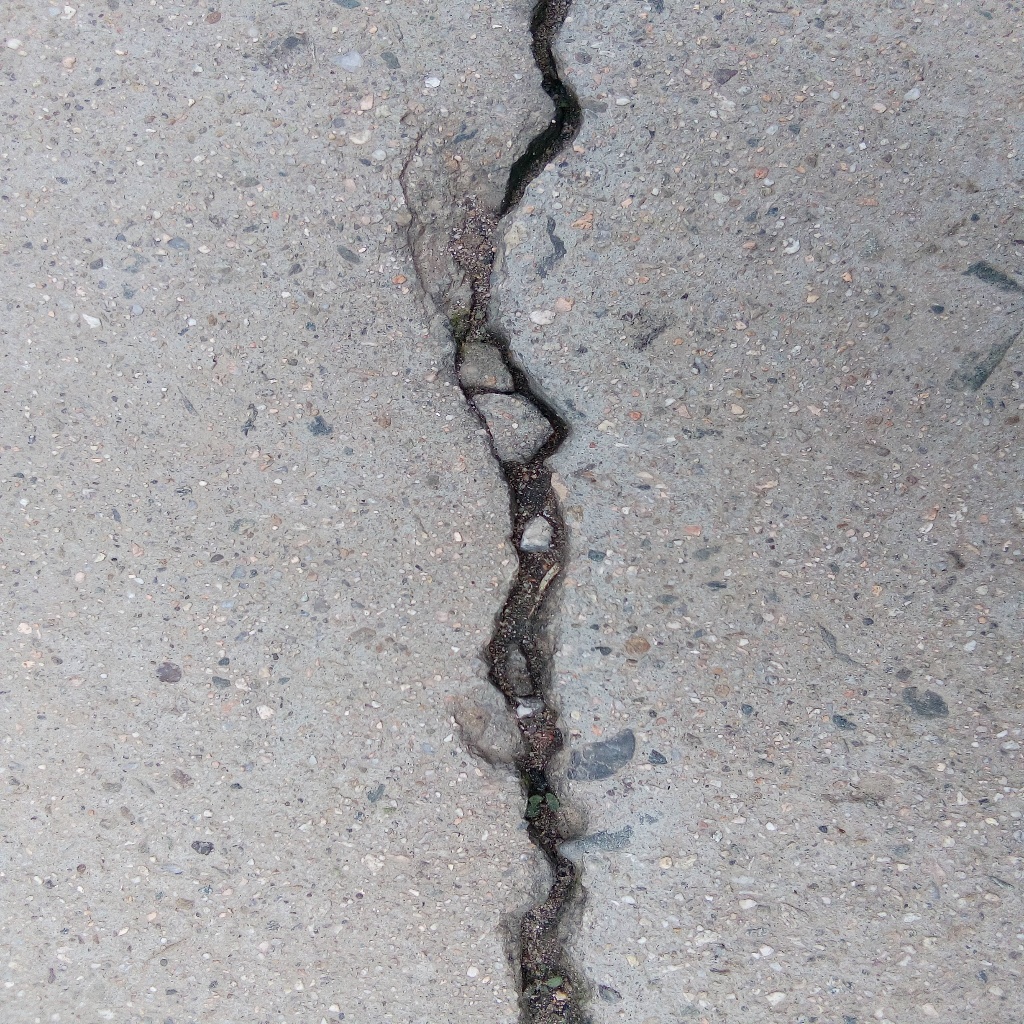

Supplement: S2 File — (ZIP) [file pone.0330218.s002.zip › 1 (332).jpg]

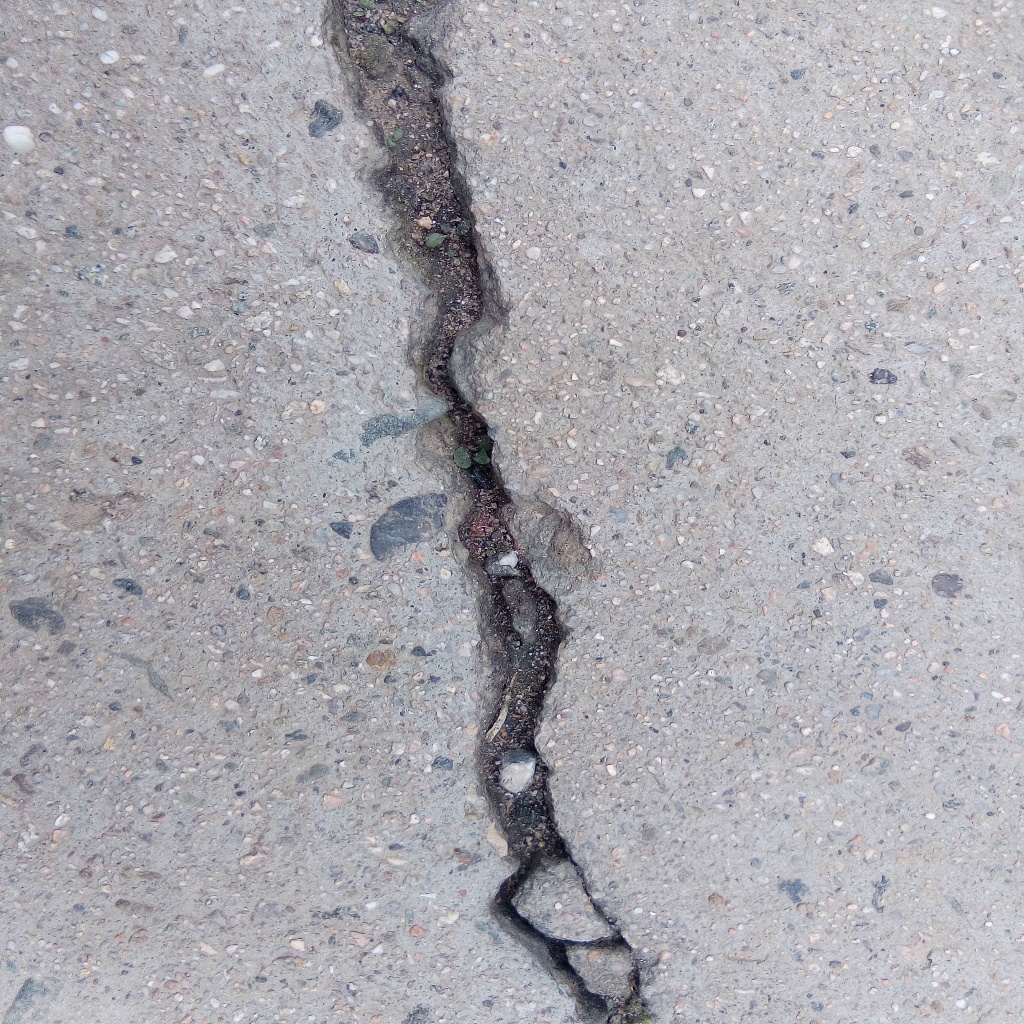

Supplement: S2 File — (ZIP) [file pone.0330218.s002.zip › 1 (333).jpg]

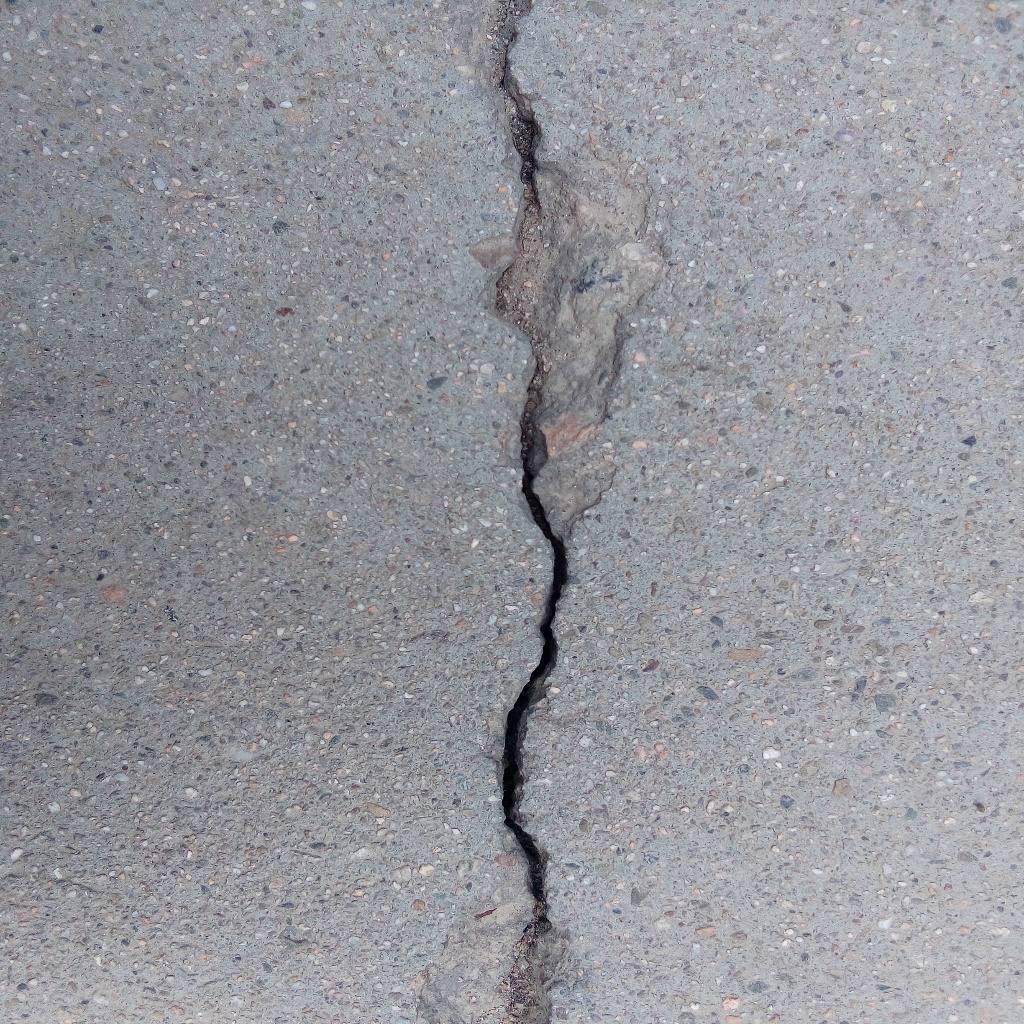

Supplement: S2 File — (ZIP) [file pone.0330218.s002.zip › 1 (334).jpg]

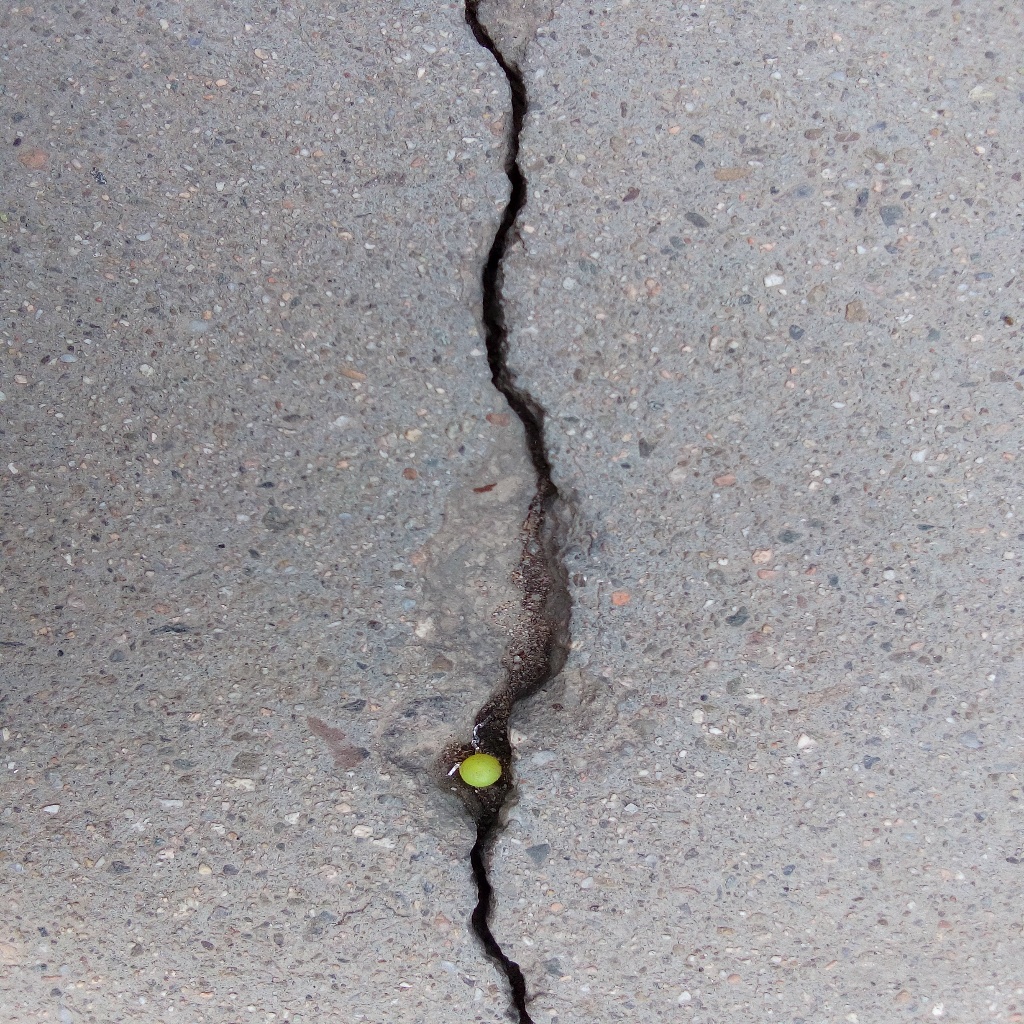

Supplement: S2 File — (ZIP) [file pone.0330218.s002.zip › 1 (335).jpg]

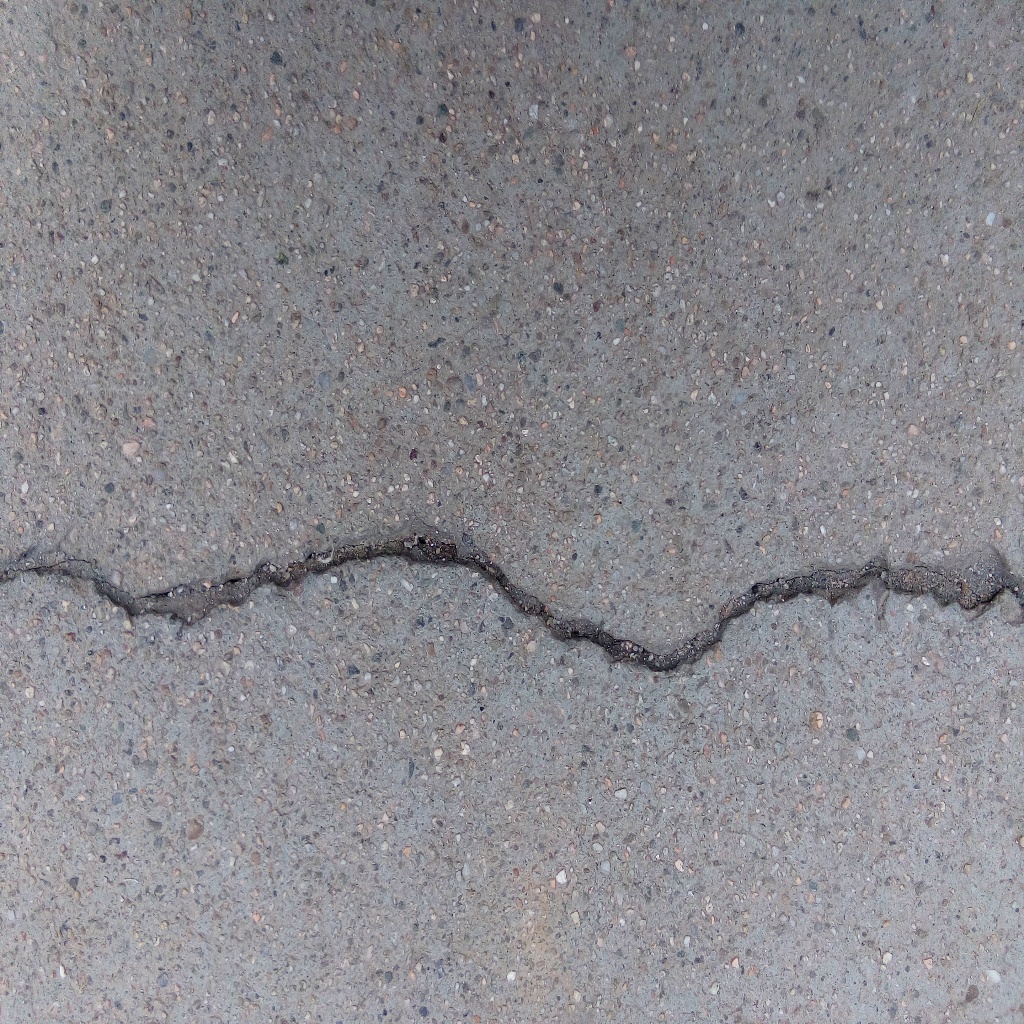

Supplement: S2 File — (ZIP) [file pone.0330218.s002.zip › 1 (336).jpg]

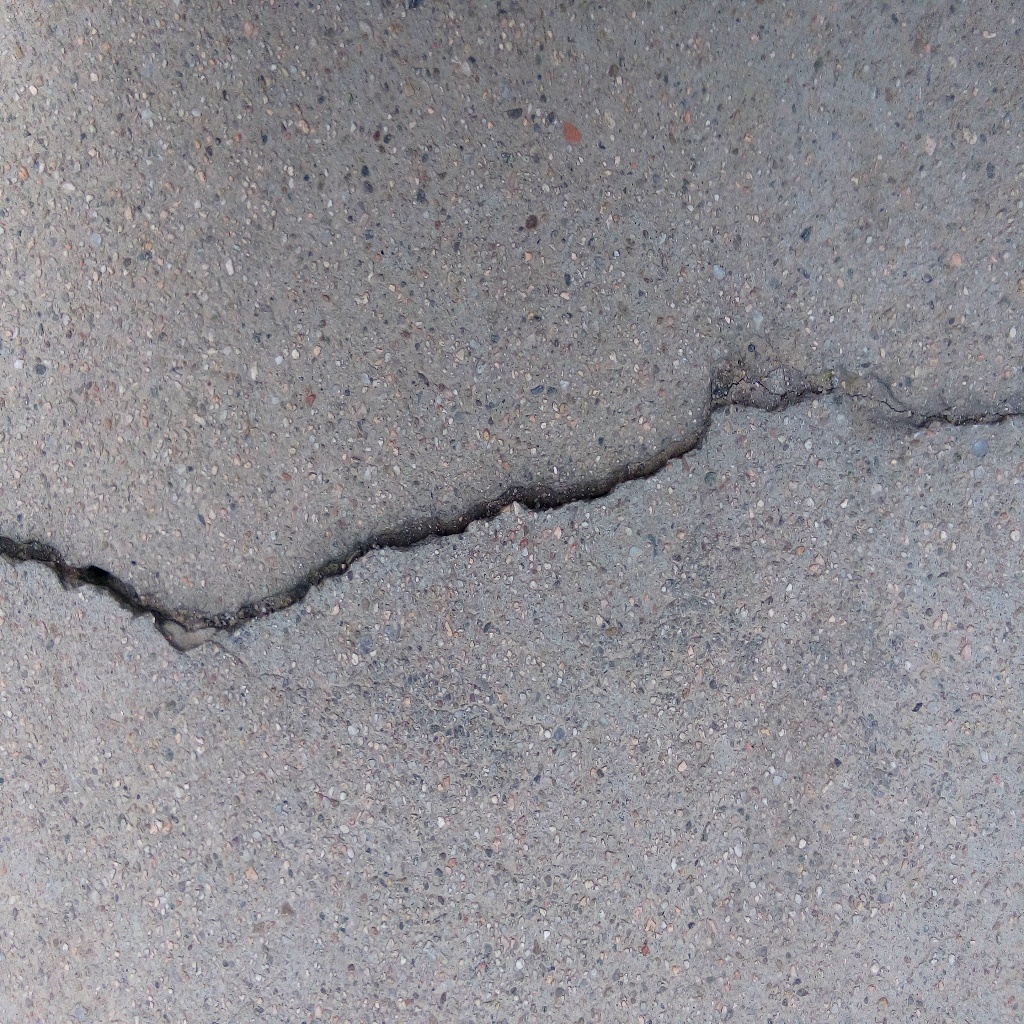

Supplement: S2 File — (ZIP) [file pone.0330218.s002.zip › 1 (337).jpg]

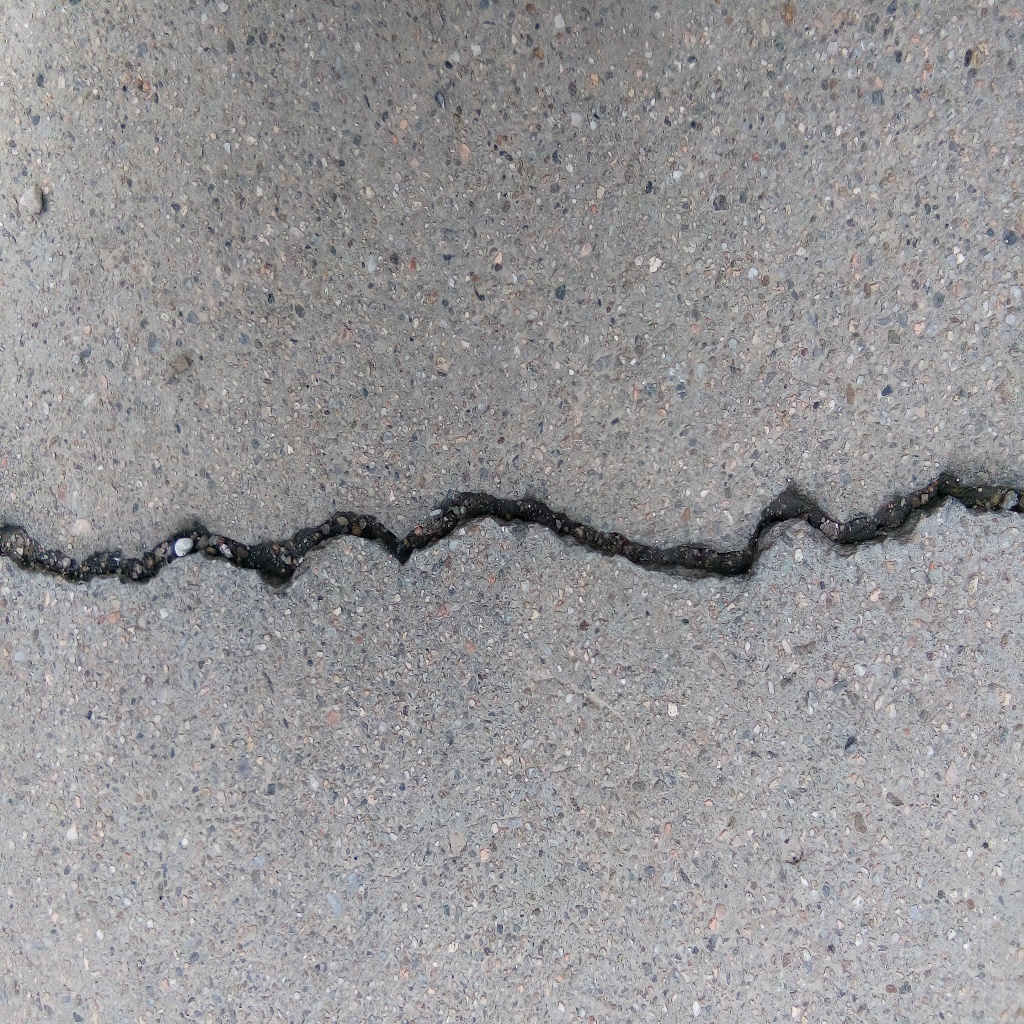

Supplement: S2 File — (ZIP) [file pone.0330218.s002.zip › 1 (338).jpg]

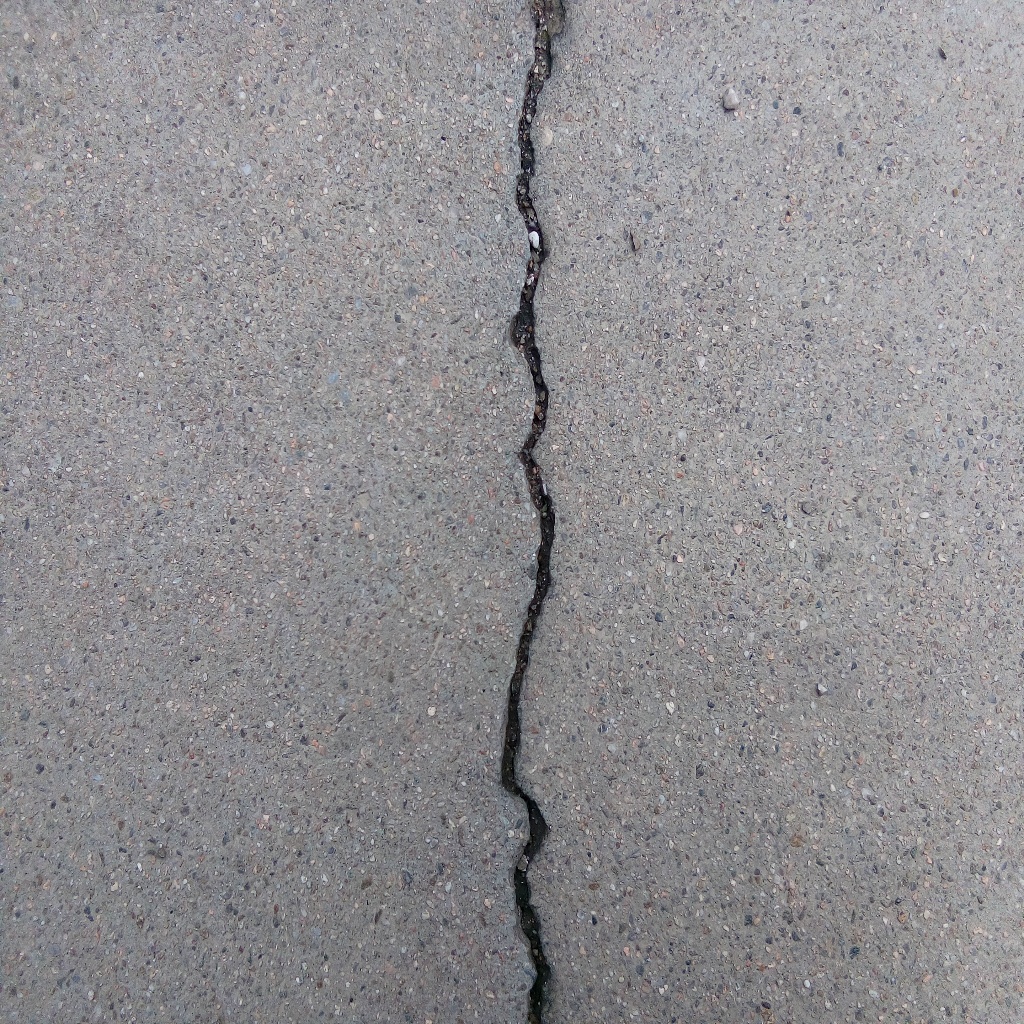

Supplement: S2 File — (ZIP) [file pone.0330218.s002.zip › 1 (339).jpg]

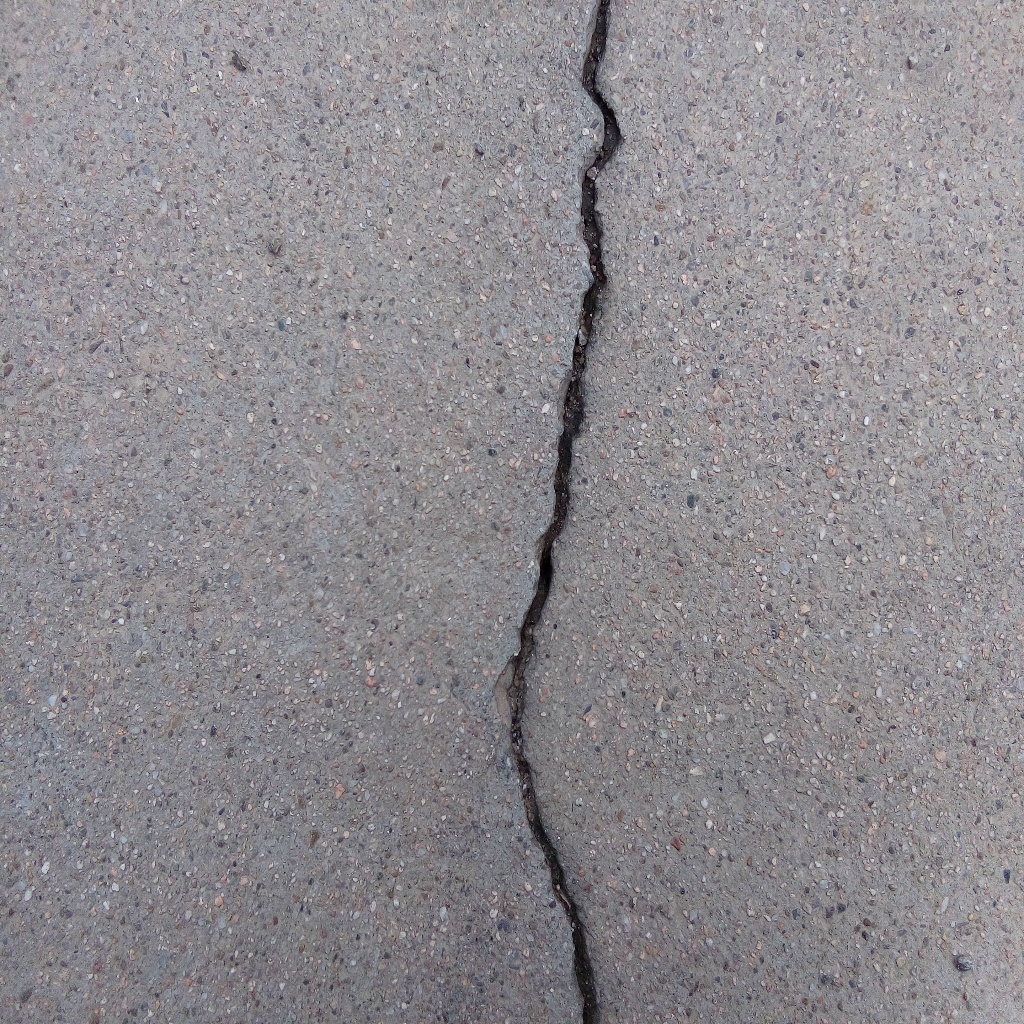

Supplement: S2 File — (ZIP) [file pone.0330218.s002.zip › 1 (340).jpg]

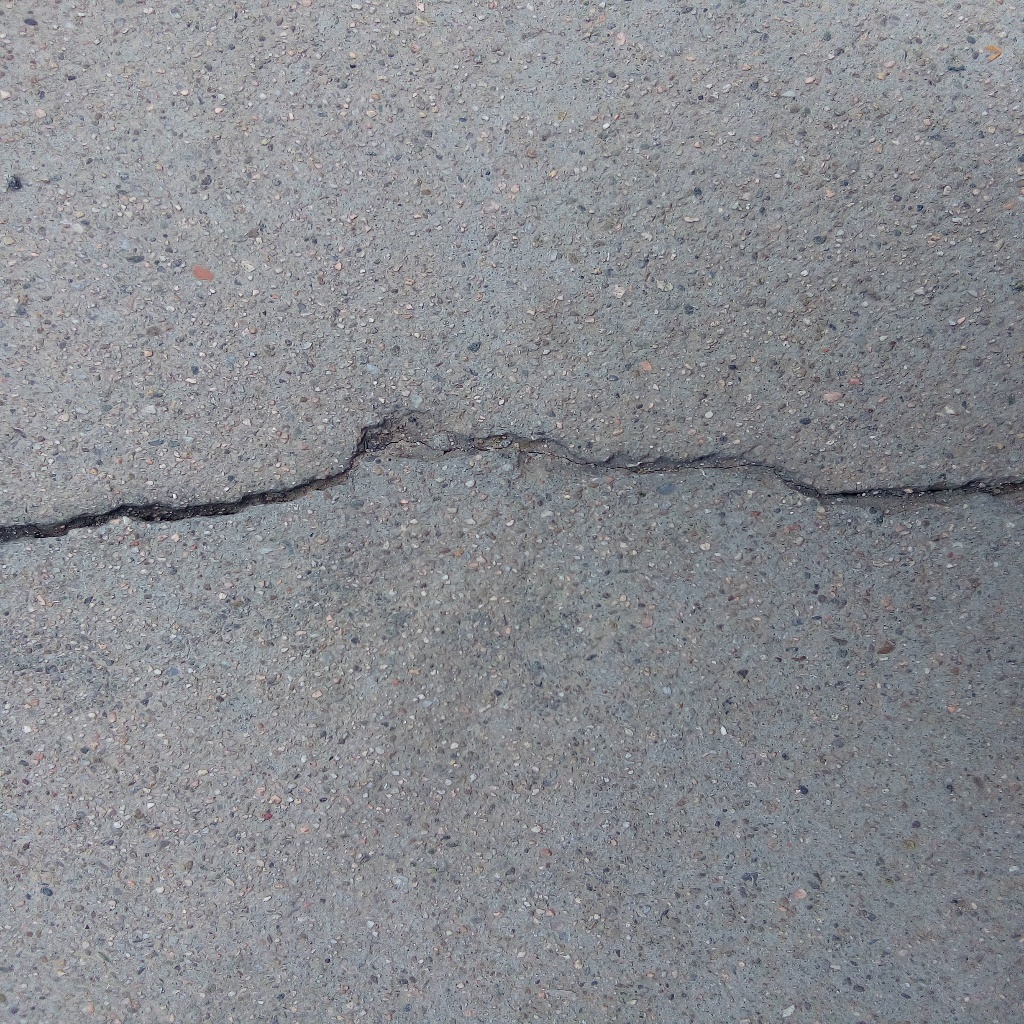

Supplement: S2 File — (ZIP) [file pone.0330218.s002.zip › 1 (341).jpg]

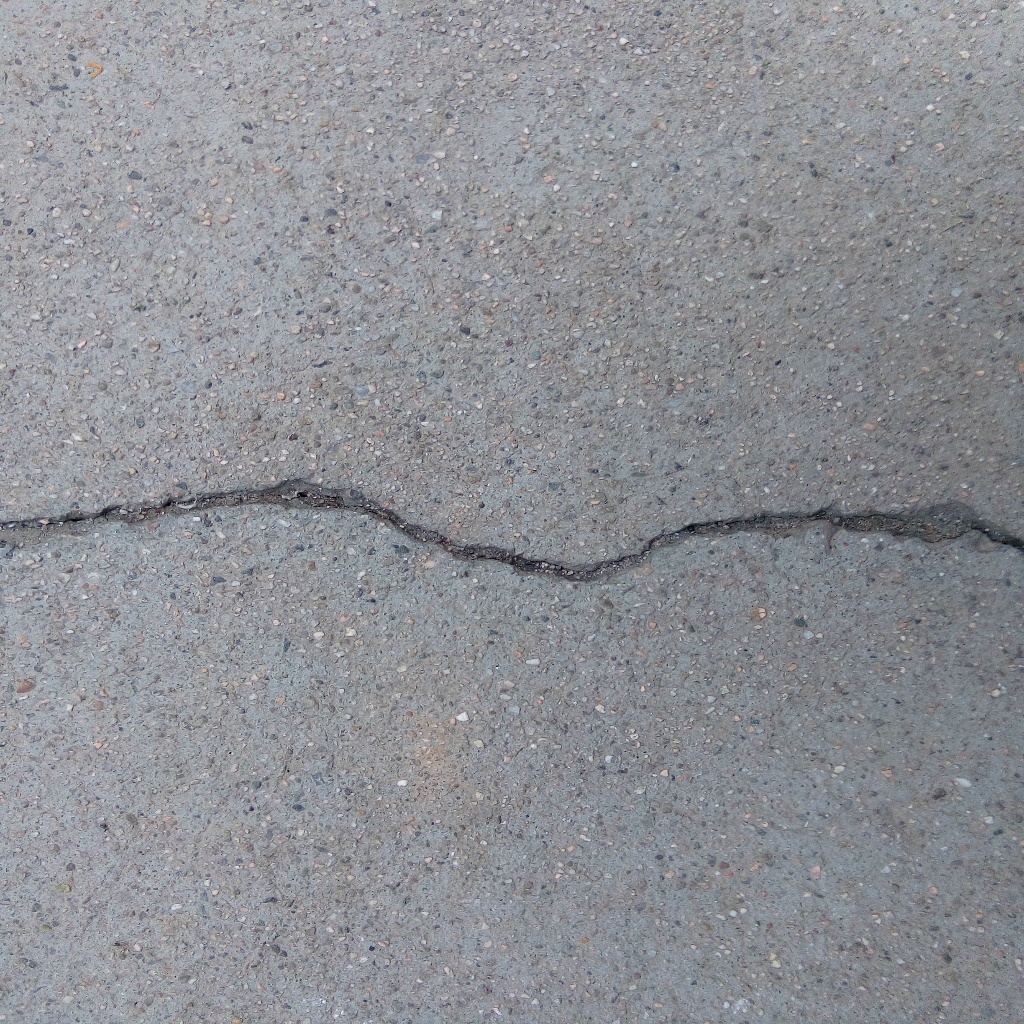

Supplement: S2 File — (ZIP) [file pone.0330218.s002.zip › 1 (342).jpg]

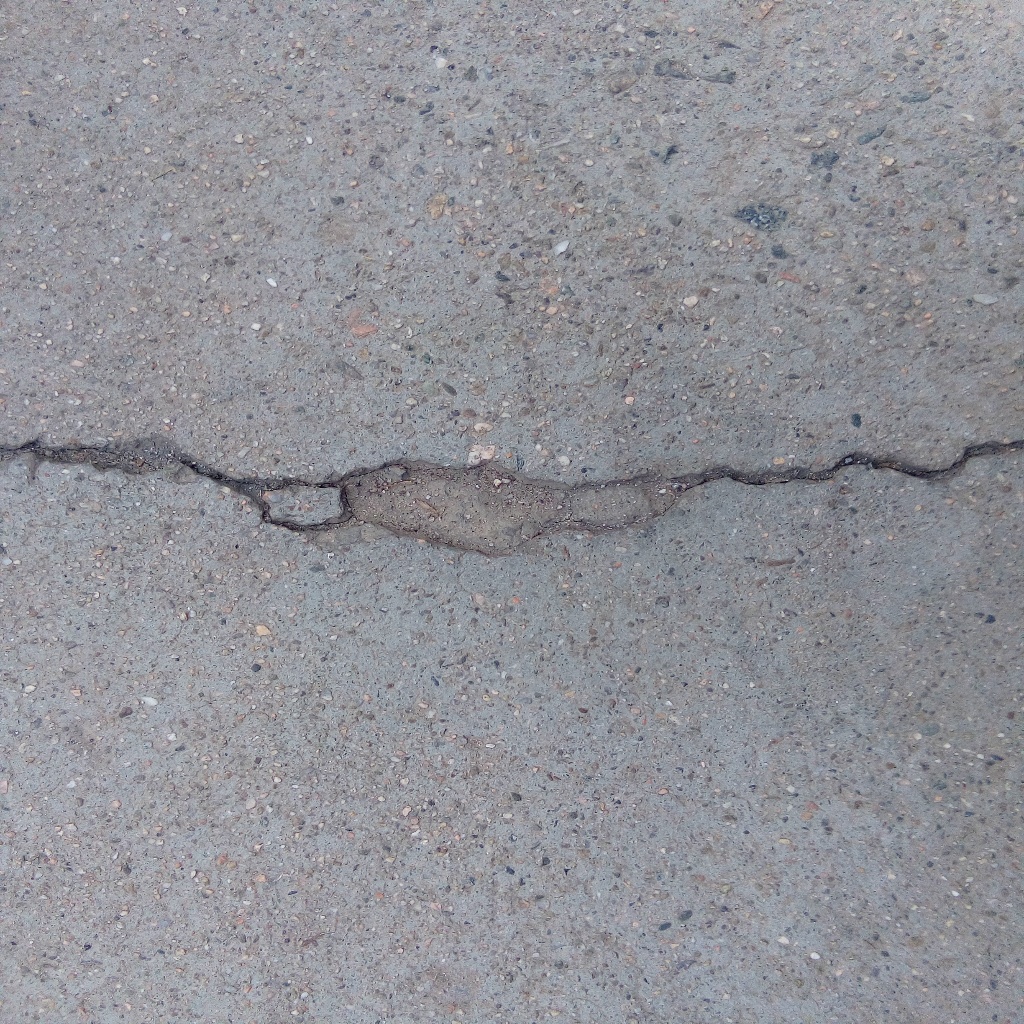

Supplement: S2 File — (ZIP) [file pone.0330218.s002.zip › 1 (343).jpg]

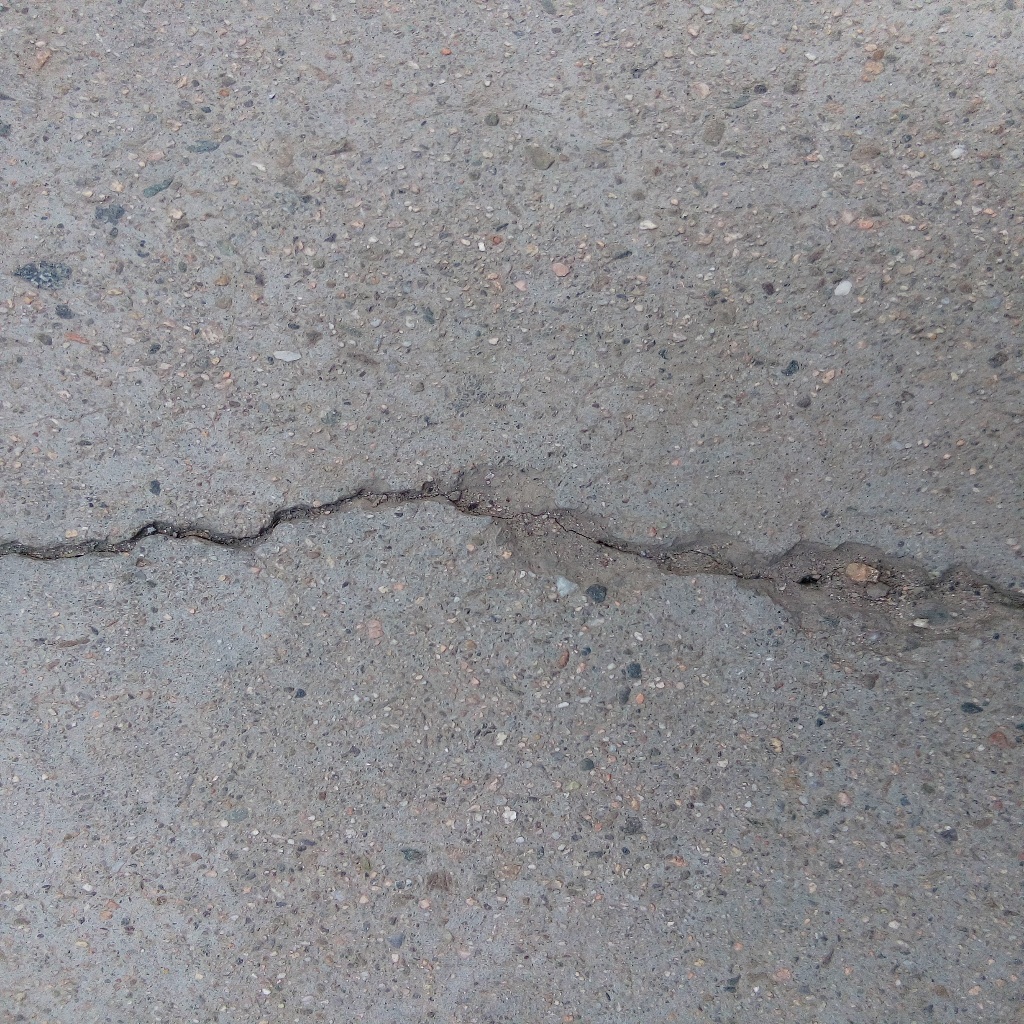

Supplement: S2 File — (ZIP) [file pone.0330218.s002.zip › 1 (344).jpg]

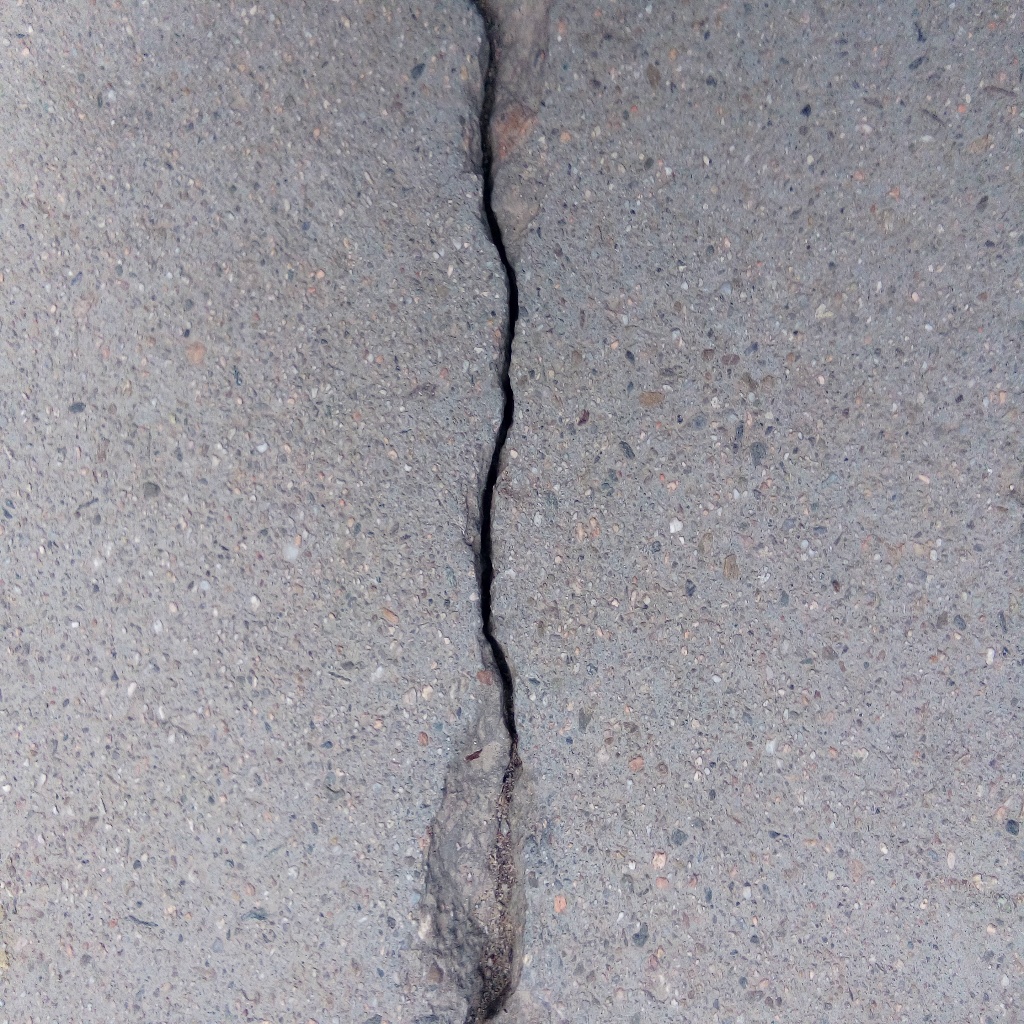

Supplement: S2 File — (ZIP) [file pone.0330218.s002.zip › 1 (346).jpg]

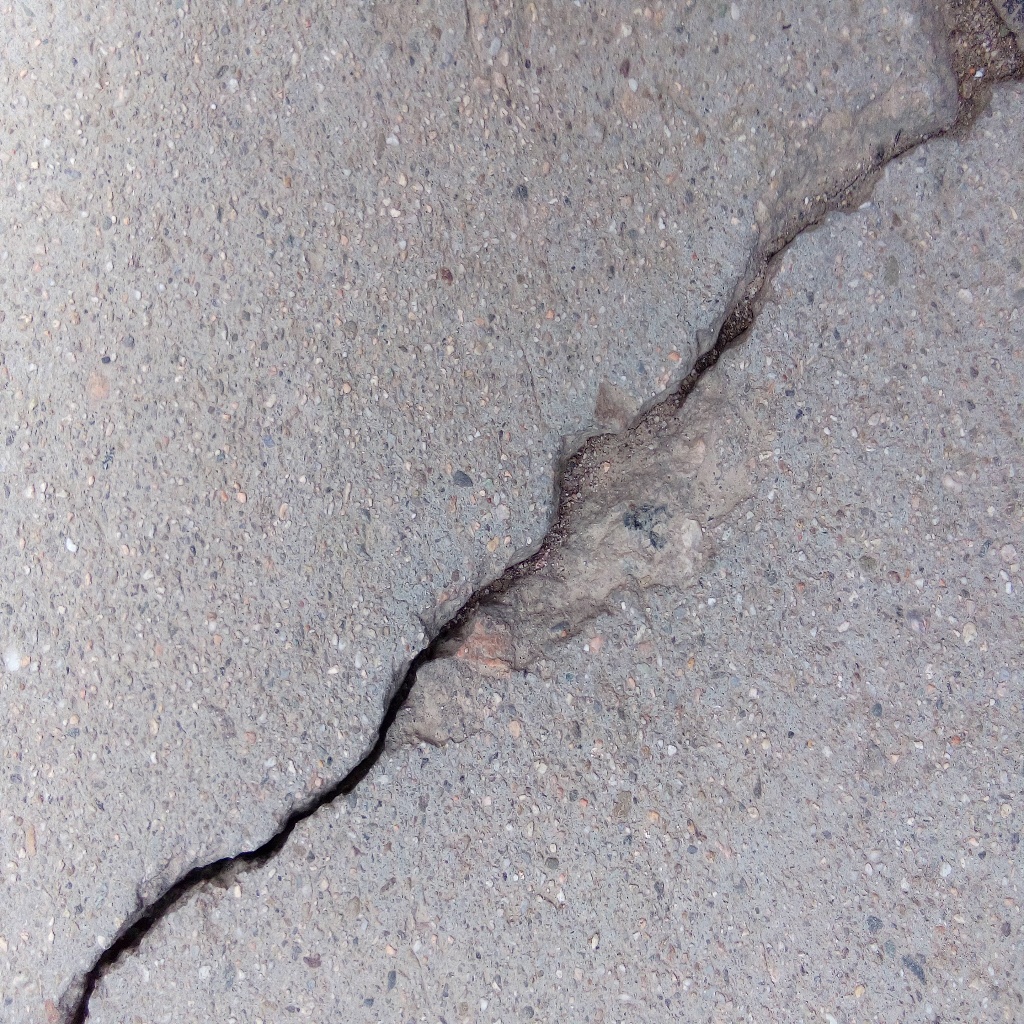

Supplement: S2 File — (ZIP) [file pone.0330218.s002.zip › 1 (347).jpg]

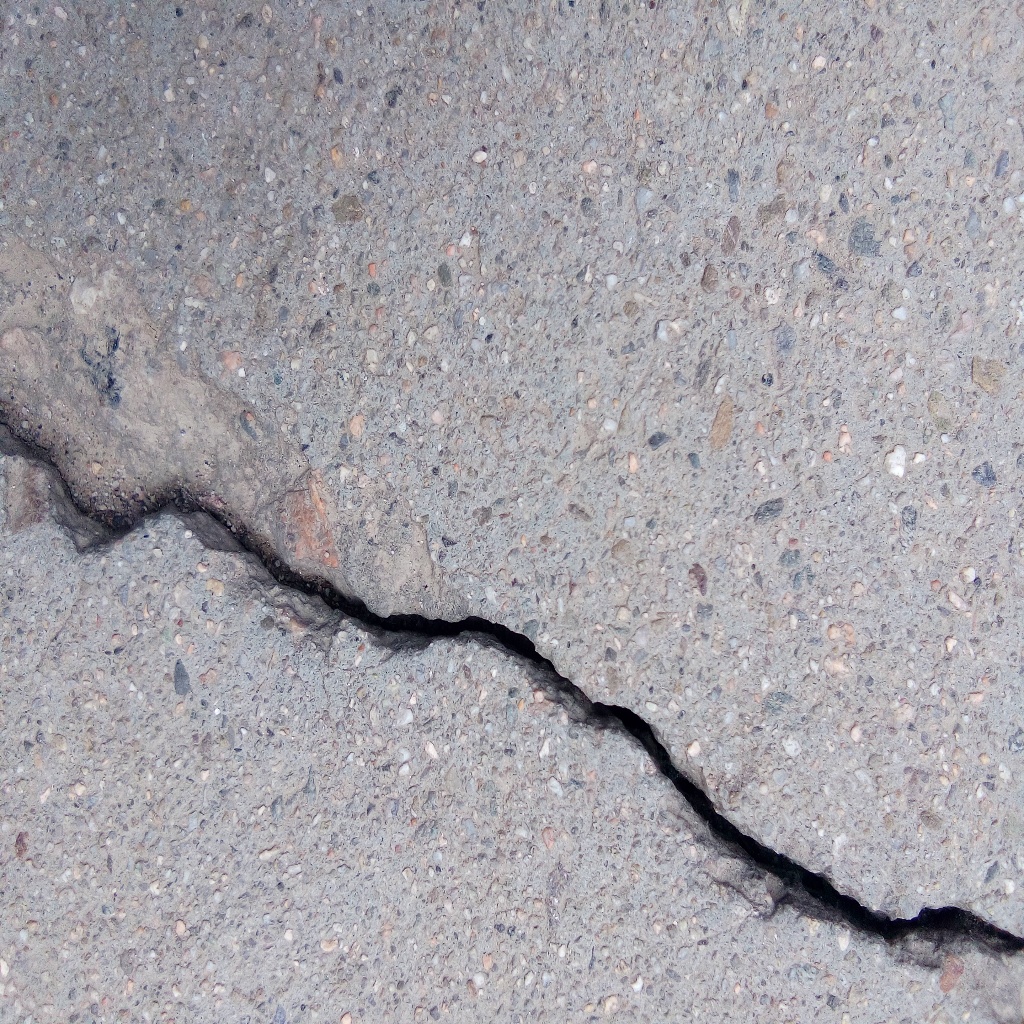

Supplement: S2 File — (ZIP) [file pone.0330218.s002.zip › 1 (348).jpg]

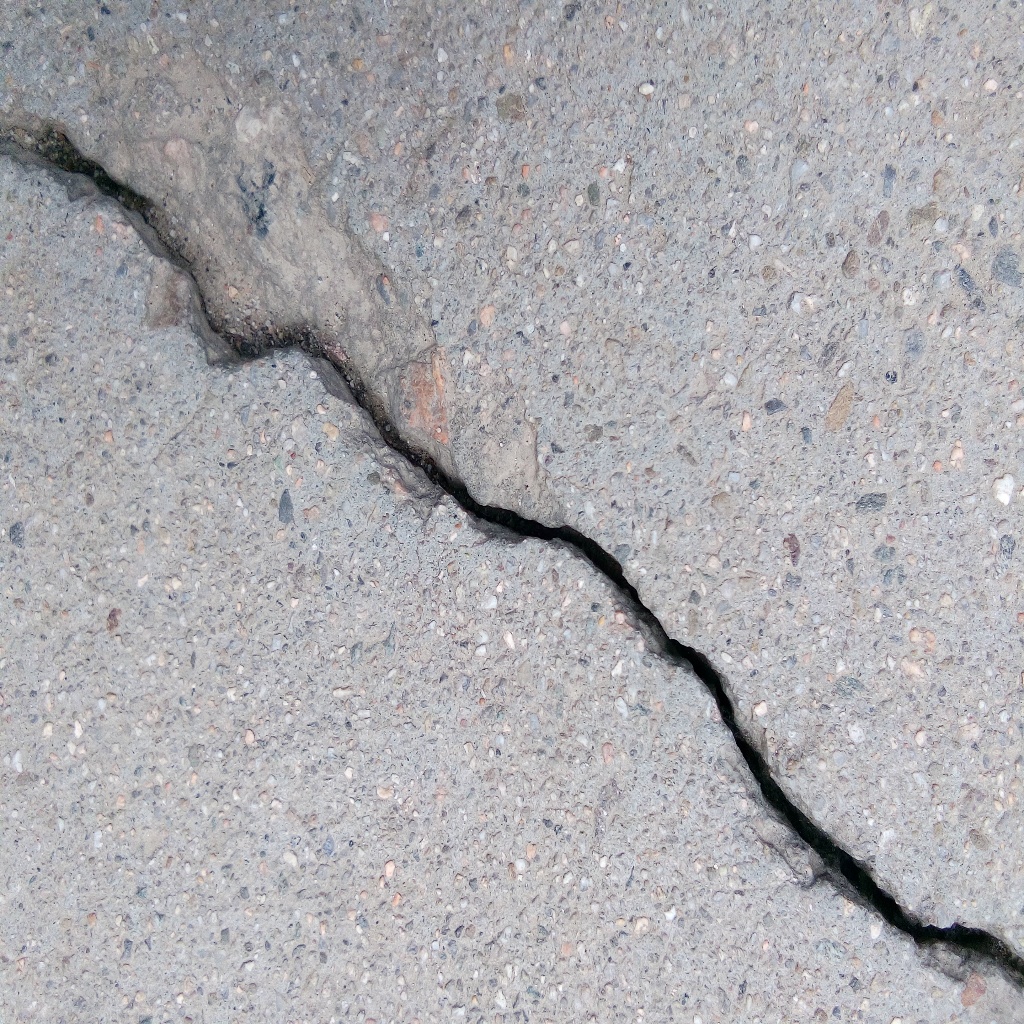

Supplement: S2 File — (ZIP) [file pone.0330218.s002.zip › 1 (349).jpg]

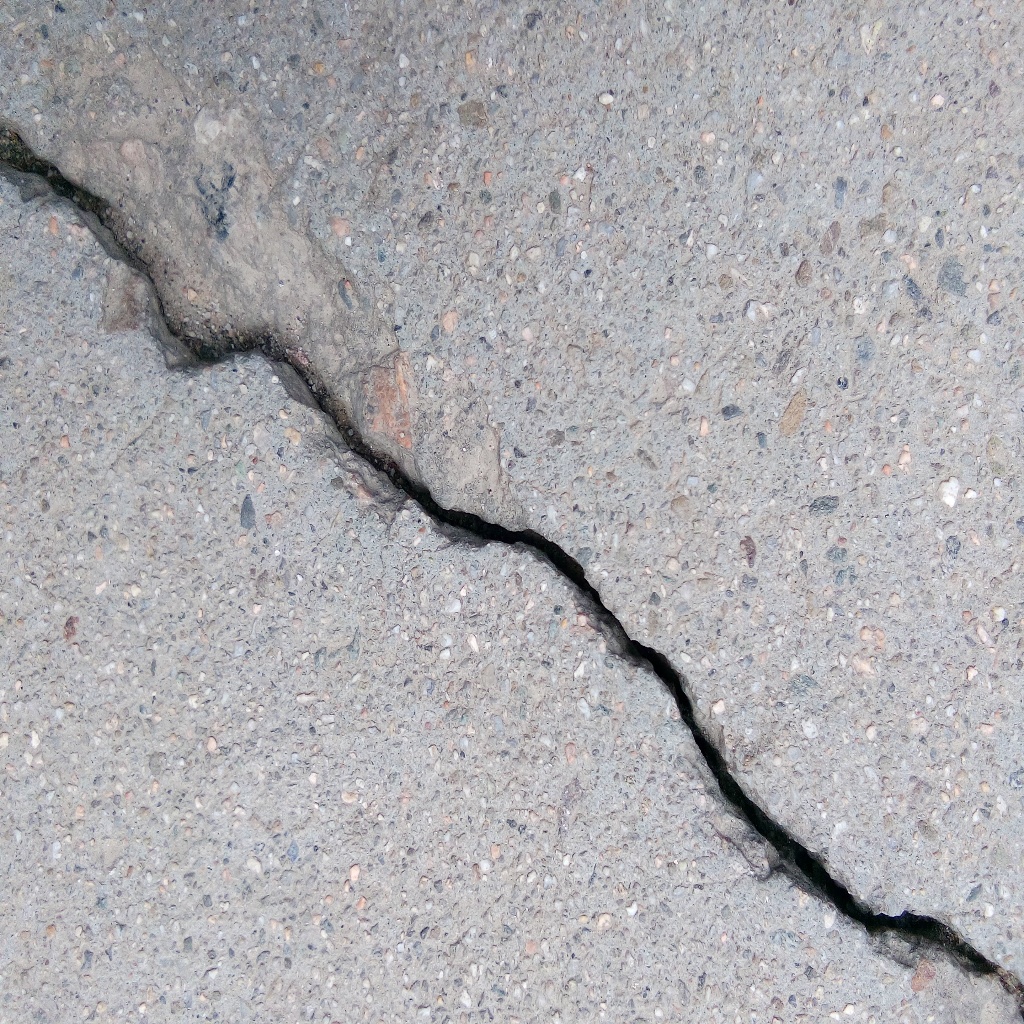

Supplement: S2 File — (ZIP) [file pone.0330218.s002.zip › 1 (350).jpg]

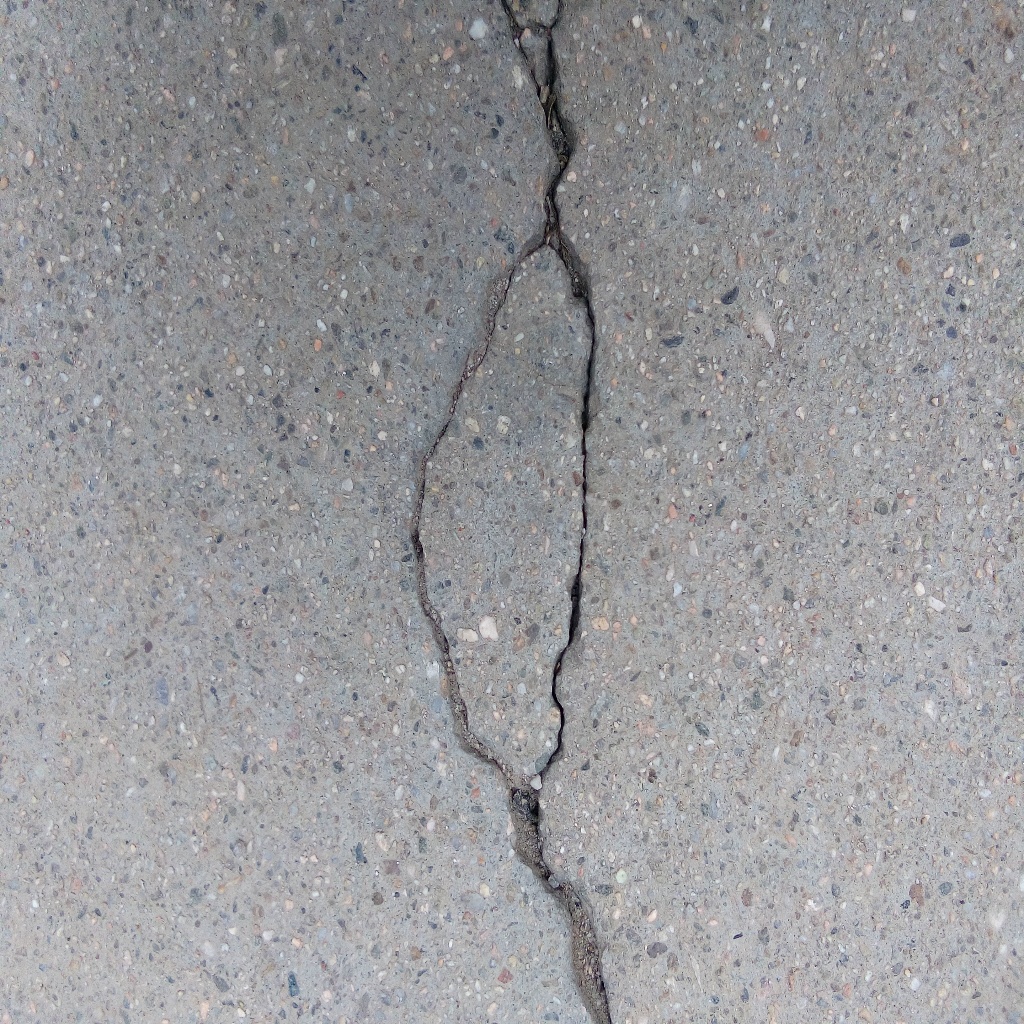

Supplement: S2 File — (ZIP) [file pone.0330218.s002.zip › 1 (355).jpg]

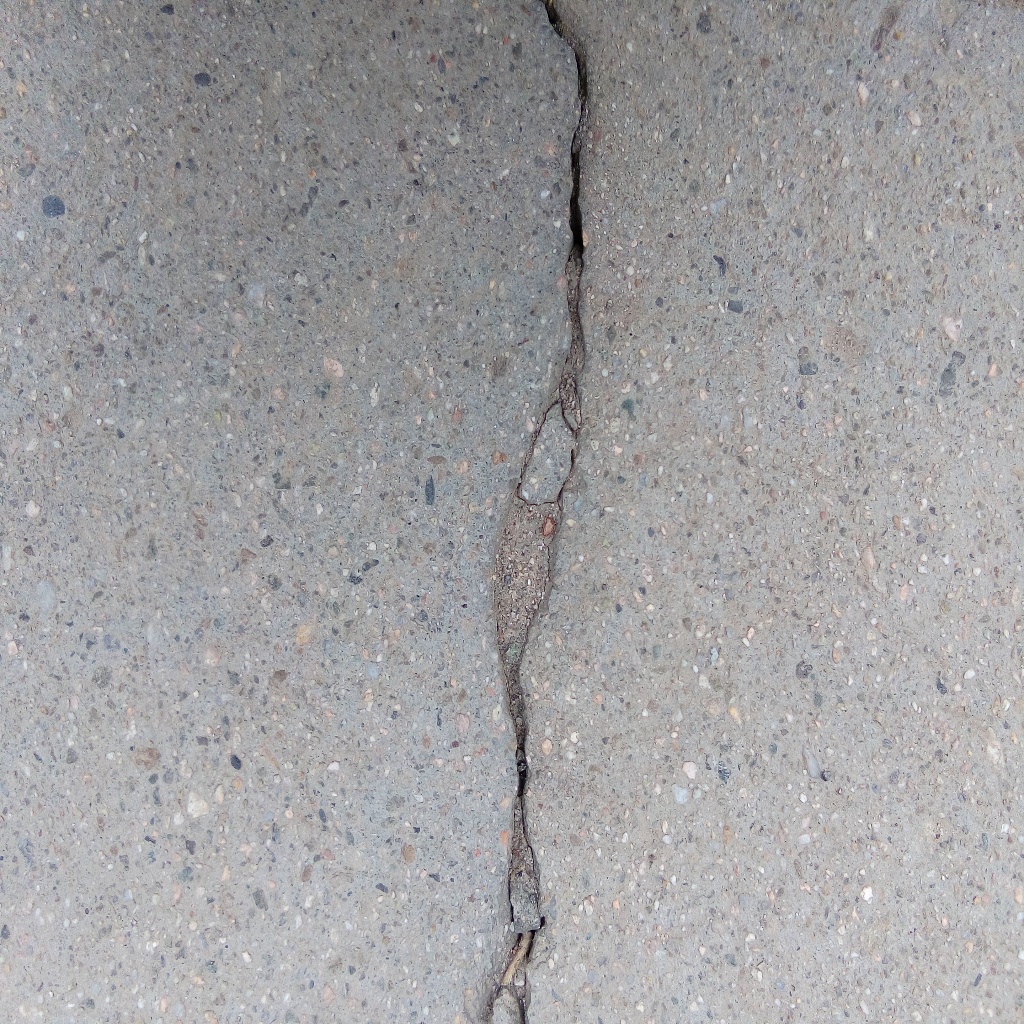

Supplement: S2 File — (ZIP) [file pone.0330218.s002.zip › 1 (356).jpg]

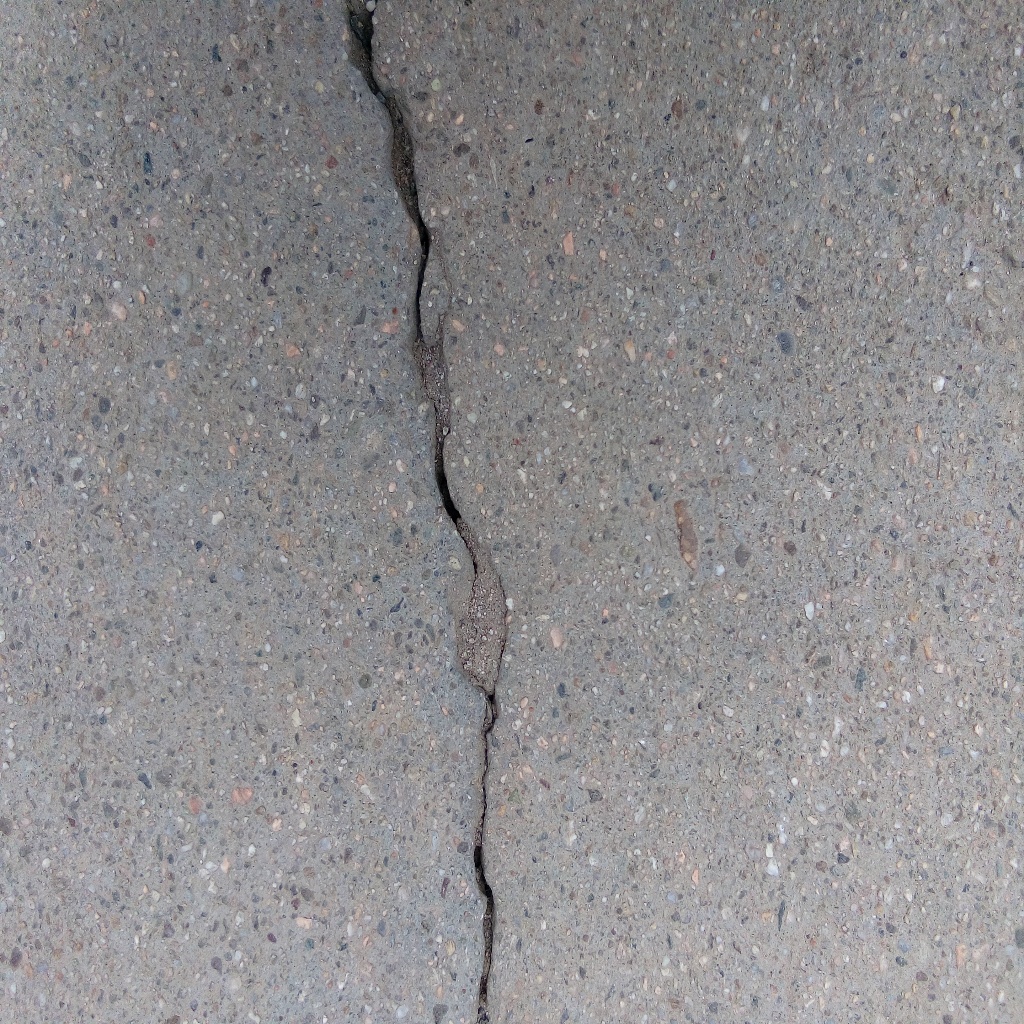

Supplement: S2 File — (ZIP) [file pone.0330218.s002.zip › 1 (357).jpg]

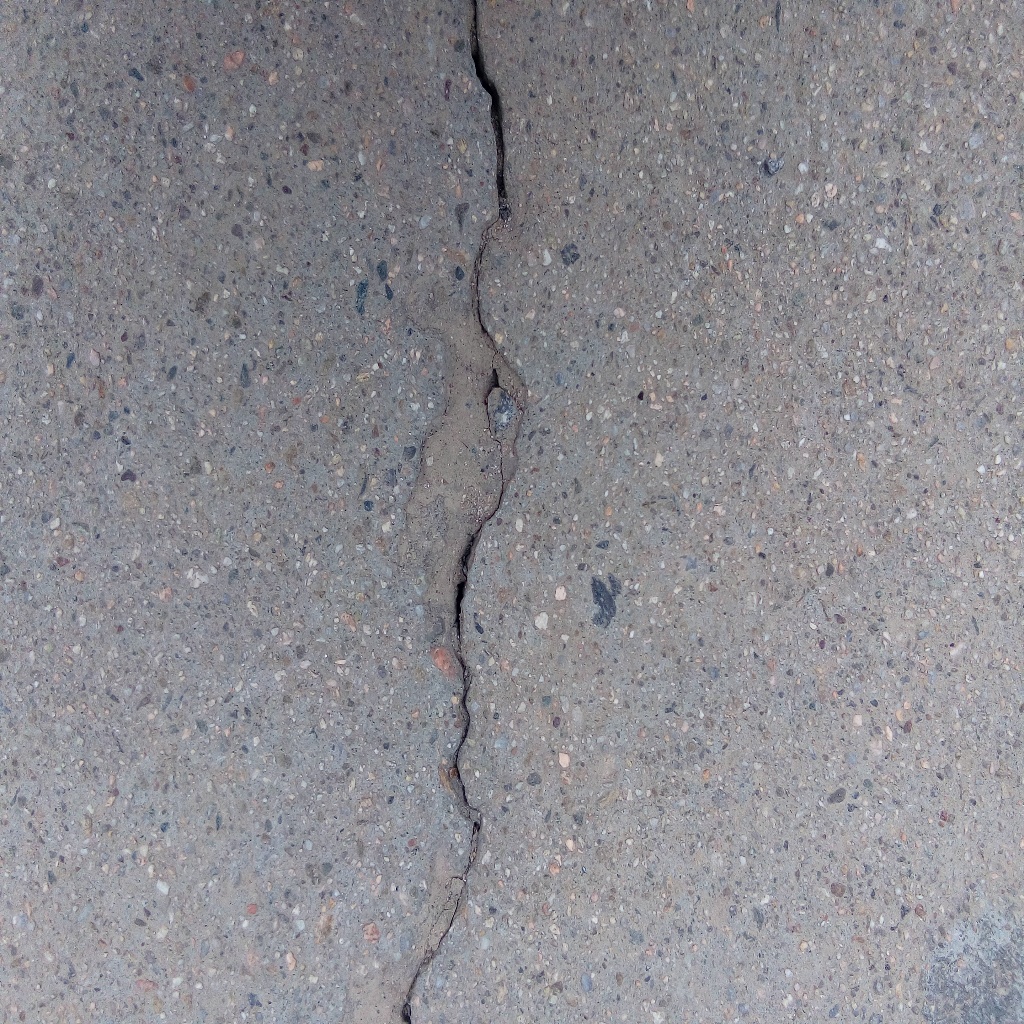

Supplement: S2 File — (ZIP) [file pone.0330218.s002.zip › 1 (358).jpg]

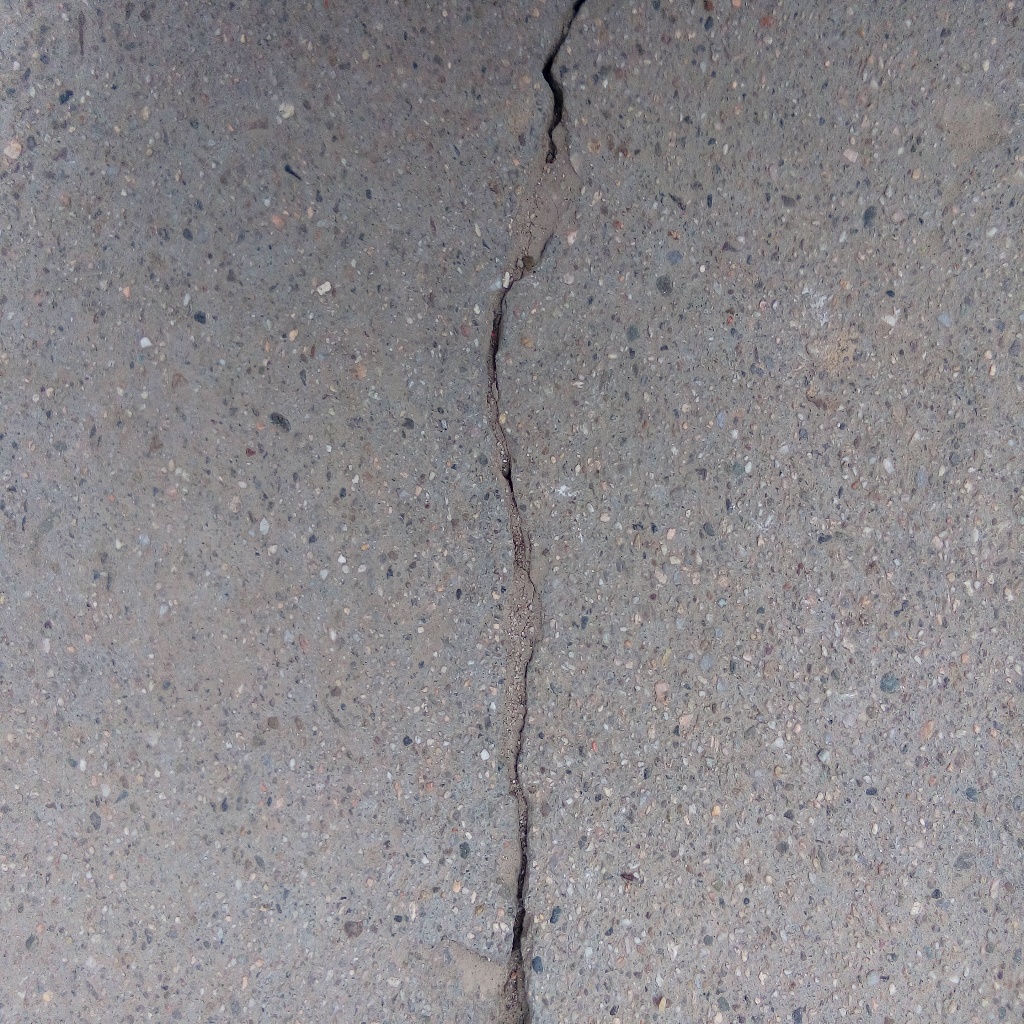

Supplement: S2 File — (ZIP) [file pone.0330218.s002.zip › 1 (359).jpg]

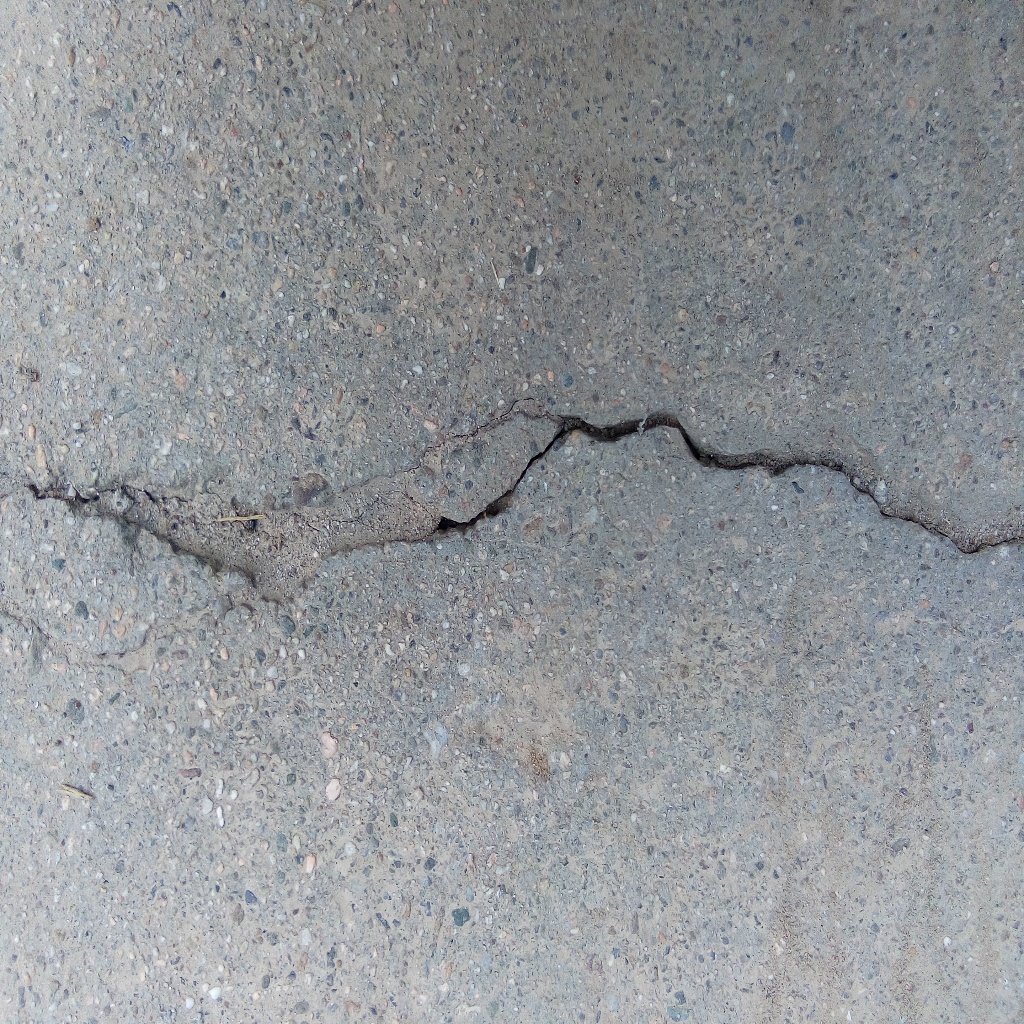

Supplement: S2 File — (ZIP) [file pone.0330218.s002.zip › 1 (364).jpg]

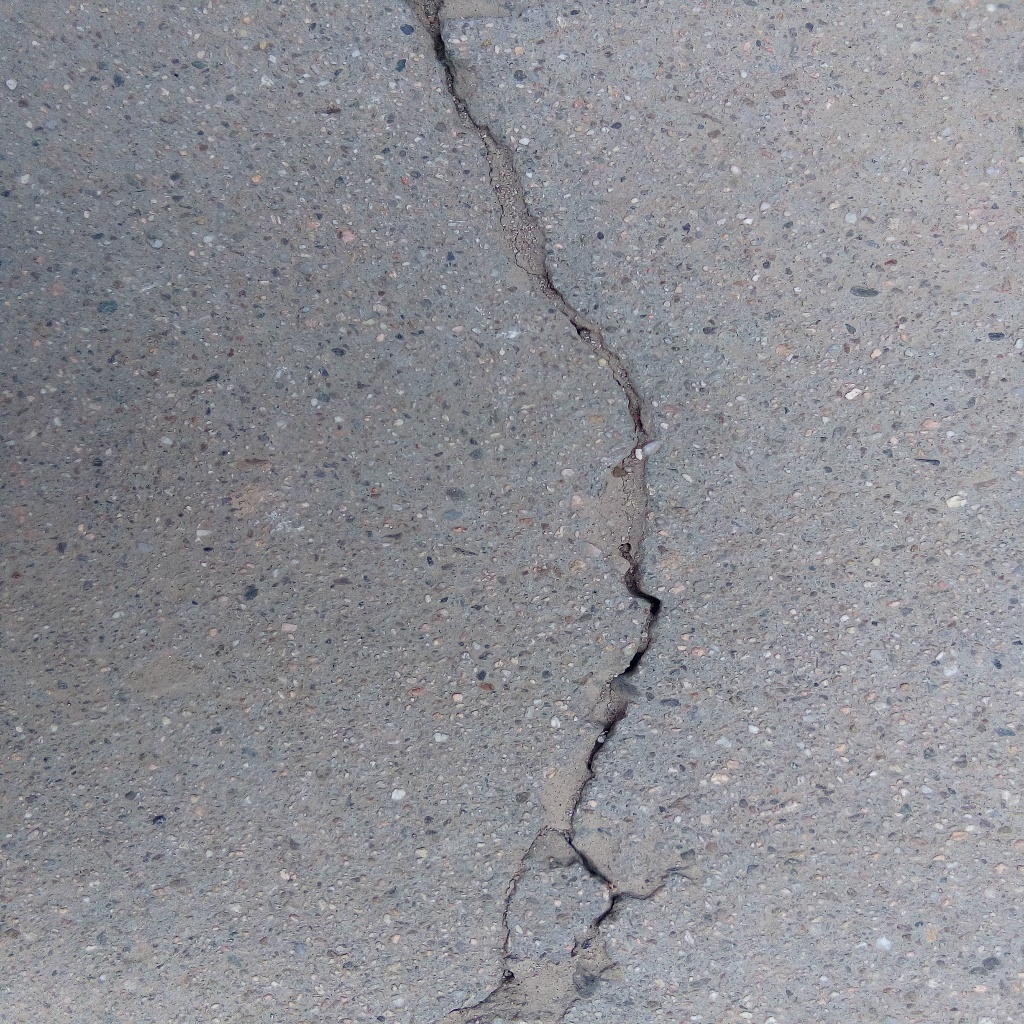

Supplement: S2 File — (ZIP) [file pone.0330218.s002.zip › 1 (365).jpg]

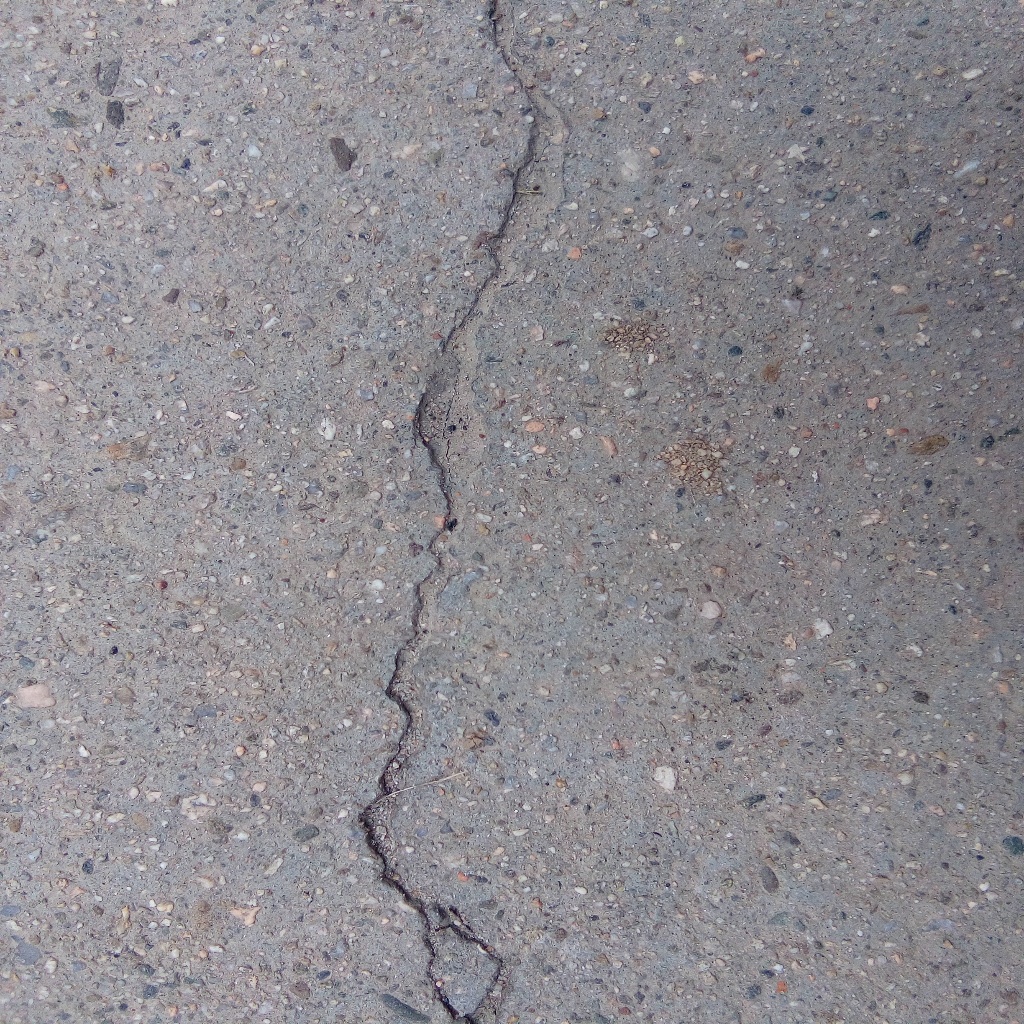

Supplement: S2 File — (ZIP) [file pone.0330218.s002.zip › 1 (406).jpg]

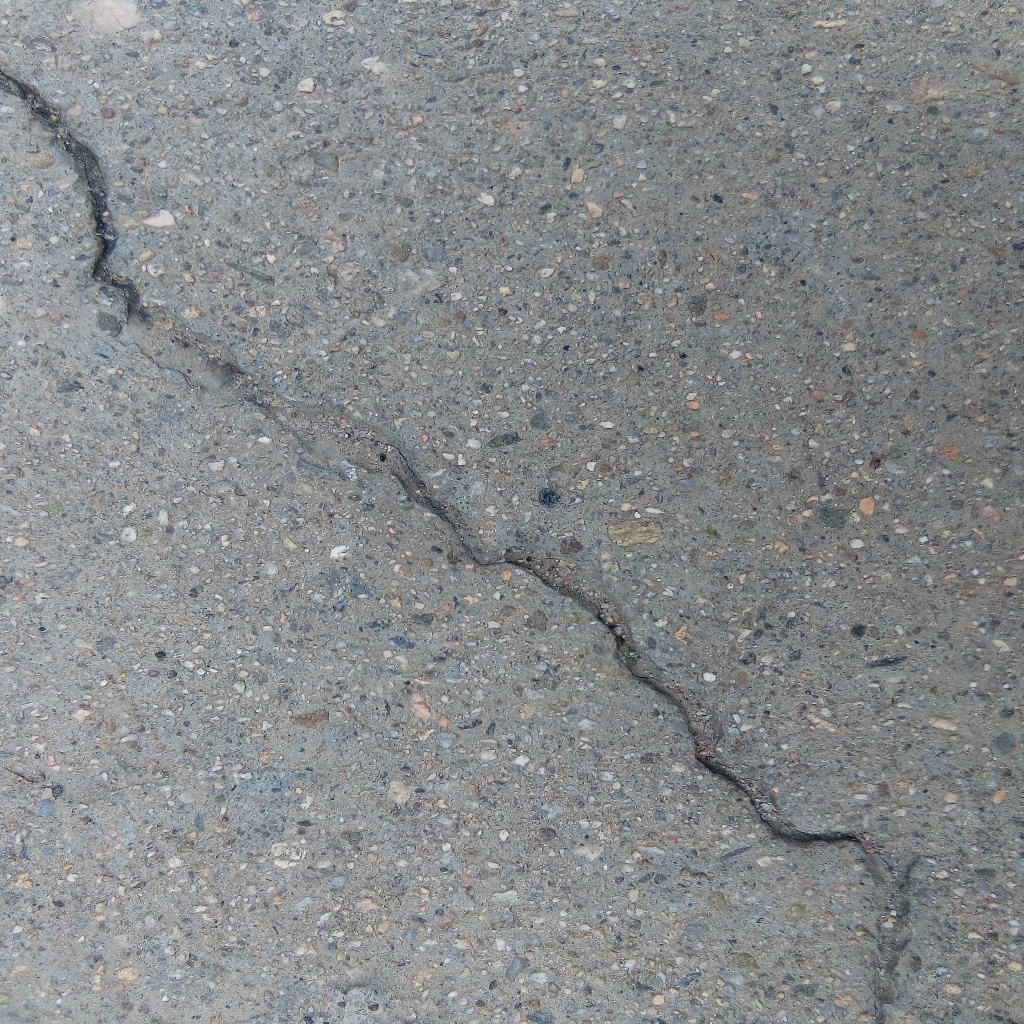

Supplement: S2 File — (ZIP) [file pone.0330218.s002.zip › 1 (440).jpg]

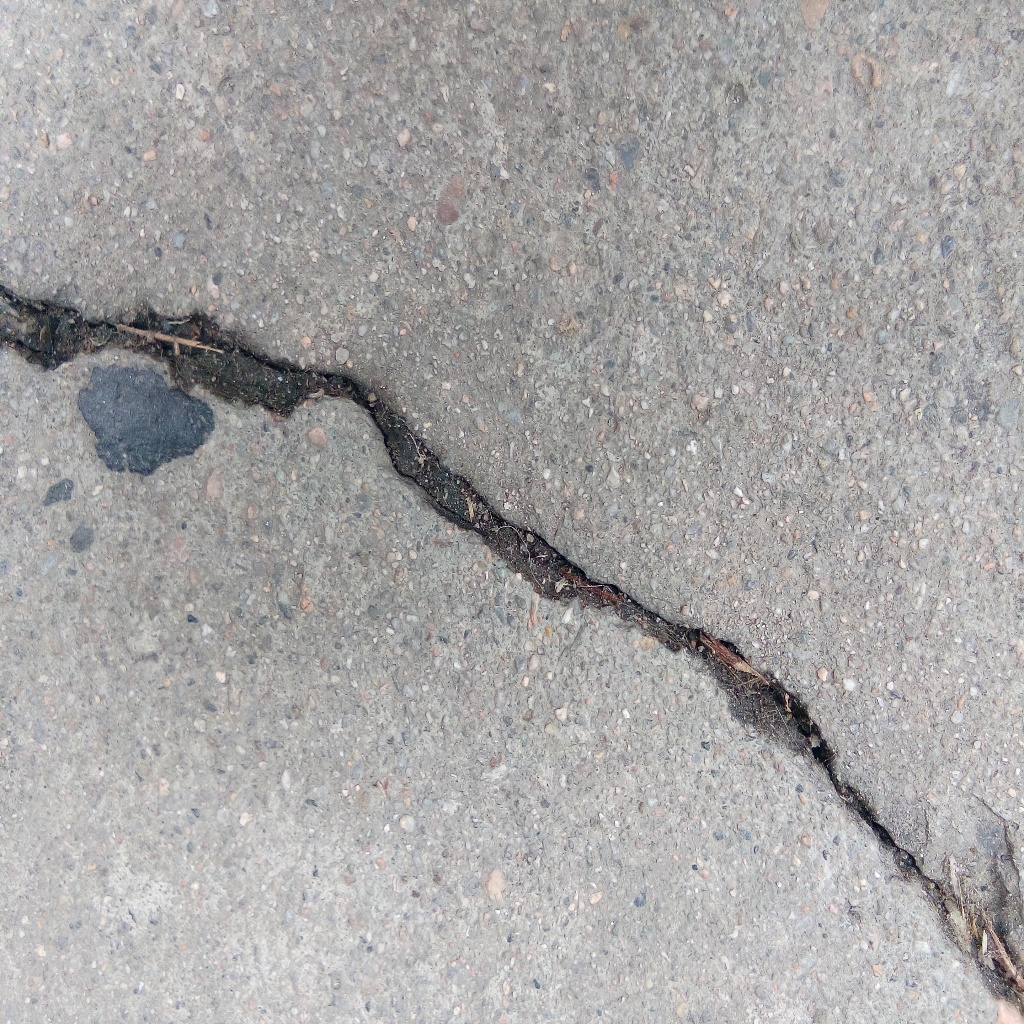

Supplement: S2 File — (ZIP) [file pone.0330218.s002.zip › 1 (460).jpg]

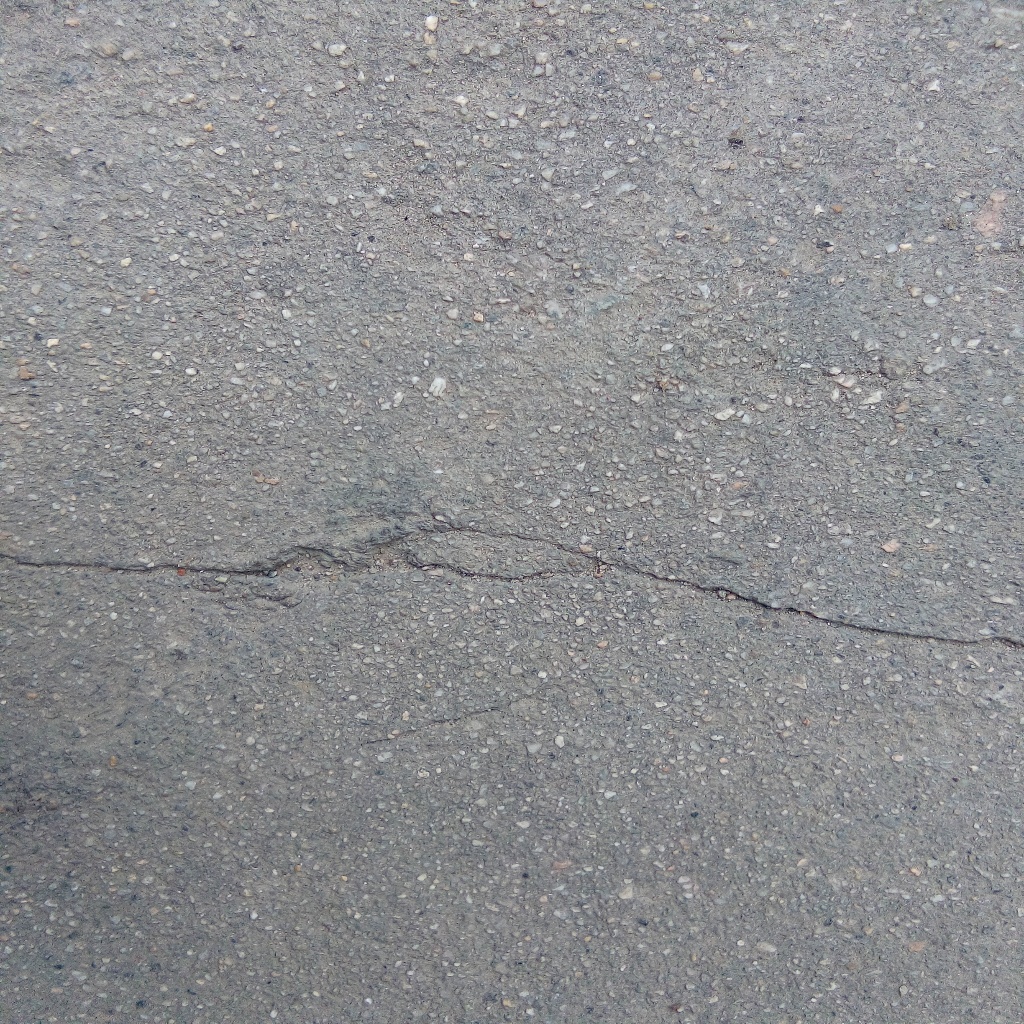

Supplement: S2 File — (ZIP) [file pone.0330218.s002.zip › 1 (466).jpg]

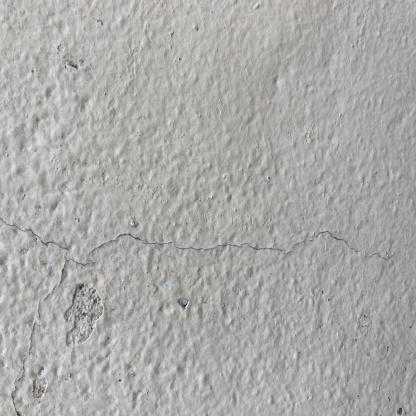

Supplement: S2 File — (ZIP) [file pone.0330218.s002.zip › 1616.rf.c868709931a671796794fdbb95352c5a.jpg]

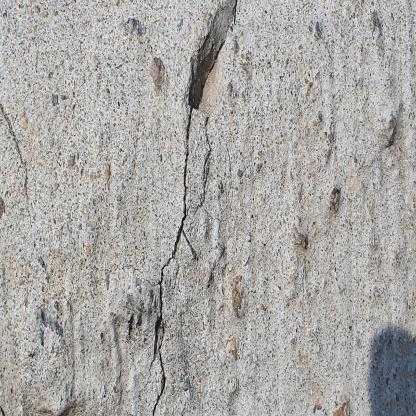

Supplement: S2 File — (ZIP) [file pone.0330218.s002.zip › 1675.rf.e3aa3f8d28d0247ef0284dd46dacc29f.jpg]

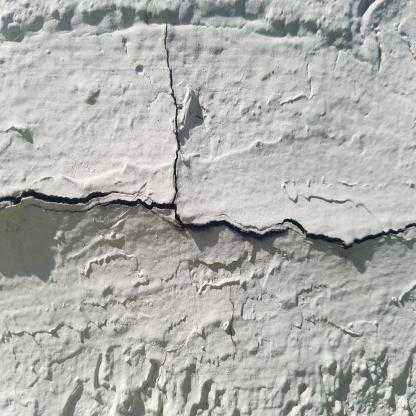

Supplement: S2 File — (ZIP) [file pone.0330218.s002.zip › 1686.rf.809fb1b51c607e5cf787e44ef4ddd7b8.jpg]

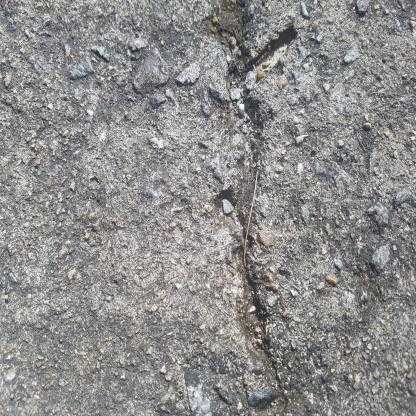

Supplement: S2 File — (ZIP) [file pone.0330218.s002.zip › 1706.rf.011d213c21ec78896c36728dcbc156f5.jpg]

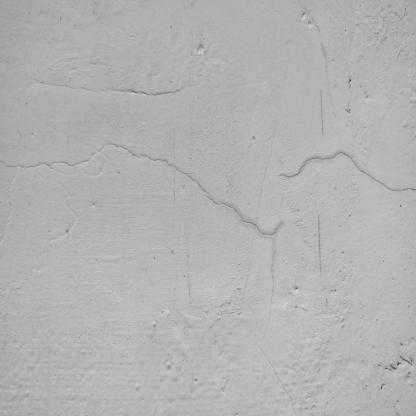

Supplement: S2 File — (ZIP) [file pone.0330218.s002.zip › 1716.rf.85ea38b36008beaa72c5d8541f734eb0.jpg]

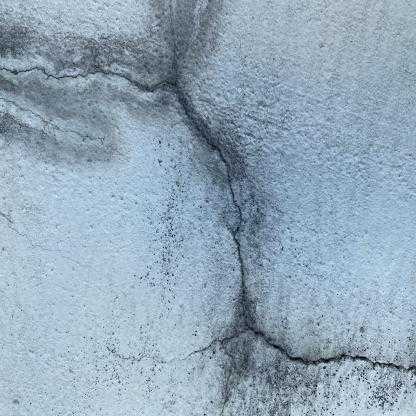

Supplement: S2 File — (ZIP) [file pone.0330218.s002.zip › 1722.rf.38b38f2e833309a4f35bfbf0432dffff.jpg]

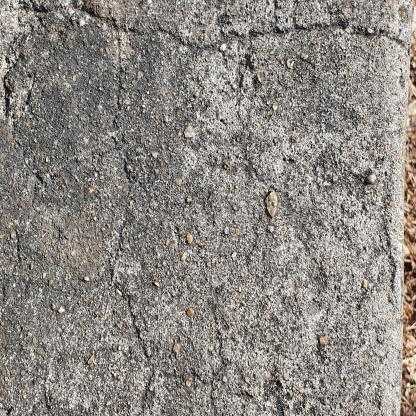

Supplement: S2 File — (ZIP) [file pone.0330218.s002.zip › 1794.rf.7a03ca09d05e9e2941f768bc8570cb54.jpg]

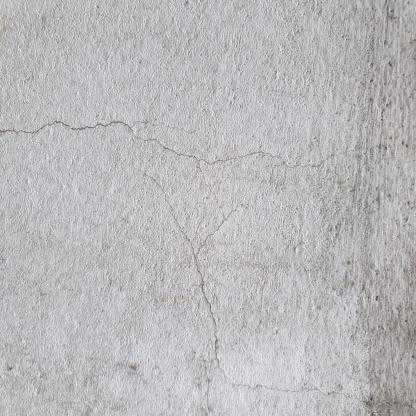

Supplement: S2 File — (ZIP) [file pone.0330218.s002.zip › 1804.rf.7e39a73f7b0d1c4bdf8094006e0cf495.jpg]

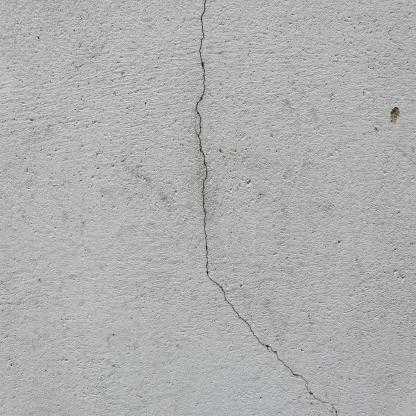

Supplement: S2 File — (ZIP) [file pone.0330218.s002.zip › 1806.rf.8c87862452f4ce0232a666c180ec9942.jpg]

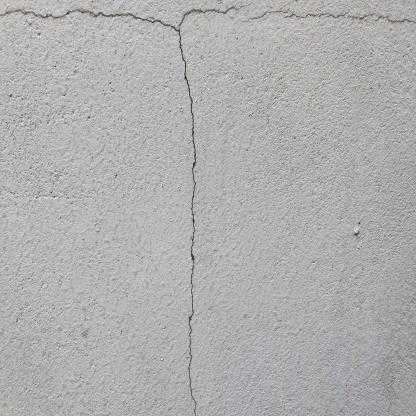

Supplement: S2 File — (ZIP) [file pone.0330218.s002.zip › 1813.rf.79f1af872f76dd8b46df4a83ed5f54c6.jpg]

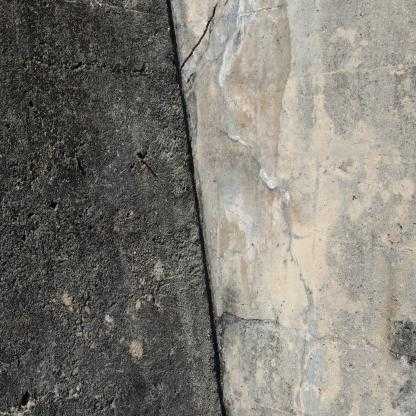

Supplement: S2 File — (ZIP) [file pone.0330218.s002.zip › 1818.rf.7e52c1687bfb625e86c54c9d71a66d99.jpg]

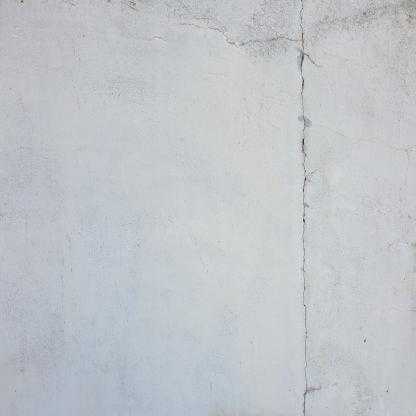

Supplement: S2 File — (ZIP) [file pone.0330218.s002.zip › 1819.rf.d2d41865c85e1019dc3e8b9daf73c434.jpg]

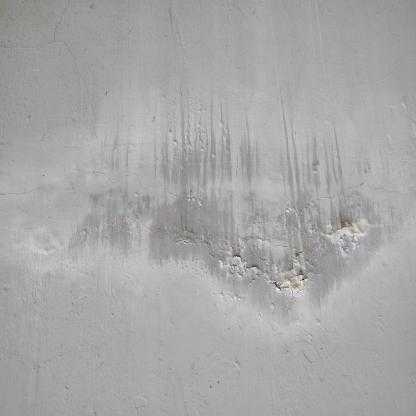

Supplement: S2 File — (ZIP) [file pone.0330218.s002.zip › 1839.rf.3495cbba8ee9a583ed0dff8d0e5fc0f7.jpg]

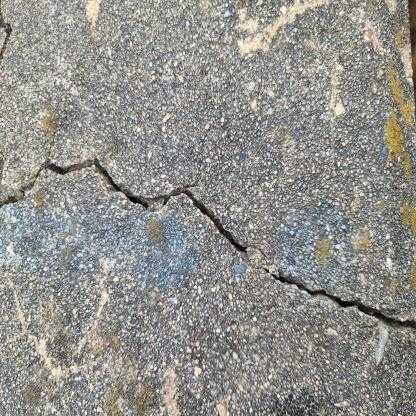

Supplement: S2 File — (ZIP) [file pone.0330218.s002.zip › 1896.rf.e718dd26abc98c2645ea37a808039034.jpg]

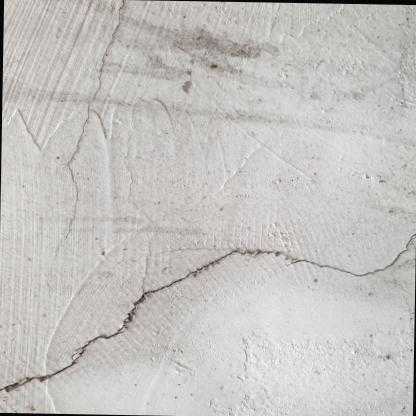

Supplement: S2 File — (ZIP) [file pone.0330218.s002.zip › 1899.rf.05853f25ccaa4fe0a74c0fa674106547.jpg]

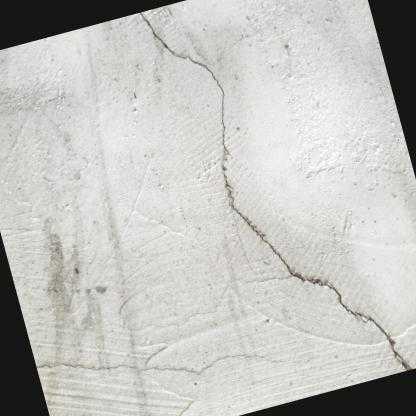

Supplement: S2 File — (ZIP) [file pone.0330218.s002.zip › 1899.rf.8a93805fbfc20cdad1fb03376b6233a1.jpg]

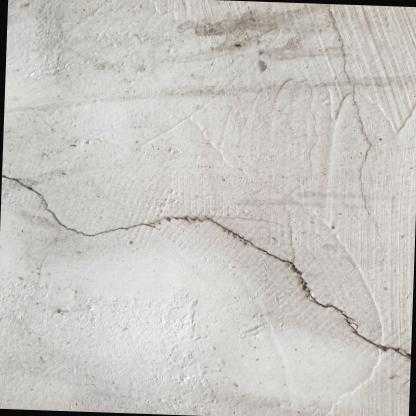

Supplement: S2 File — (ZIP) [file pone.0330218.s002.zip › 1899.rf.c05583a0d1327f794ed389b8932cbd8a.jpg]

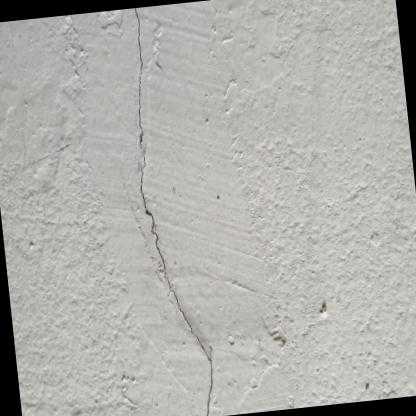

Supplement: S2 File — (ZIP) [file pone.0330218.s002.zip › 1901.rf.0b5b87ee5d3a31b27ac9ba0d602c066a.jpg]

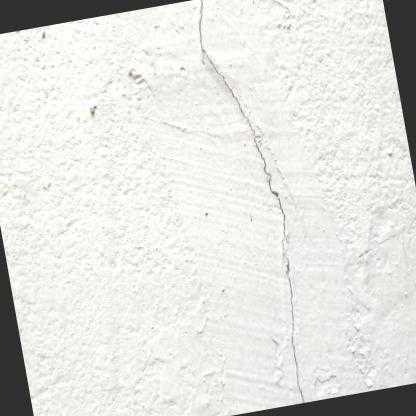

Supplement: S2 File — (ZIP) [file pone.0330218.s002.zip › 1901.rf.4440f2b6662d5cc8cd32afd7c5541aa9.jpg]

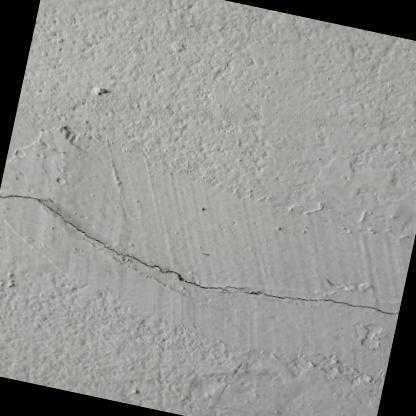

Supplement: S2 File — (ZIP) [file pone.0330218.s002.zip › 1901.rf.c431762b416338616fe9726e06fac419.jpg]

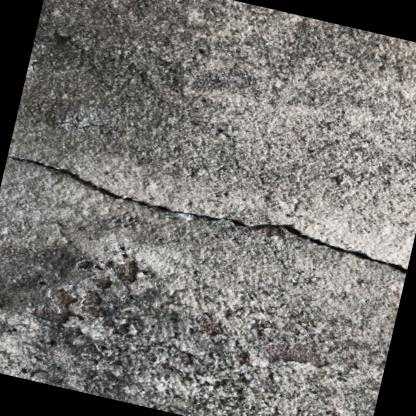

Supplement: S2 File — (ZIP) [file pone.0330218.s002.zip › 1903.rf.42f0b145a662953cc954281f099f5dc7.jpg]

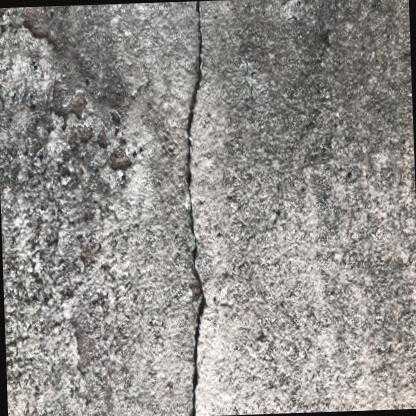

Supplement: S2 File — (ZIP) [file pone.0330218.s002.zip › 1903.rf.f39c77cf88a182bf267e78b08049529a.jpg]

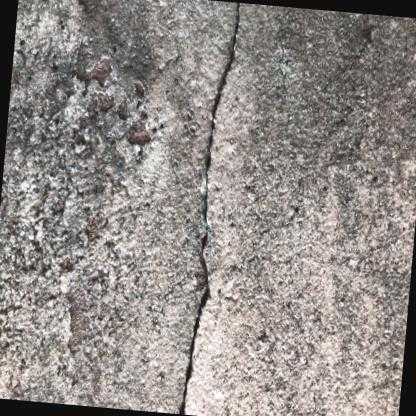

Supplement: S2 File — (ZIP) [file pone.0330218.s002.zip › 1903.rf.fcde61244262d1033370ebc1666d621d.jpg]

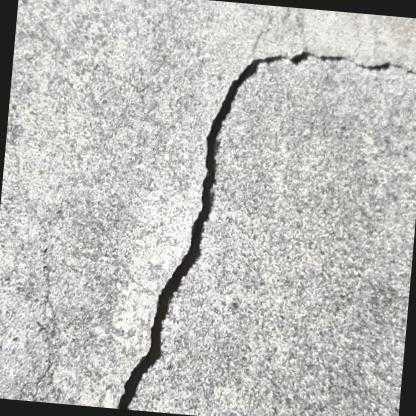

Supplement: S2 File — (ZIP) [file pone.0330218.s002.zip › 1904.rf.2385f6f6e78df7355637719a395e3147.jpg]

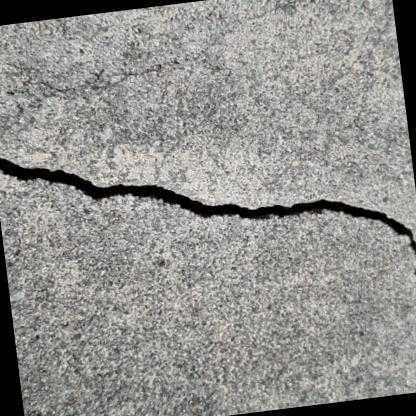

Supplement: S2 File — (ZIP) [file pone.0330218.s002.zip › 1904.rf.7afe2fb6a925cd90ea1adfe97966ef15.jpg]

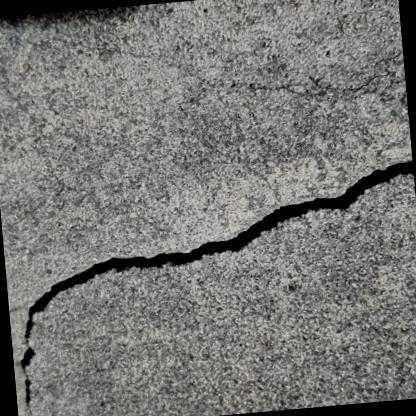

Supplement: S2 File — (ZIP) [file pone.0330218.s002.zip › 1904.rf.8df525de17f98443658fb6776a38ece5.jpg]

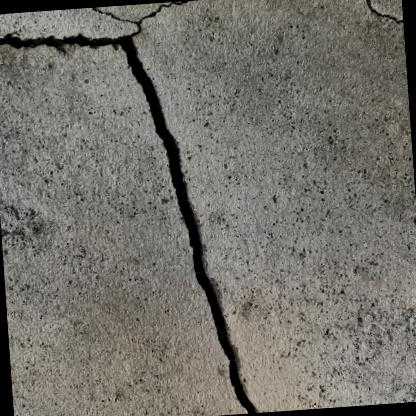

Supplement: S2 File — (ZIP) [file pone.0330218.s002.zip › 1906.rf.449130a9b23338145e698ea28e827aa5.jpg]

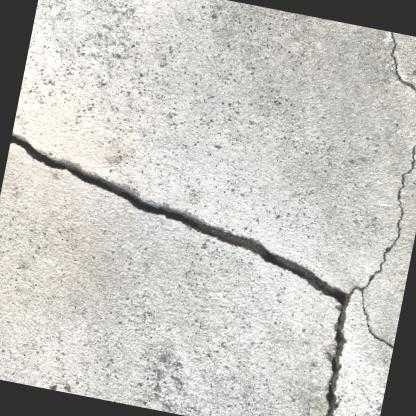

Supplement: S2 File — (ZIP) [file pone.0330218.s002.zip › 1906.rf.58b3bf4d6f1c68228ecc260358d6a55d.jpg]

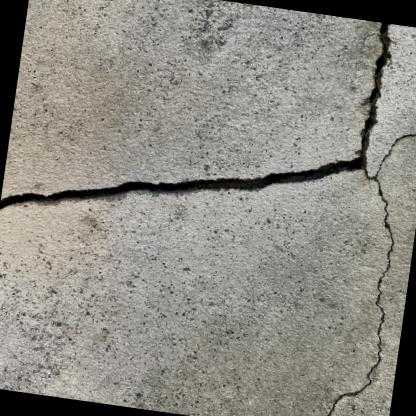

Supplement: S2 File — (ZIP) [file pone.0330218.s002.zip › 1906.rf.da2f5dad3bf32d22d3fcefdda835fc53.jpg]

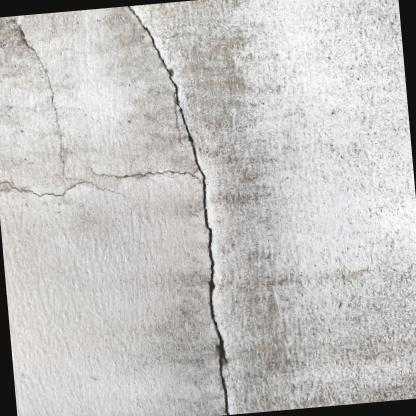

Supplement: S2 File — (ZIP) [file pone.0330218.s002.zip › 1907.rf.4fd41c5883540f708964f149135bd6ae.jpg]

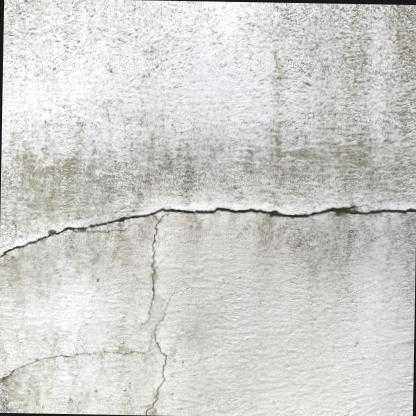

Supplement: S2 File — (ZIP) [file pone.0330218.s002.zip › 1907.rf.85a0cb730aa41741b3c9a68b07ce9eaa.jpg]

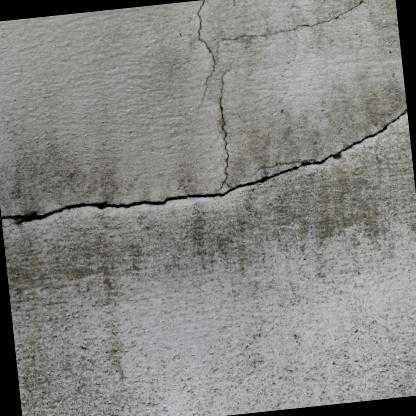

Supplement: S2 File — (ZIP) [file pone.0330218.s002.zip › 1907.rf.95e2d13e83355a0106502fde266a8a2e.jpg]

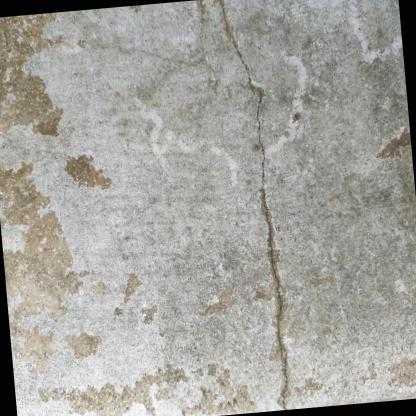

Supplement: S2 File — (ZIP) [file pone.0330218.s002.zip › 1909.rf.3aed37b6ab5b9e14651355859282f80f.jpg]

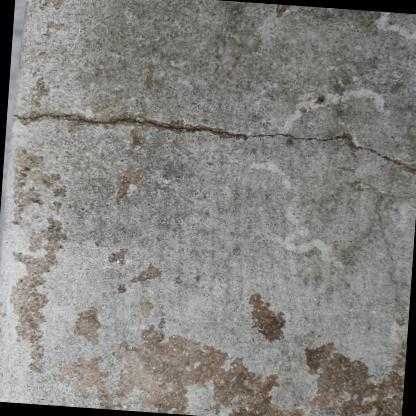

Supplement: S2 File — (ZIP) [file pone.0330218.s002.zip › 1909.rf.7d448f405a38454568bebe346cf3f2e3.jpg]

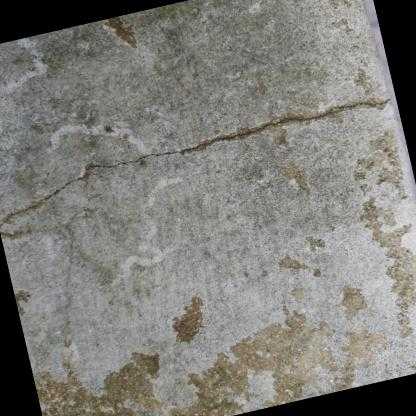

Supplement: S2 File — (ZIP) [file pone.0330218.s002.zip › 1909.rf.f91e86a77fa4e50dedfa76945f9d87b9.jpg]

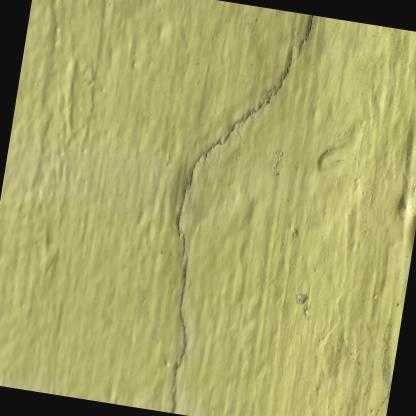

Supplement: S2 File — (ZIP) [file pone.0330218.s002.zip › 1910.rf.07aca26334eb51c26267cc6d045a3417.jpg]

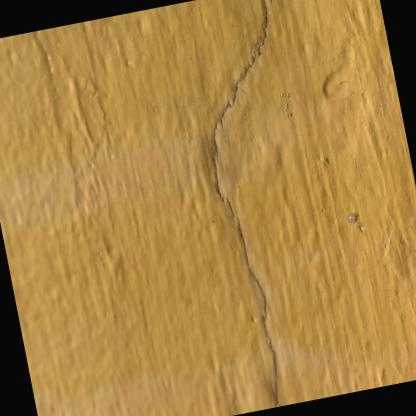

Supplement: S2 File — (ZIP) [file pone.0330218.s002.zip › 1910.rf.34b926c1893793845084b69361a346ea.jpg]

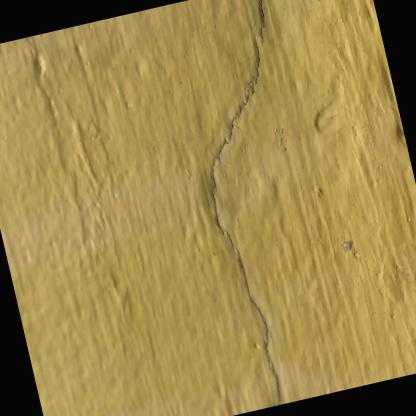

Supplement: S2 File — (ZIP) [file pone.0330218.s002.zip › 1910.rf.b65bee39f44270661f6853f0b3077617.jpg]

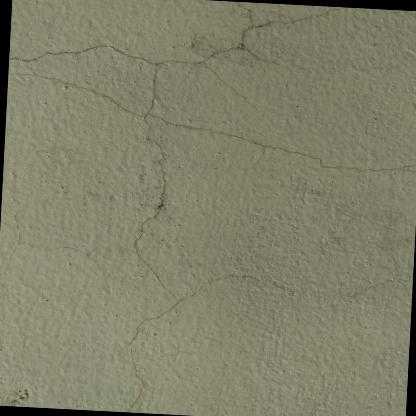

Supplement: S2 File — (ZIP) [file pone.0330218.s002.zip › 1915.rf.5e32f5823878513dde8ab3639ec98cdd.jpg]

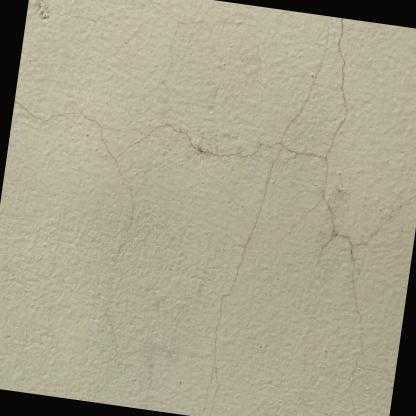

Supplement: S2 File — (ZIP) [file pone.0330218.s002.zip › 1915.rf.9ea941a0c185808b51887f710be6304c.jpg]
