# Supplementary figures and images for: Research on enhancing road apparent crack detection based on the improved YOLOv8n model (part 1 of 2)
Source: PLoS One. 2025 Sep 4;20(9):e0330218. doi: 10.1371/journal.pone.0330218 (PMC12410743; doi:10.1371/journal.pone.0330218)

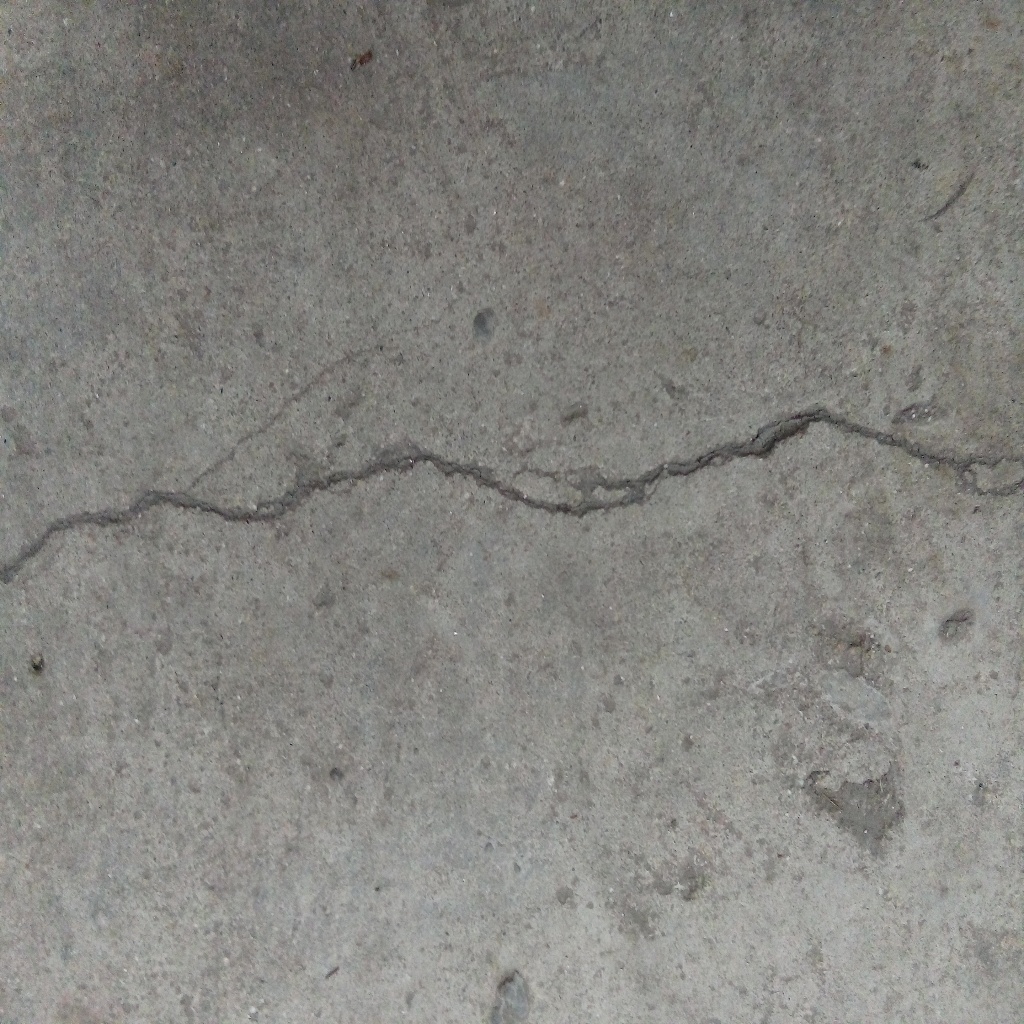

Supplement: S2 File — (ZIP) [file pone.0330218.s002.zip › 1 (1418).jpg]

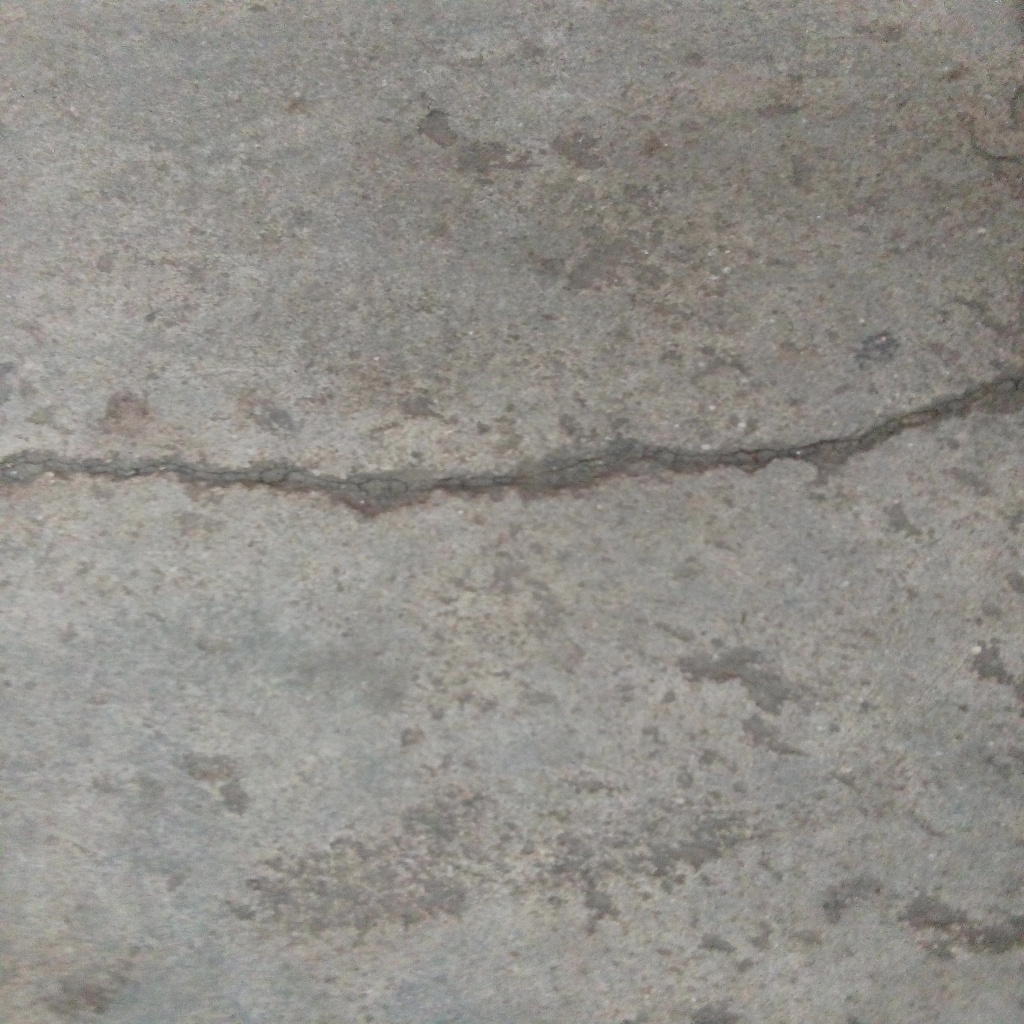

Supplement: S2 File — (ZIP) [file pone.0330218.s002.zip › 1 (1427).jpg]

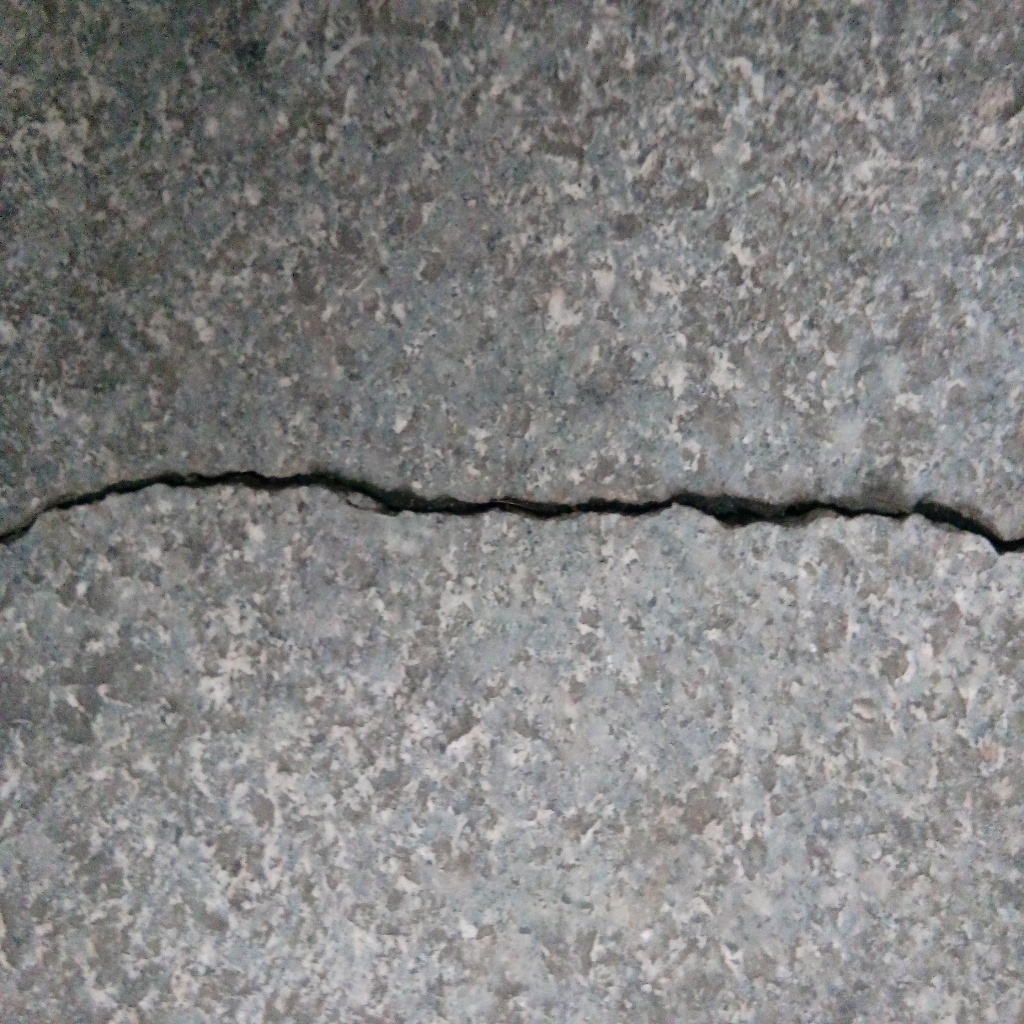

Supplement: S2 File — (ZIP) [file pone.0330218.s002.zip › 1 (1449).jpg]

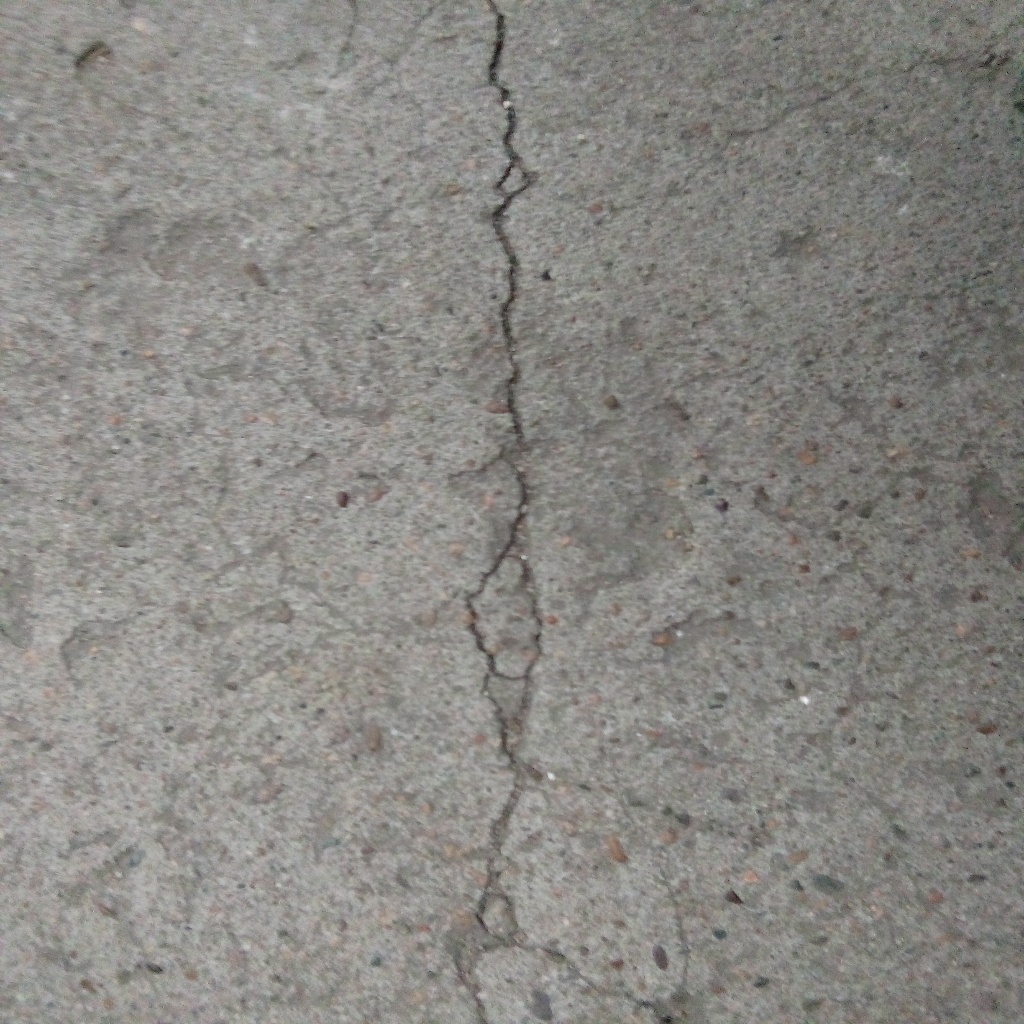

Supplement: S2 File — (ZIP) [file pone.0330218.s002.zip › 1 (1501).jpg]

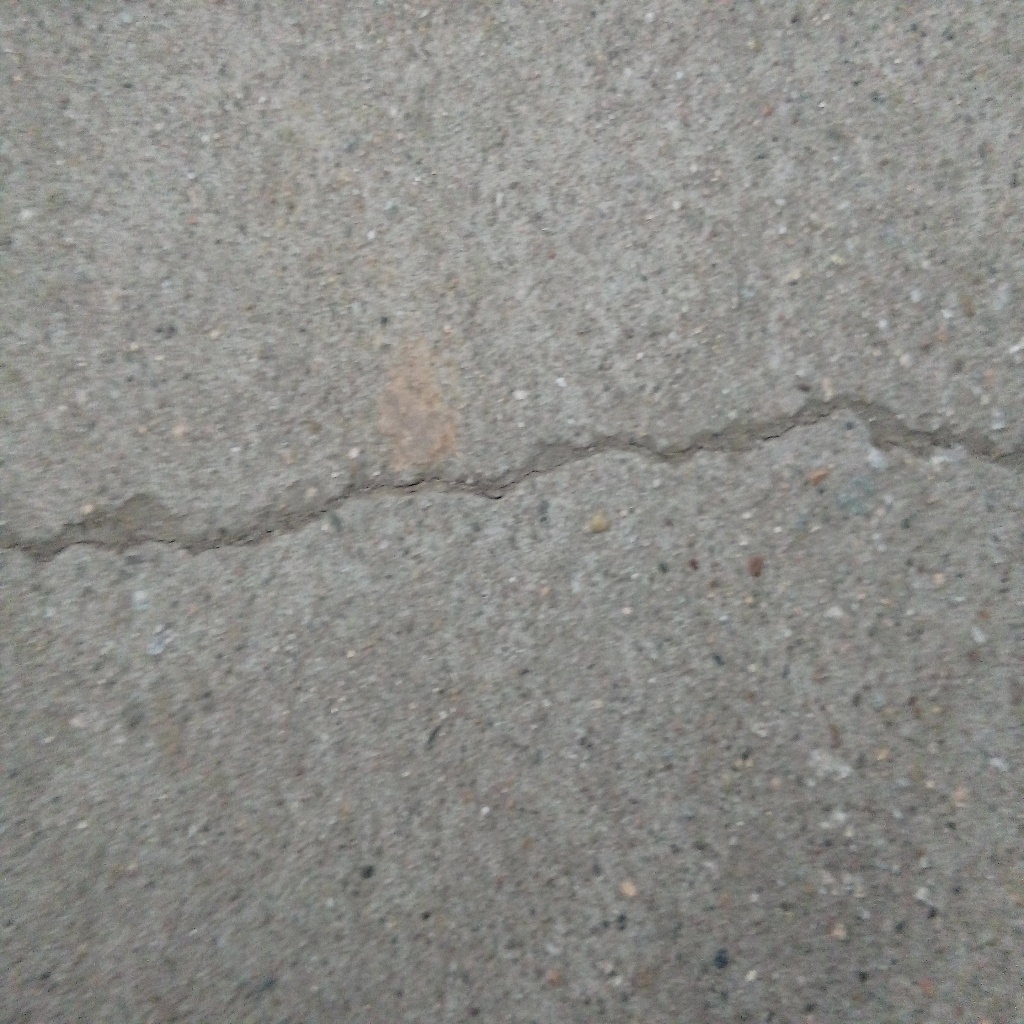

Supplement: S2 File — (ZIP) [file pone.0330218.s002.zip › 1 (1524).jpg]

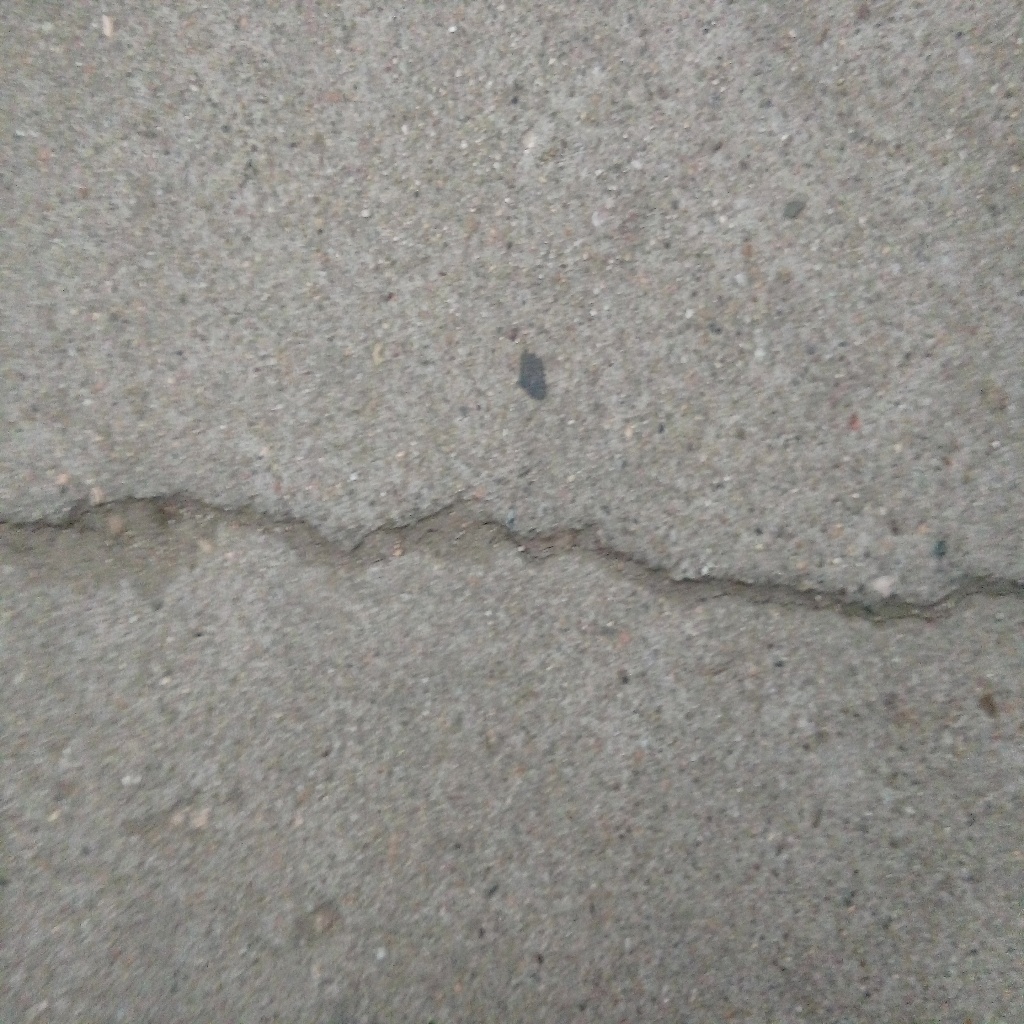

Supplement: S2 File — (ZIP) [file pone.0330218.s002.zip › 1 (1536).jpg]

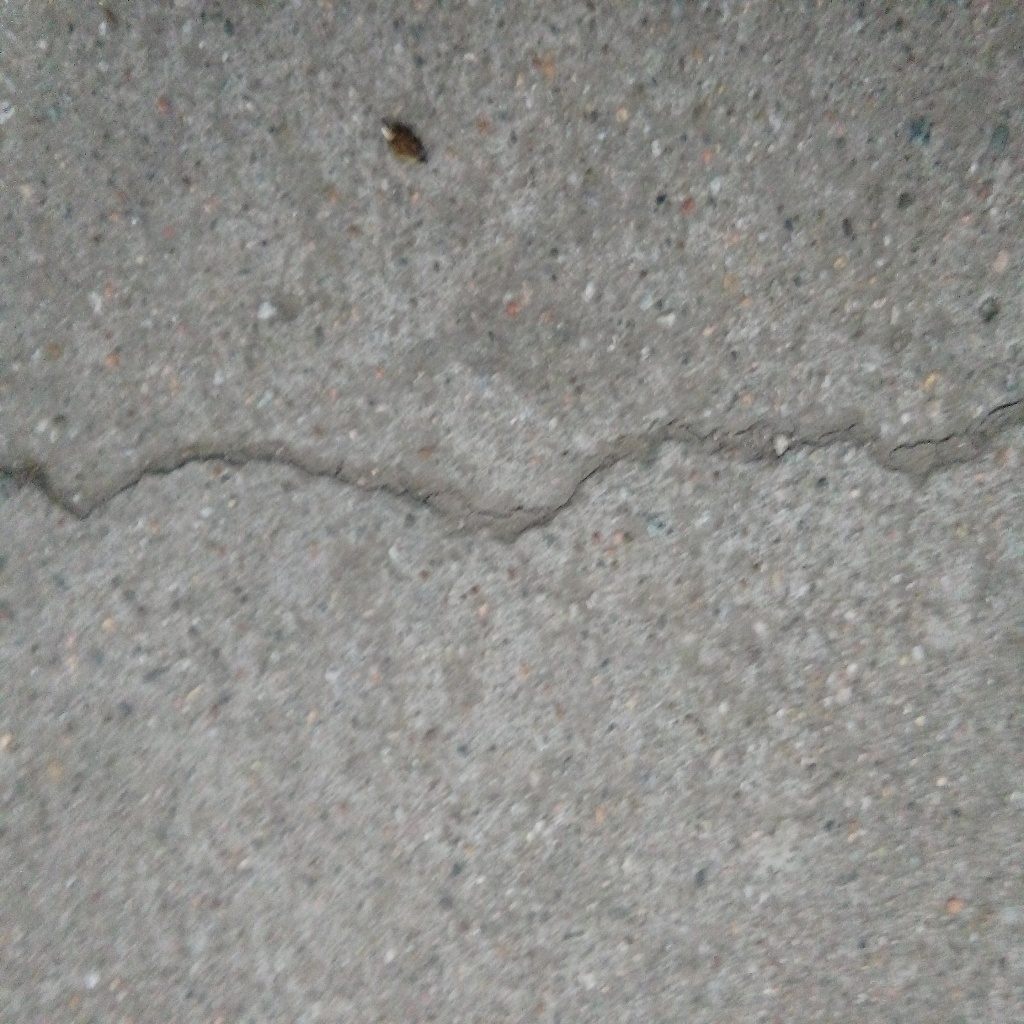

Supplement: S2 File — (ZIP) [file pone.0330218.s002.zip › 1 (1549).jpg]

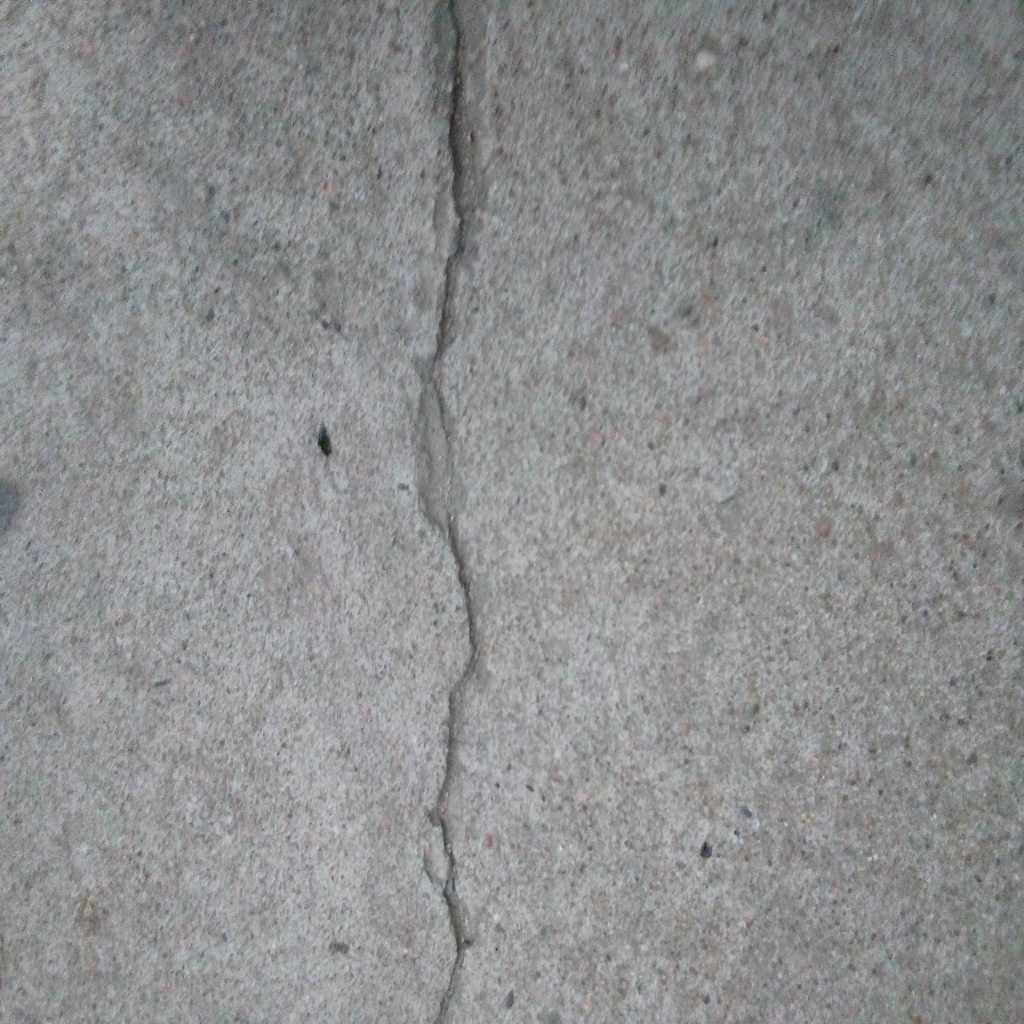

Supplement: S2 File — (ZIP) [file pone.0330218.s002.zip › 1 (1589).jpg]

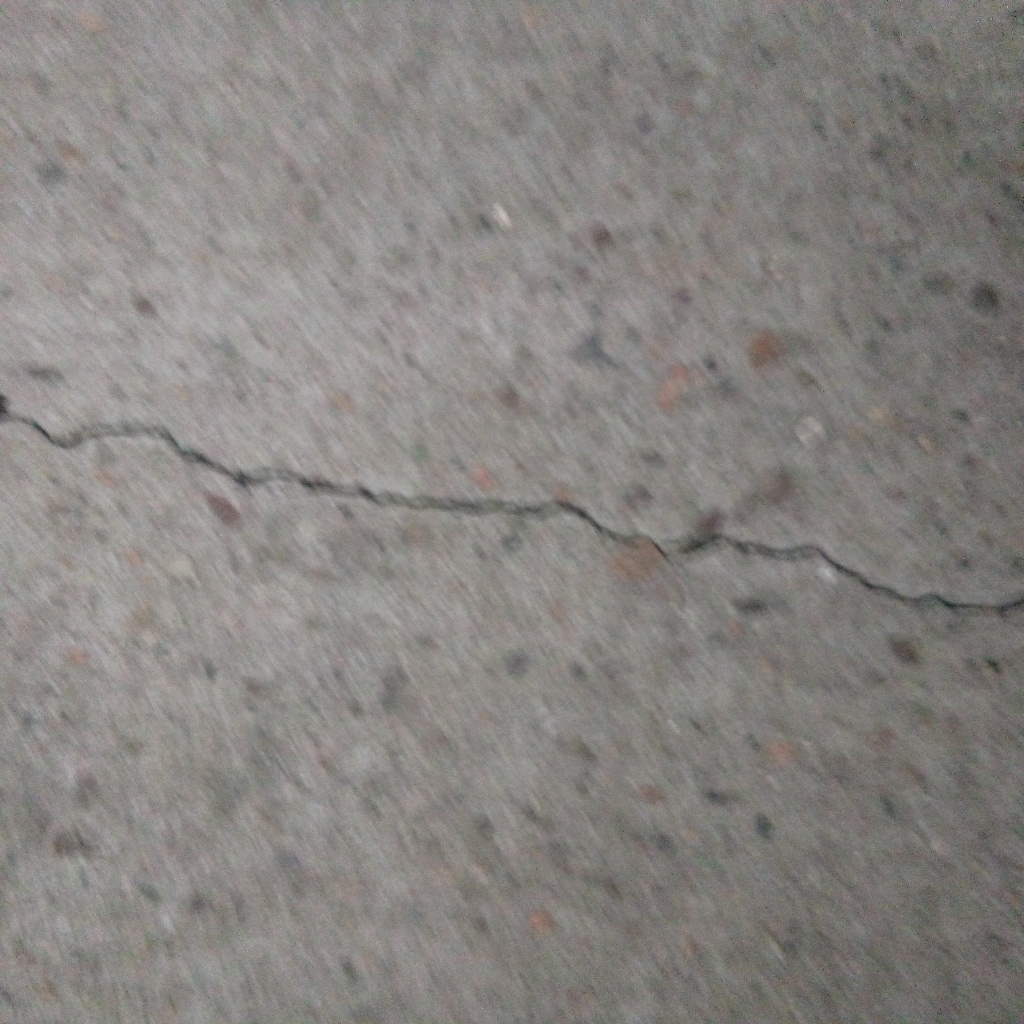

Supplement: S2 File — (ZIP) [file pone.0330218.s002.zip › 1 (1645).jpg]

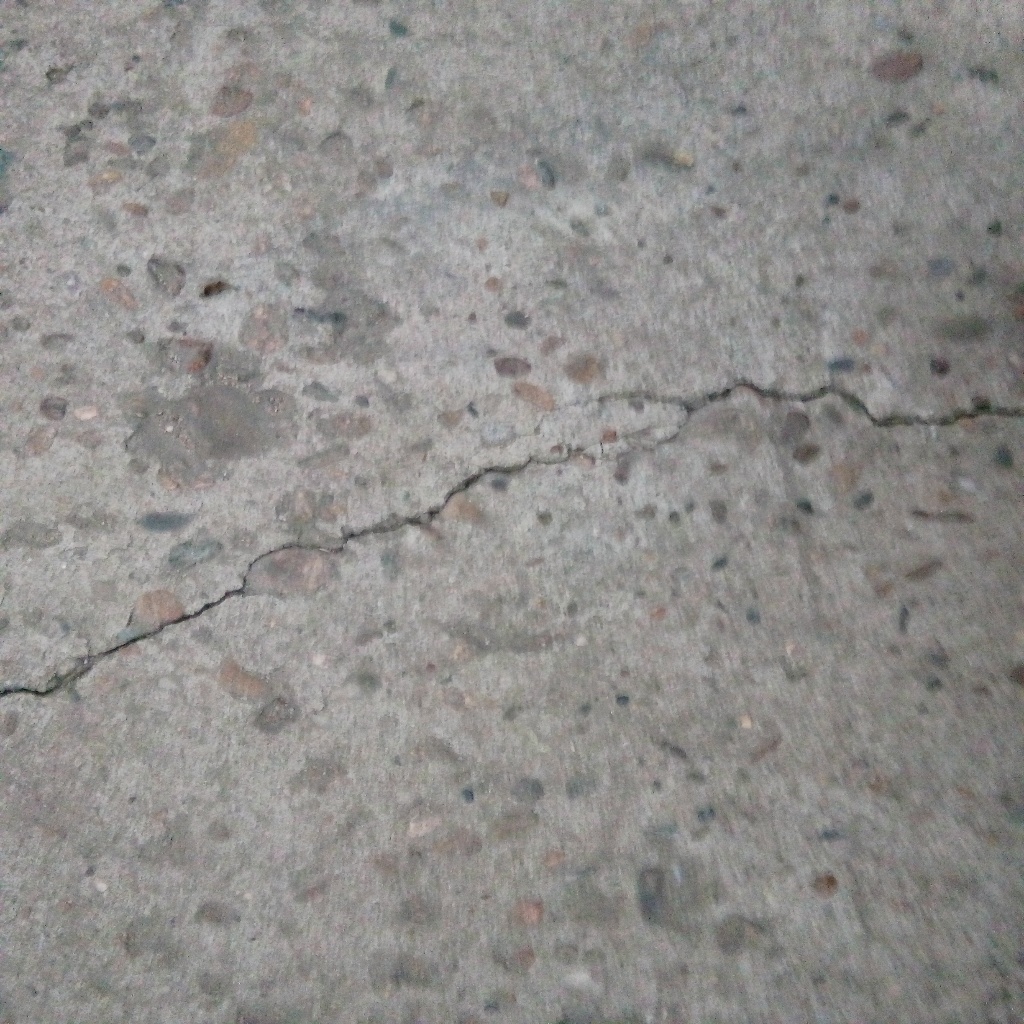

Supplement: S2 File — (ZIP) [file pone.0330218.s002.zip › 1 (1646).jpg]

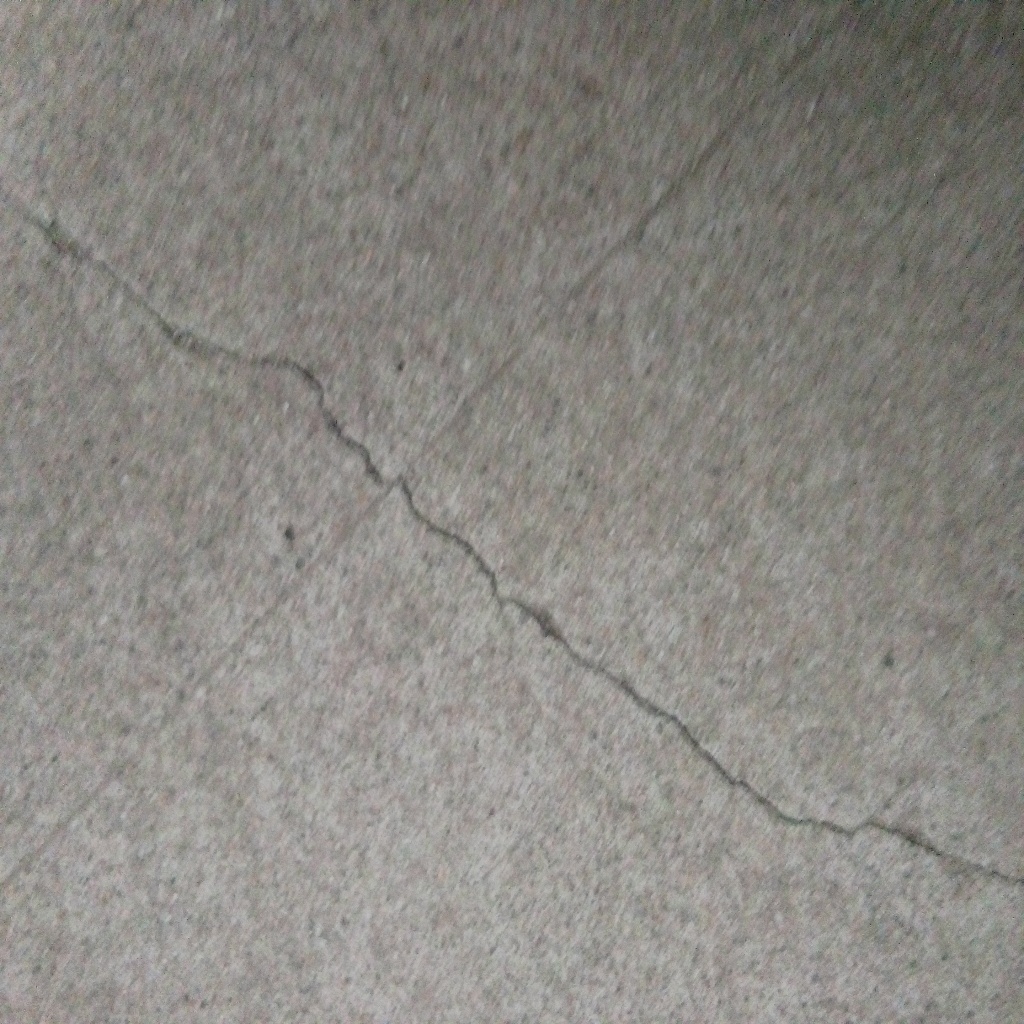

Supplement: S2 File — (ZIP) [file pone.0330218.s002.zip › 1 (1665).jpg]

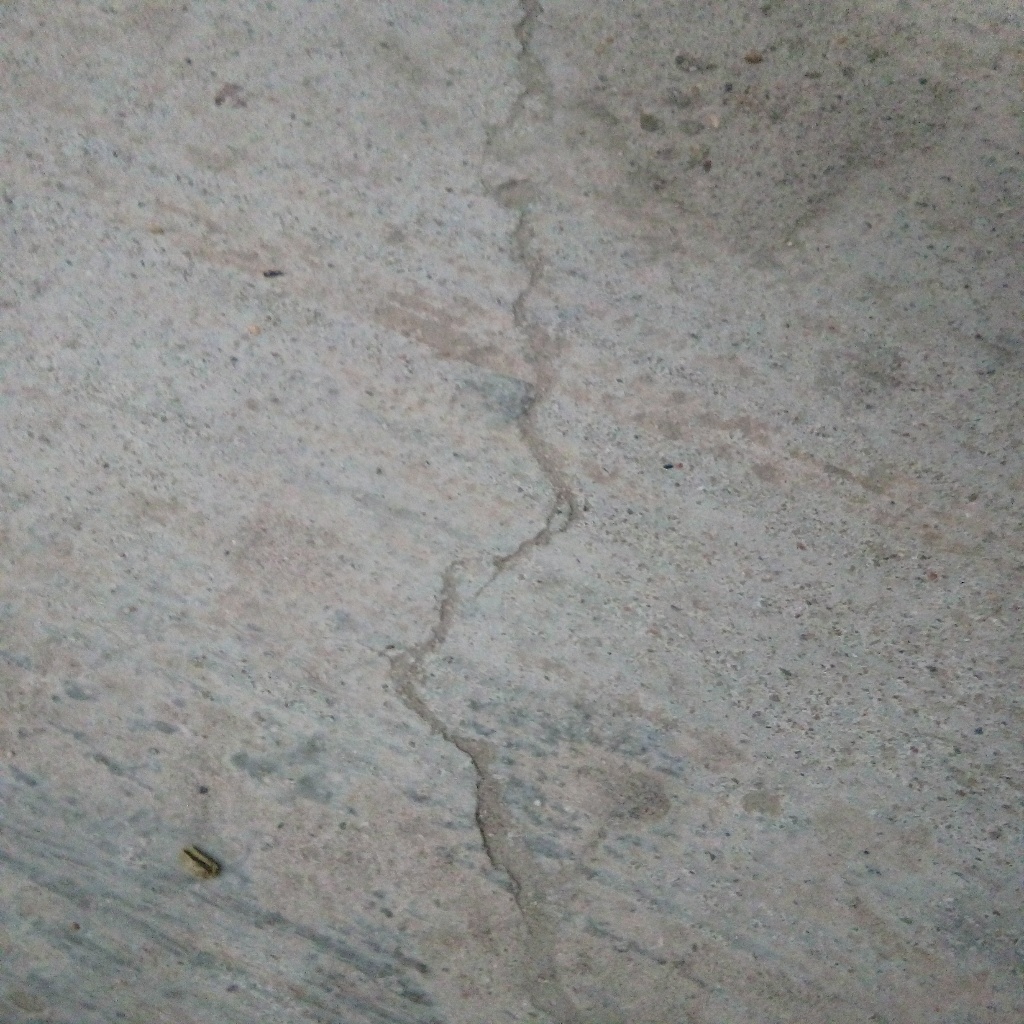

Supplement: S2 File — (ZIP) [file pone.0330218.s002.zip › 1 (1670).jpg]

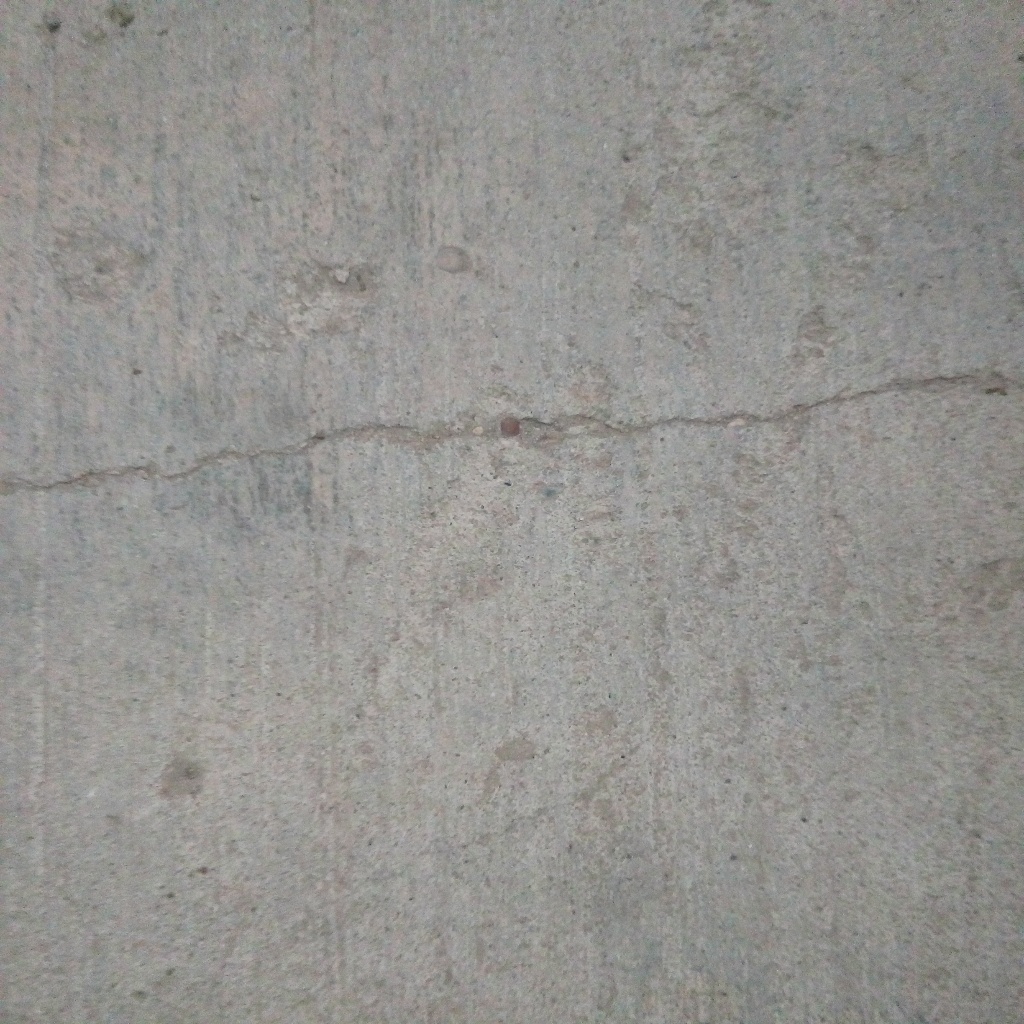

Supplement: S2 File — (ZIP) [file pone.0330218.s002.zip › 1 (1676).jpg]

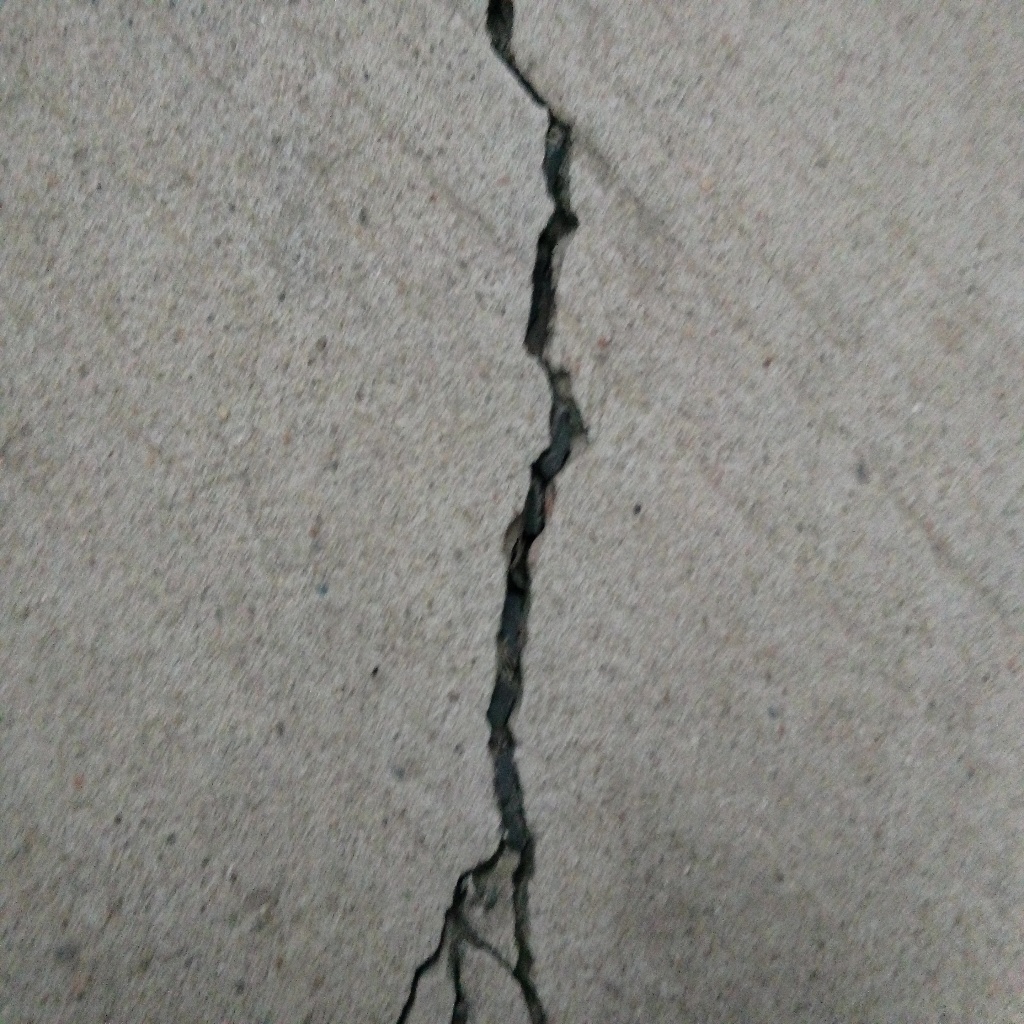

Supplement: S2 File — (ZIP) [file pone.0330218.s002.zip › 1 (1687).jpg]

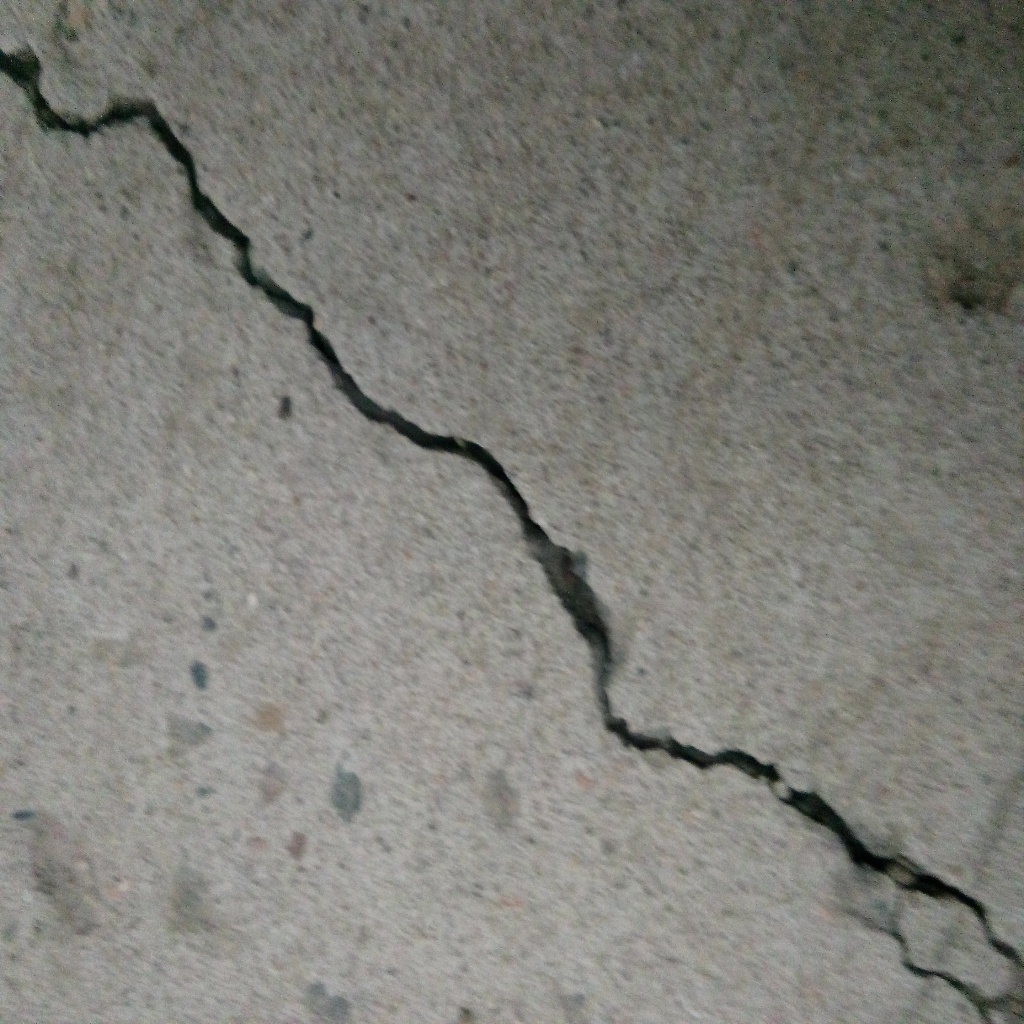

Supplement: S2 File — (ZIP) [file pone.0330218.s002.zip › 1 (1702).jpg]

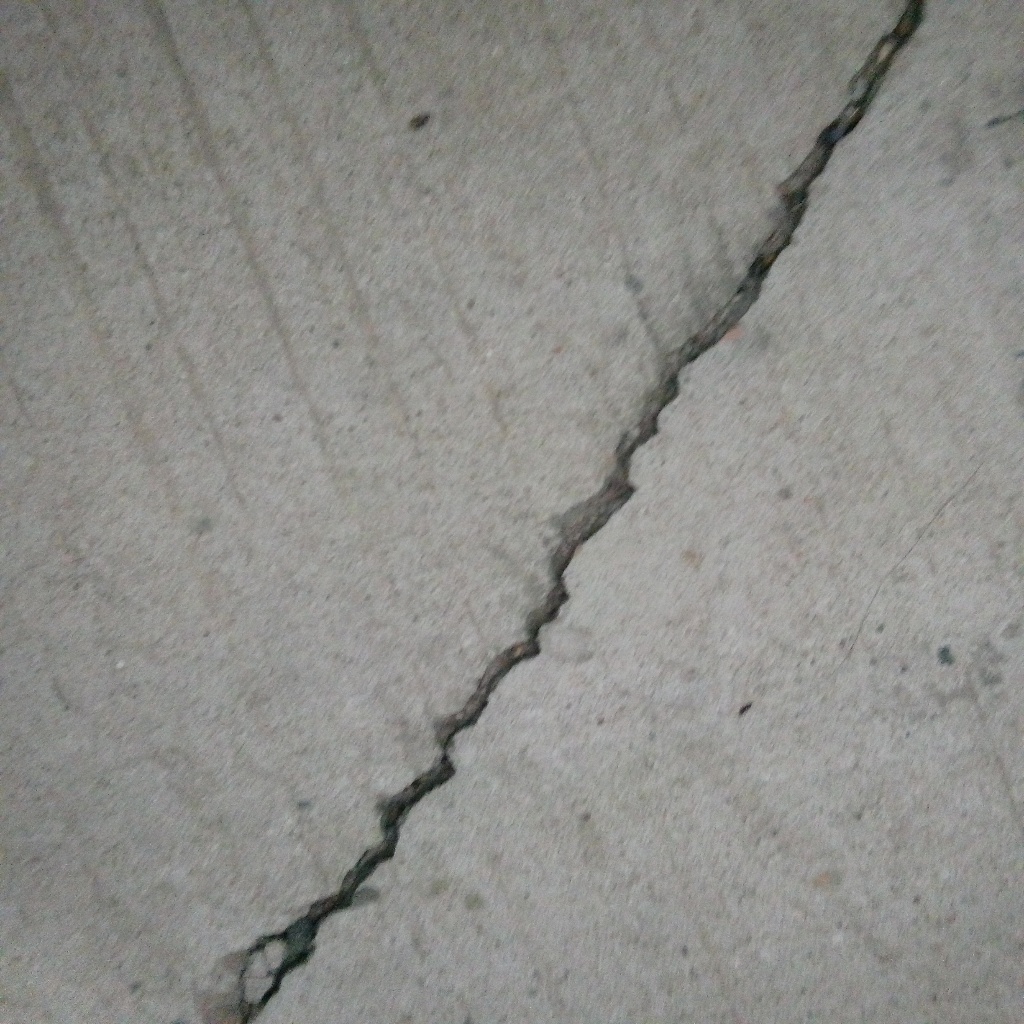

Supplement: S2 File — (ZIP) [file pone.0330218.s002.zip › 1 (1726).jpg]

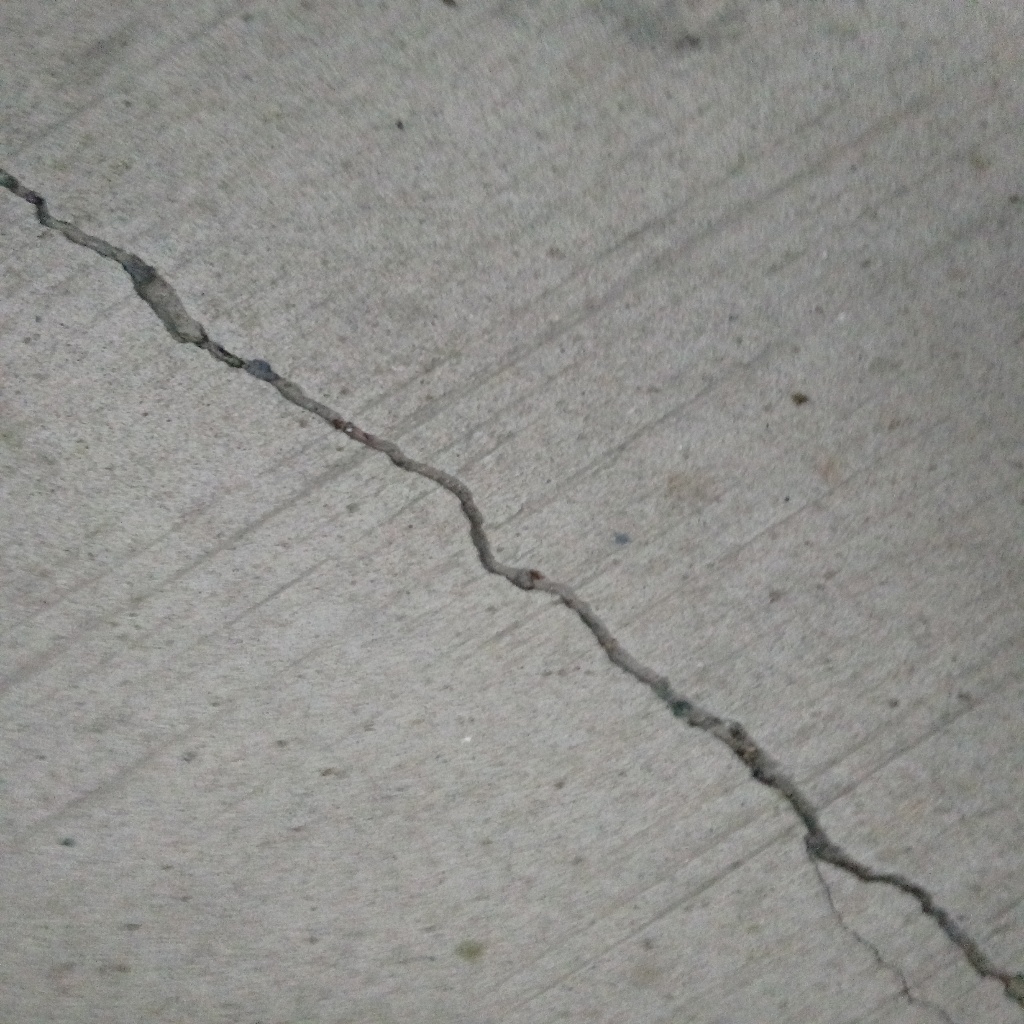

Supplement: S2 File — (ZIP) [file pone.0330218.s002.zip › 1 (1753).jpg]

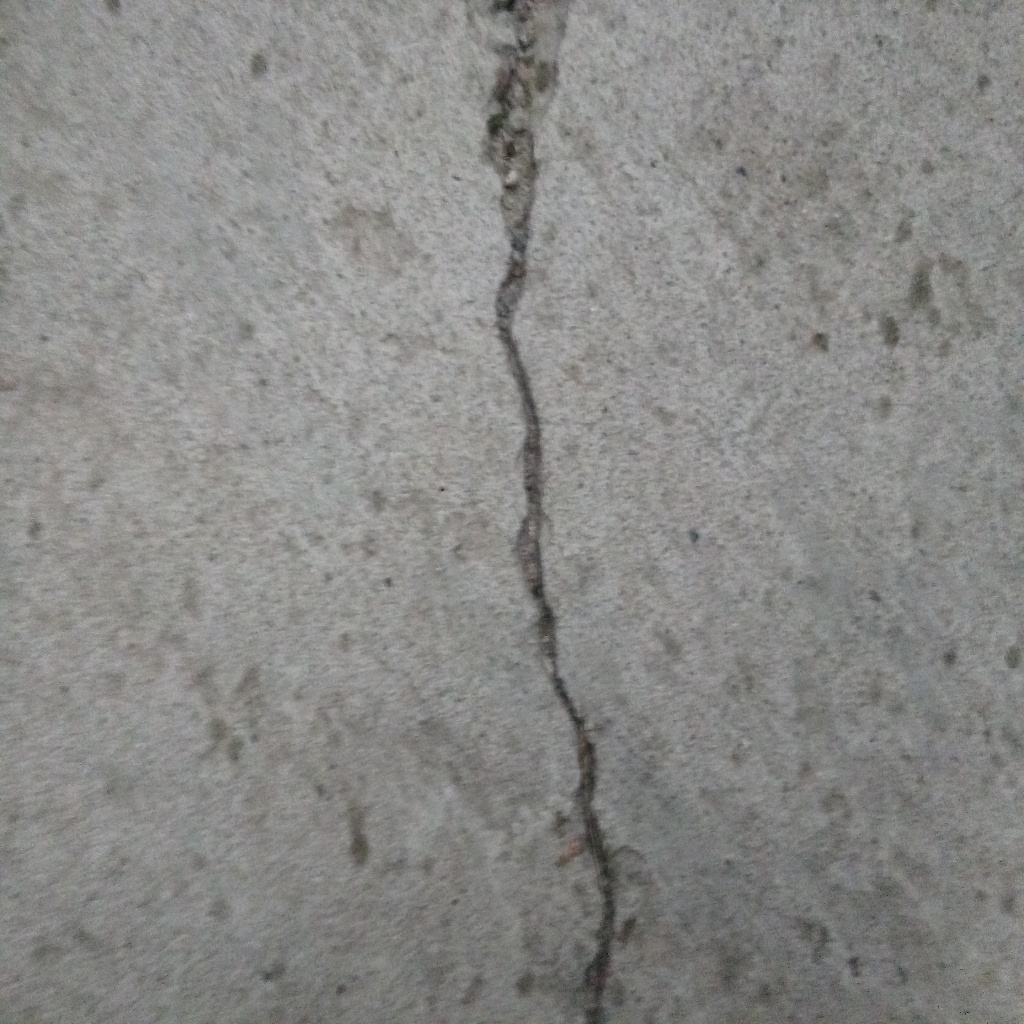

Supplement: S2 File — (ZIP) [file pone.0330218.s002.zip › 1 (1765).jpg]

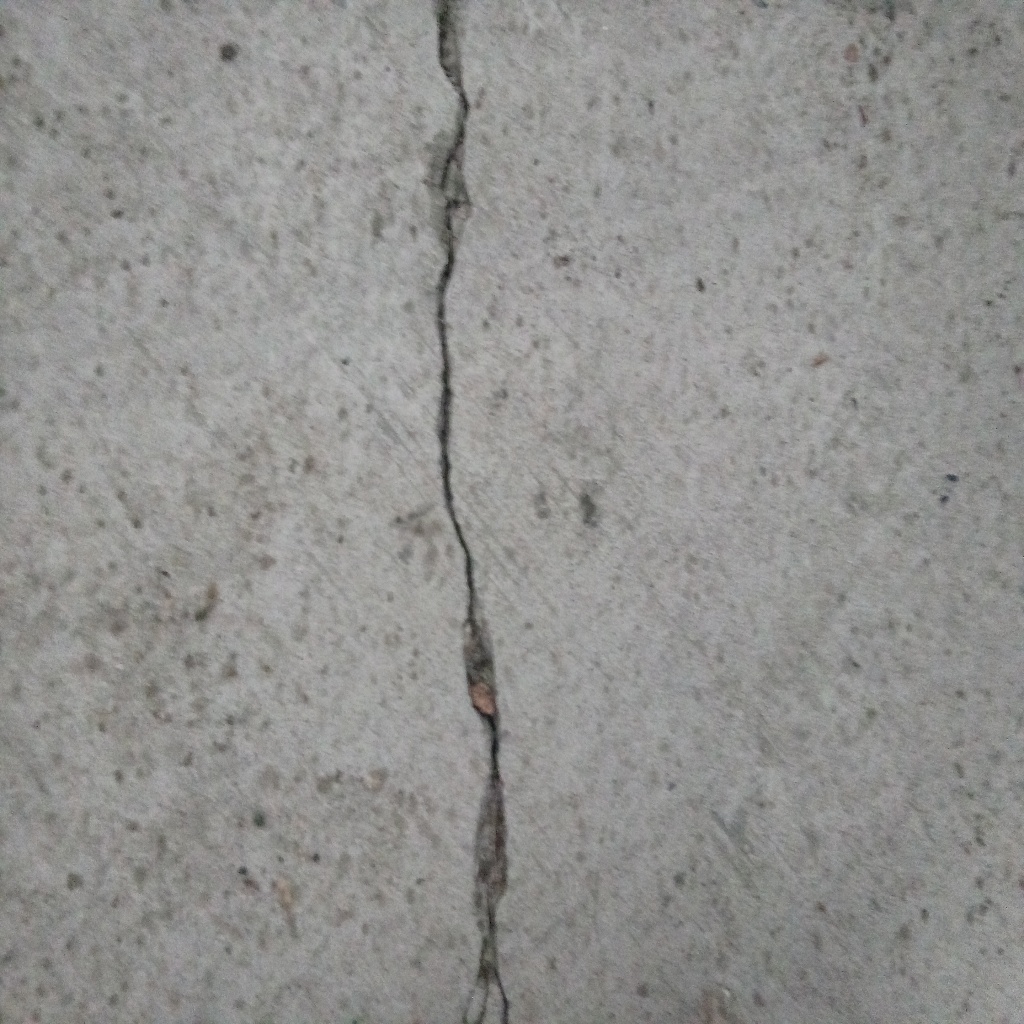

Supplement: S2 File — (ZIP) [file pone.0330218.s002.zip › 1 (1774).jpg]

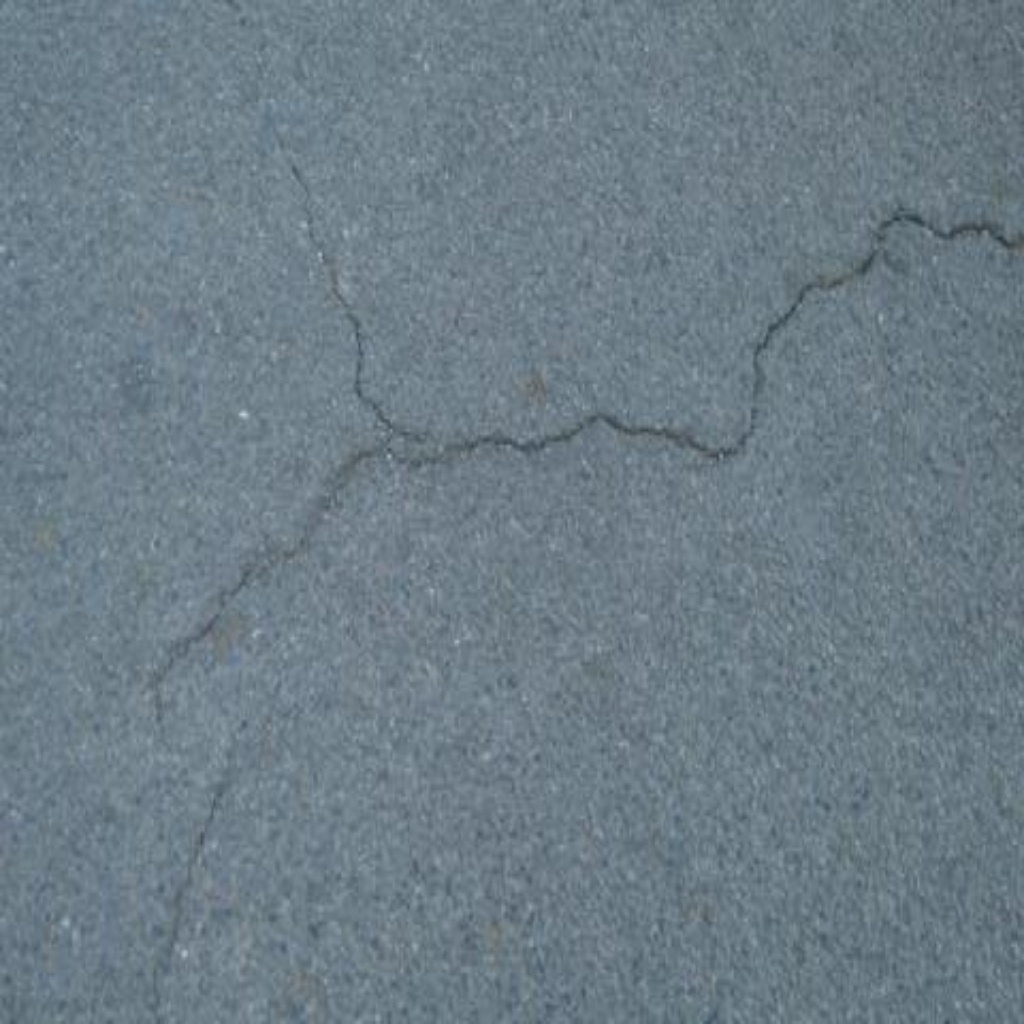

Supplement: S2 File — (ZIP) [file pone.0330218.s002.zip › 1 (1847).jpg]

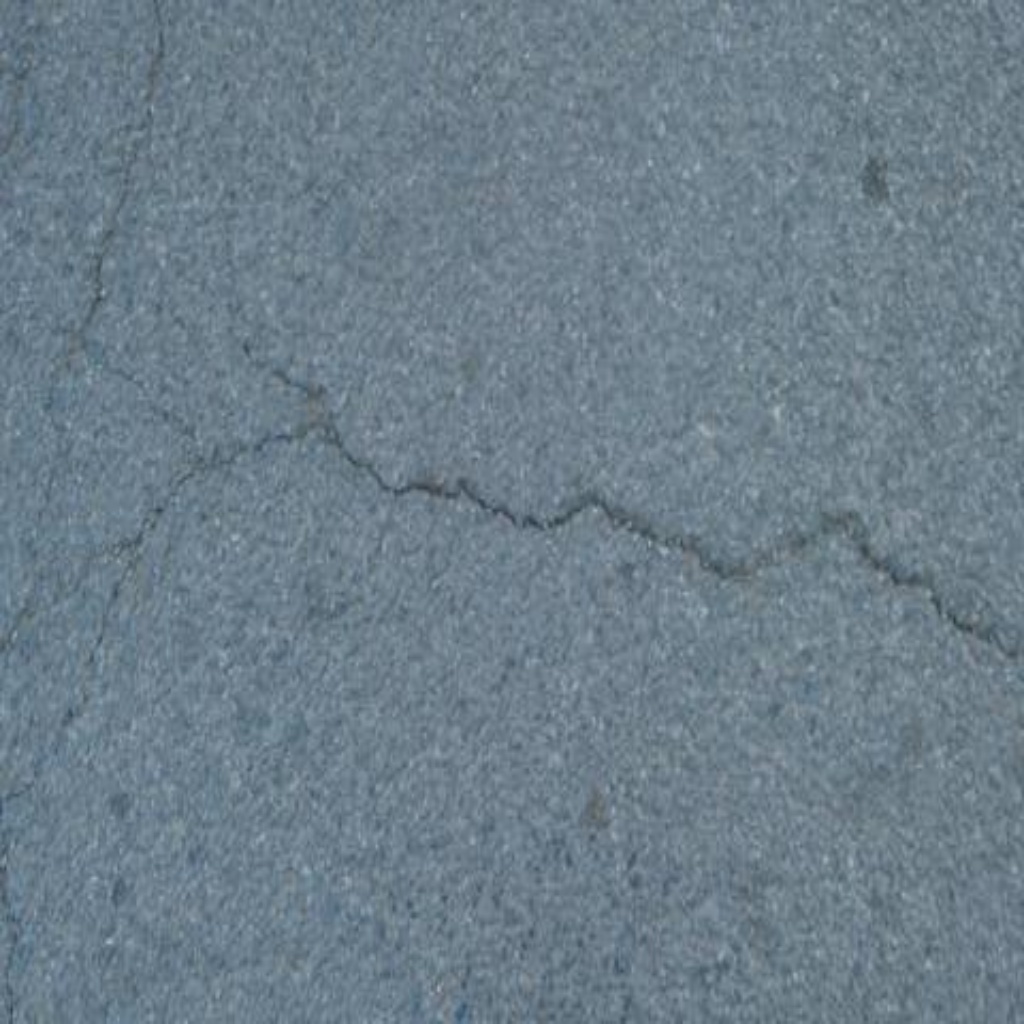

Supplement: S2 File — (ZIP) [file pone.0330218.s002.zip › 1 (1855).jpg]

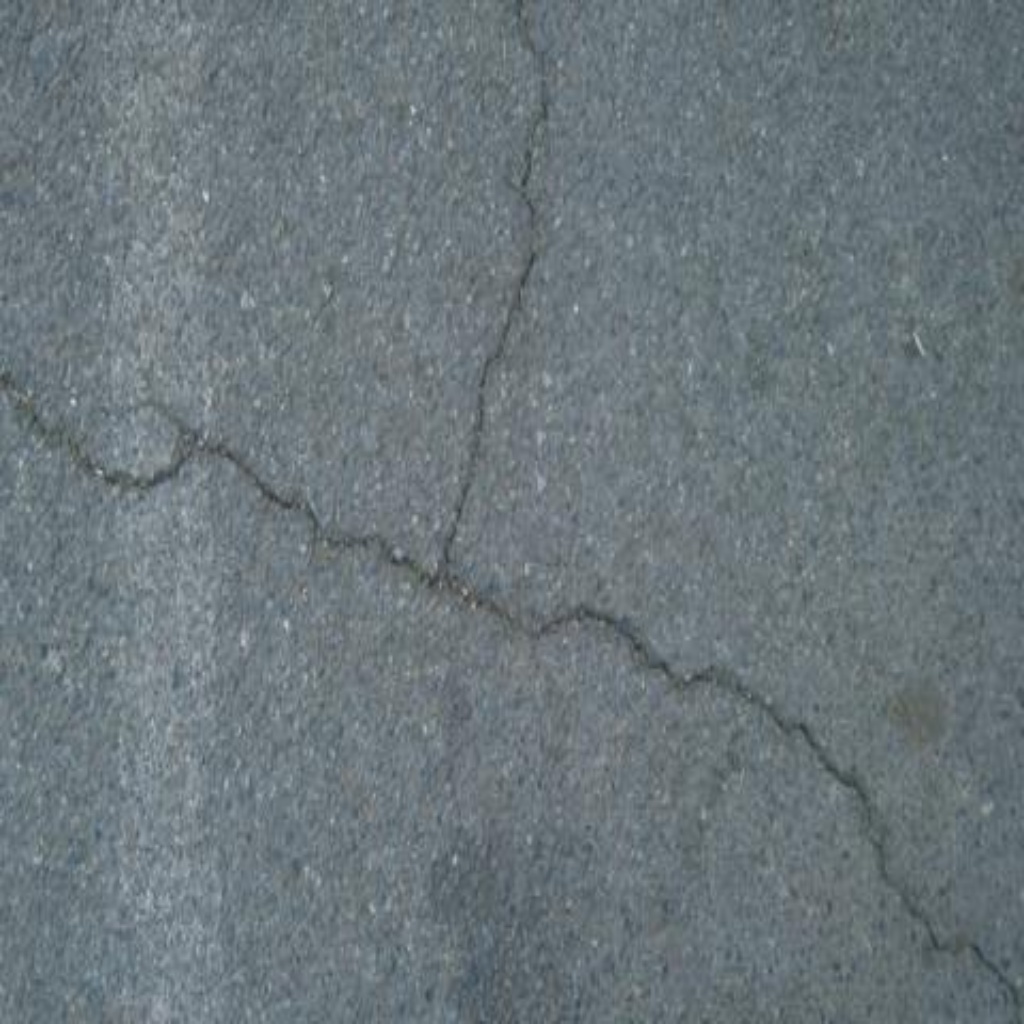

Supplement: S2 File — (ZIP) [file pone.0330218.s002.zip › 1 (1860).jpg]

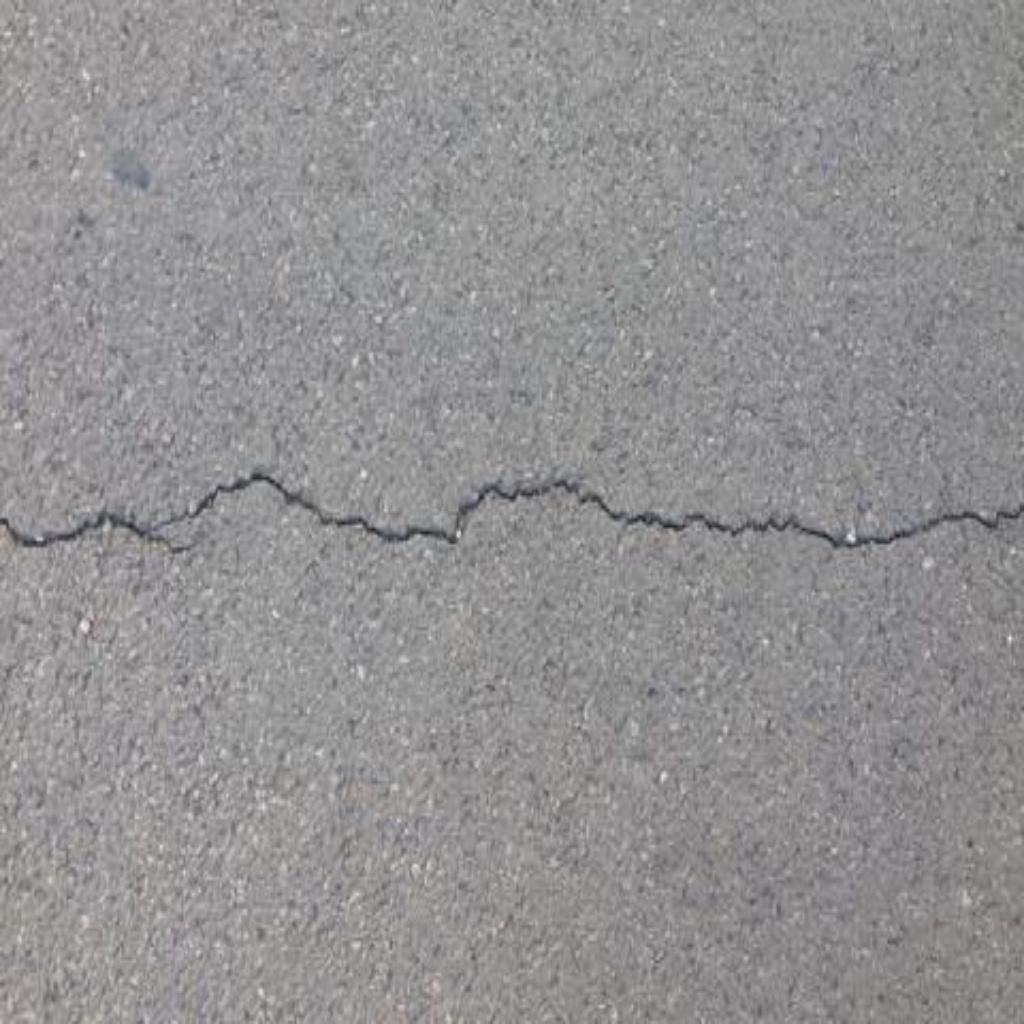

Supplement: S2 File — (ZIP) [file pone.0330218.s002.zip › 1 (1878).jpg]

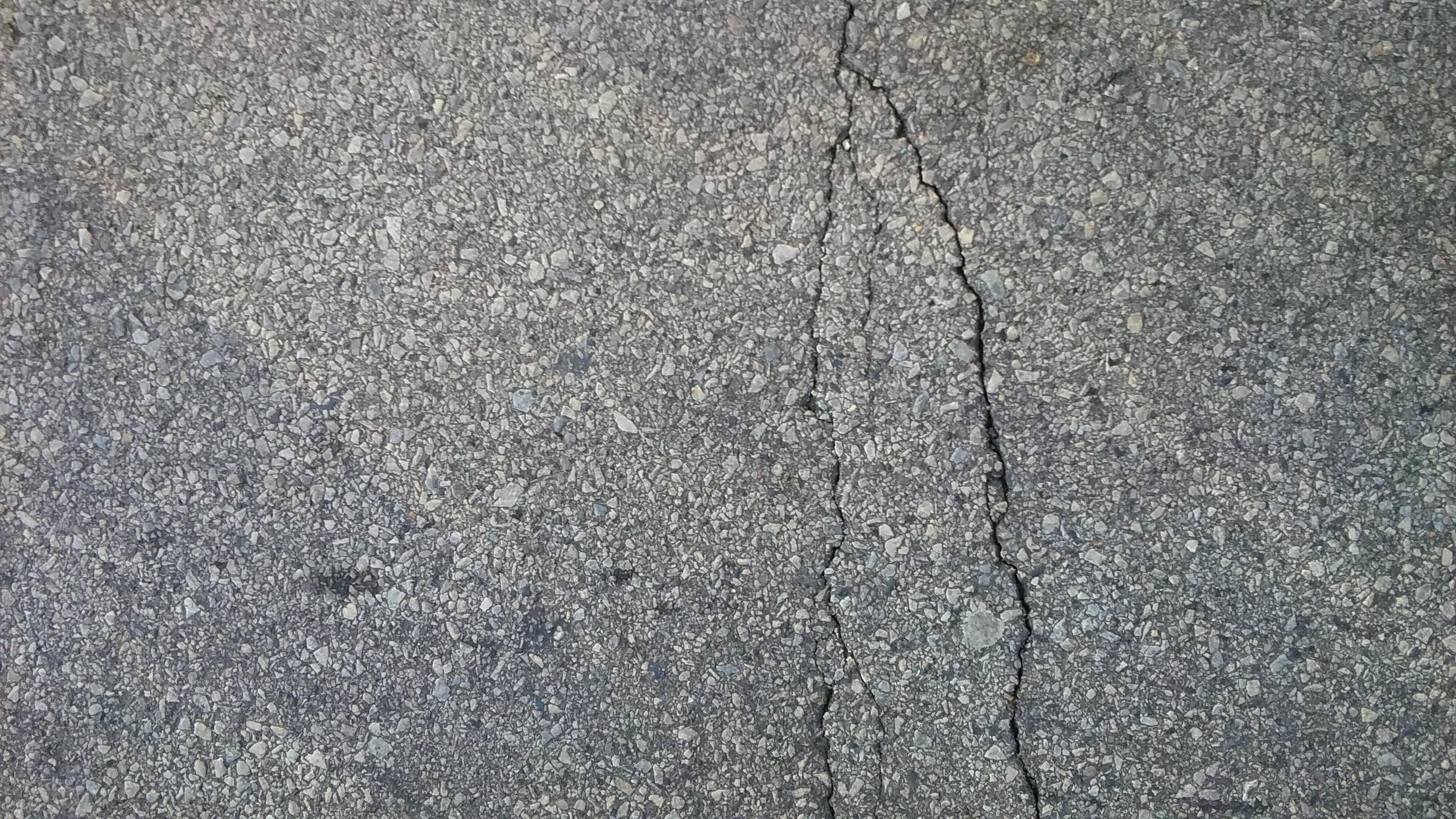

Supplement: S2 File — (ZIP) [file pone.0330218.s002.zip › 1 (1904).jpg]

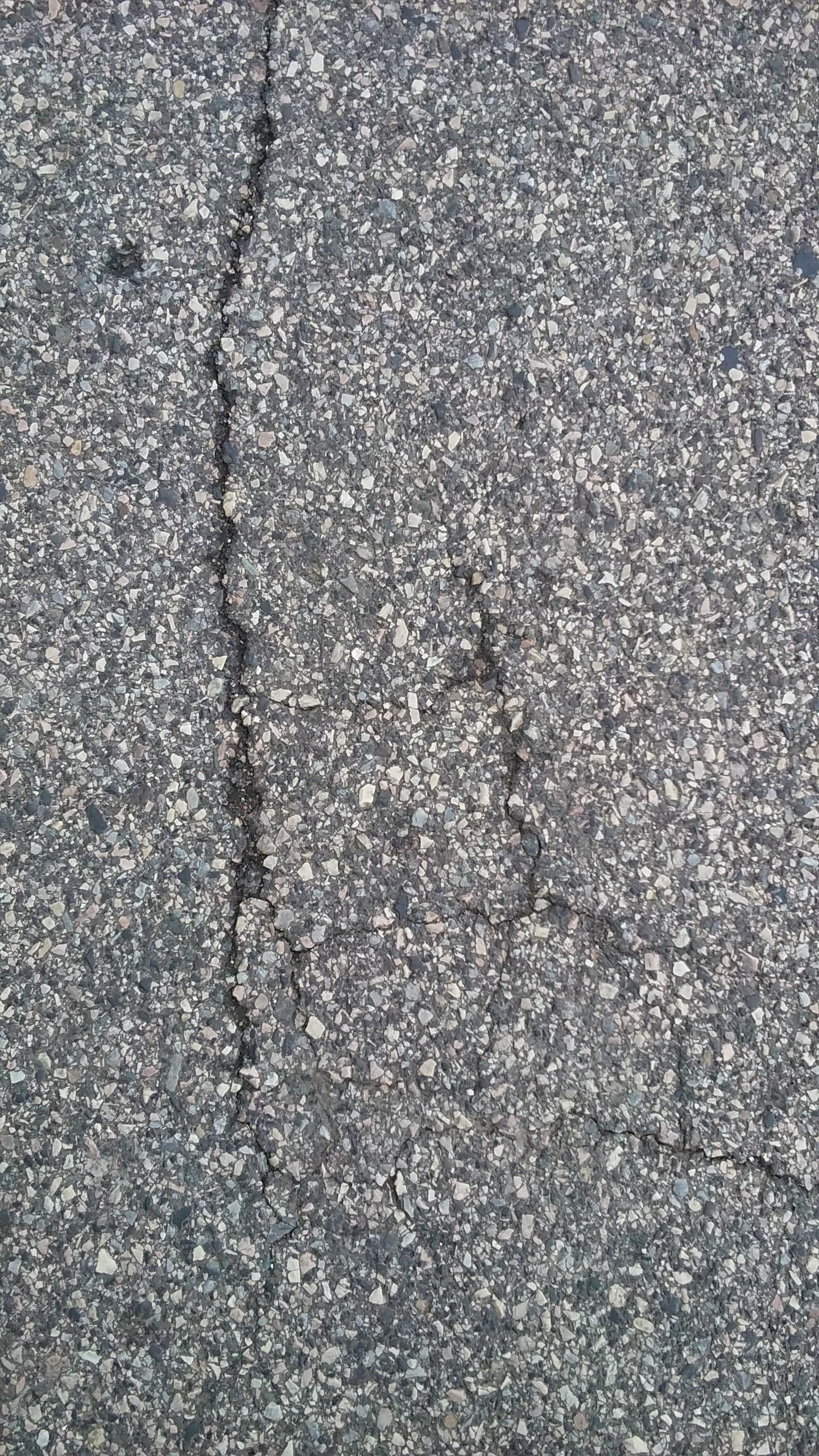

Supplement: S2 File — (ZIP) [file pone.0330218.s002.zip › 1 (1959).jpg]

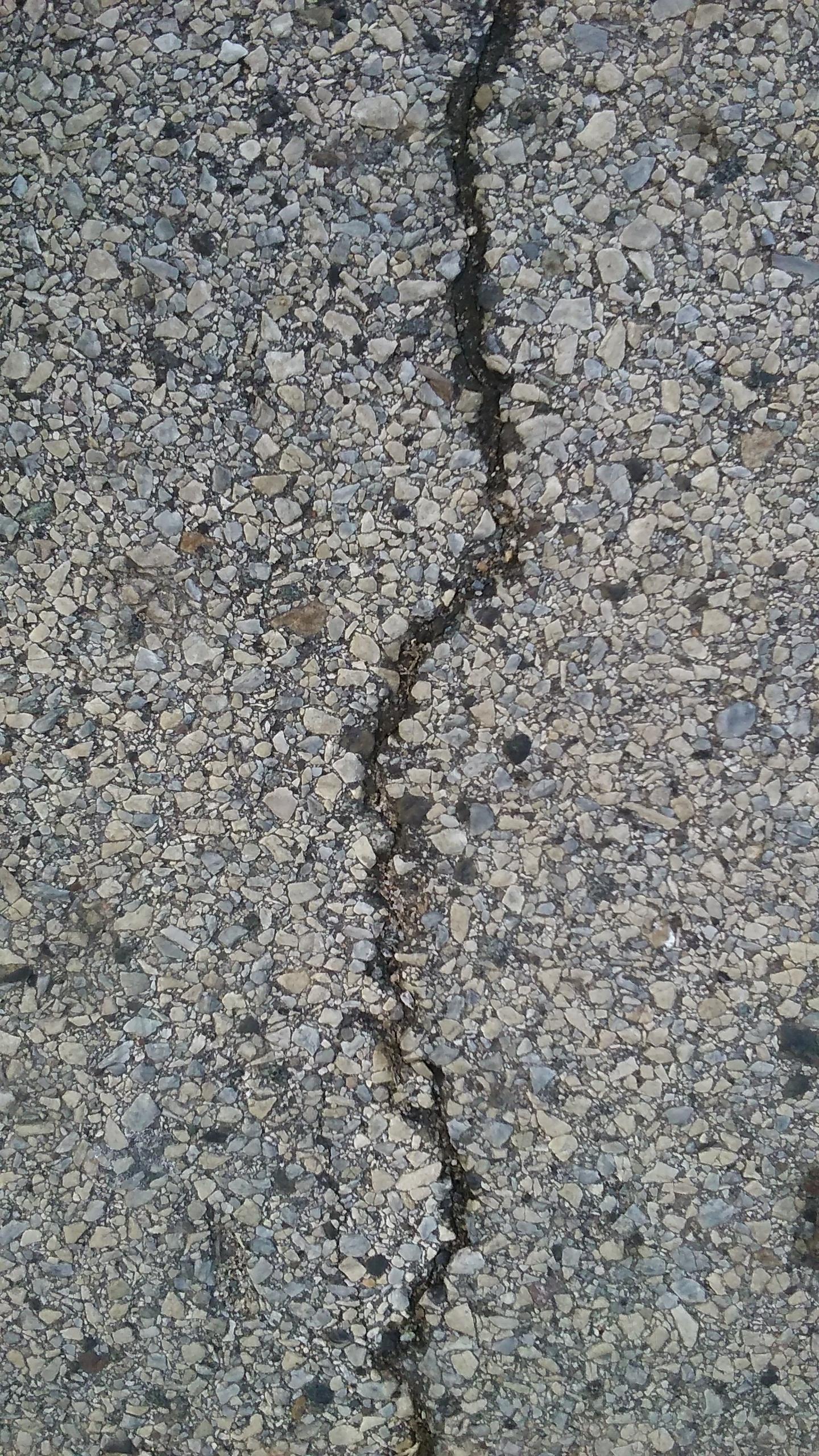

Supplement: S2 File — (ZIP) [file pone.0330218.s002.zip › 1 (1998).jpg]

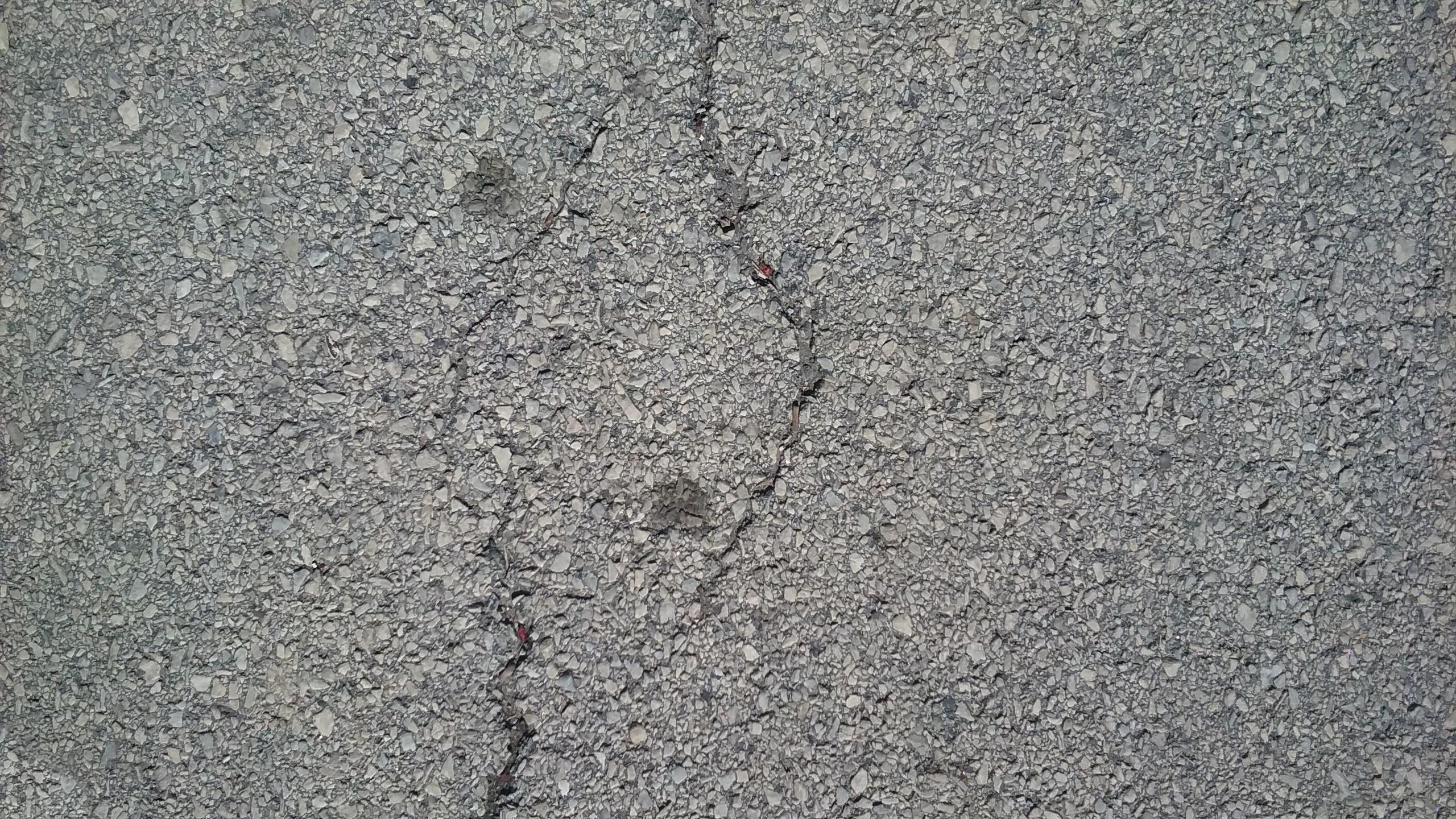

Supplement: S2 File — (ZIP) [file pone.0330218.s002.zip › 1 (2040).jpg]

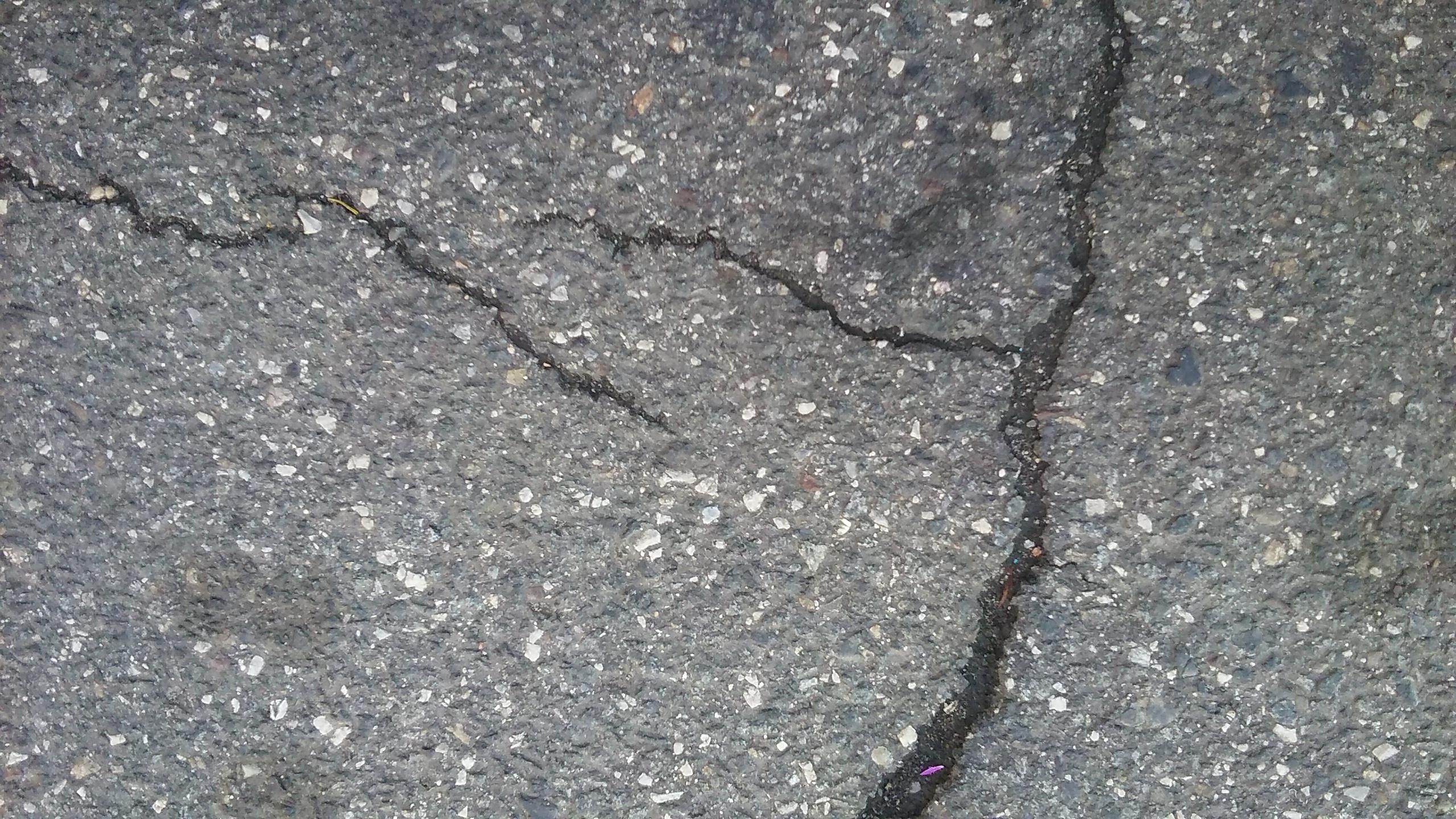

Supplement: S2 File — (ZIP) [file pone.0330218.s002.zip › 1 (2052).jpg]

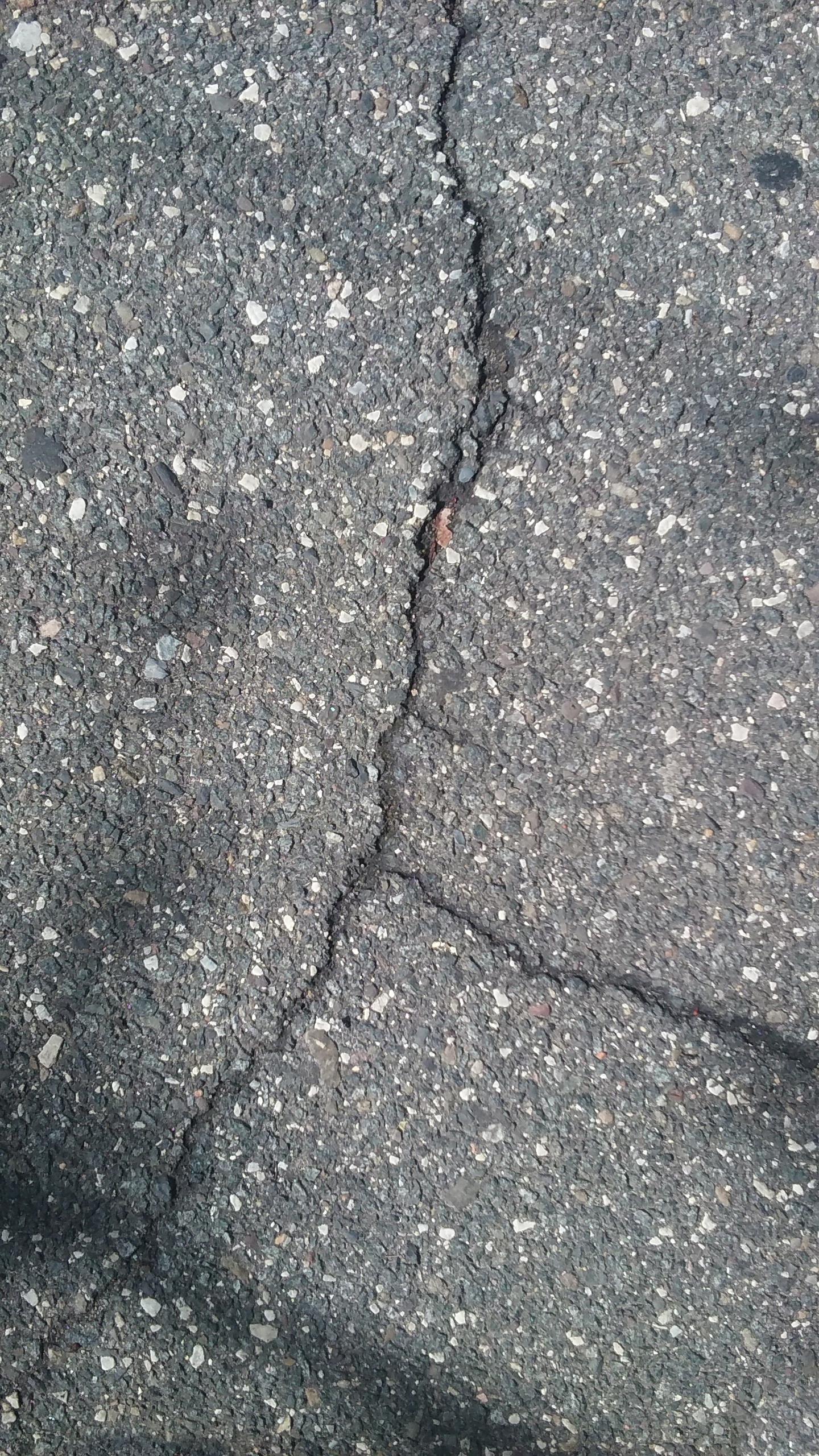

Supplement: S2 File — (ZIP) [file pone.0330218.s002.zip › 1 (2057).jpg]

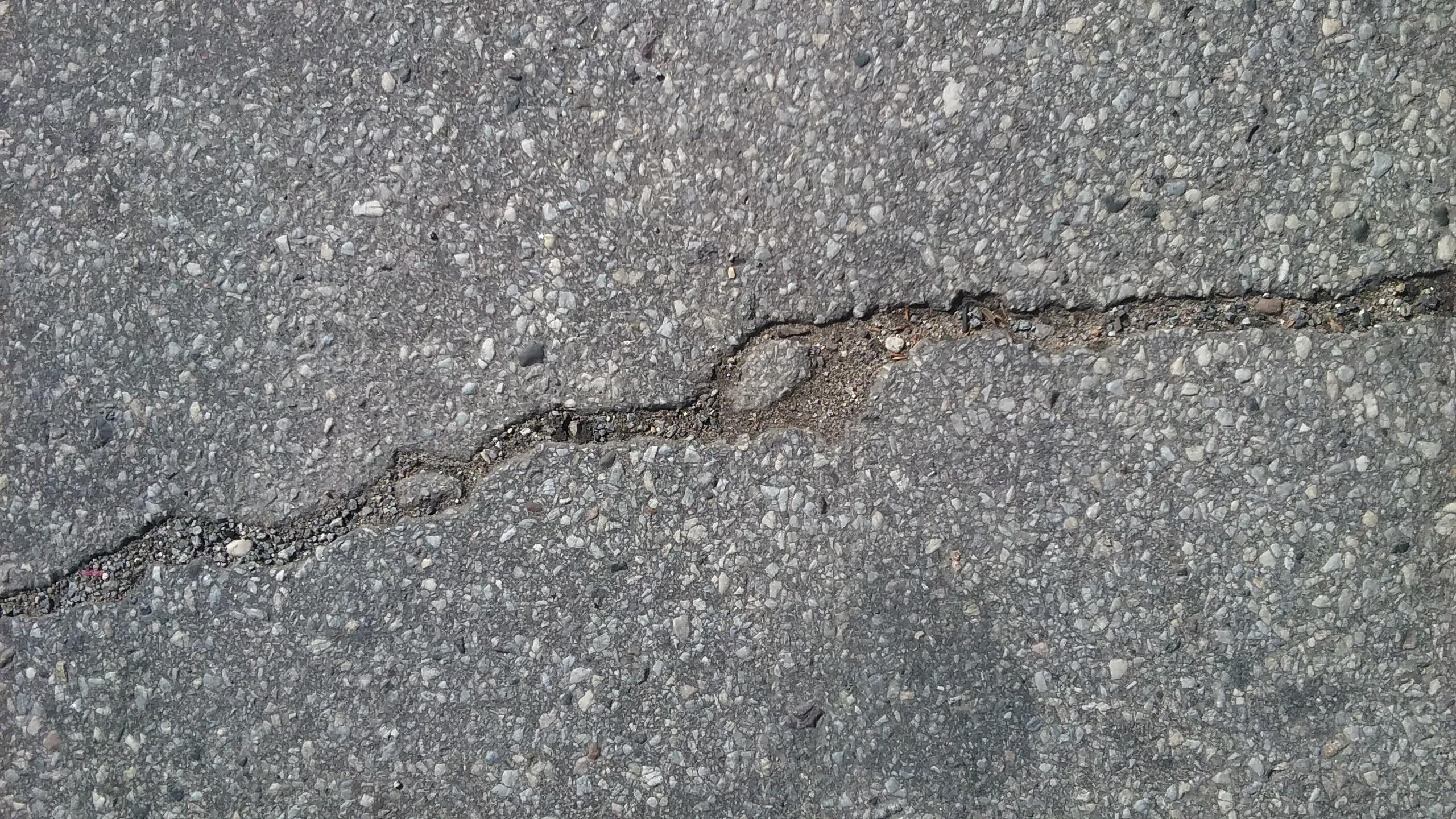

Supplement: S2 File — (ZIP) [file pone.0330218.s002.zip › 1 (2087).jpg]

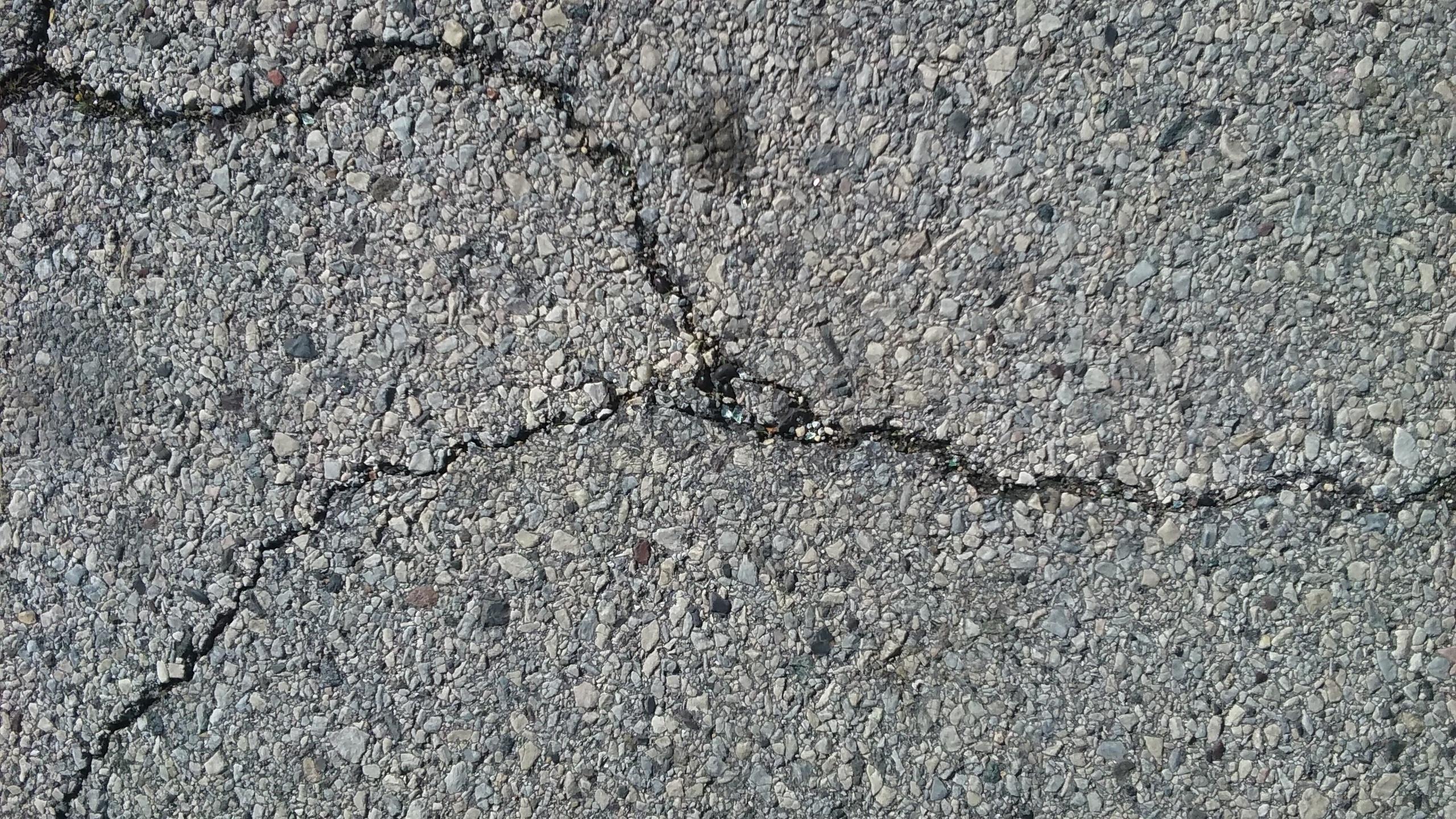

Supplement: S2 File — (ZIP) [file pone.0330218.s002.zip › 1 (2109).jpg]

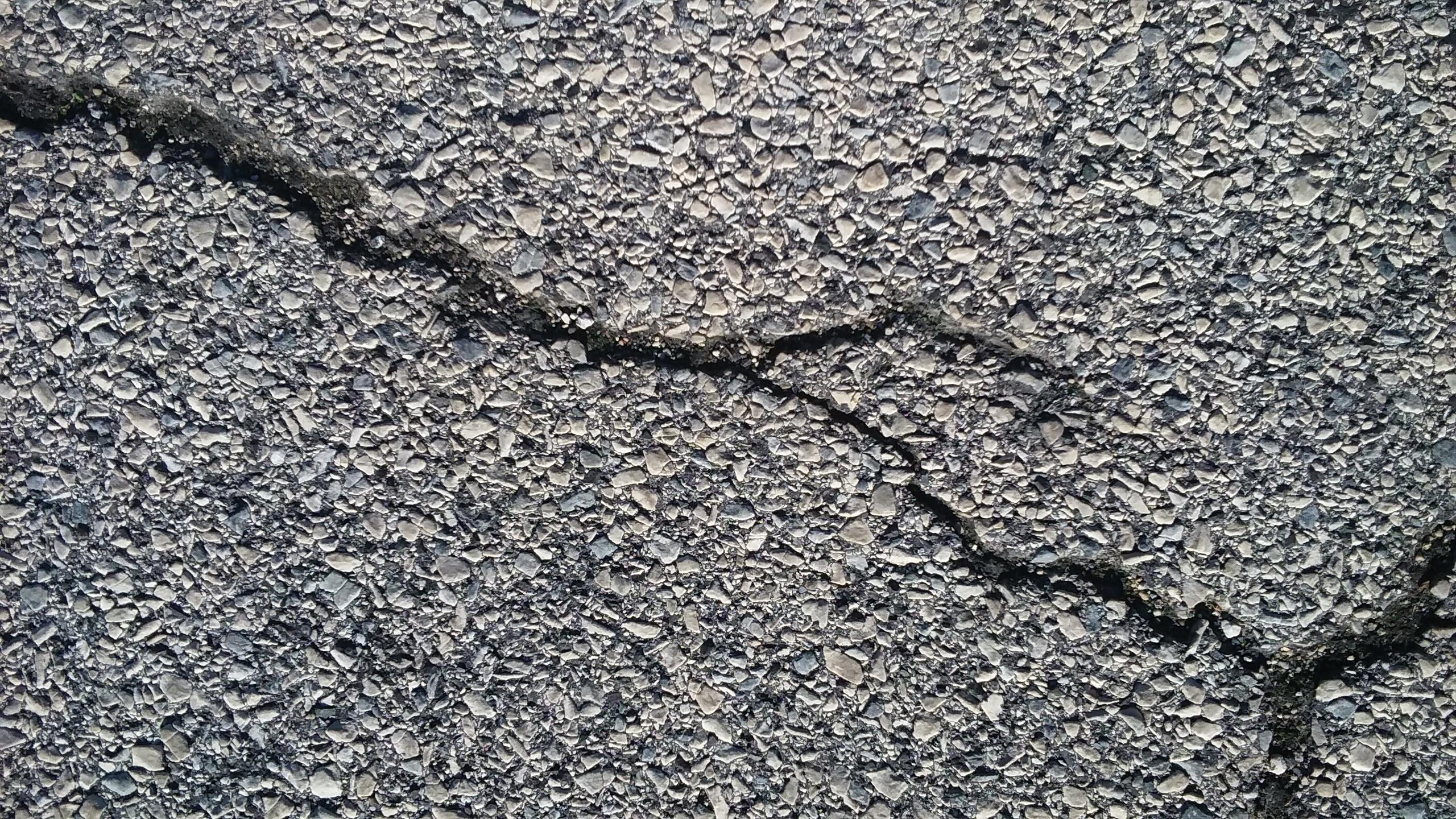

Supplement: S2 File — (ZIP) [file pone.0330218.s002.zip › 1 (2178).jpg]

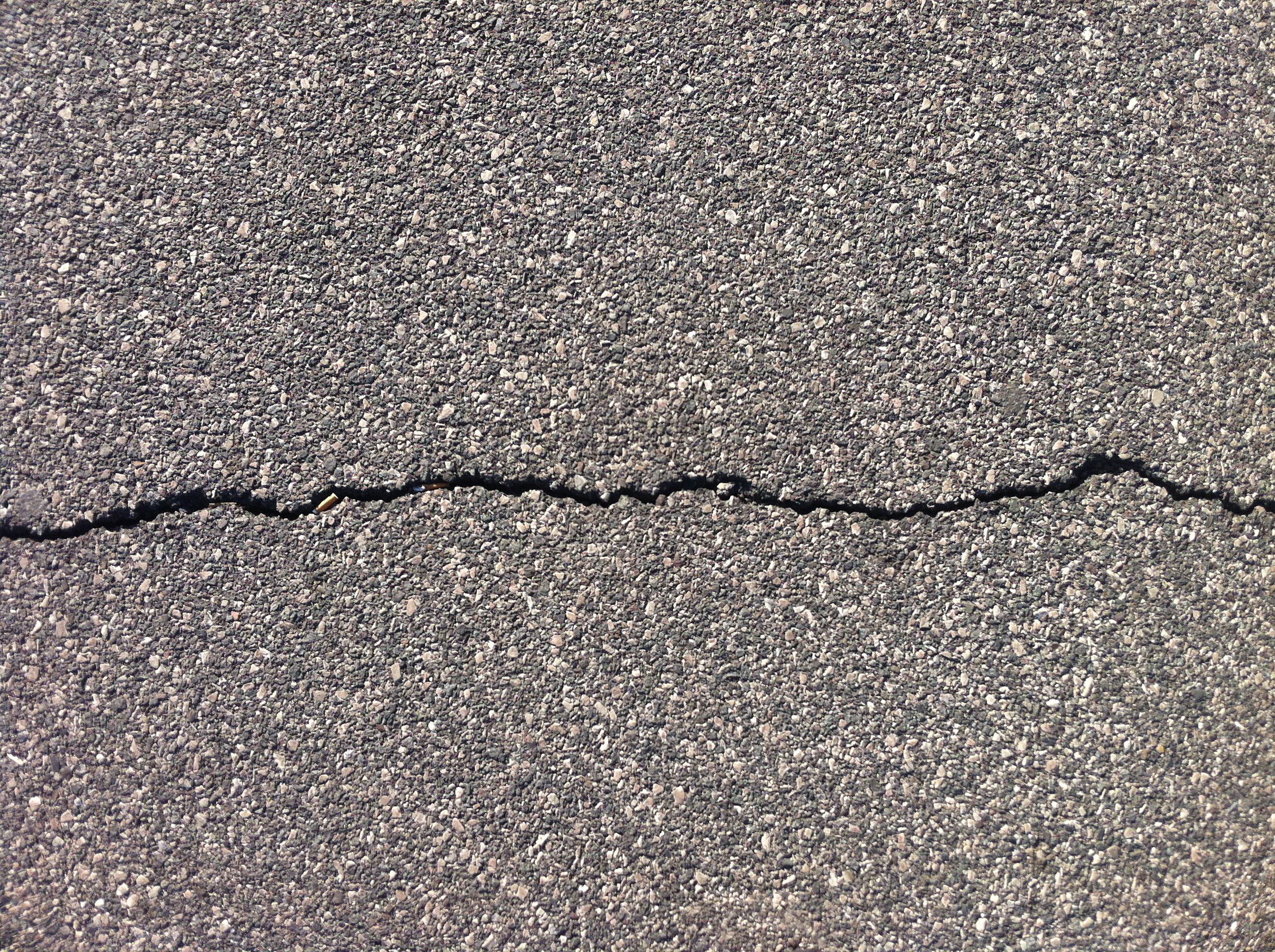

Supplement: S2 File — (ZIP) [file pone.0330218.s002.zip › 1 (2194).jpg]

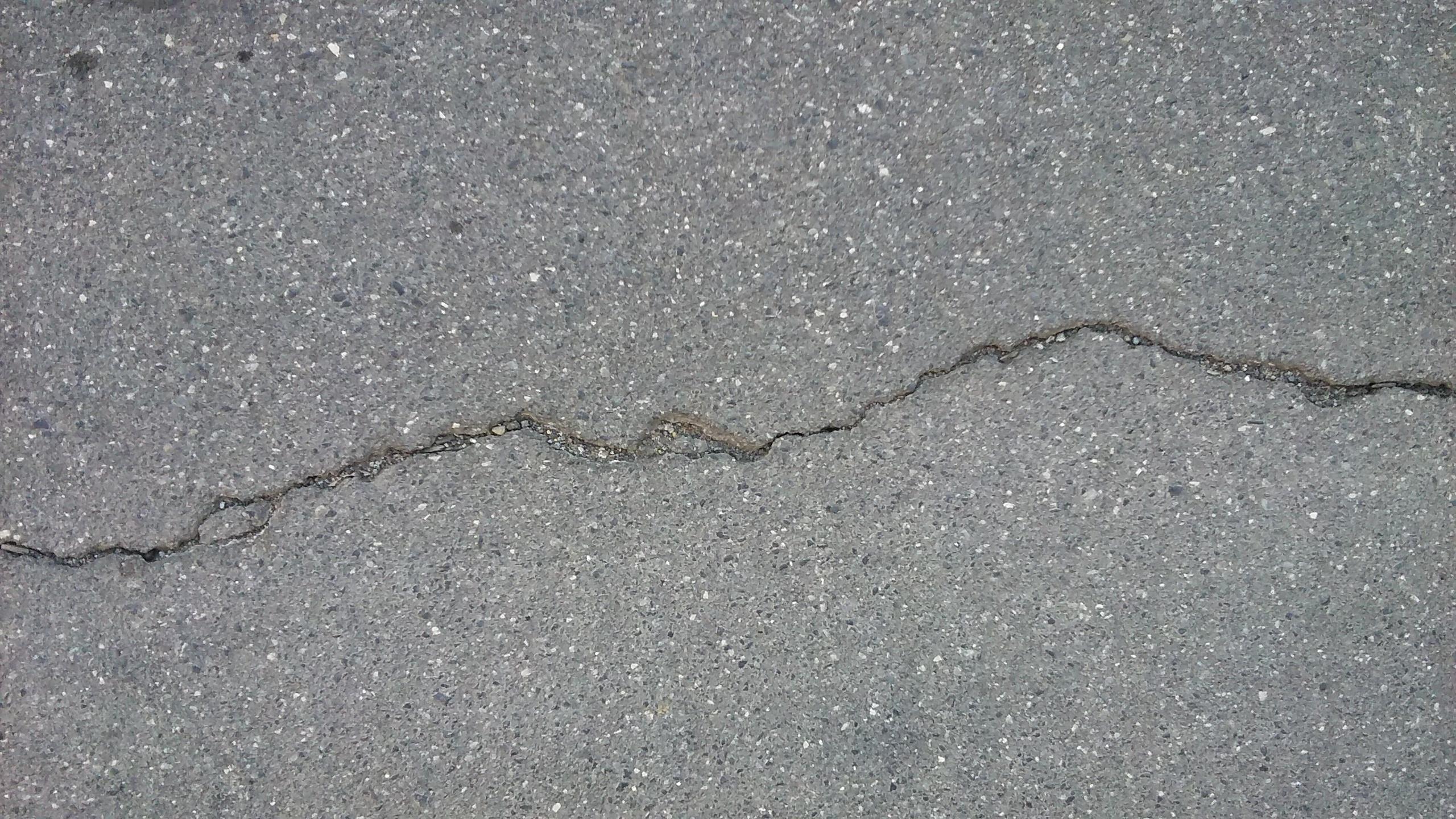

Supplement: S2 File — (ZIP) [file pone.0330218.s002.zip › 1 (2230).jpg]

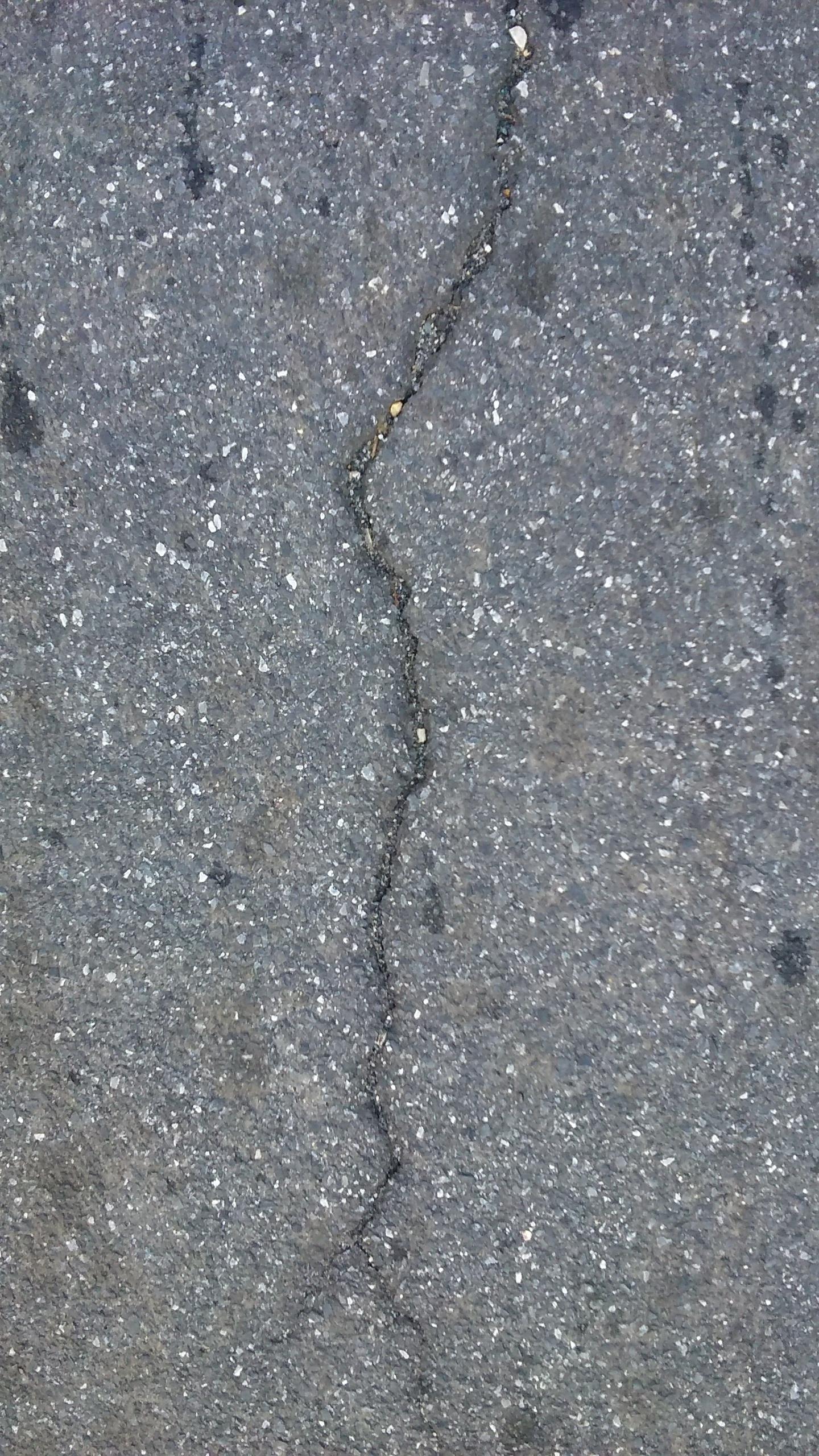

Supplement: S2 File — (ZIP) [file pone.0330218.s002.zip › 1 (2232).jpg]

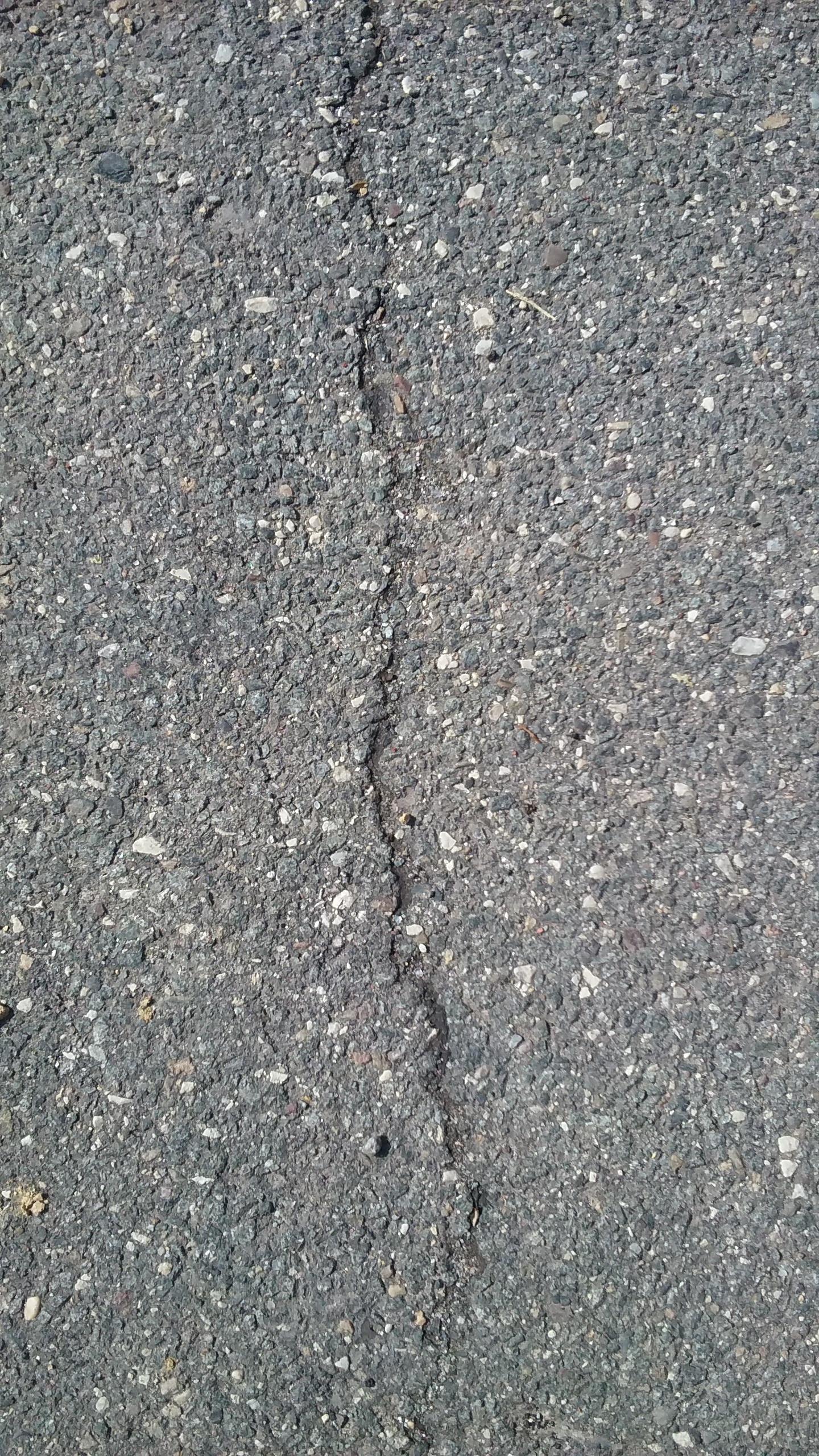

Supplement: S2 File — (ZIP) [file pone.0330218.s002.zip › 1 (2245).jpg]

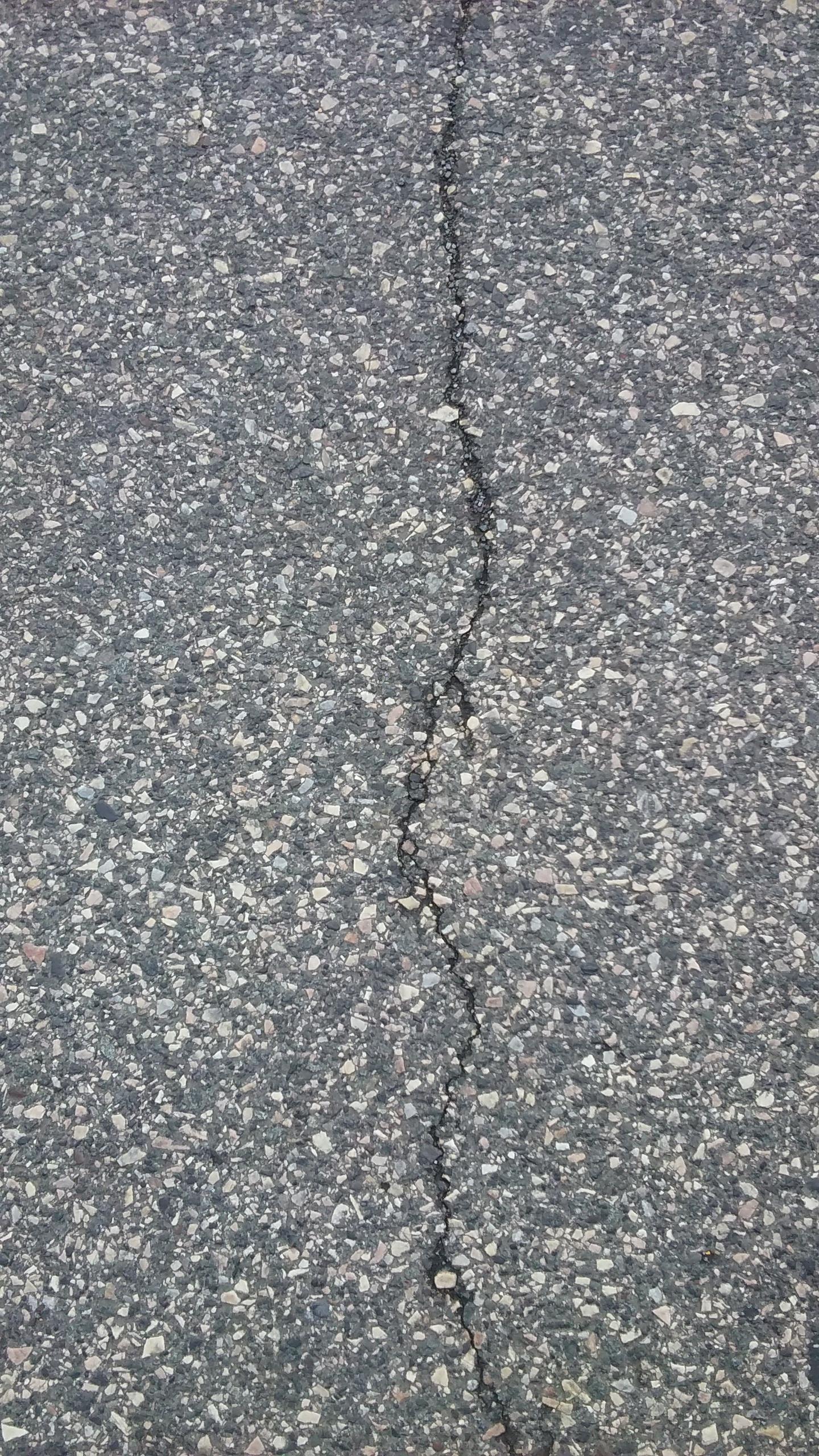

Supplement: S2 File — (ZIP) [file pone.0330218.s002.zip › 1 (2269).jpg]

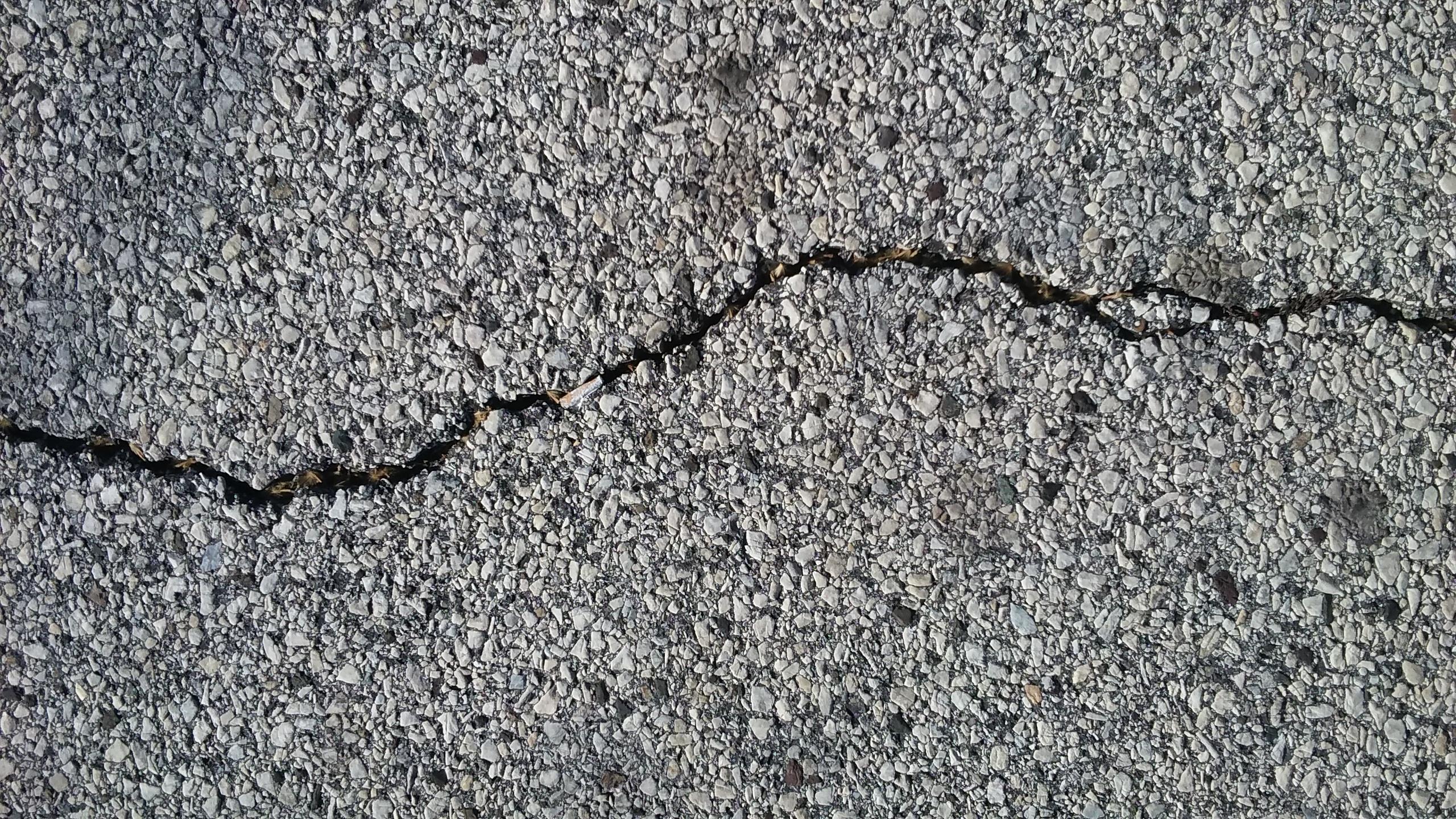

Supplement: S2 File — (ZIP) [file pone.0330218.s002.zip › 1 (2285).jpg]

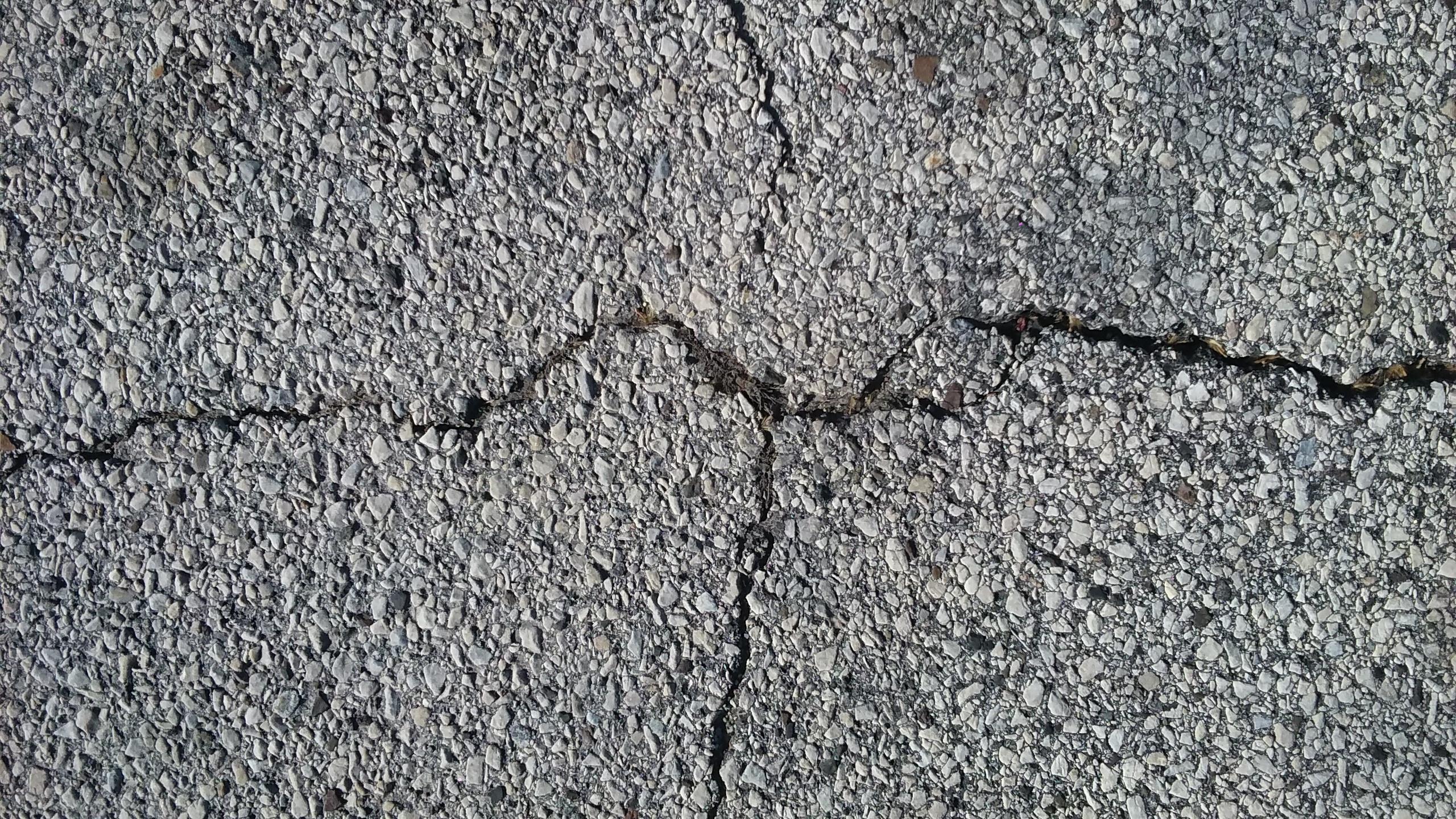

Supplement: S2 File — (ZIP) [file pone.0330218.s002.zip › 1 (2286).jpg]

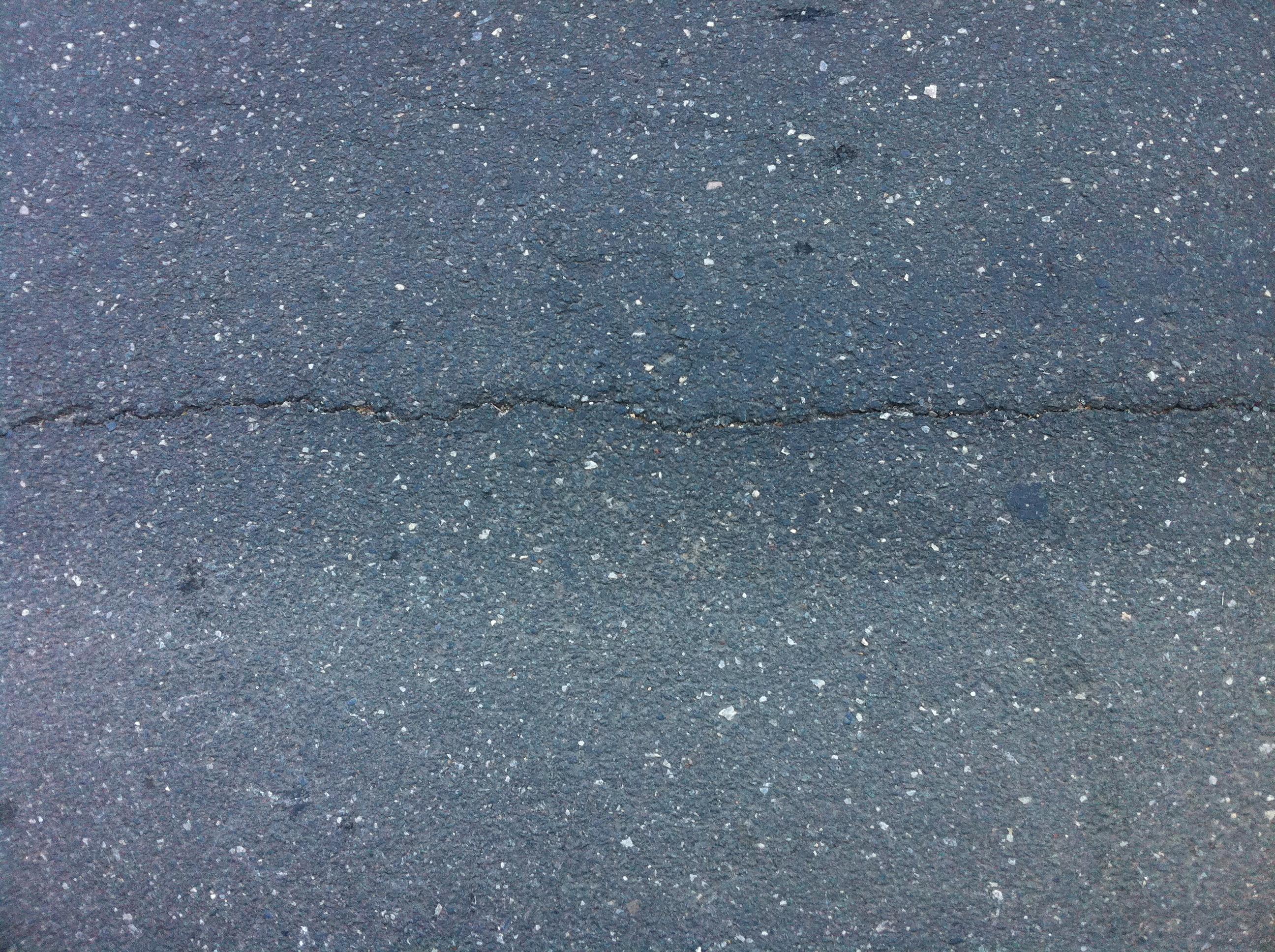

Supplement: S2 File — (ZIP) [file pone.0330218.s002.zip › 1 (2300).jpg]

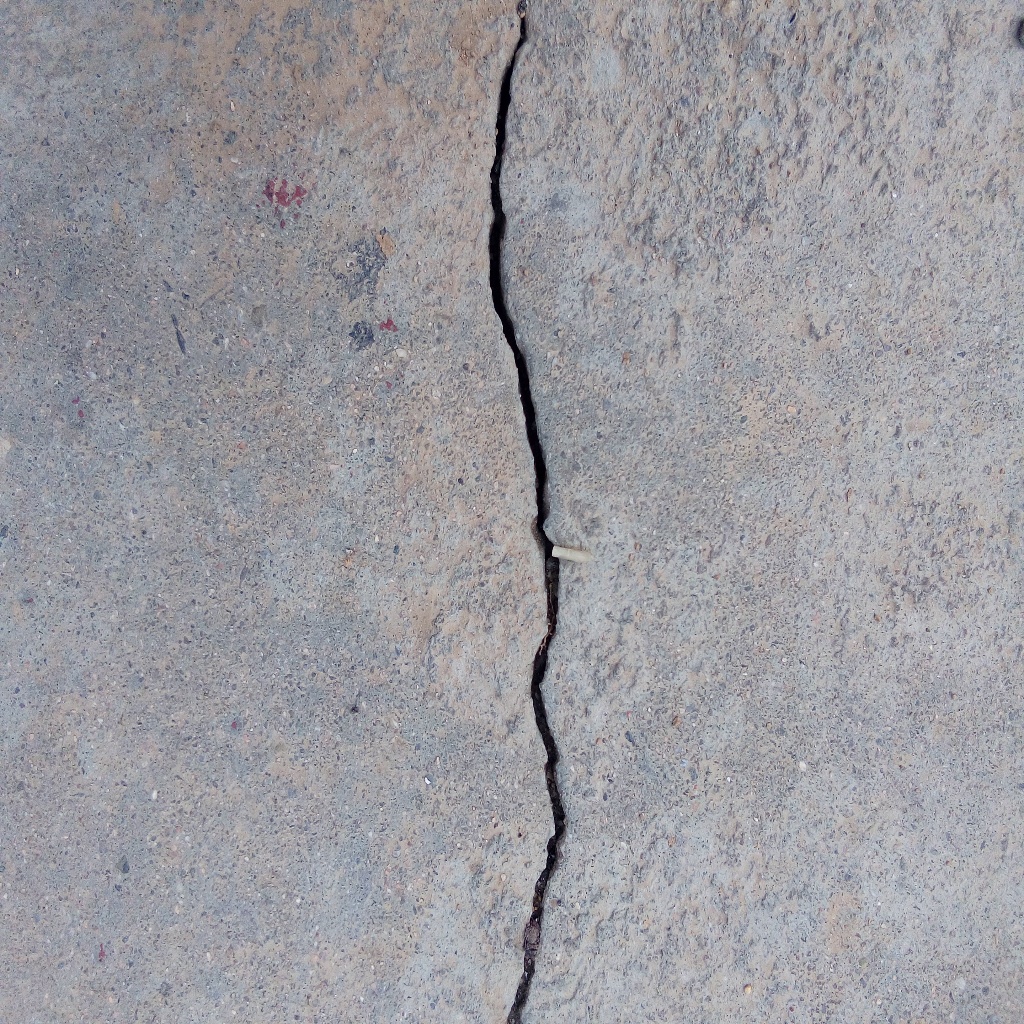

Supplement: S2 File — (ZIP) [file pone.0330218.s002.zip › 1 (232).jpg]

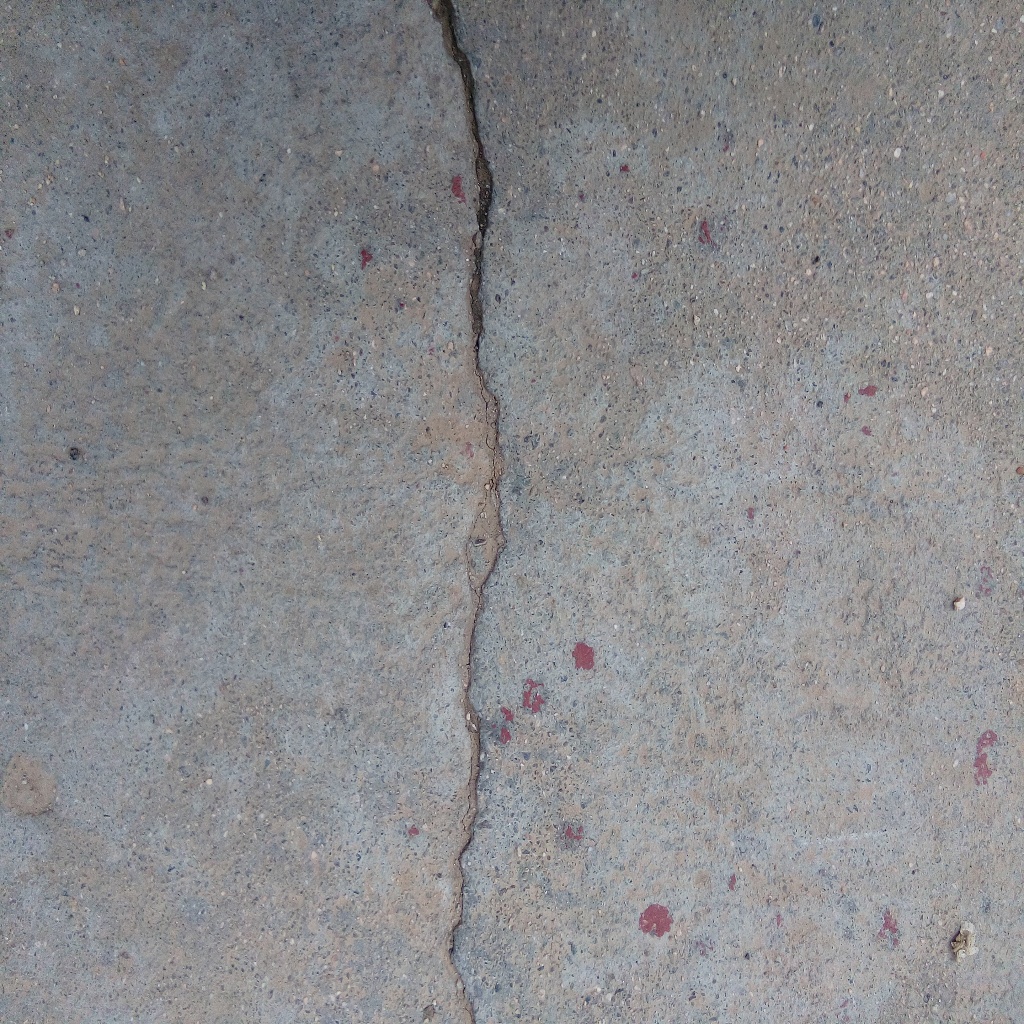

Supplement: S2 File — (ZIP) [file pone.0330218.s002.zip › 1 (234).jpg]

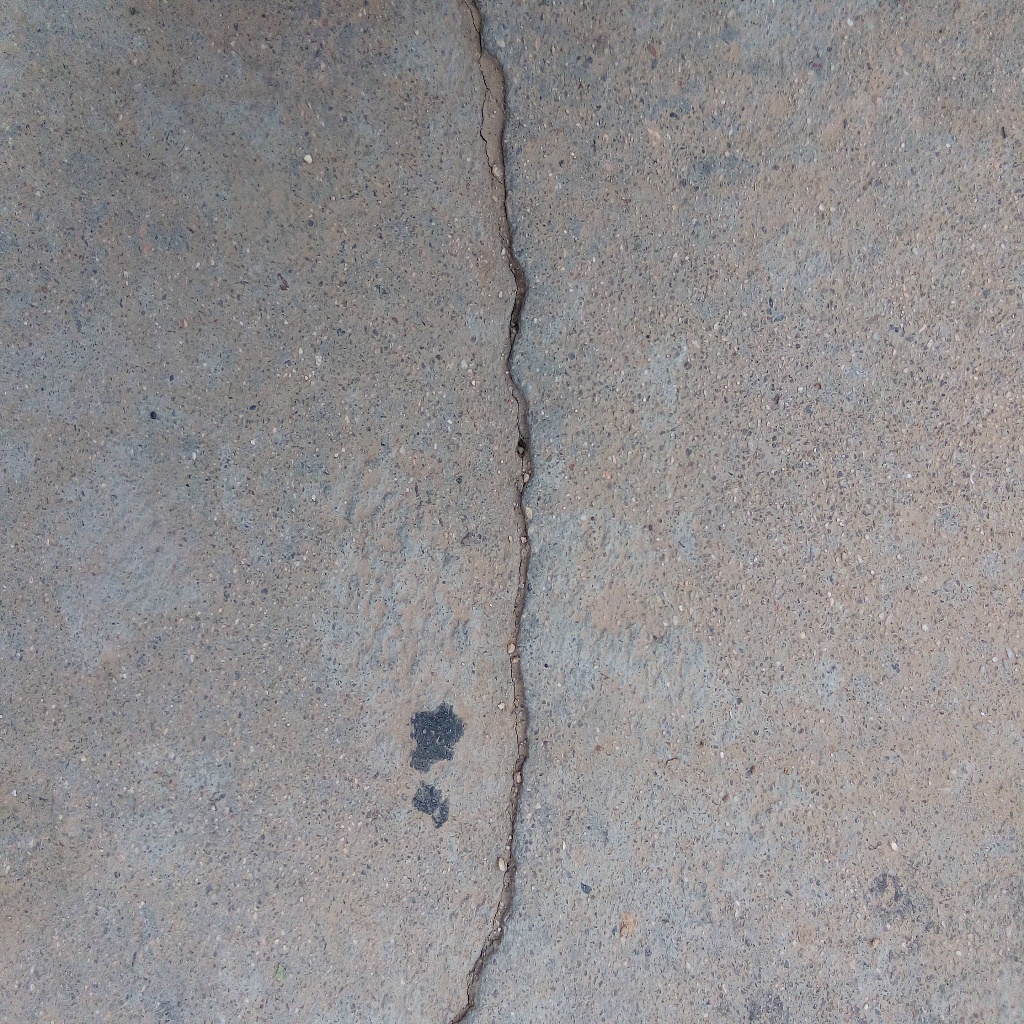

Supplement: S2 File — (ZIP) [file pone.0330218.s002.zip › 1 (235).jpg]

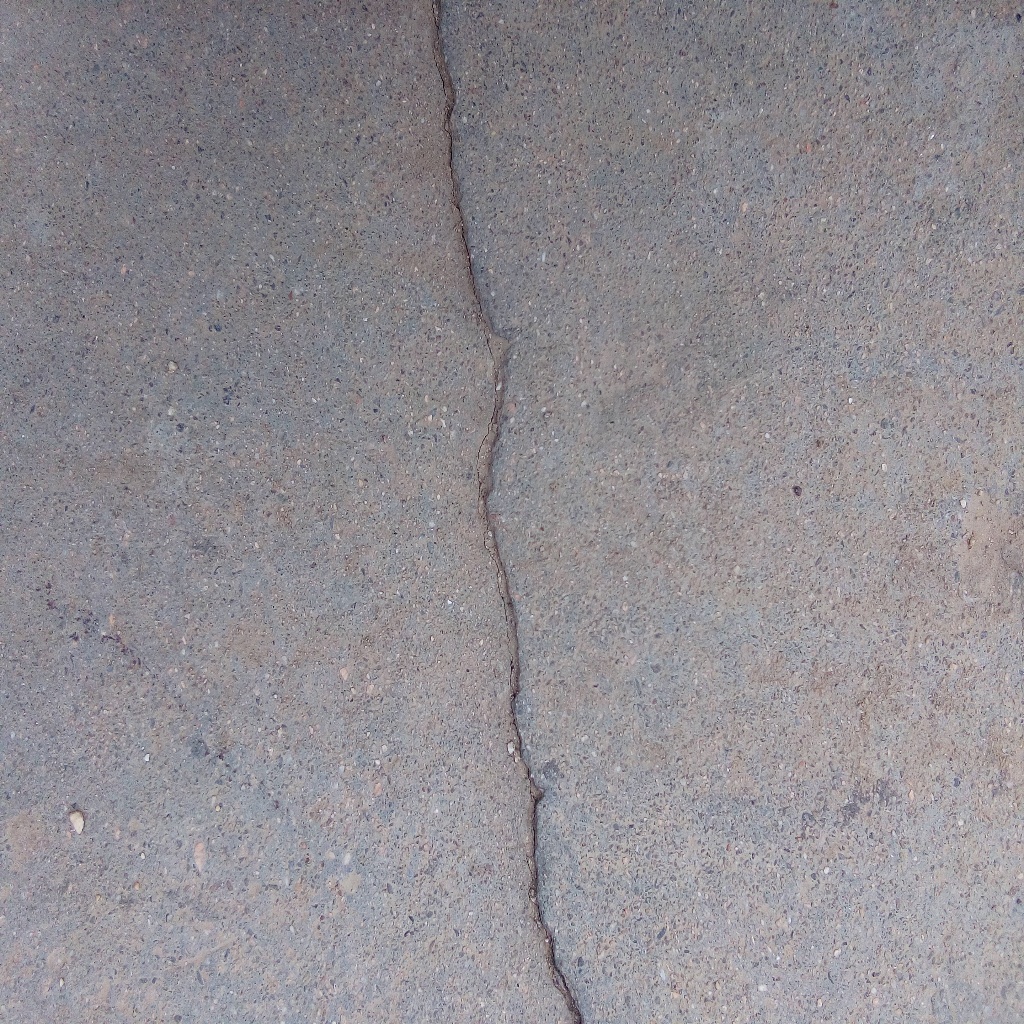

Supplement: S2 File — (ZIP) [file pone.0330218.s002.zip › 1 (236).jpg]

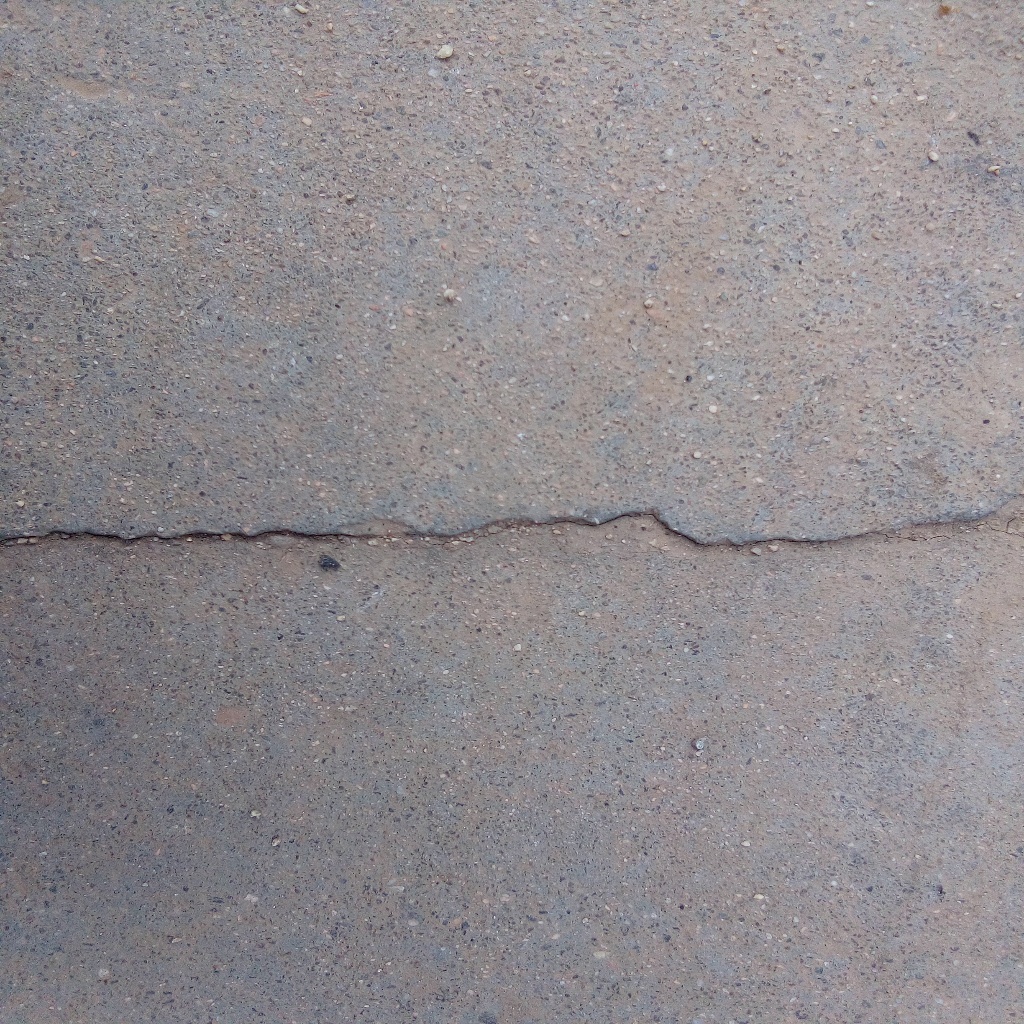

Supplement: S2 File — (ZIP) [file pone.0330218.s002.zip › 1 (237).jpg]

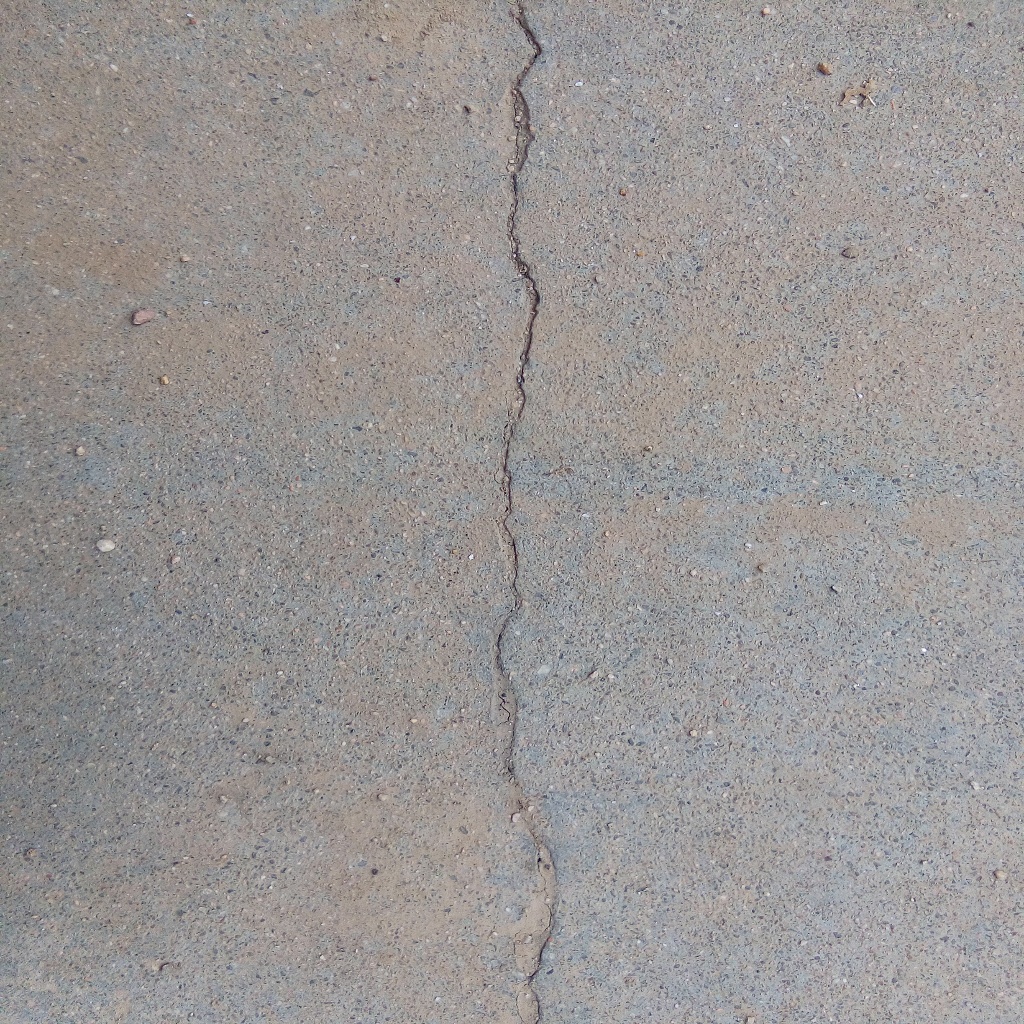

Supplement: S2 File — (ZIP) [file pone.0330218.s002.zip › 1 (240).jpg]

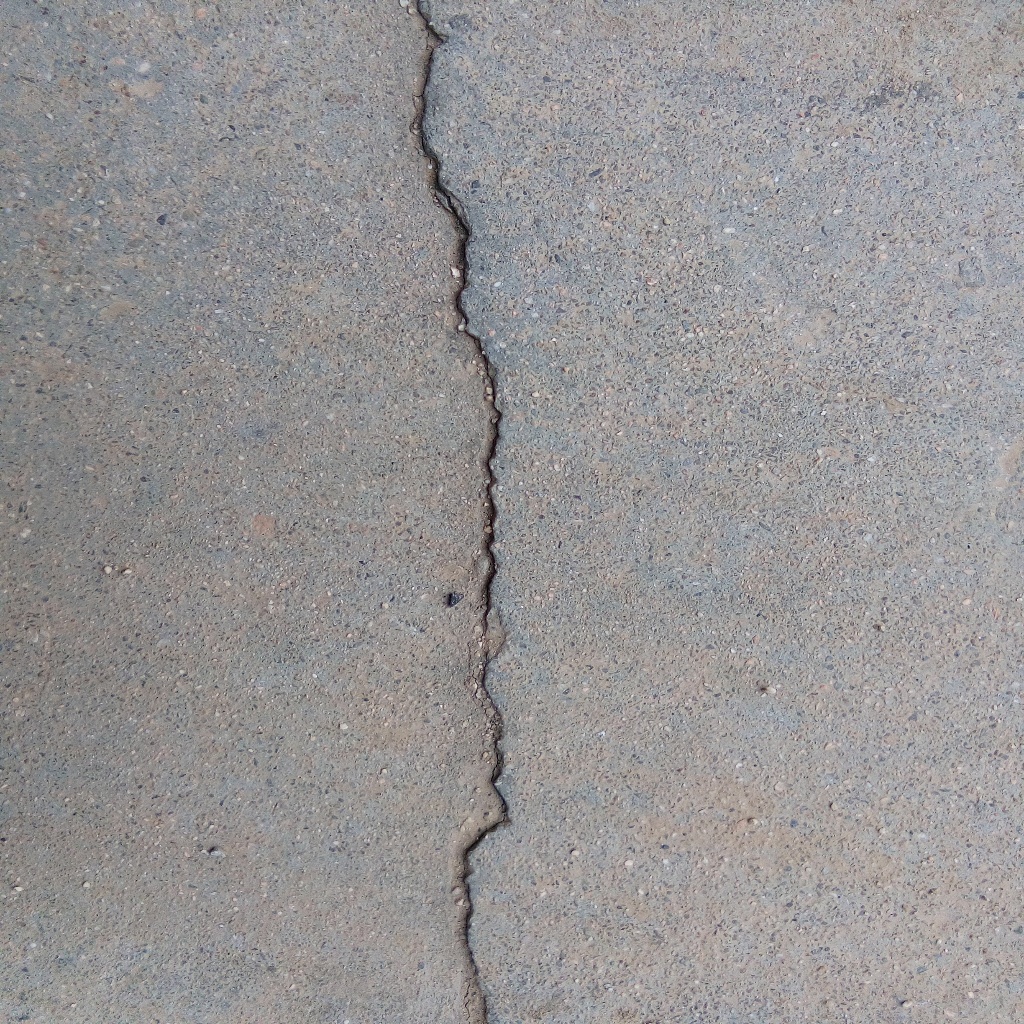

Supplement: S2 File — (ZIP) [file pone.0330218.s002.zip › 1 (241).jpg]

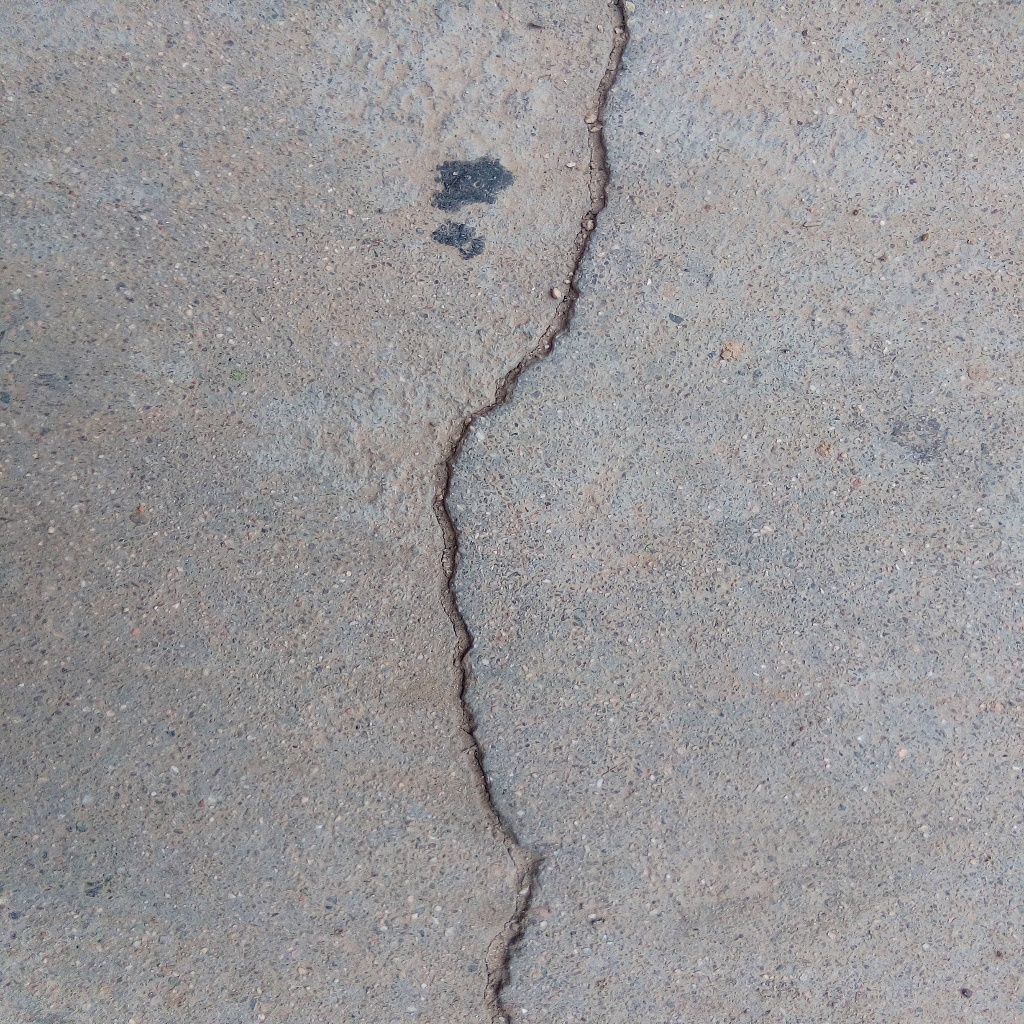

Supplement: S2 File — (ZIP) [file pone.0330218.s002.zip › 1 (242).jpg]

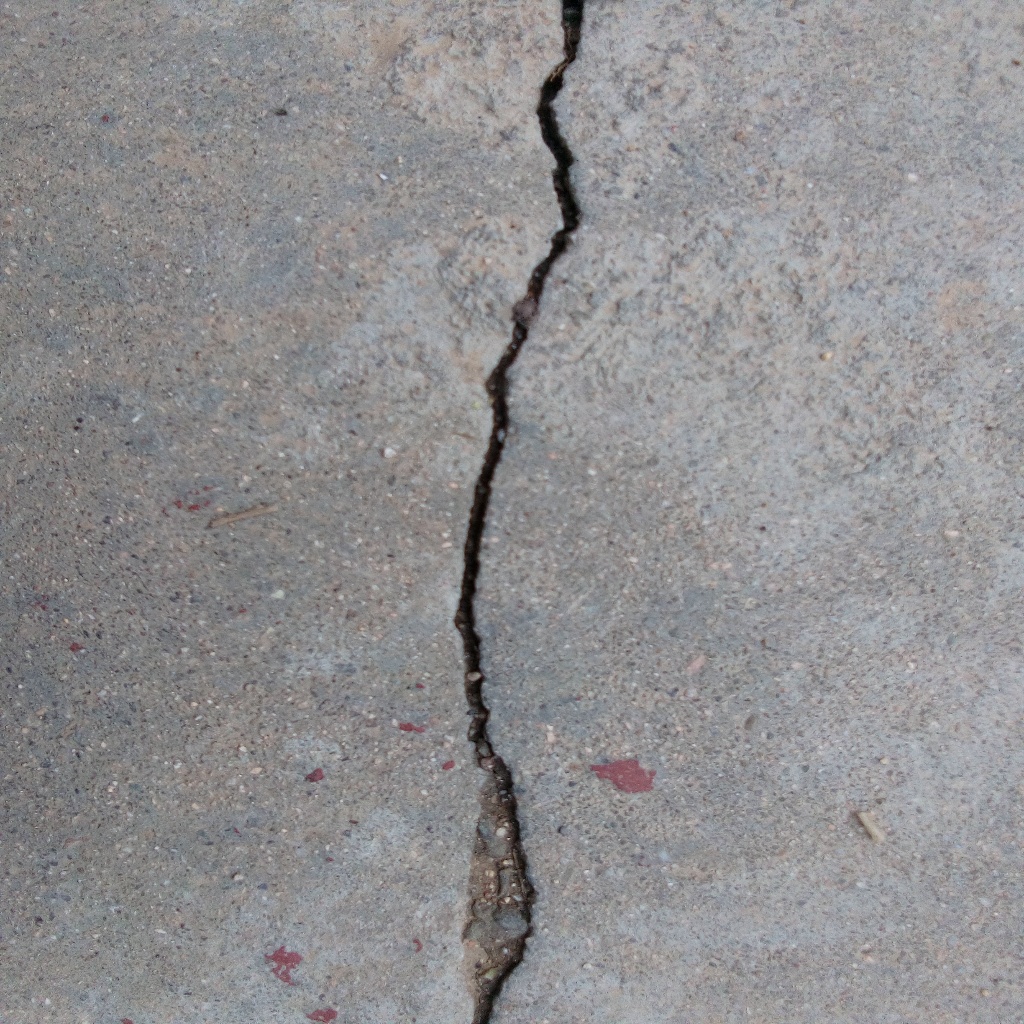

Supplement: S2 File — (ZIP) [file pone.0330218.s002.zip › 1 (244).jpg]

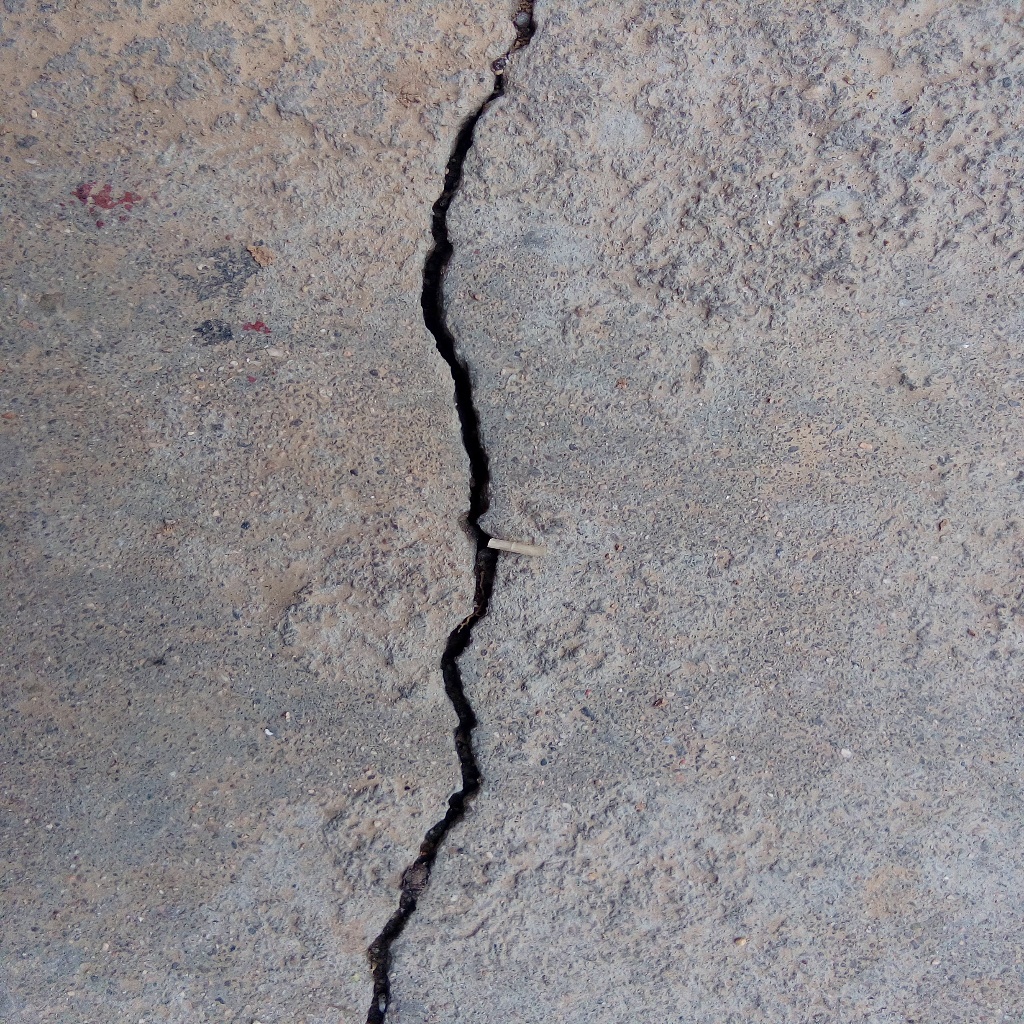

Supplement: S2 File — (ZIP) [file pone.0330218.s002.zip › 1 (245).jpg]

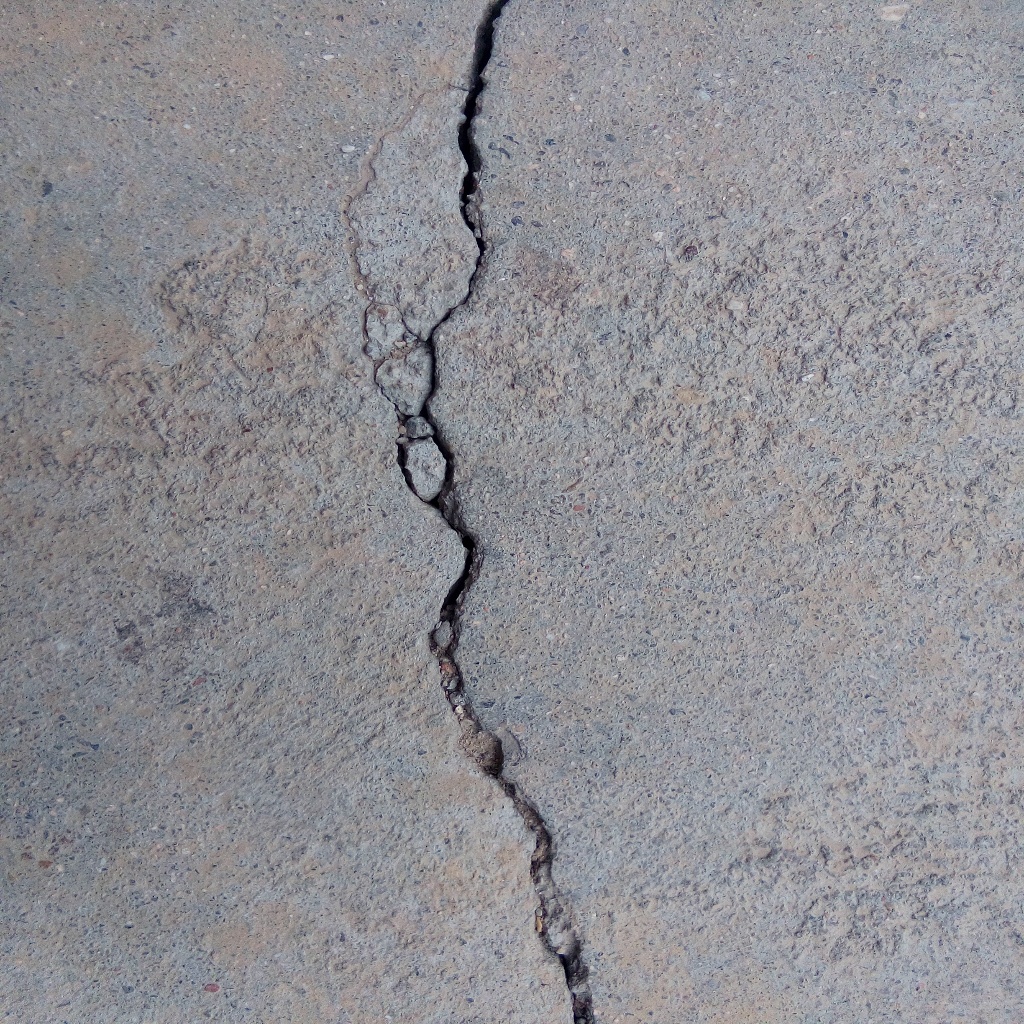

Supplement: S2 File — (ZIP) [file pone.0330218.s002.zip › 1 (246).jpg]

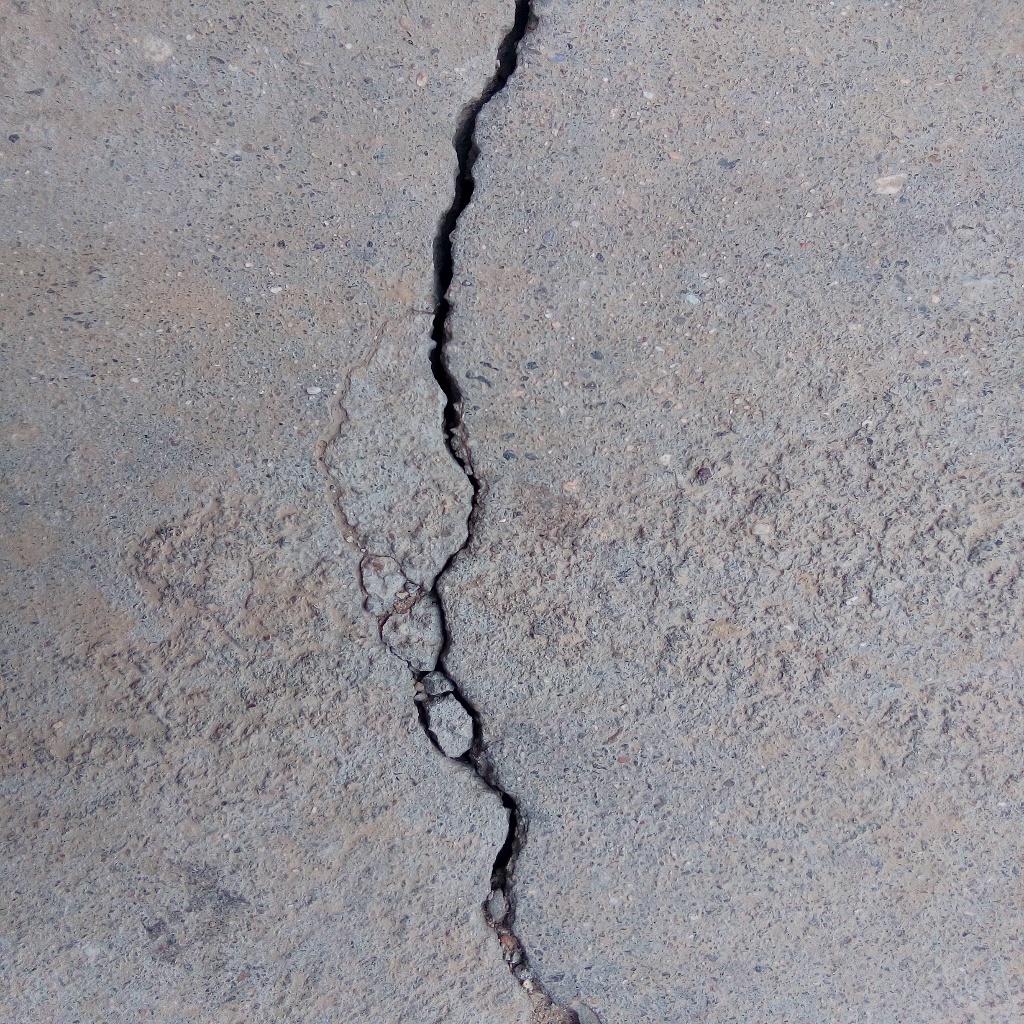

Supplement: S2 File — (ZIP) [file pone.0330218.s002.zip › 1 (247).jpg]

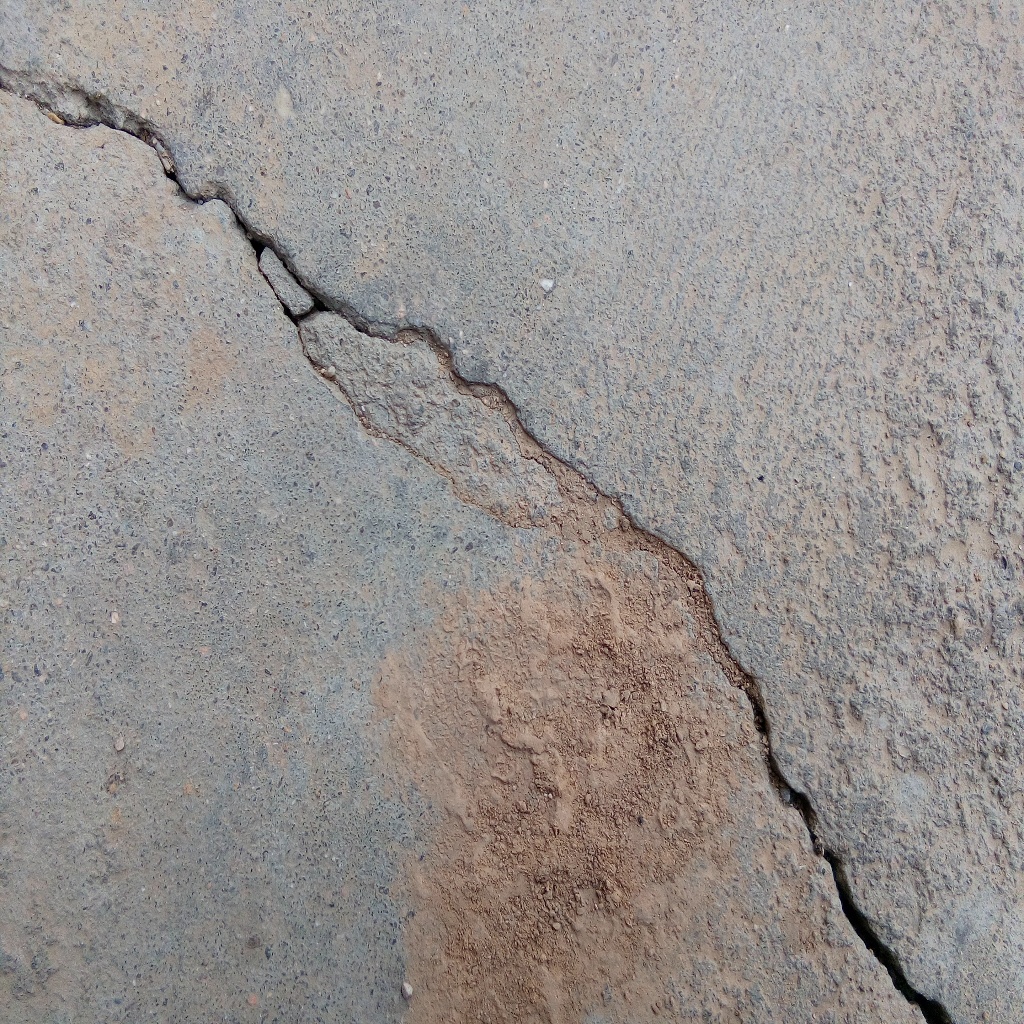

Supplement: S2 File — (ZIP) [file pone.0330218.s002.zip › 1 (249).jpg]

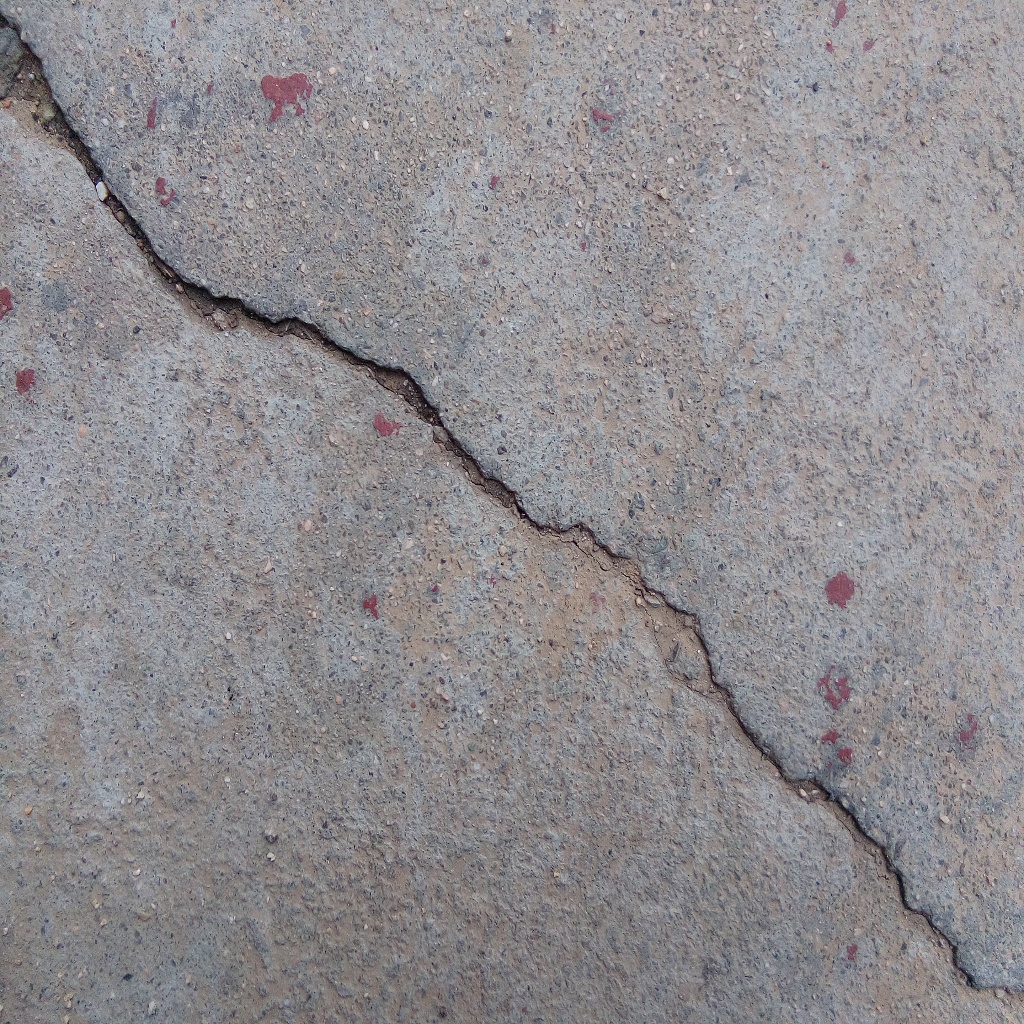

Supplement: S2 File — (ZIP) [file pone.0330218.s002.zip › 1 (252).jpg]

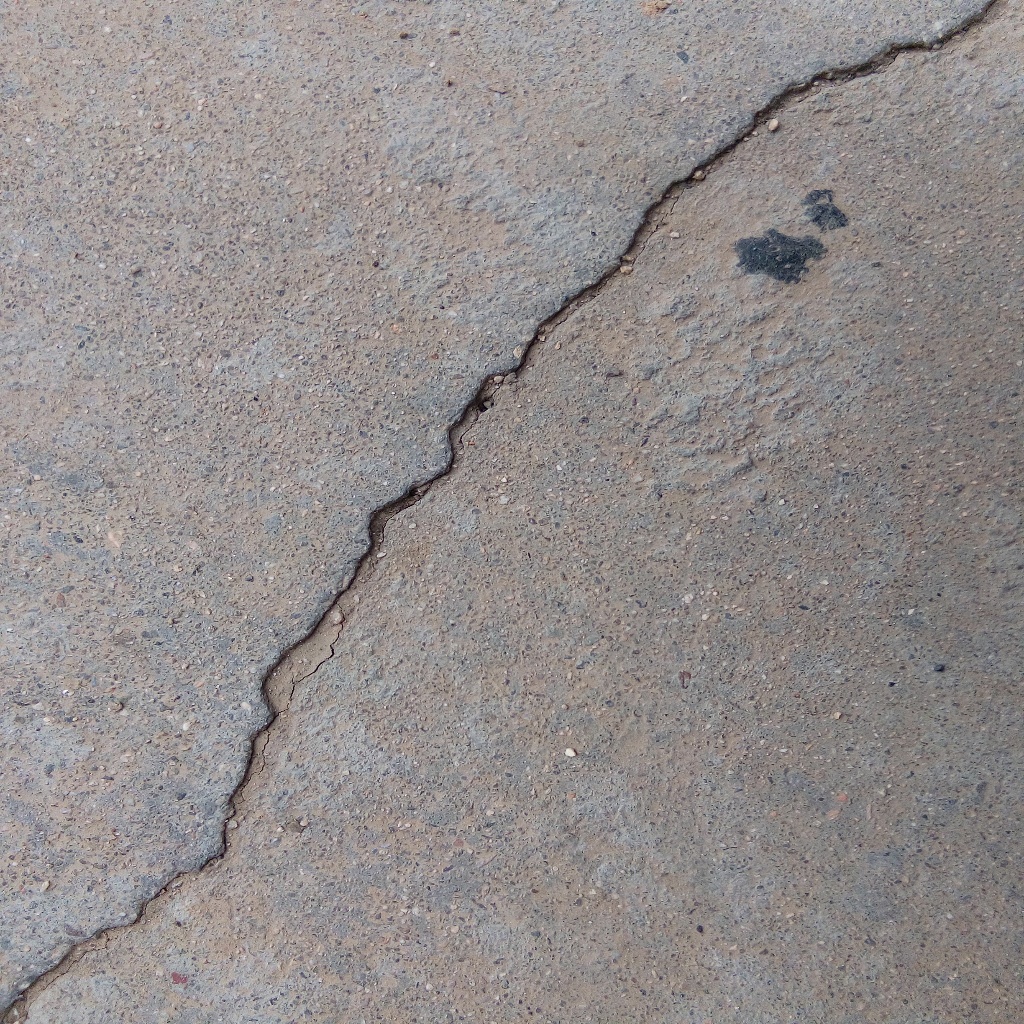

Supplement: S2 File — (ZIP) [file pone.0330218.s002.zip › 1 (253).jpg]

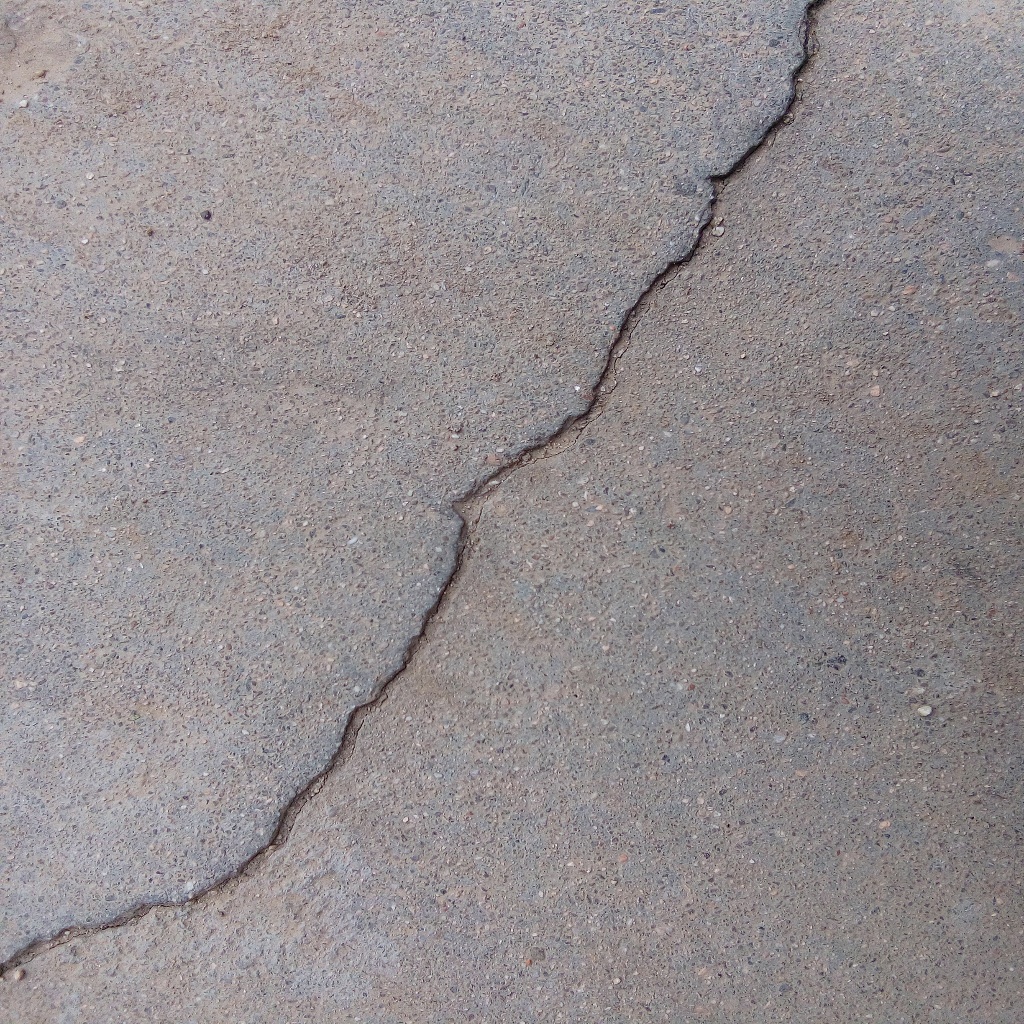

Supplement: S2 File — (ZIP) [file pone.0330218.s002.zip › 1 (254).jpg]

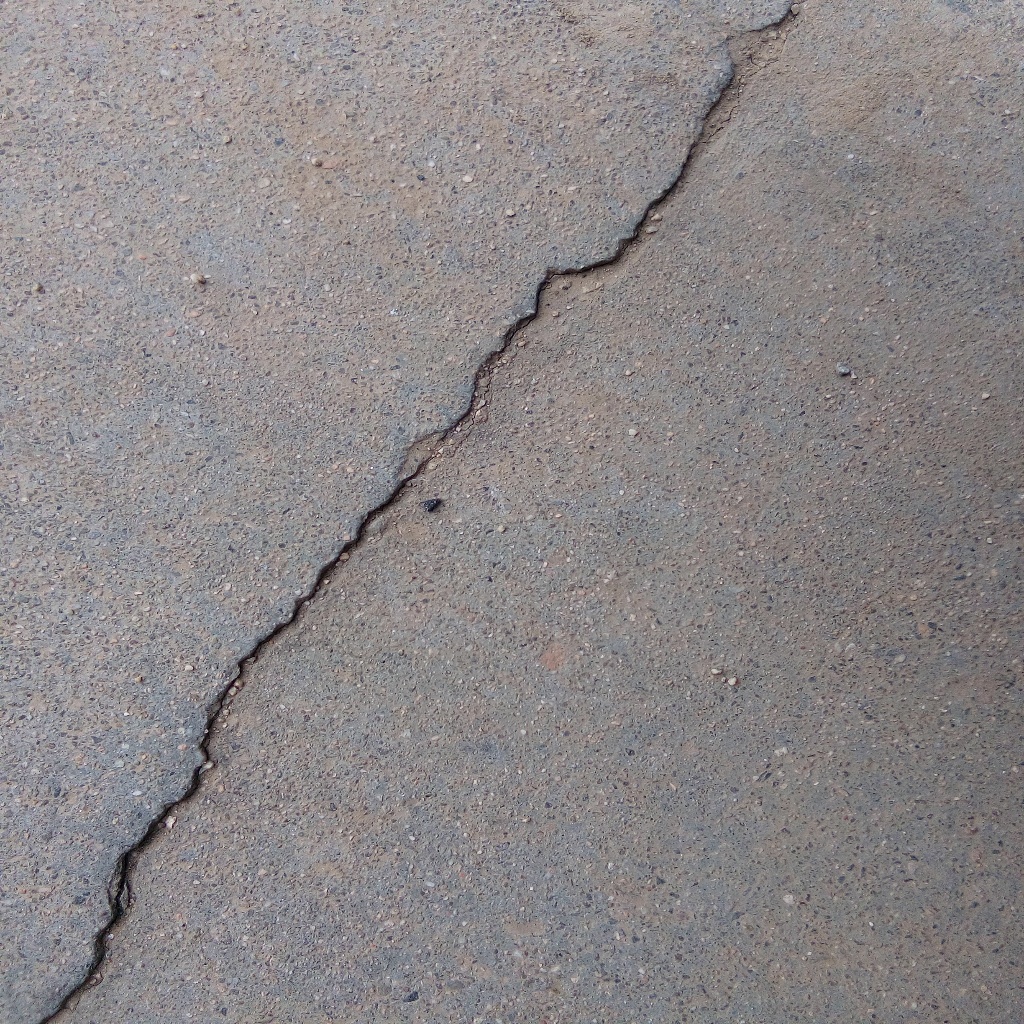

Supplement: S2 File — (ZIP) [file pone.0330218.s002.zip › 1 (255).jpg]

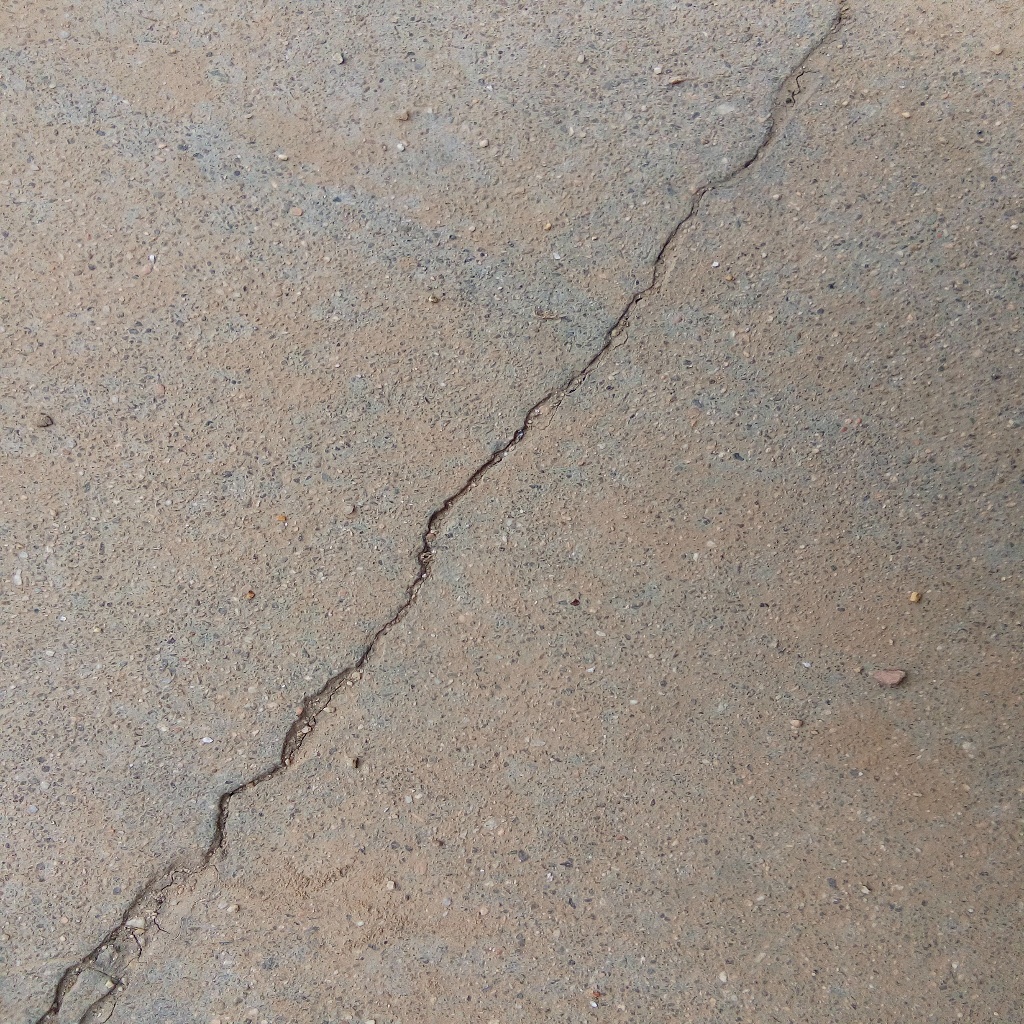

Supplement: S2 File — (ZIP) [file pone.0330218.s002.zip › 1 (256).jpg]

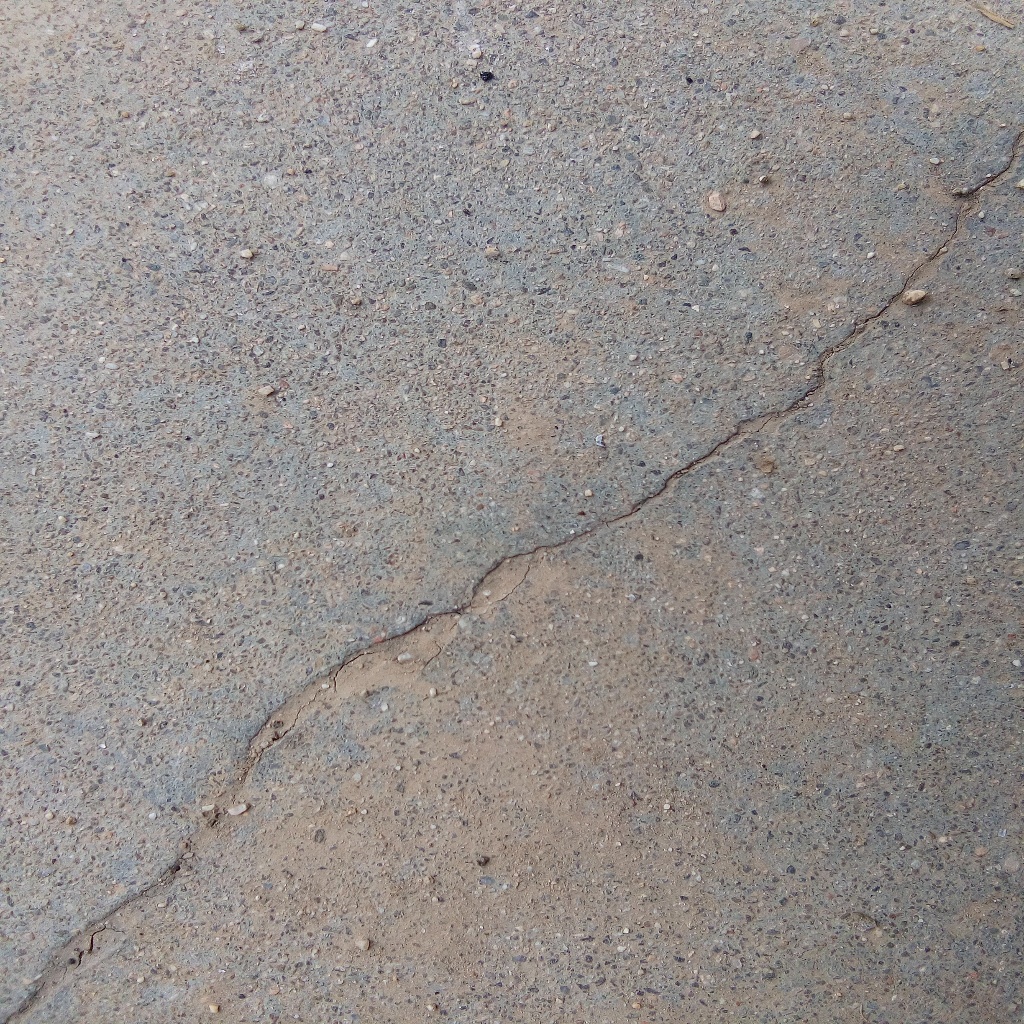

Supplement: S2 File — (ZIP) [file pone.0330218.s002.zip › 1 (257).jpg]

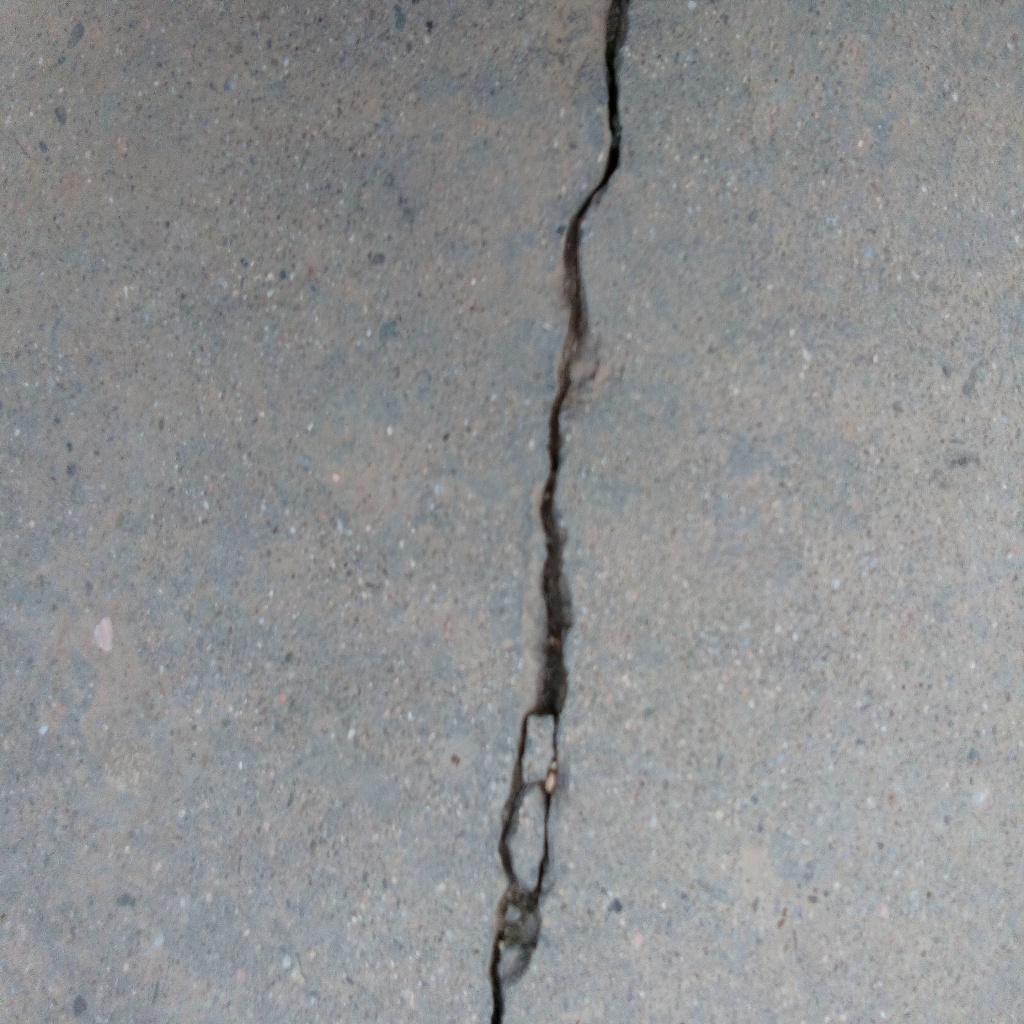

Supplement: S2 File — (ZIP) [file pone.0330218.s002.zip › 1 (258).jpg]

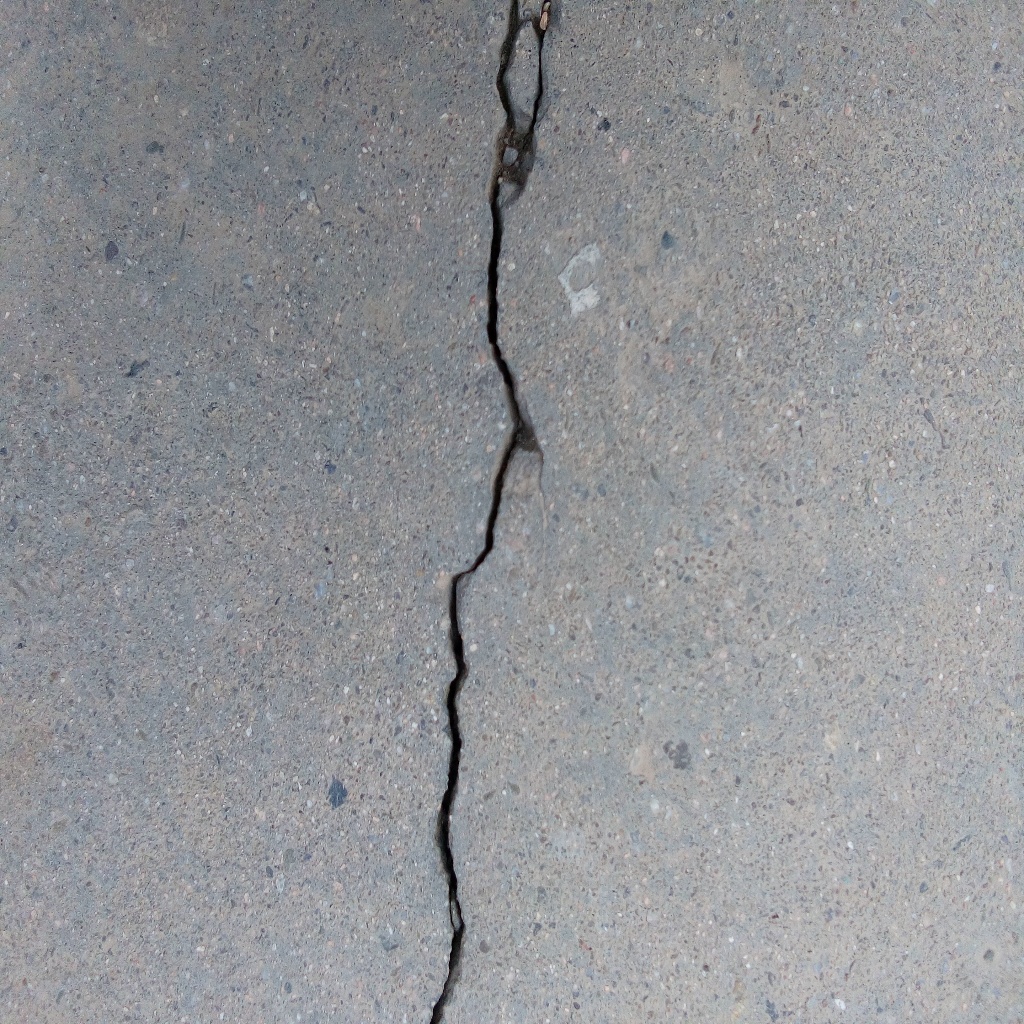

Supplement: S2 File — (ZIP) [file pone.0330218.s002.zip › 1 (259).jpg]

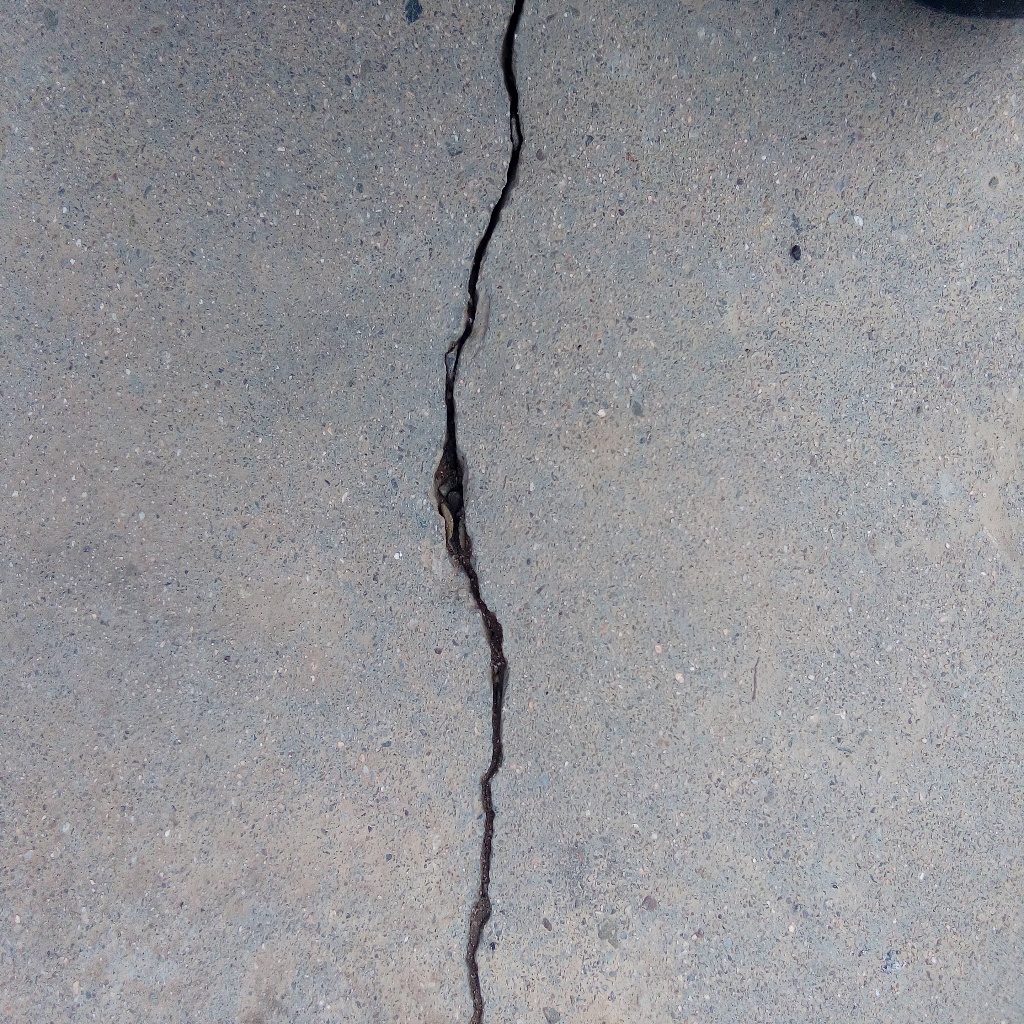

Supplement: S2 File — (ZIP) [file pone.0330218.s002.zip › 1 (260).jpg]

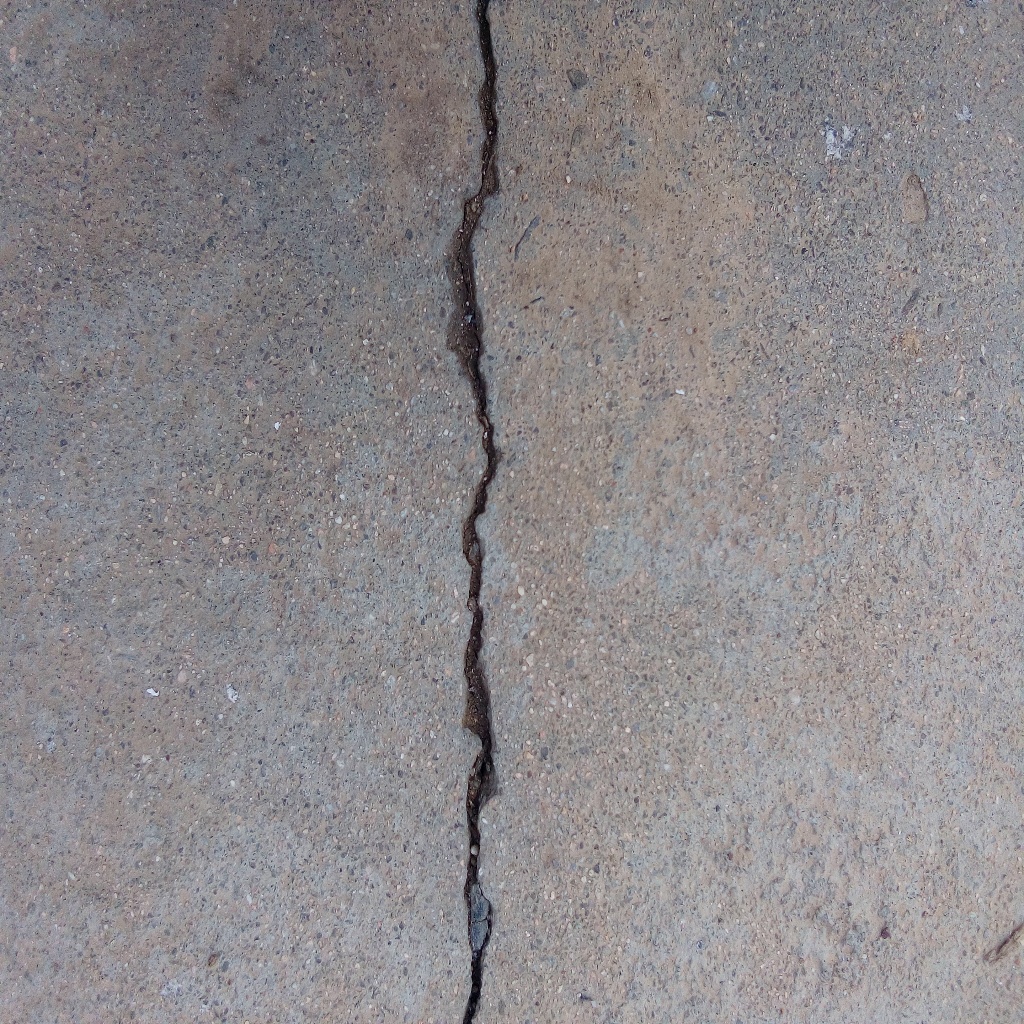

Supplement: S2 File — (ZIP) [file pone.0330218.s002.zip › 1 (261).jpg]

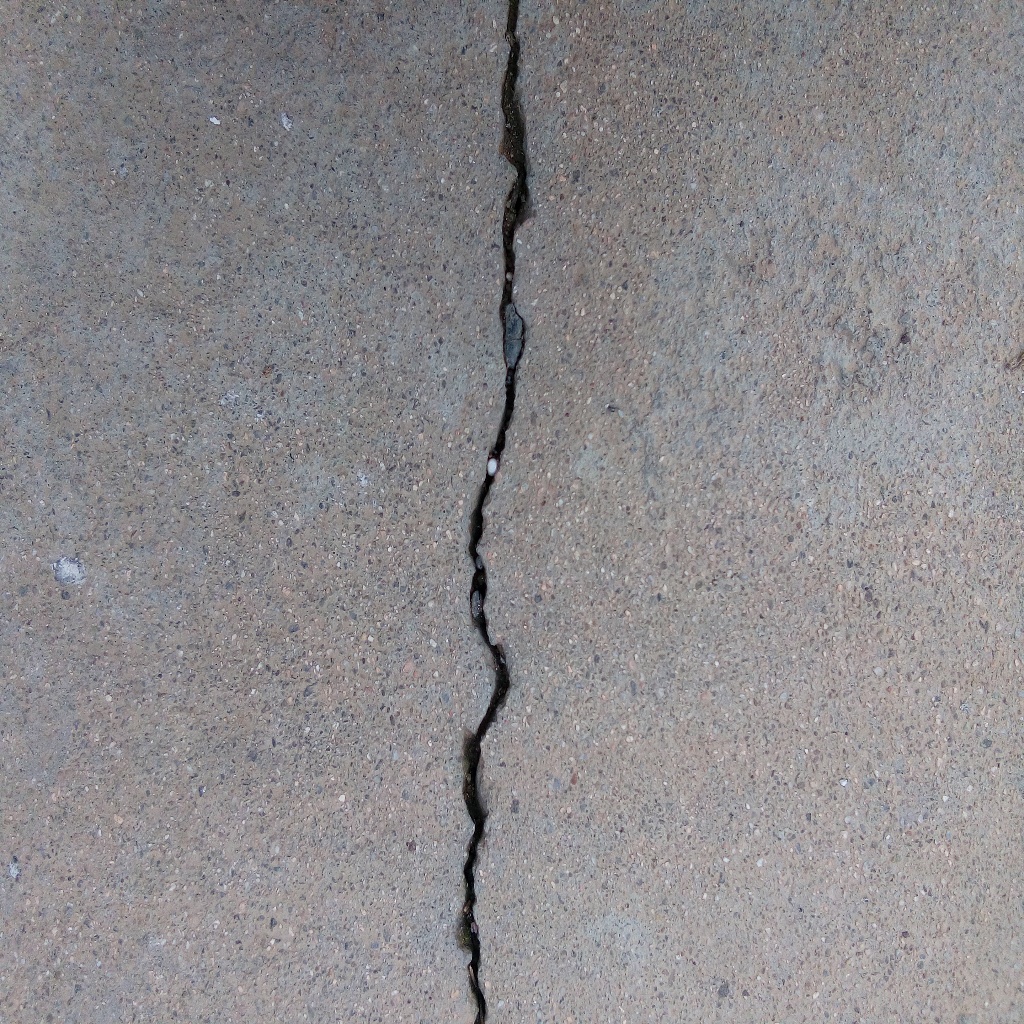

Supplement: S2 File — (ZIP) [file pone.0330218.s002.zip › 1 (262).jpg]

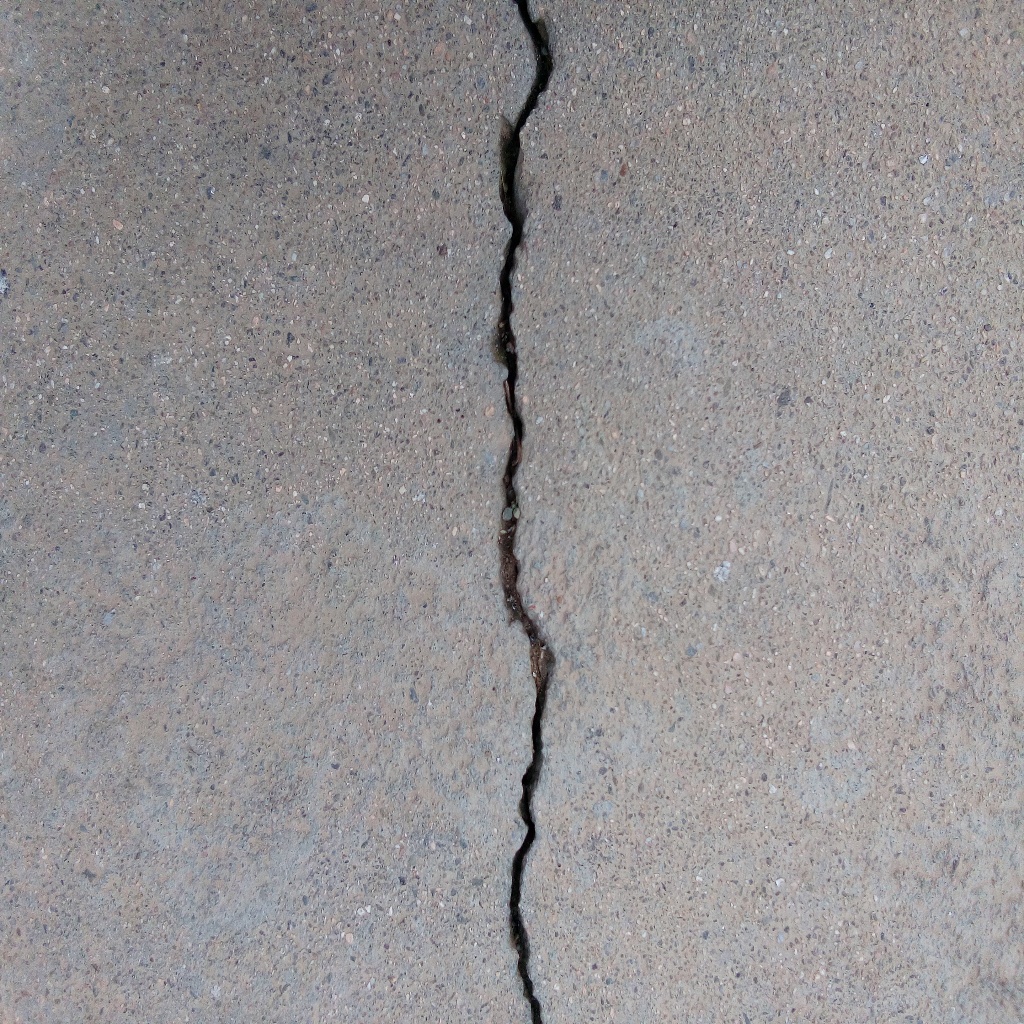

Supplement: S2 File — (ZIP) [file pone.0330218.s002.zip › 1 (263).jpg]

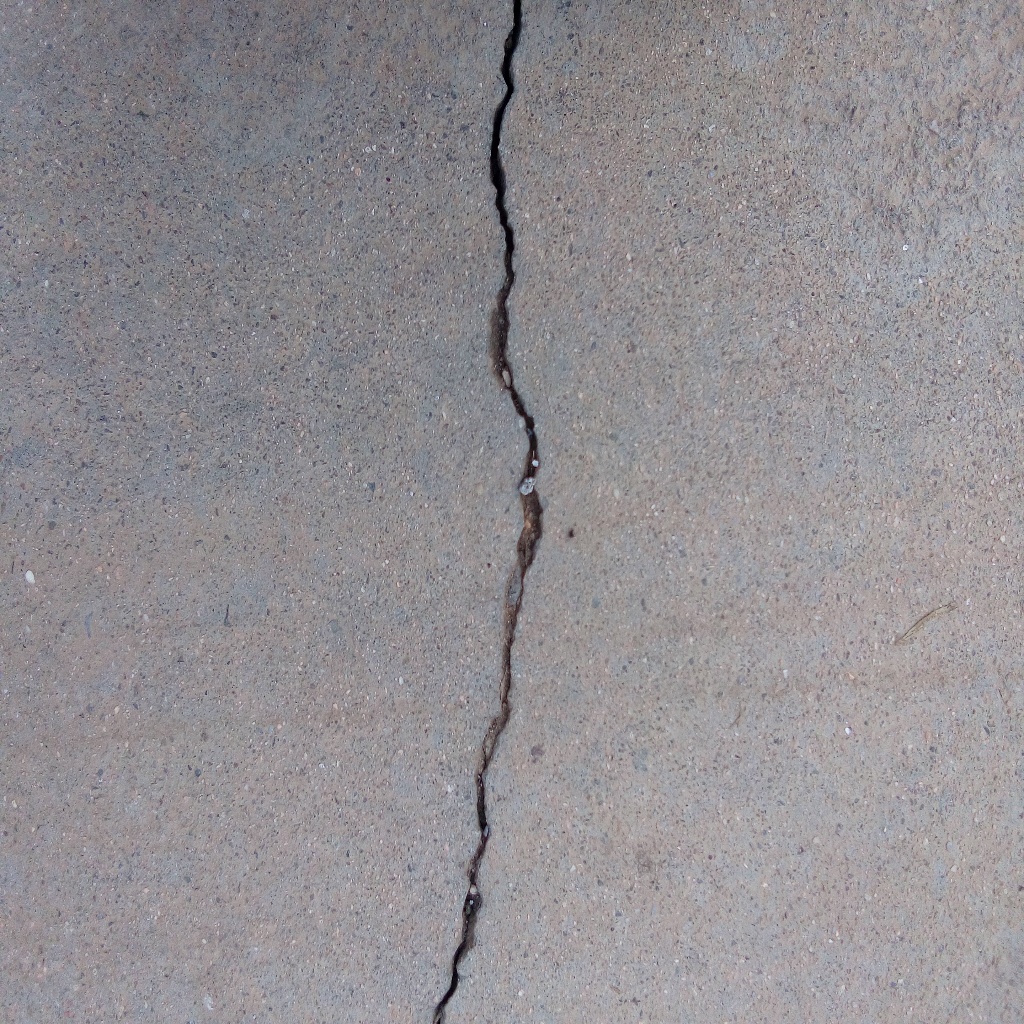

Supplement: S2 File — (ZIP) [file pone.0330218.s002.zip › 1 (264).jpg]

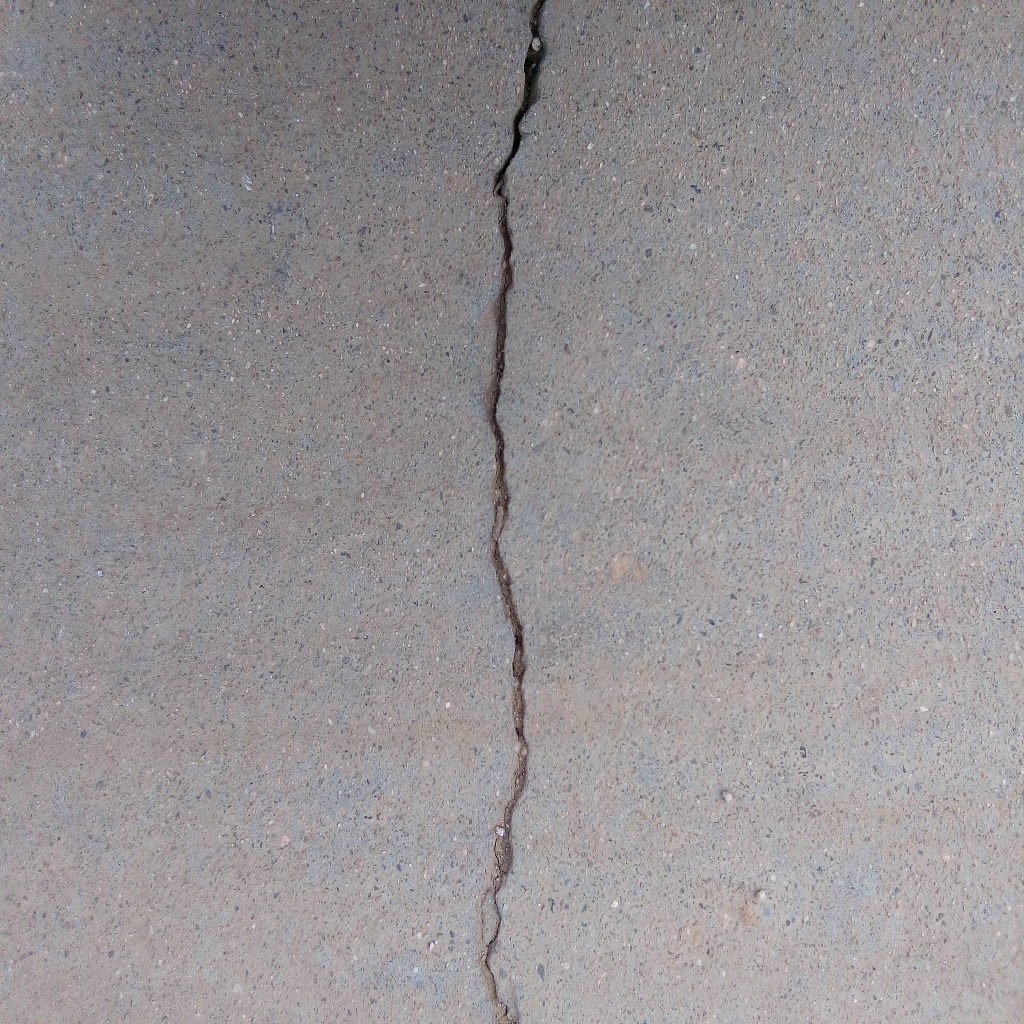

Supplement: S2 File — (ZIP) [file pone.0330218.s002.zip › 1 (265).jpg]

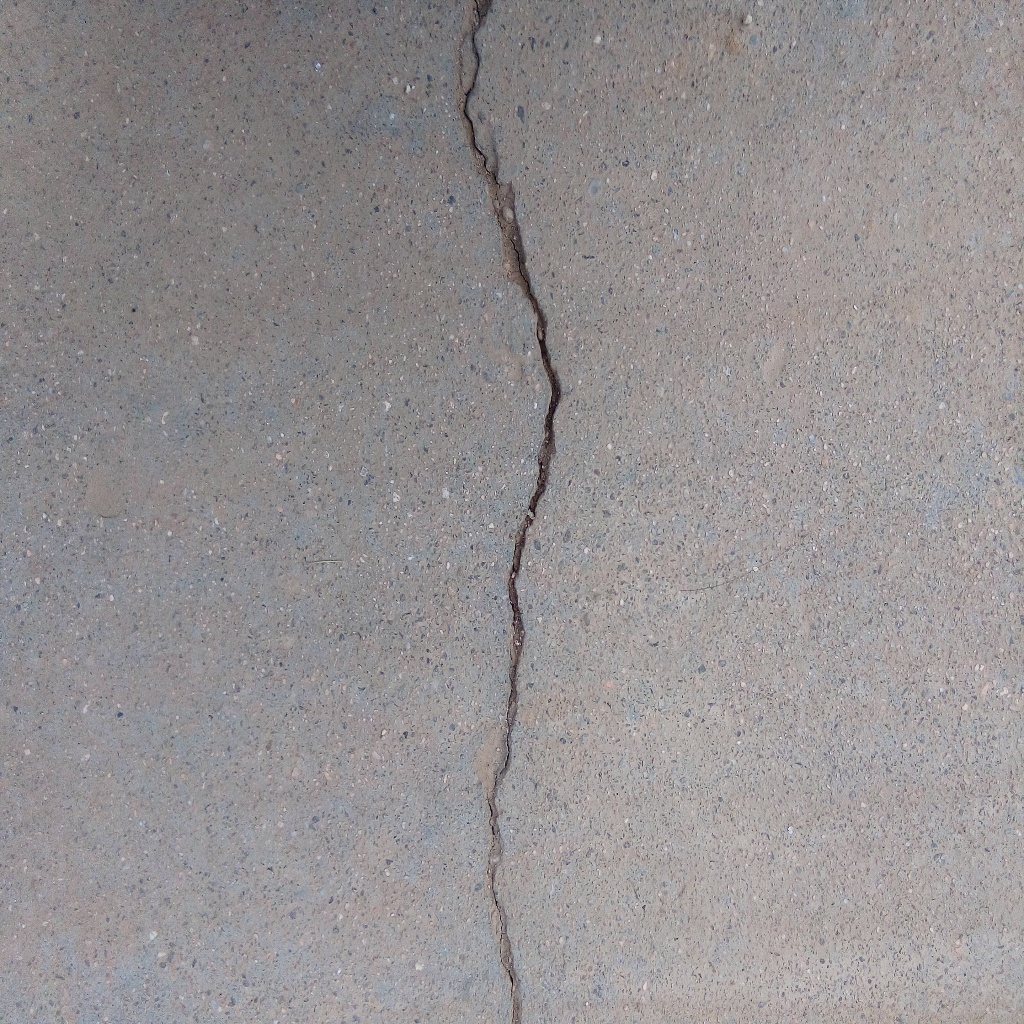

Supplement: S2 File — (ZIP) [file pone.0330218.s002.zip › 1 (266).jpg]

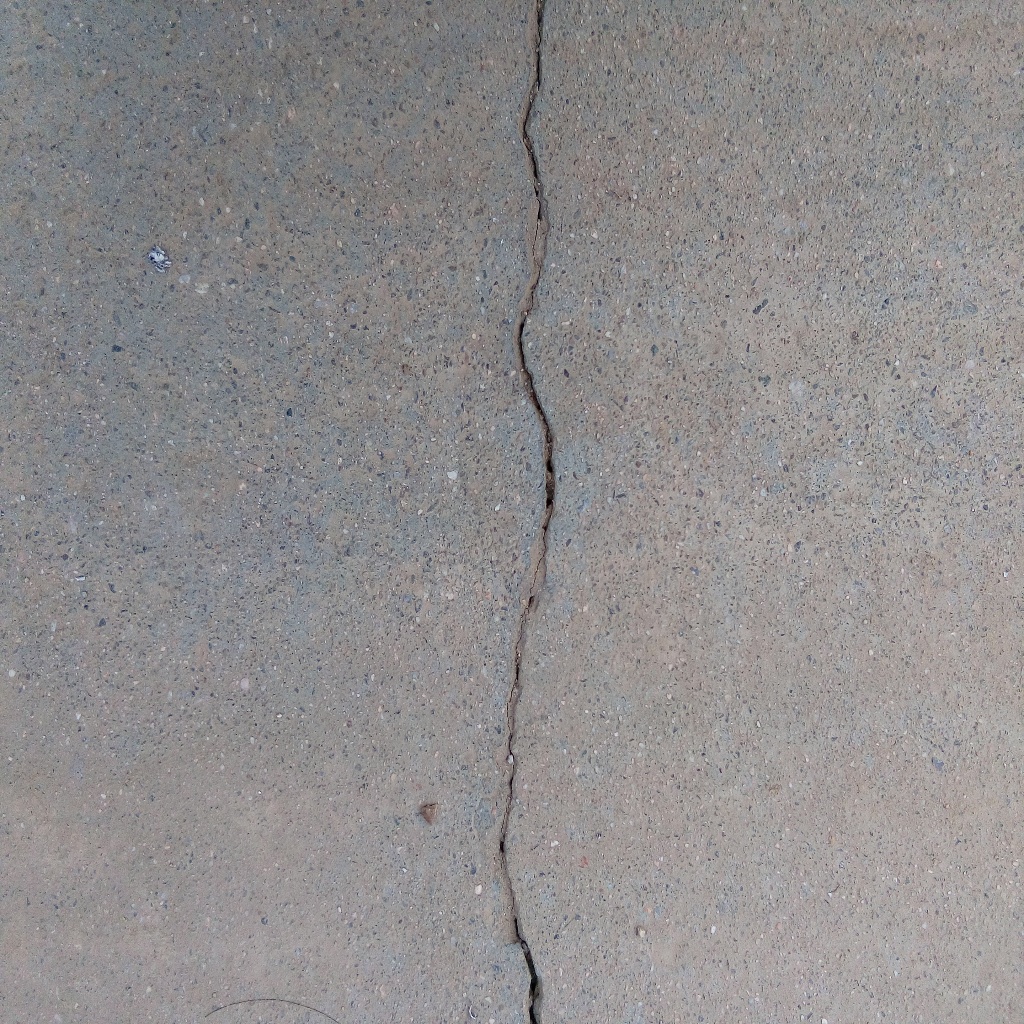

Supplement: S2 File — (ZIP) [file pone.0330218.s002.zip › 1 (267).jpg]

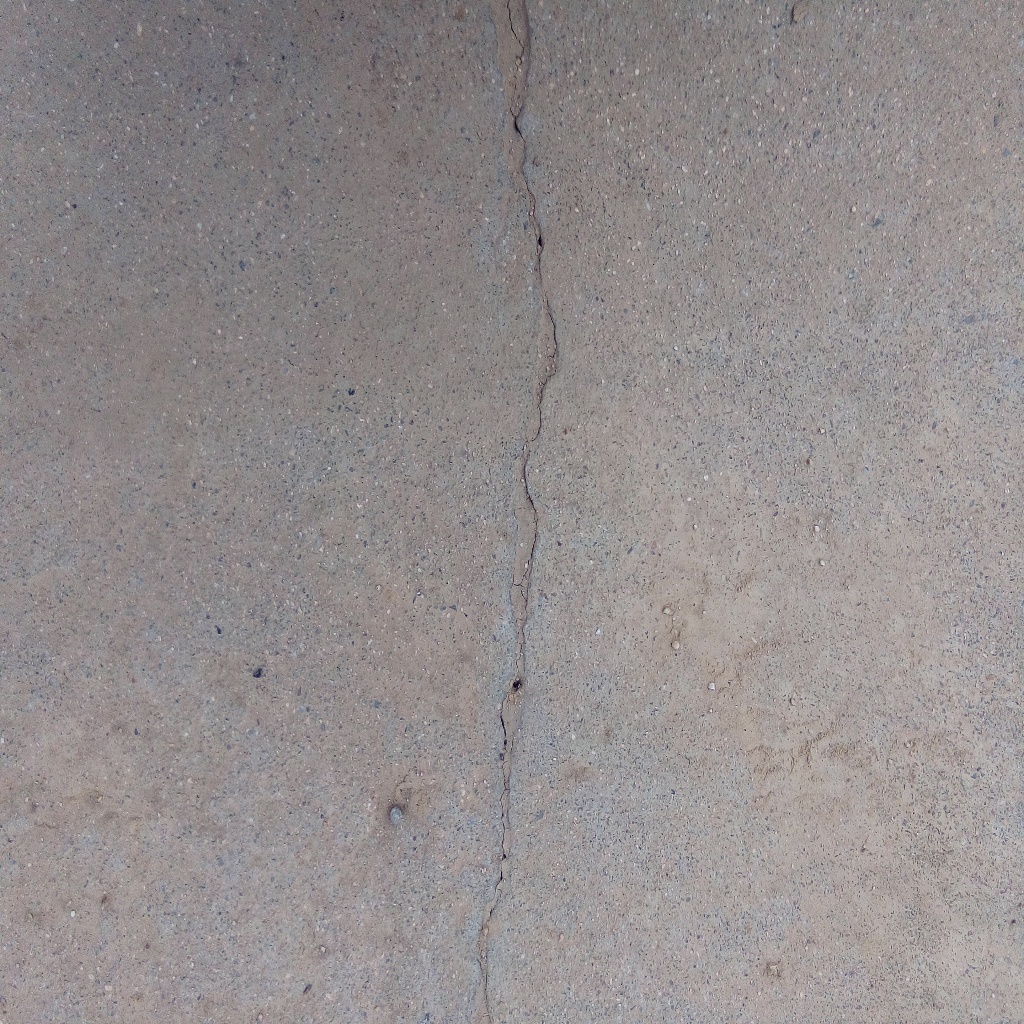

Supplement: S2 File — (ZIP) [file pone.0330218.s002.zip › 1 (268).jpg]

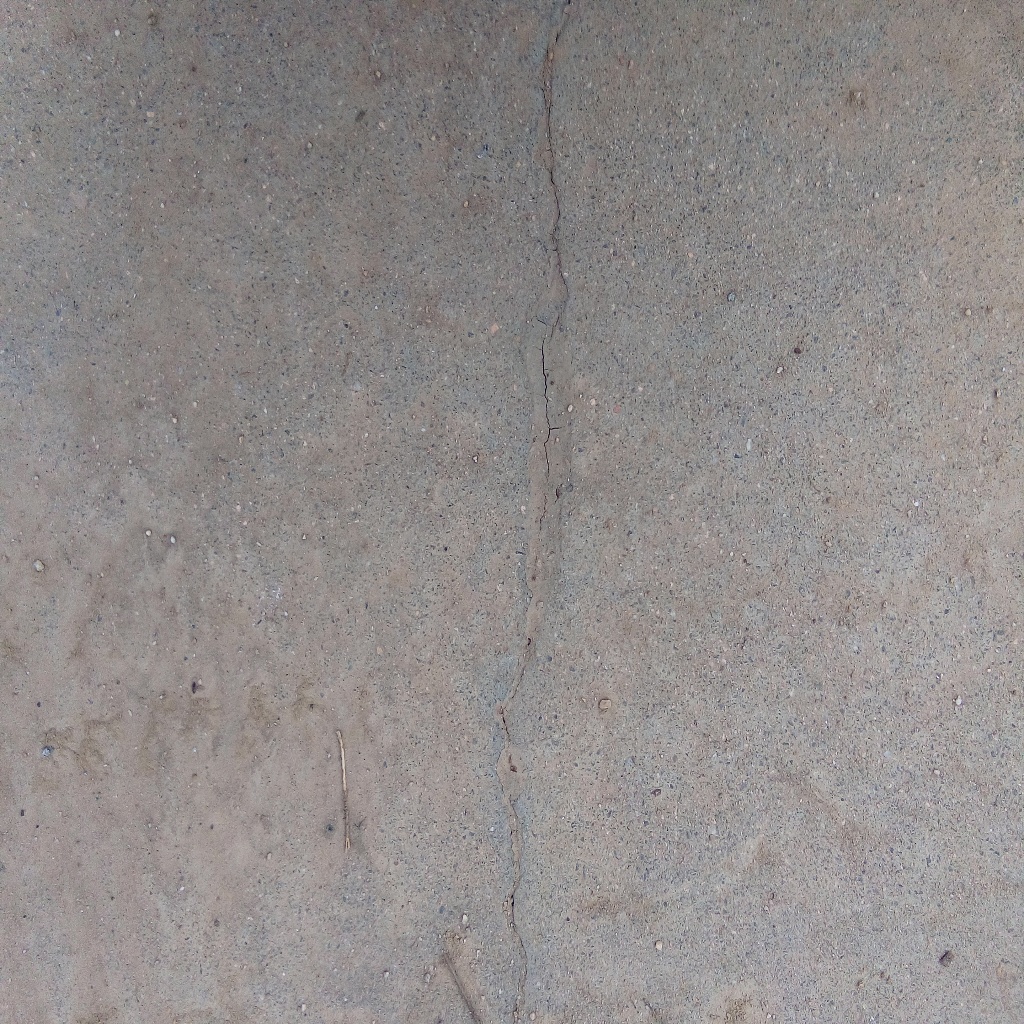

Supplement: S2 File — (ZIP) [file pone.0330218.s002.zip › 1 (269).jpg]

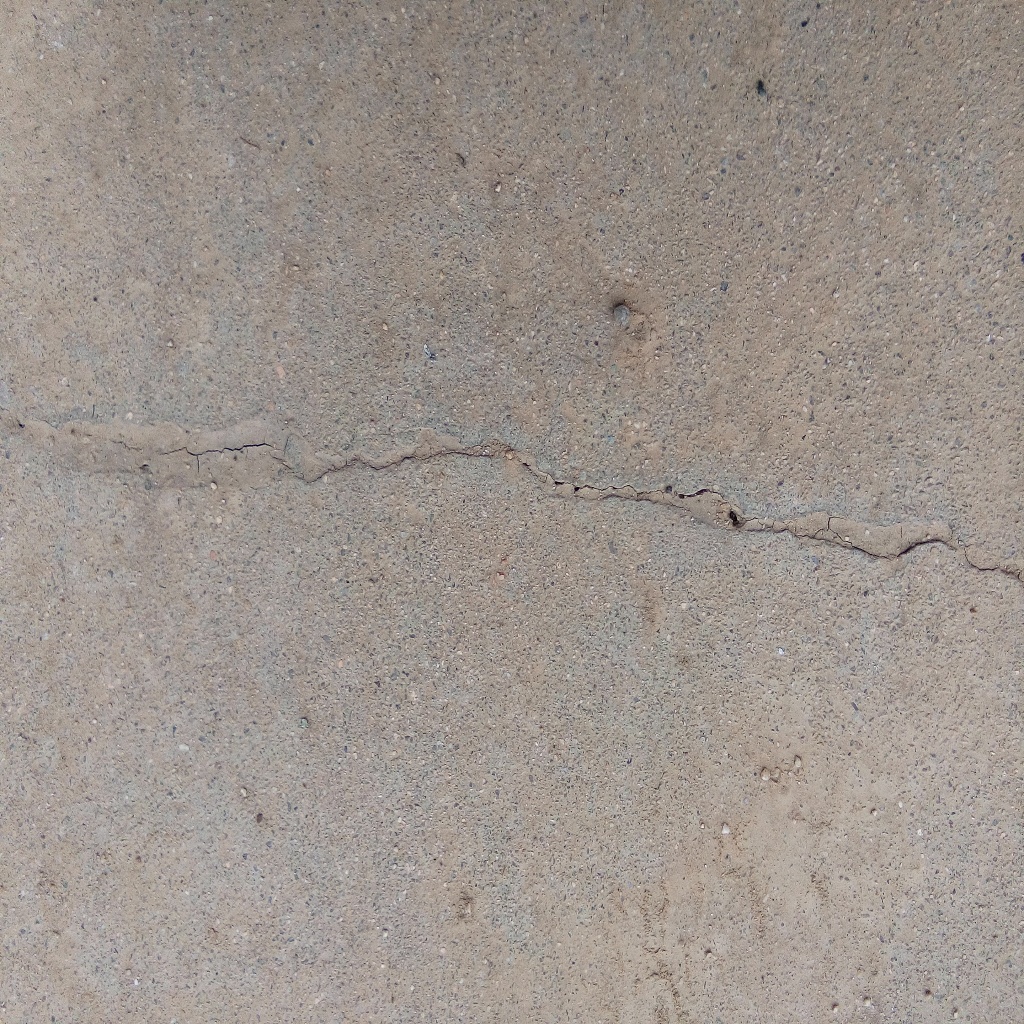

Supplement: S2 File — (ZIP) [file pone.0330218.s002.zip › 1 (270).jpg]

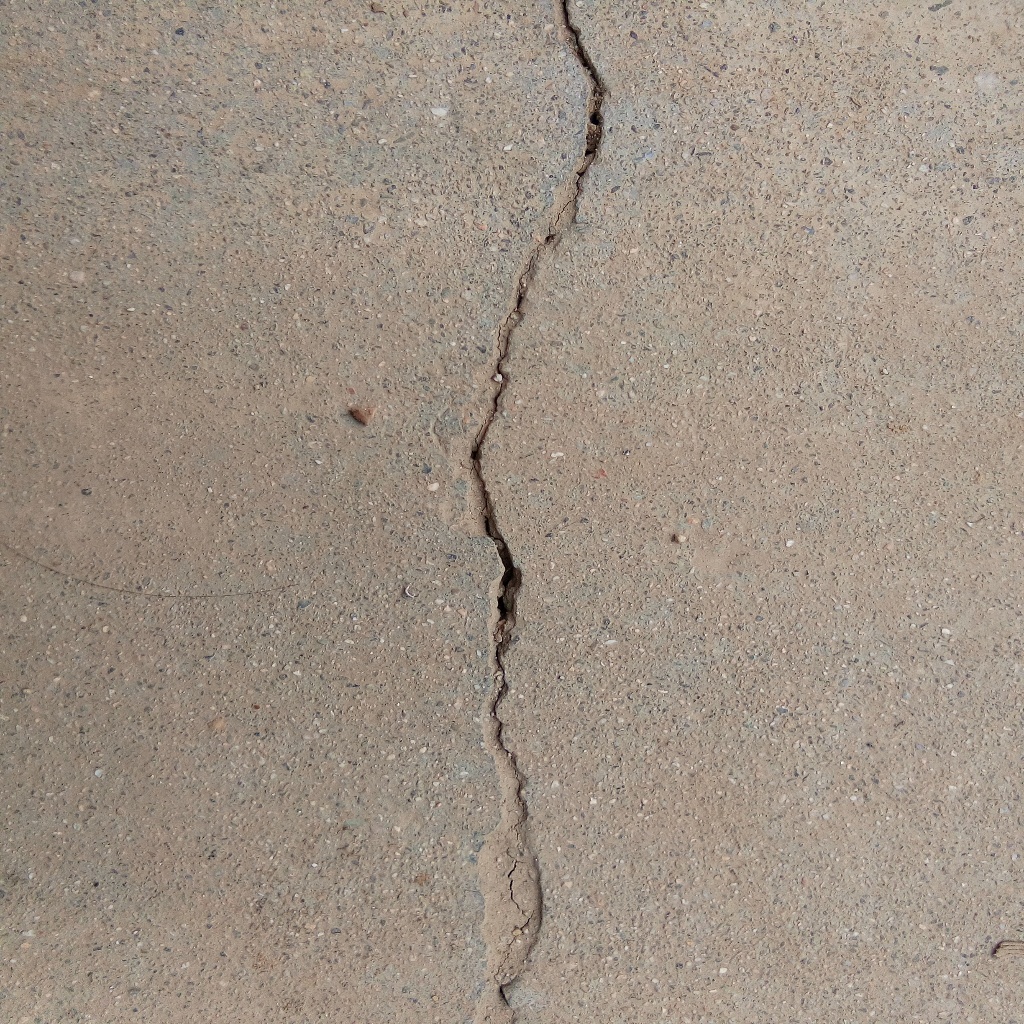

Supplement: S2 File — (ZIP) [file pone.0330218.s002.zip › 1 (271).jpg]

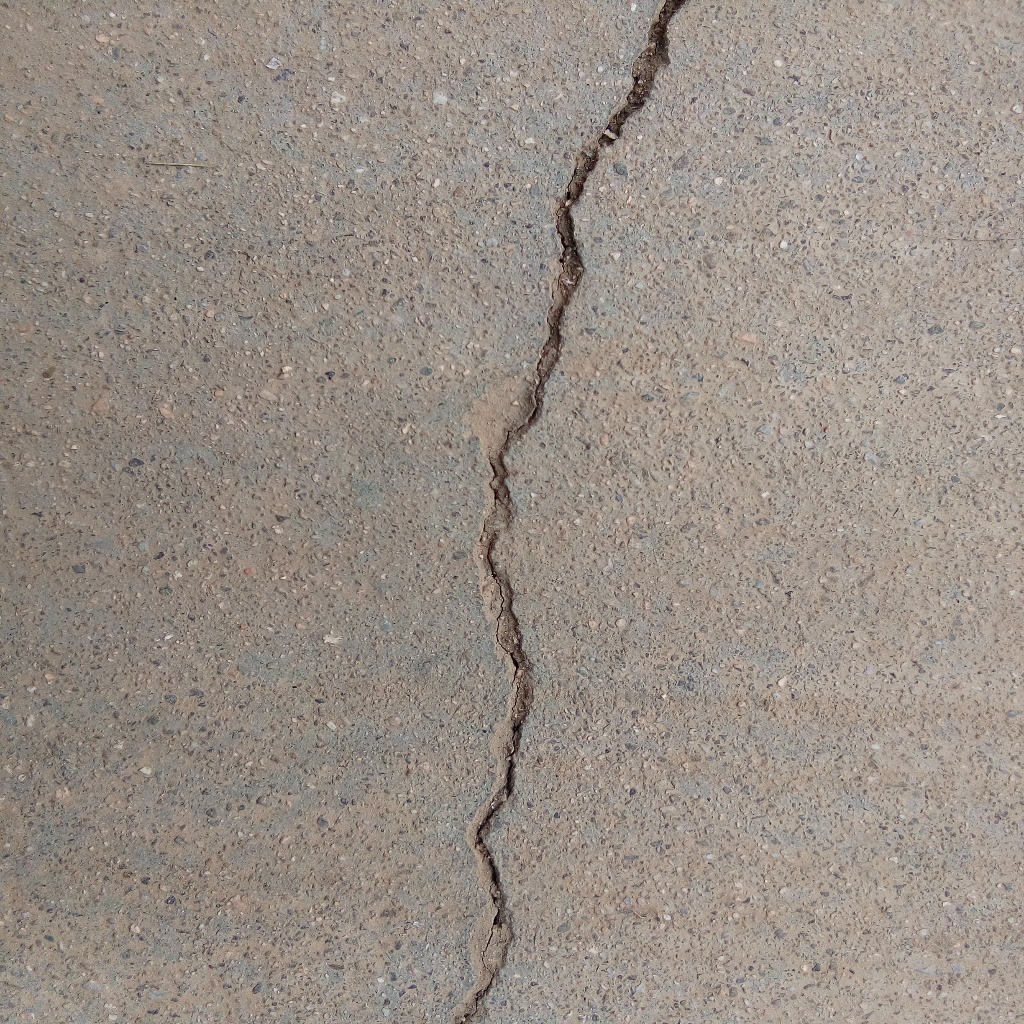

Supplement: S2 File — (ZIP) [file pone.0330218.s002.zip › 1 (272).jpg]

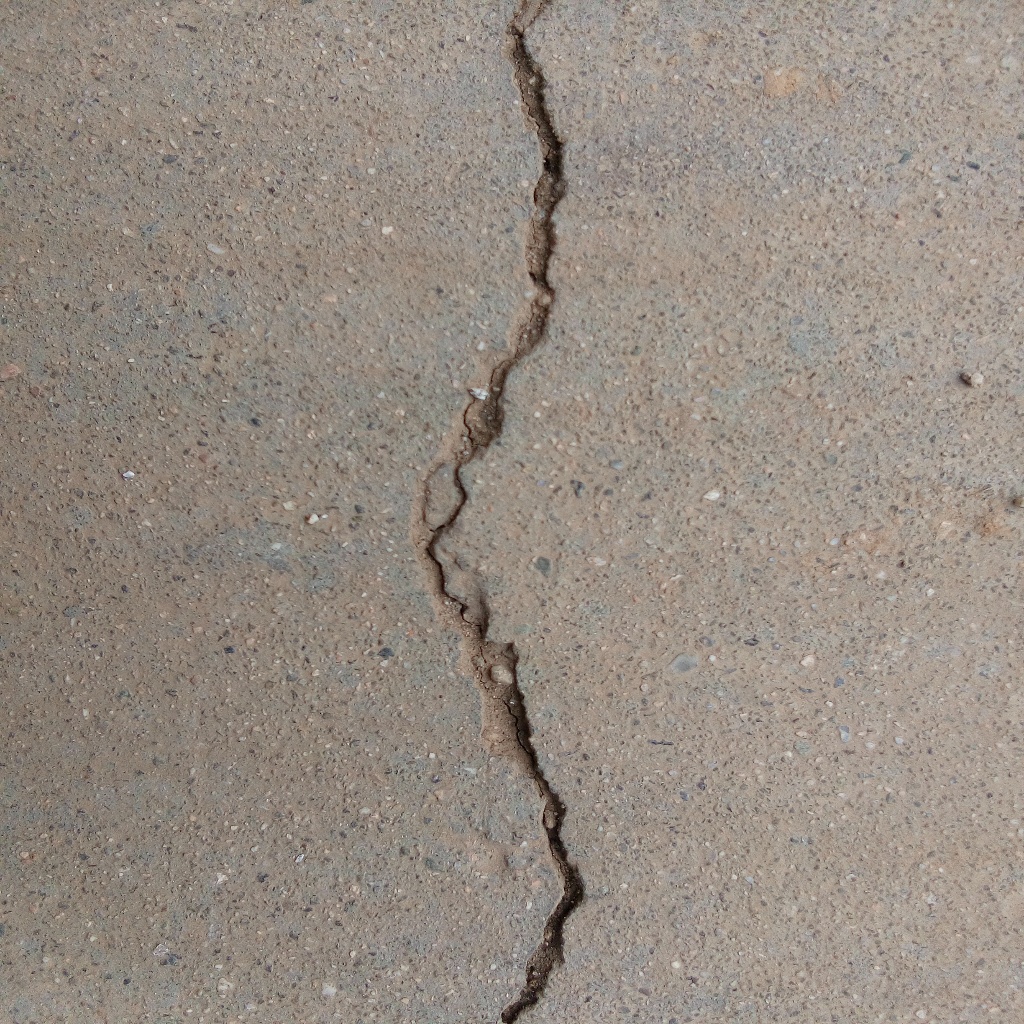

Supplement: S2 File — (ZIP) [file pone.0330218.s002.zip › 1 (273).jpg]

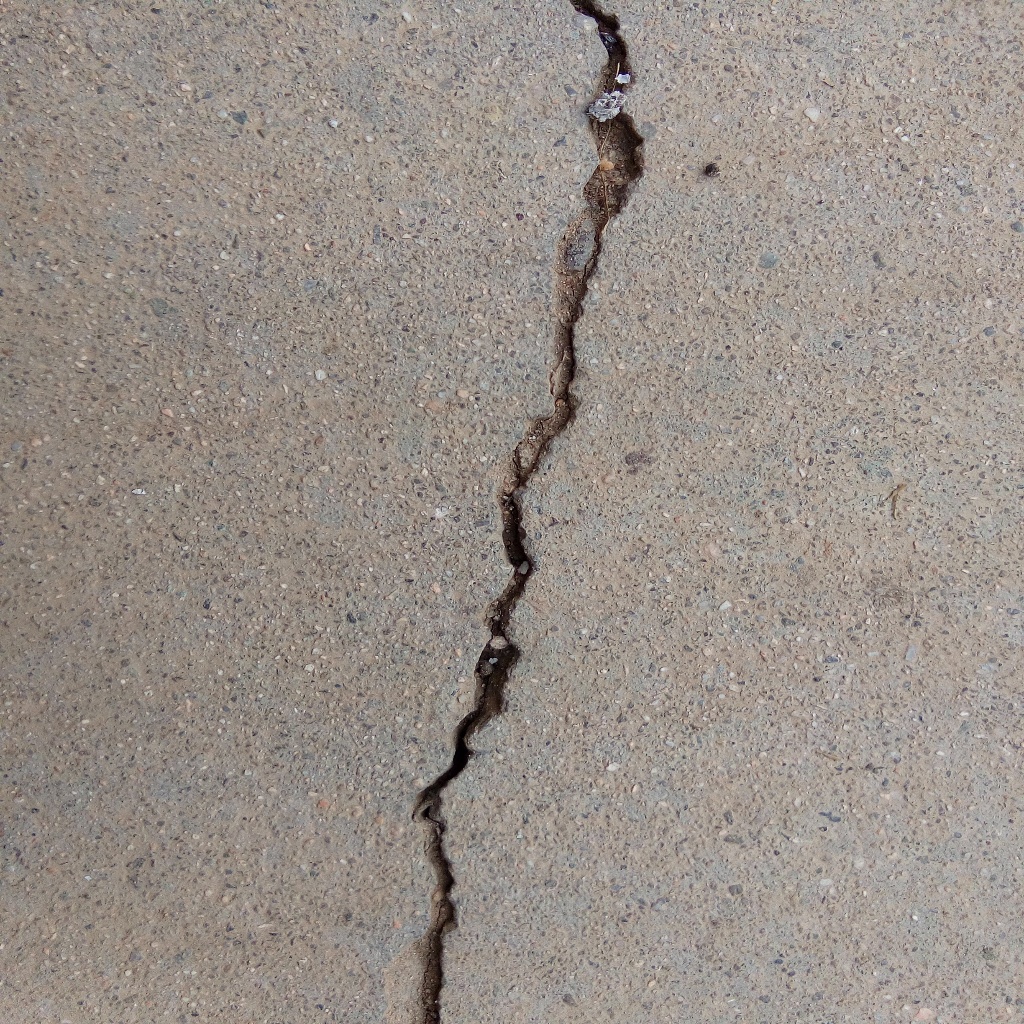

Supplement: S2 File — (ZIP) [file pone.0330218.s002.zip › 1 (274).jpg]

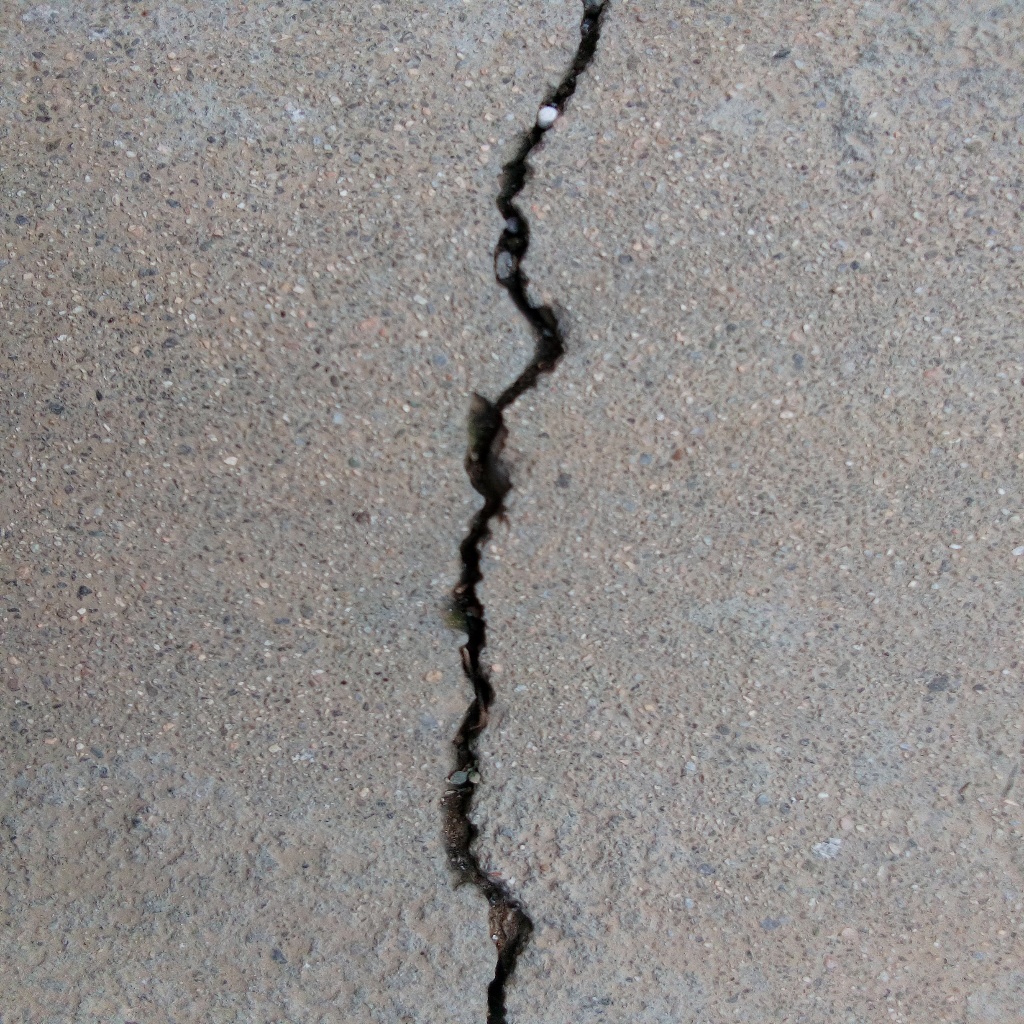

Supplement: S2 File — (ZIP) [file pone.0330218.s002.zip › 1 (275).jpg]

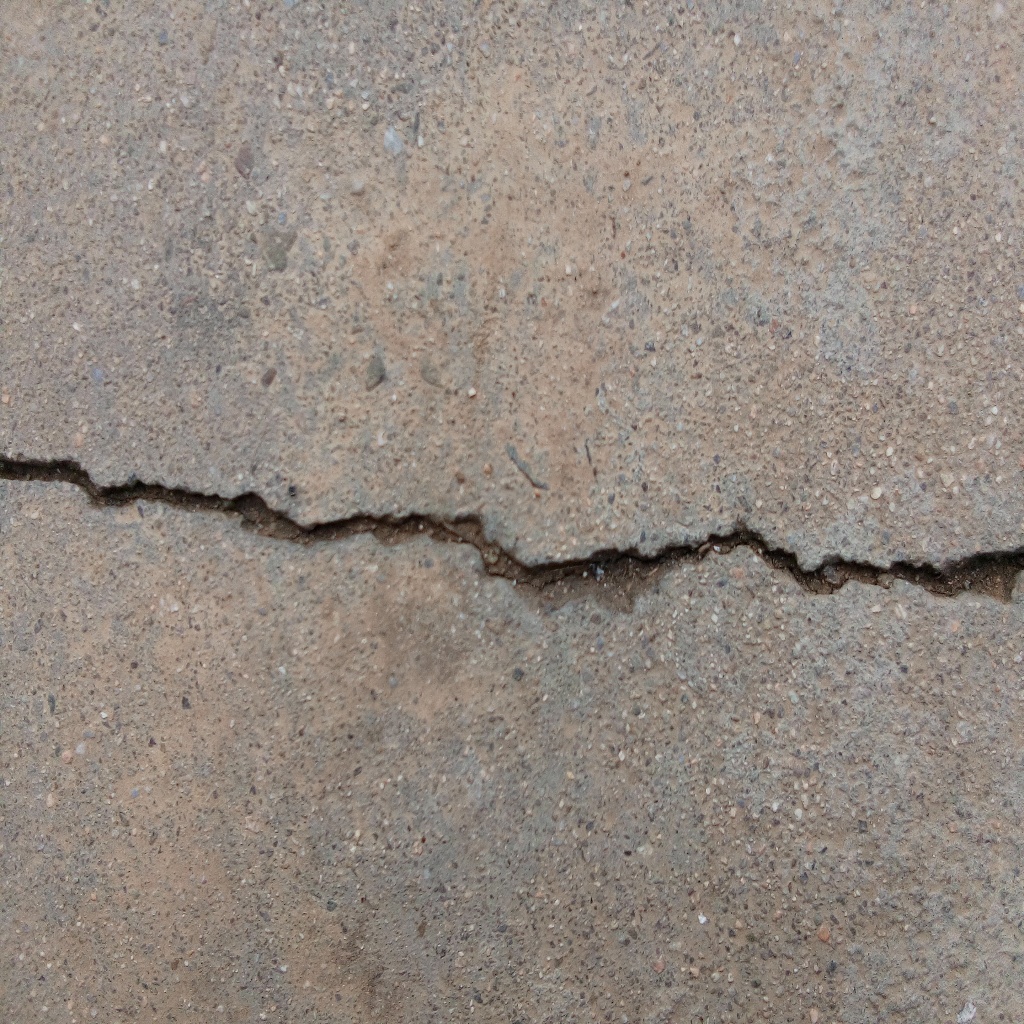

Supplement: S2 File — (ZIP) [file pone.0330218.s002.zip › 1 (276).jpg]

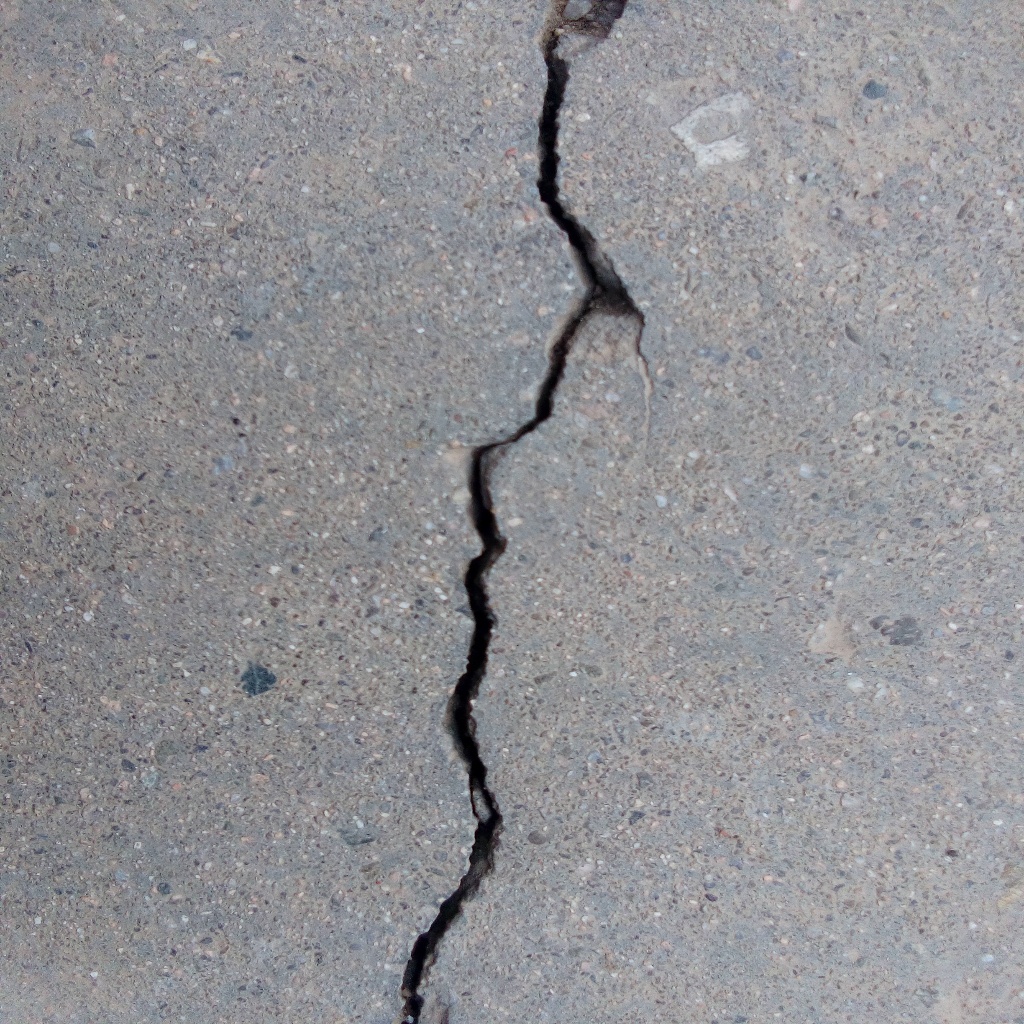

Supplement: S2 File — (ZIP) [file pone.0330218.s002.zip › 1 (277).jpg]

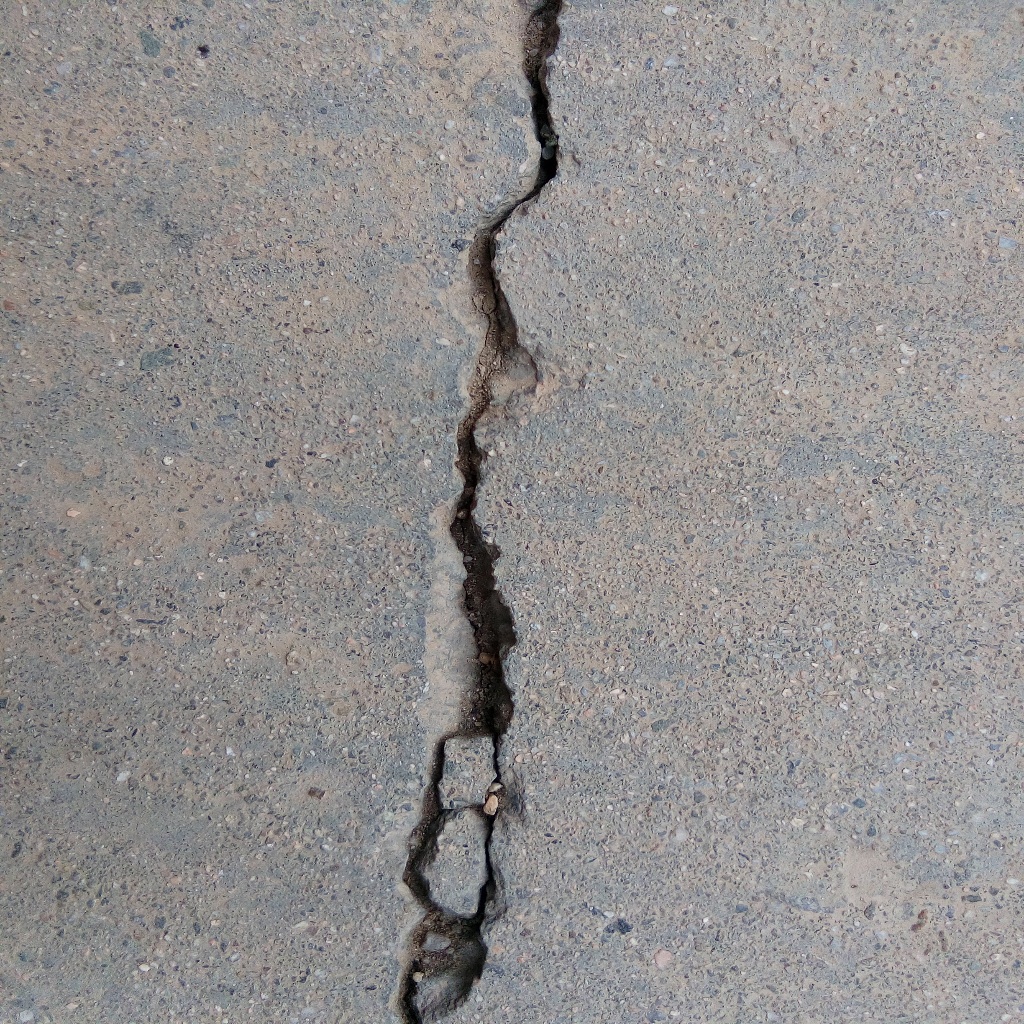

Supplement: S2 File — (ZIP) [file pone.0330218.s002.zip › 1 (278).jpg]

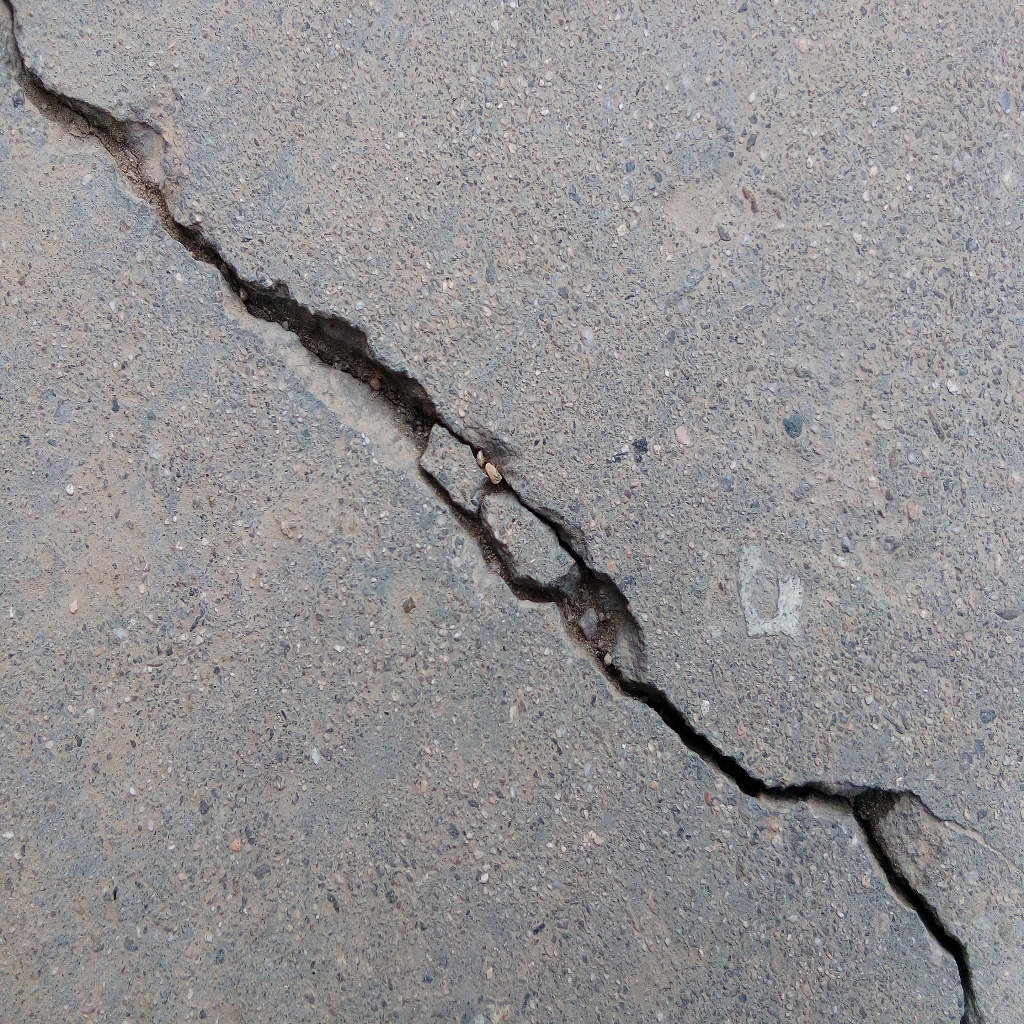

Supplement: S2 File — (ZIP) [file pone.0330218.s002.zip › 1 (279).jpg]

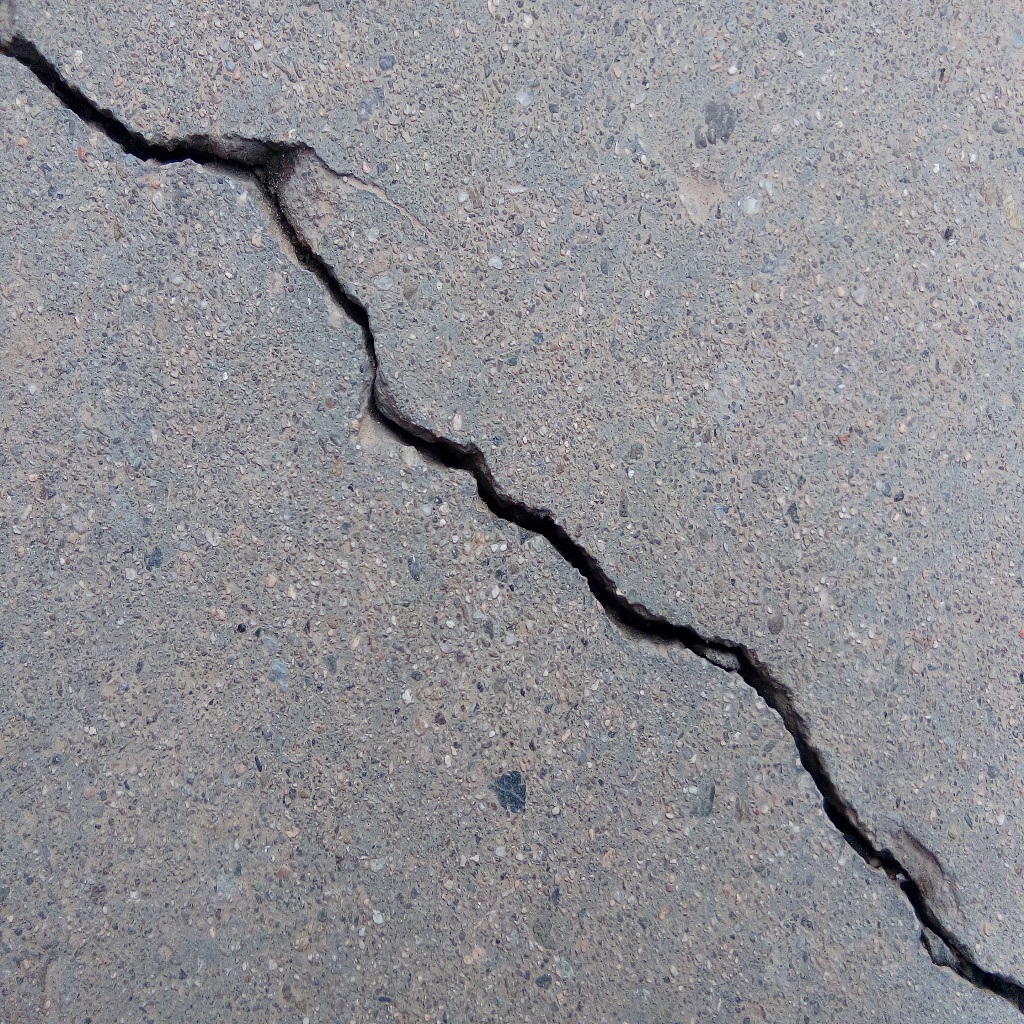

Supplement: S2 File — (ZIP) [file pone.0330218.s002.zip › 1 (280).jpg]

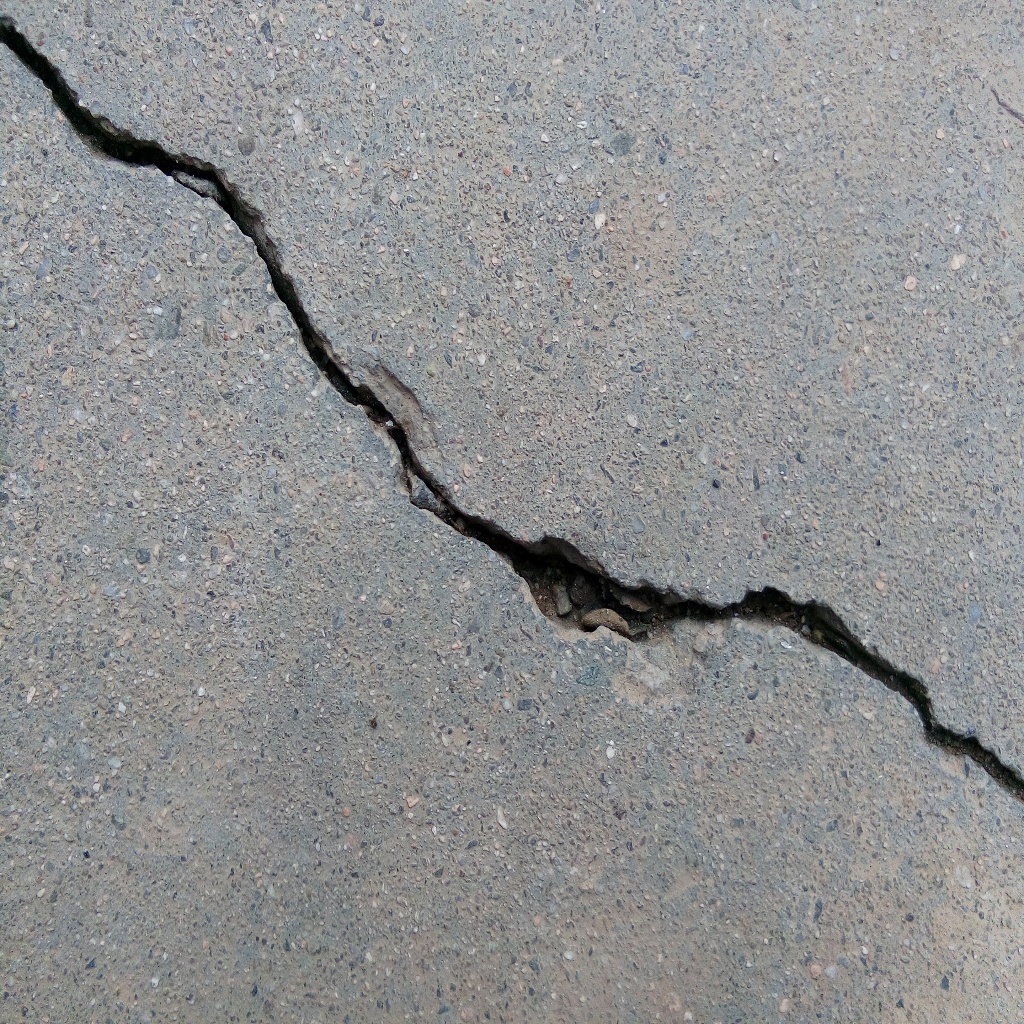

Supplement: S2 File — (ZIP) [file pone.0330218.s002.zip › 1 (281).jpg]

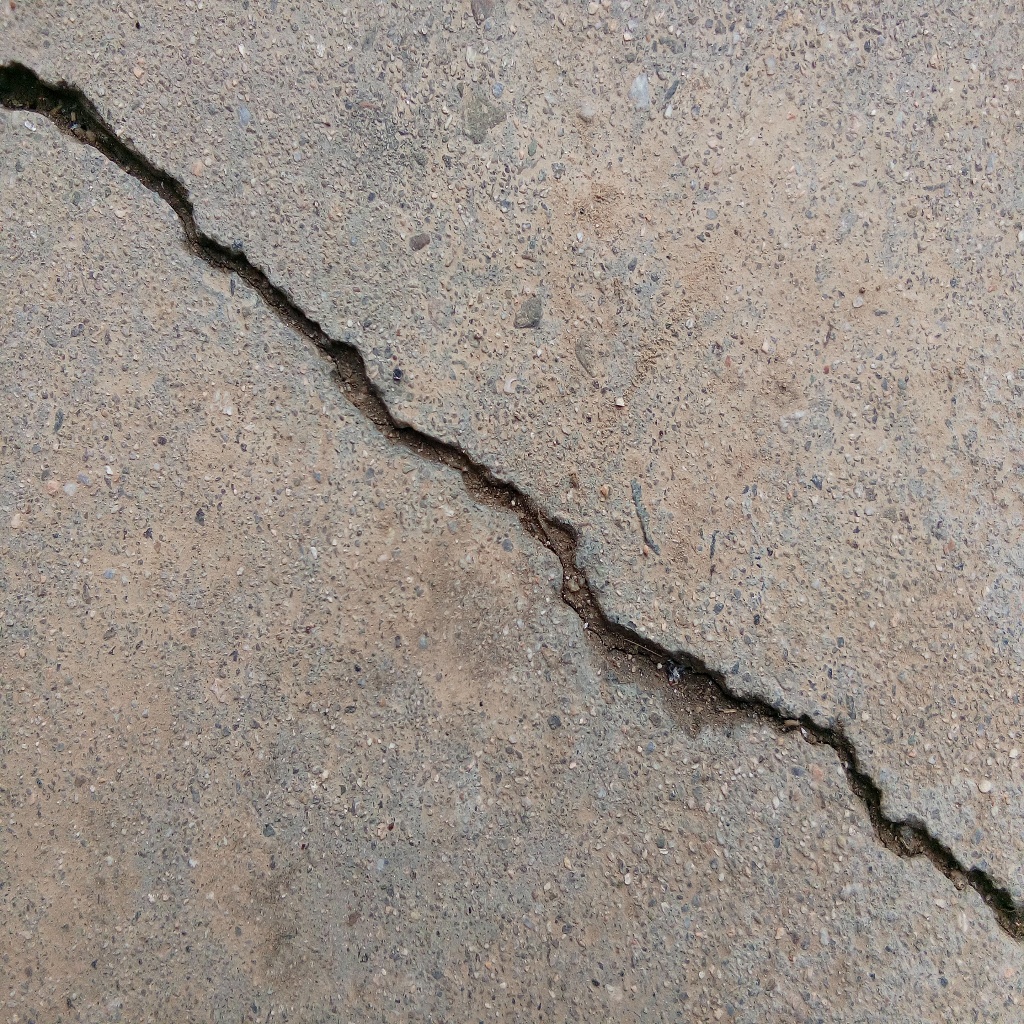

Supplement: S2 File — (ZIP) [file pone.0330218.s002.zip › 1 (282).jpg]

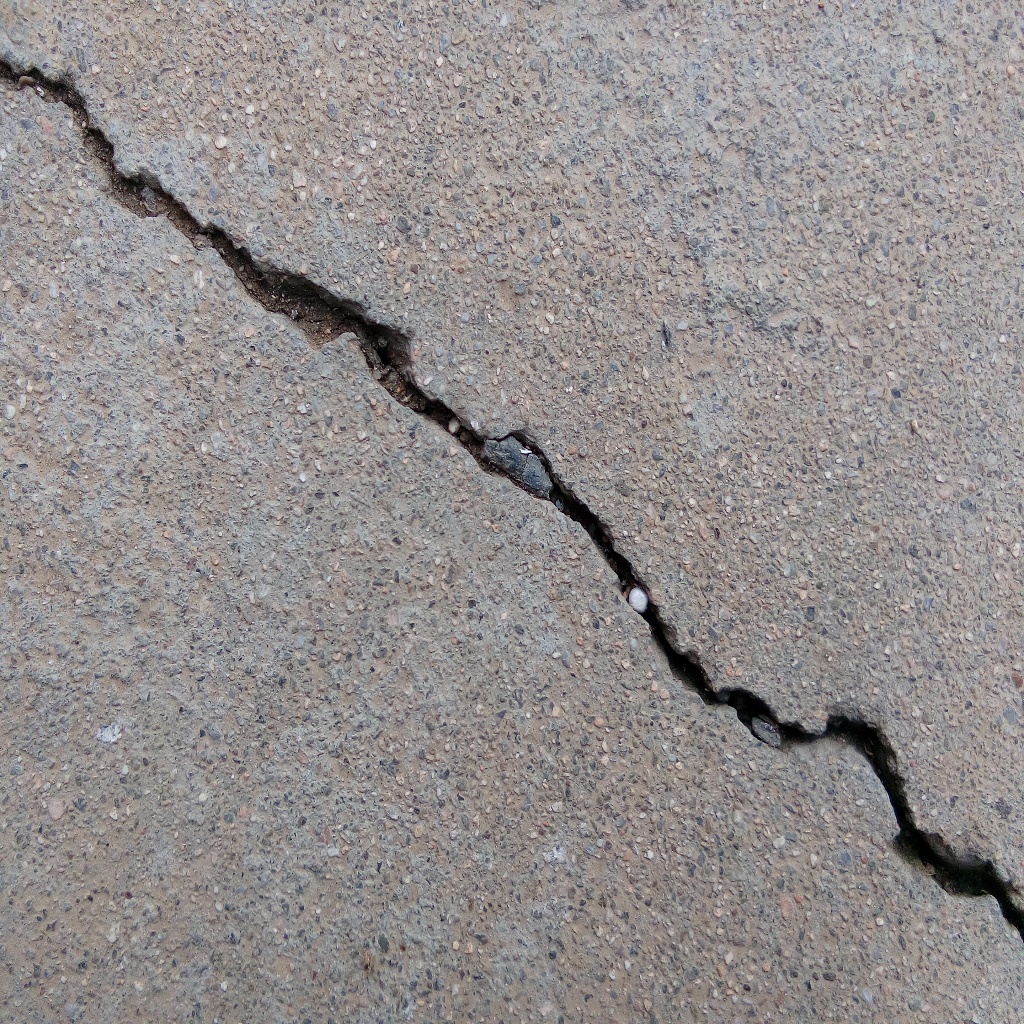

Supplement: S2 File — (ZIP) [file pone.0330218.s002.zip › 1 (283).jpg]

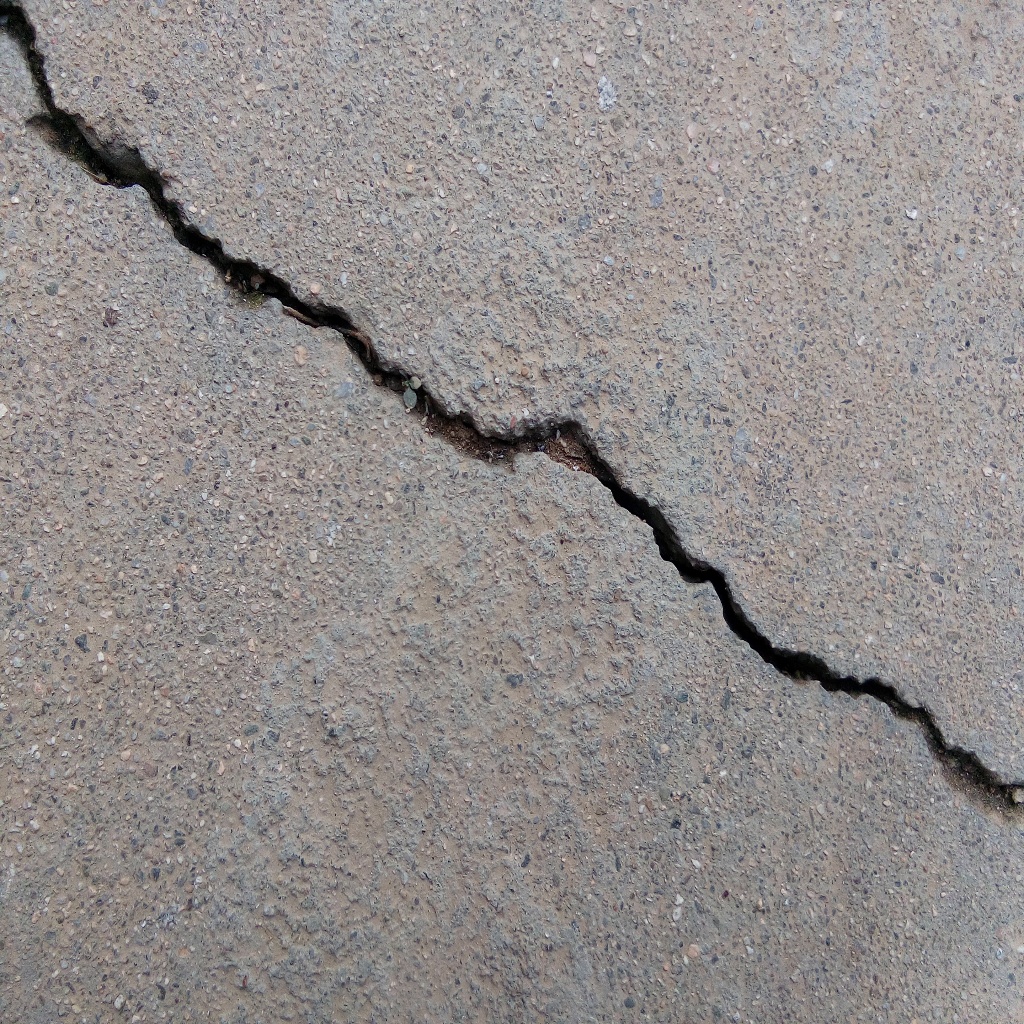

Supplement: S2 File — (ZIP) [file pone.0330218.s002.zip › 1 (284).jpg]

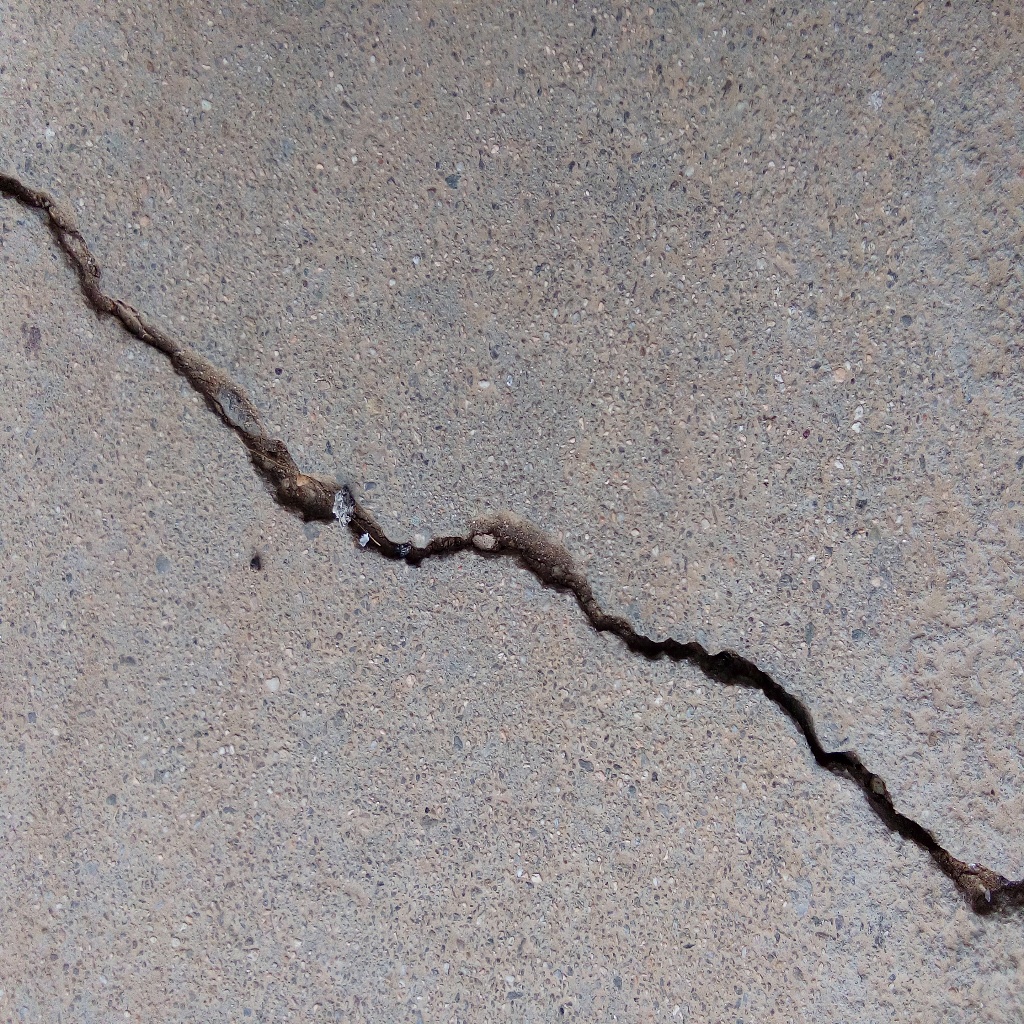

Supplement: S2 File — (ZIP) [file pone.0330218.s002.zip › 1 (285).jpg]

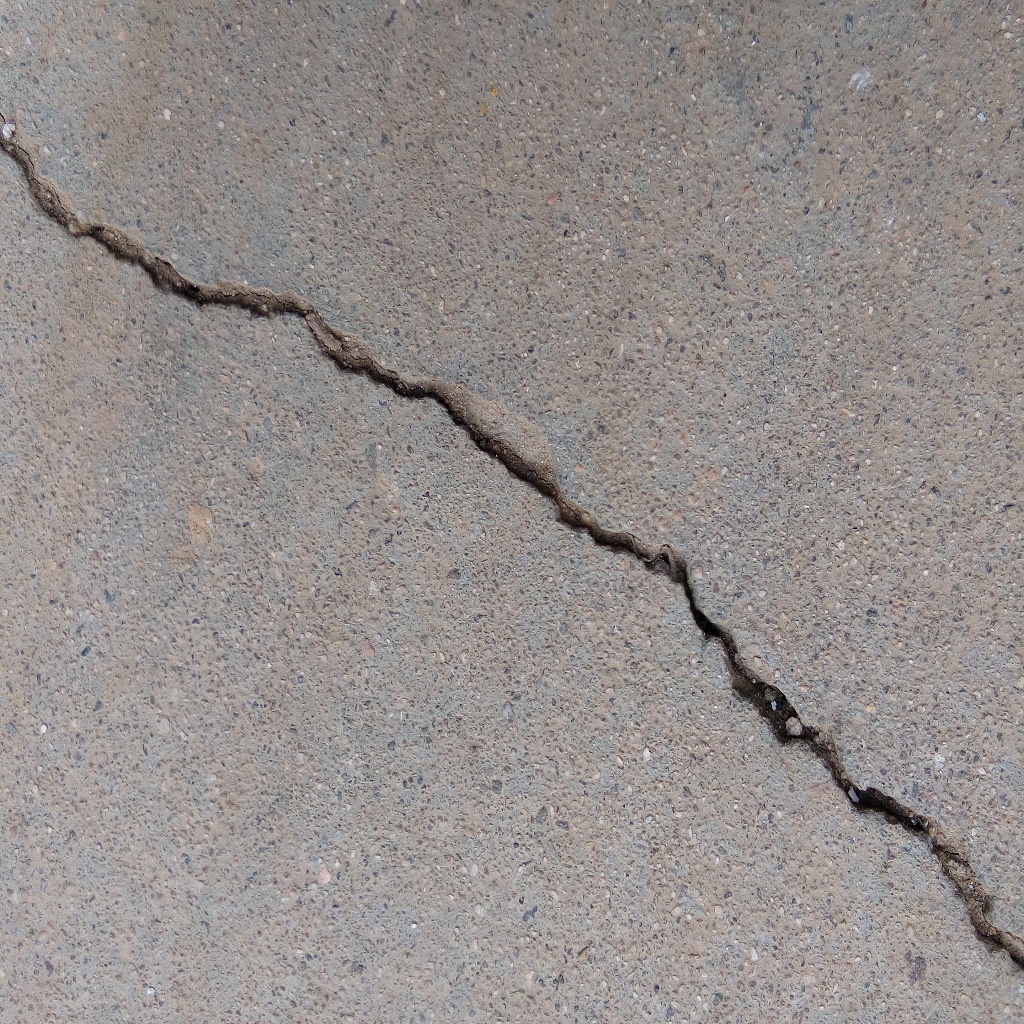

Supplement: S2 File — (ZIP) [file pone.0330218.s002.zip › 1 (286).jpg]

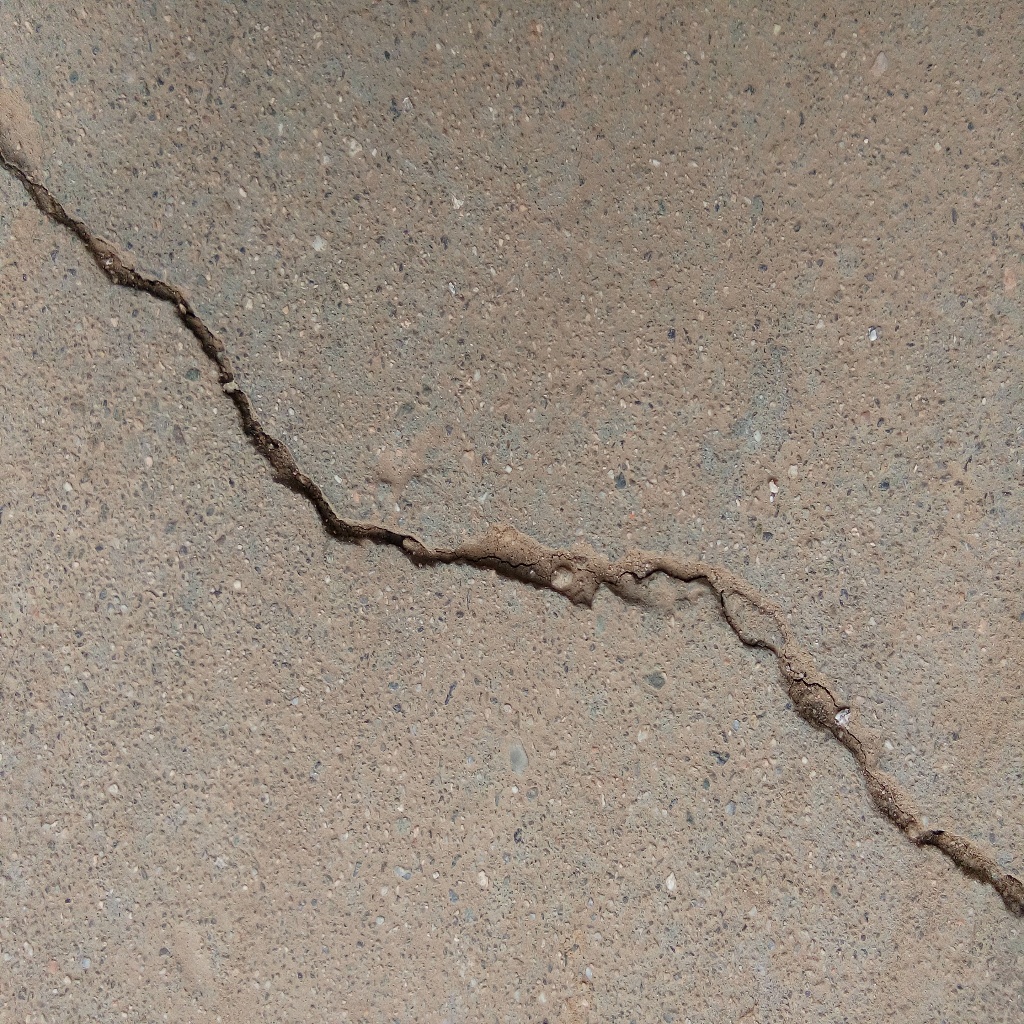

Supplement: S2 File — (ZIP) [file pone.0330218.s002.zip › 1 (287).jpg]

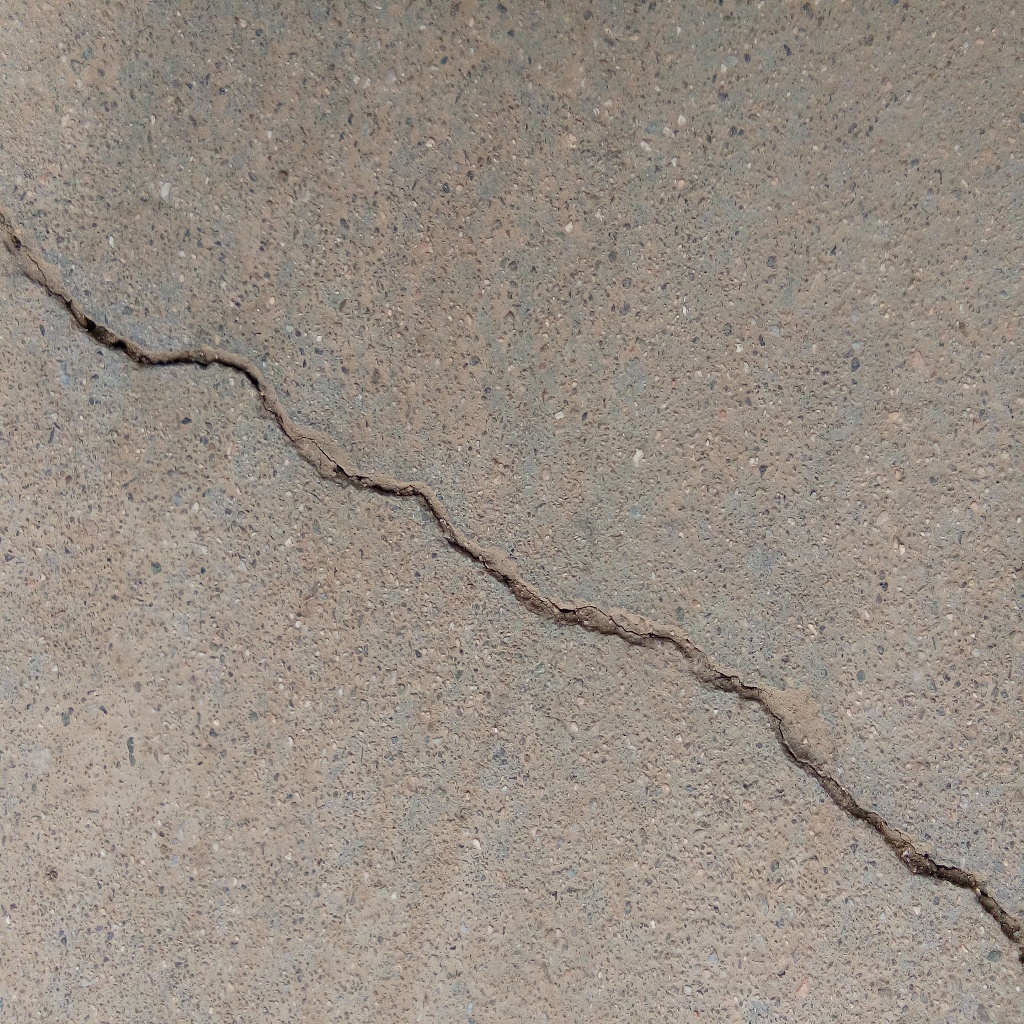

Supplement: S2 File — (ZIP) [file pone.0330218.s002.zip › 1 (288).jpg]

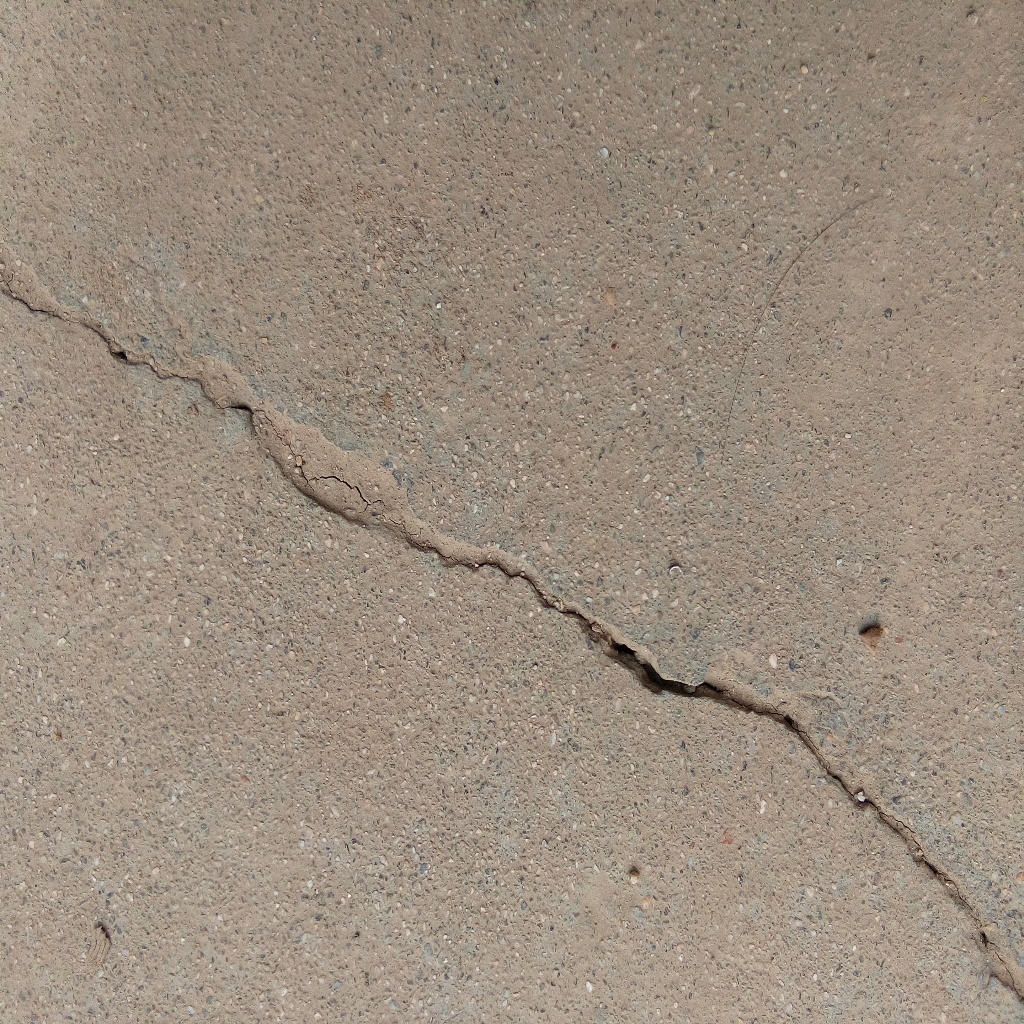

Supplement: S2 File — (ZIP) [file pone.0330218.s002.zip › 1 (289).jpg]

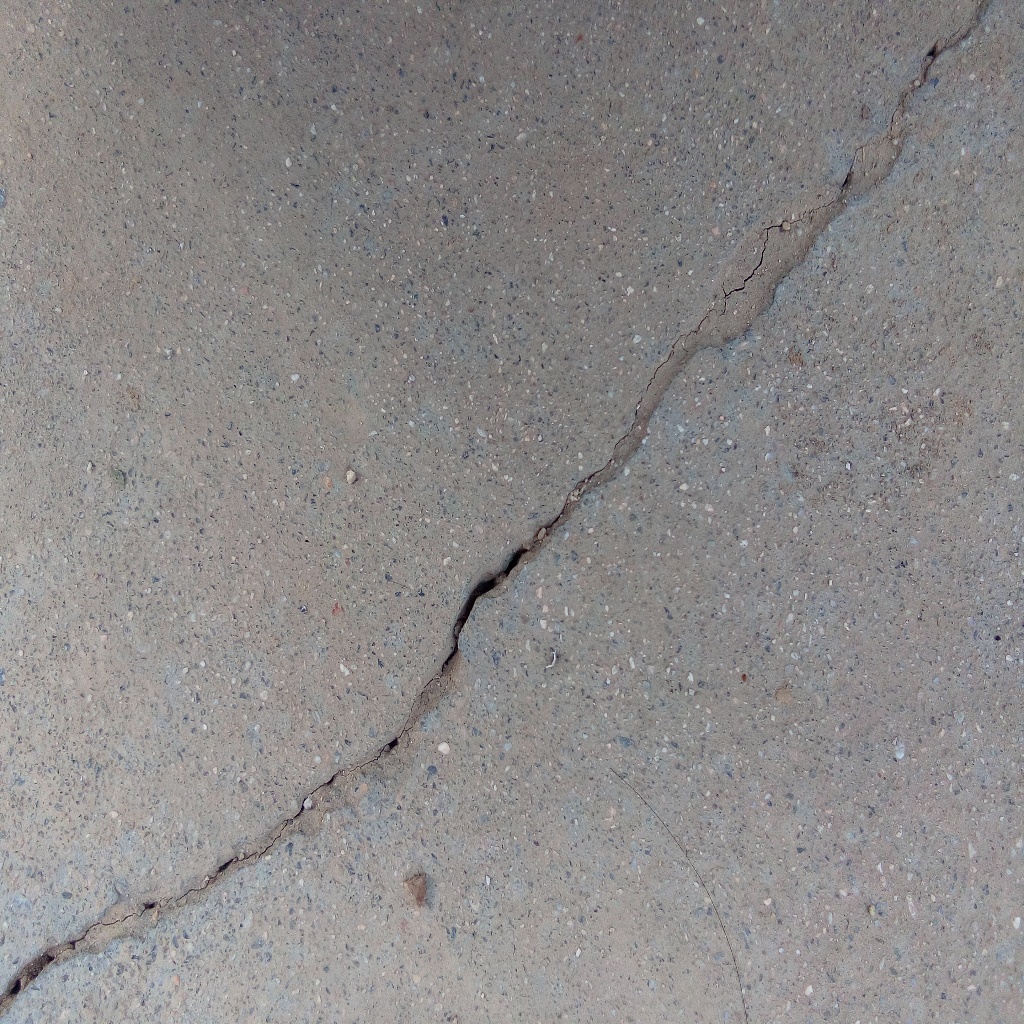

Supplement: S2 File — (ZIP) [file pone.0330218.s002.zip › 1 (290).jpg]

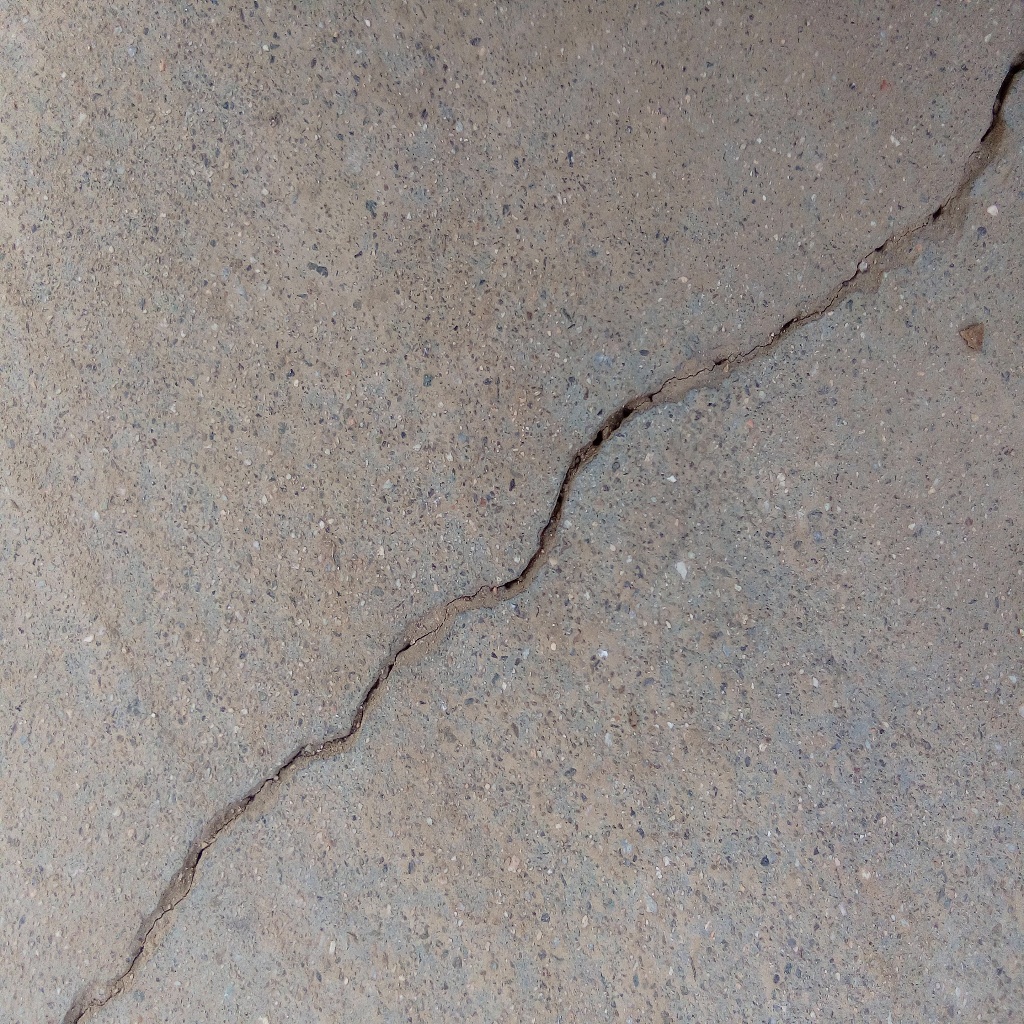

Supplement: S2 File — (ZIP) [file pone.0330218.s002.zip › 1 (291).jpg]

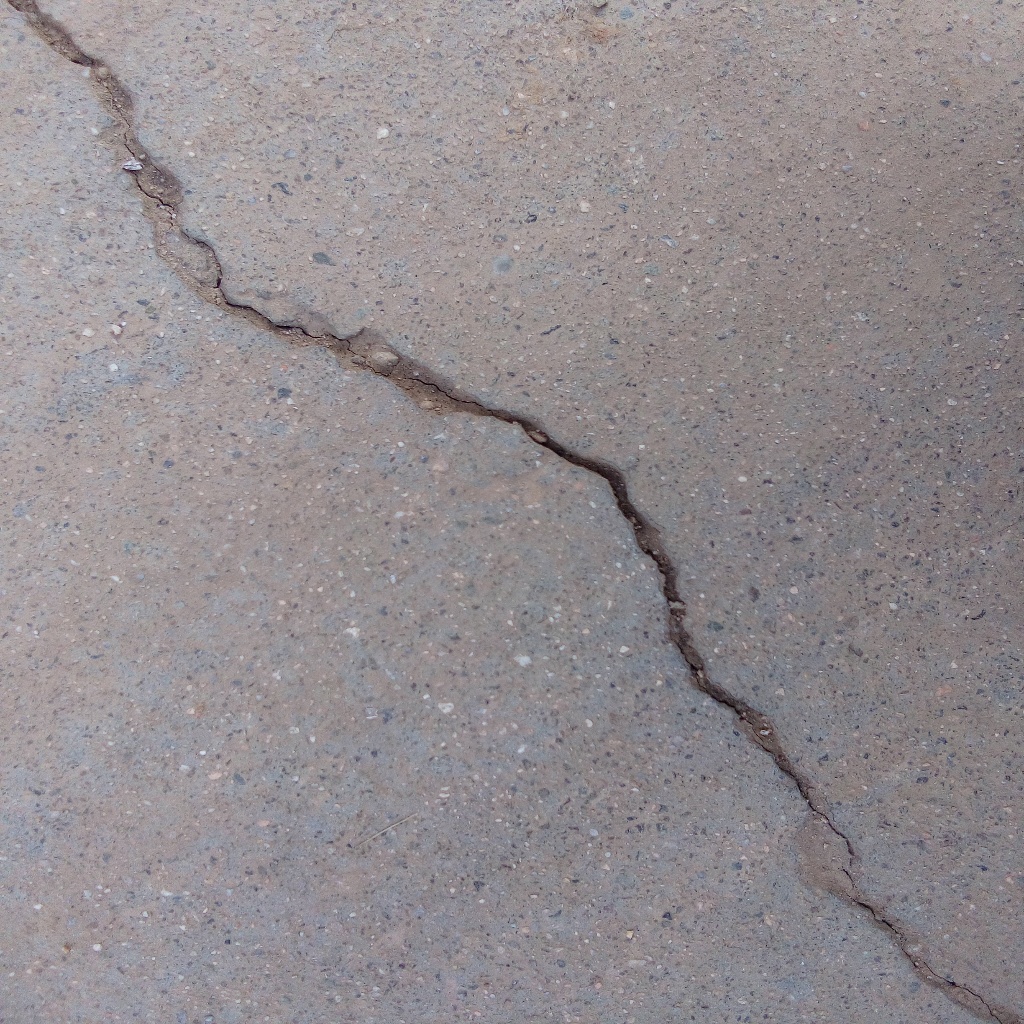

Supplement: S2 File — (ZIP) [file pone.0330218.s002.zip › 1 (292).jpg]

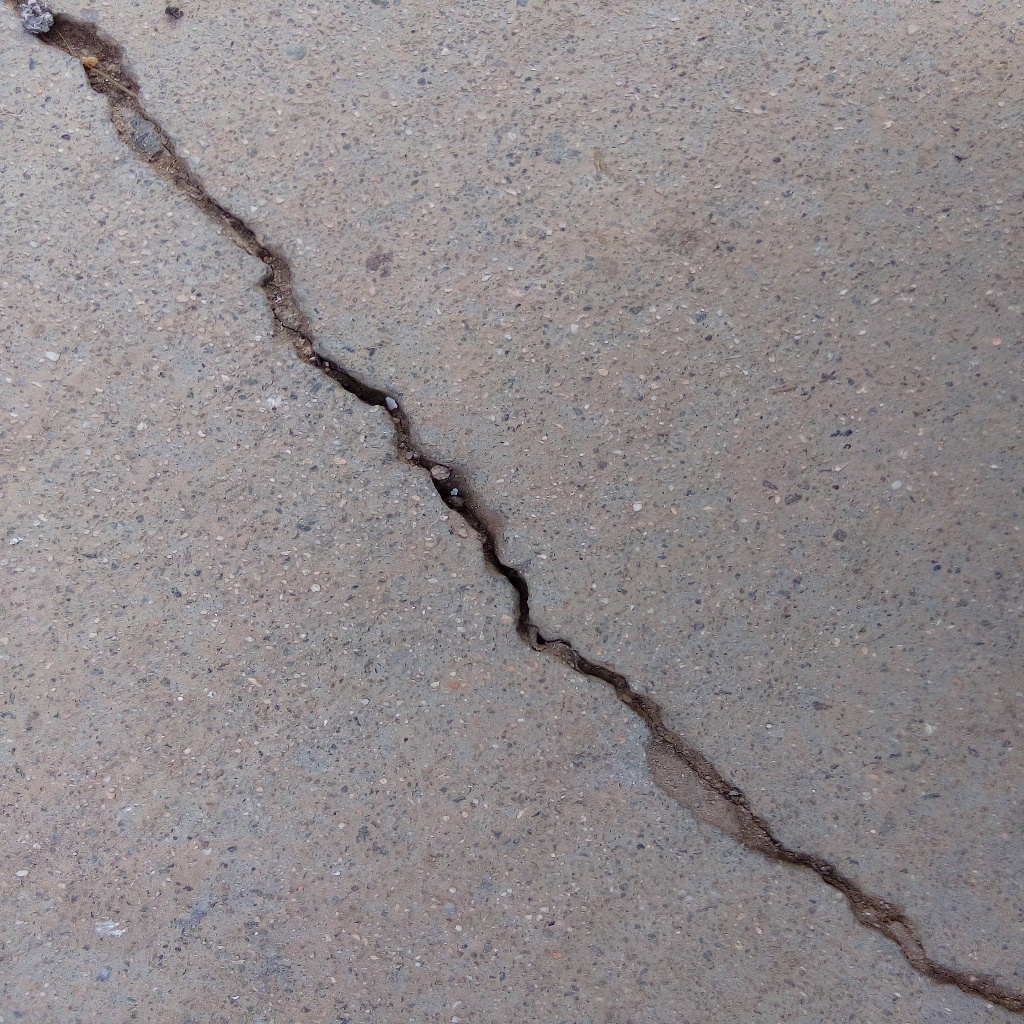

Supplement: S2 File — (ZIP) [file pone.0330218.s002.zip › 1 (293).jpg]

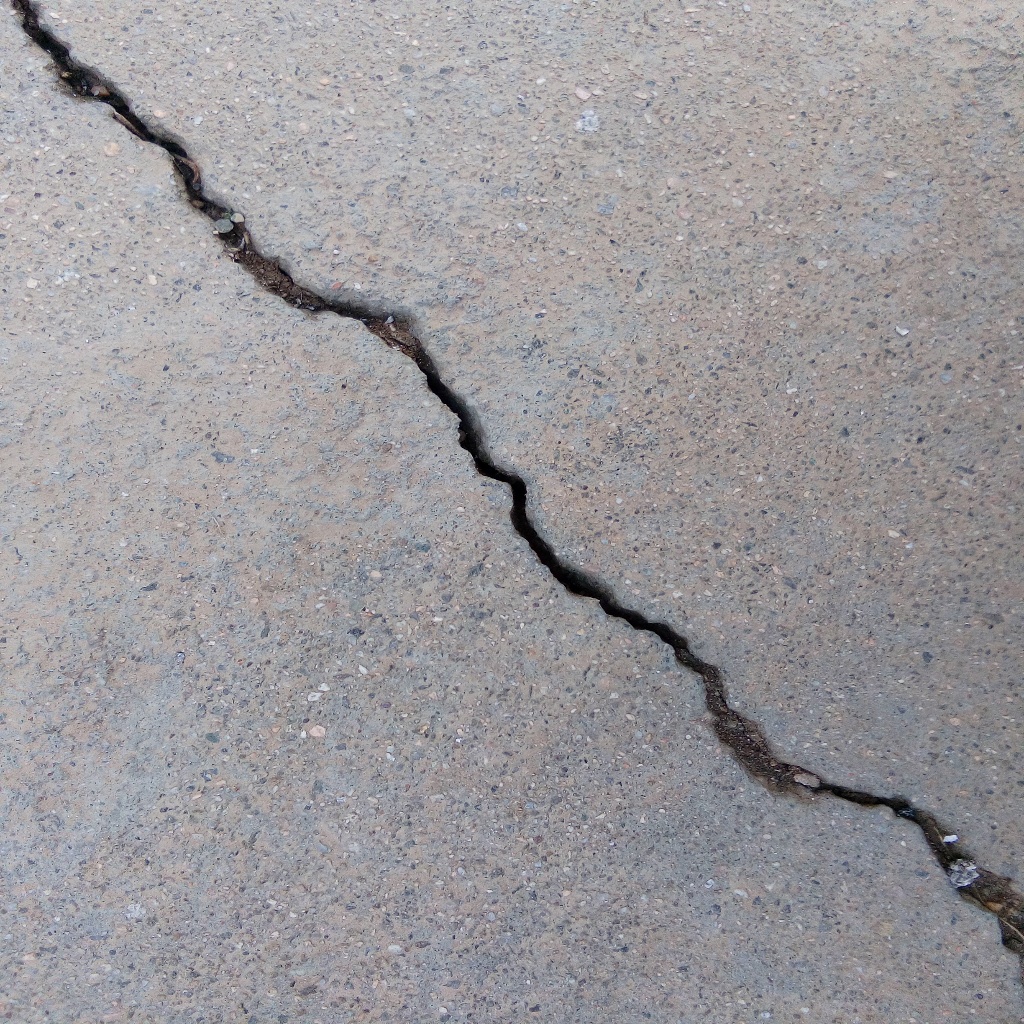

Supplement: S2 File — (ZIP) [file pone.0330218.s002.zip › 1 (294).jpg]

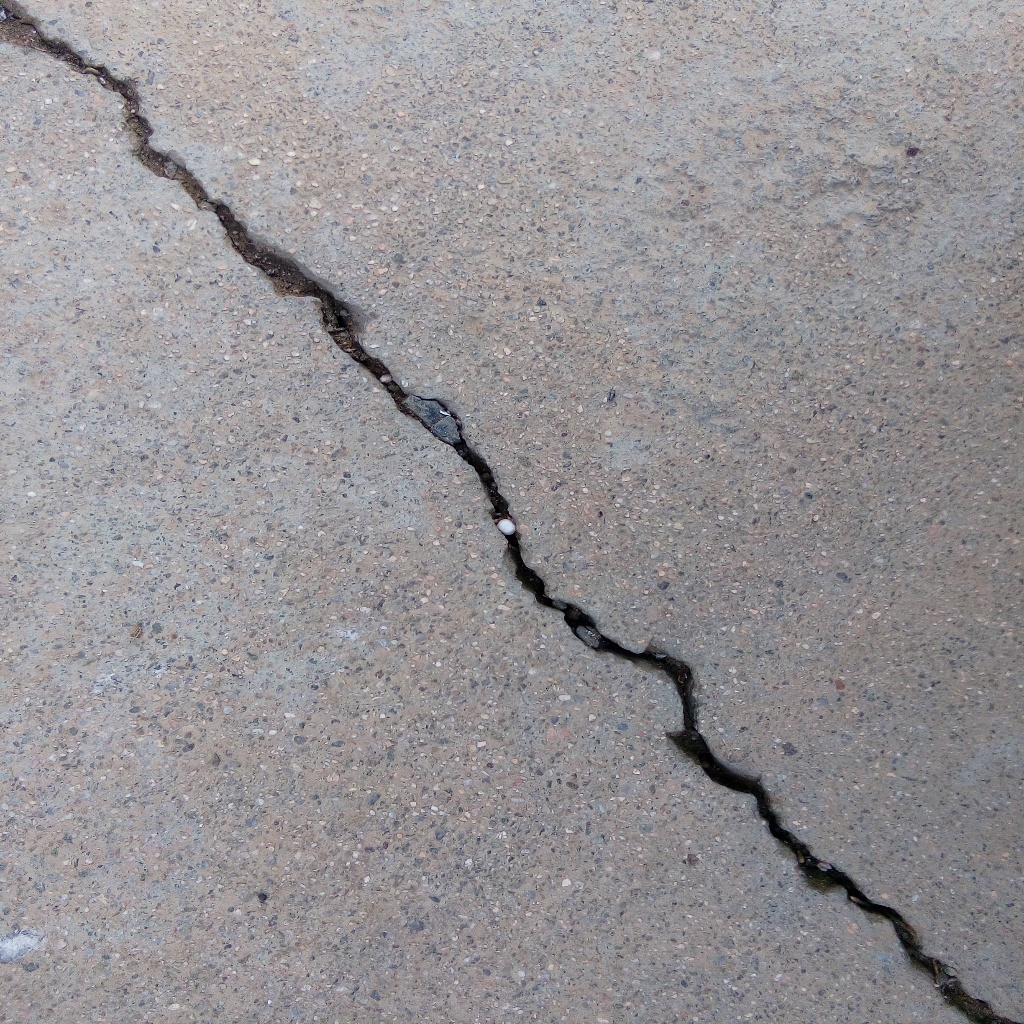

Supplement: S2 File — (ZIP) [file pone.0330218.s002.zip › 1 (295).jpg]

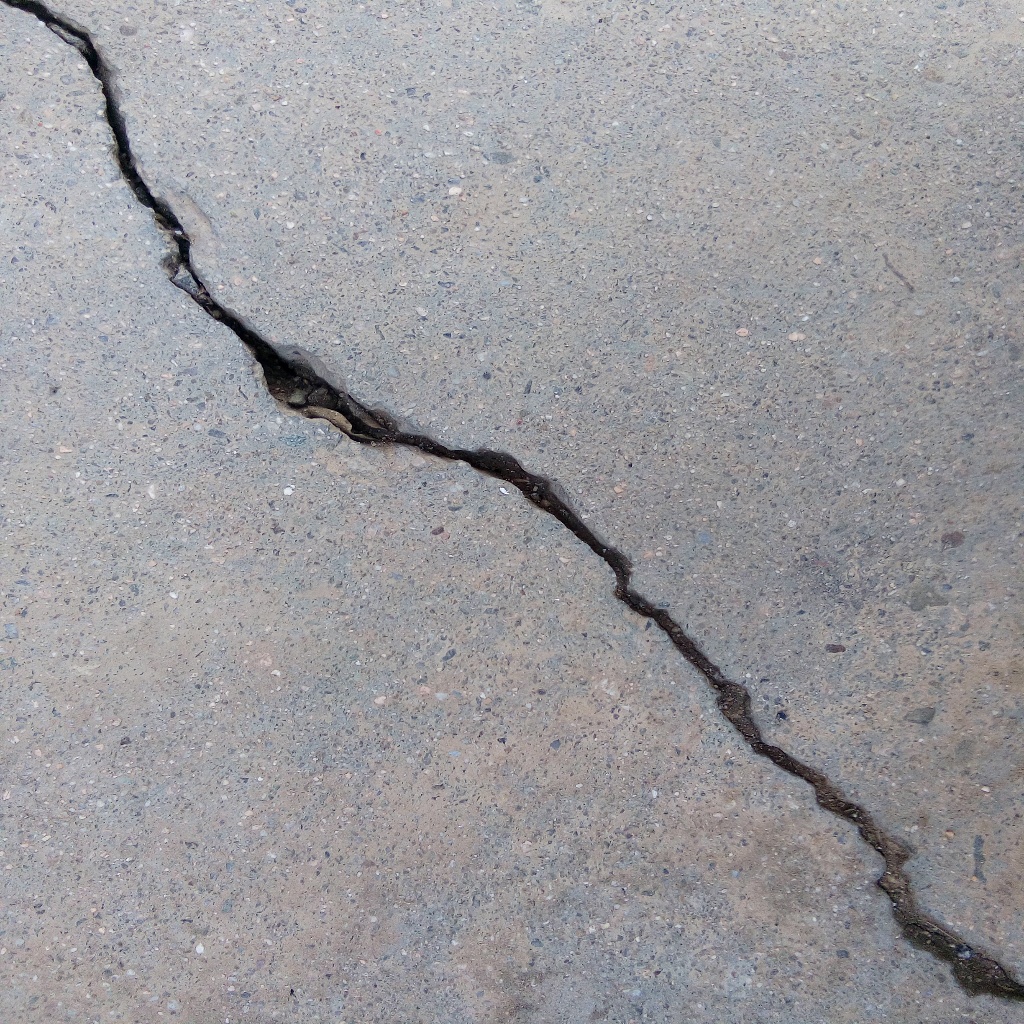

Supplement: S2 File — (ZIP) [file pone.0330218.s002.zip › 1 (296).jpg]

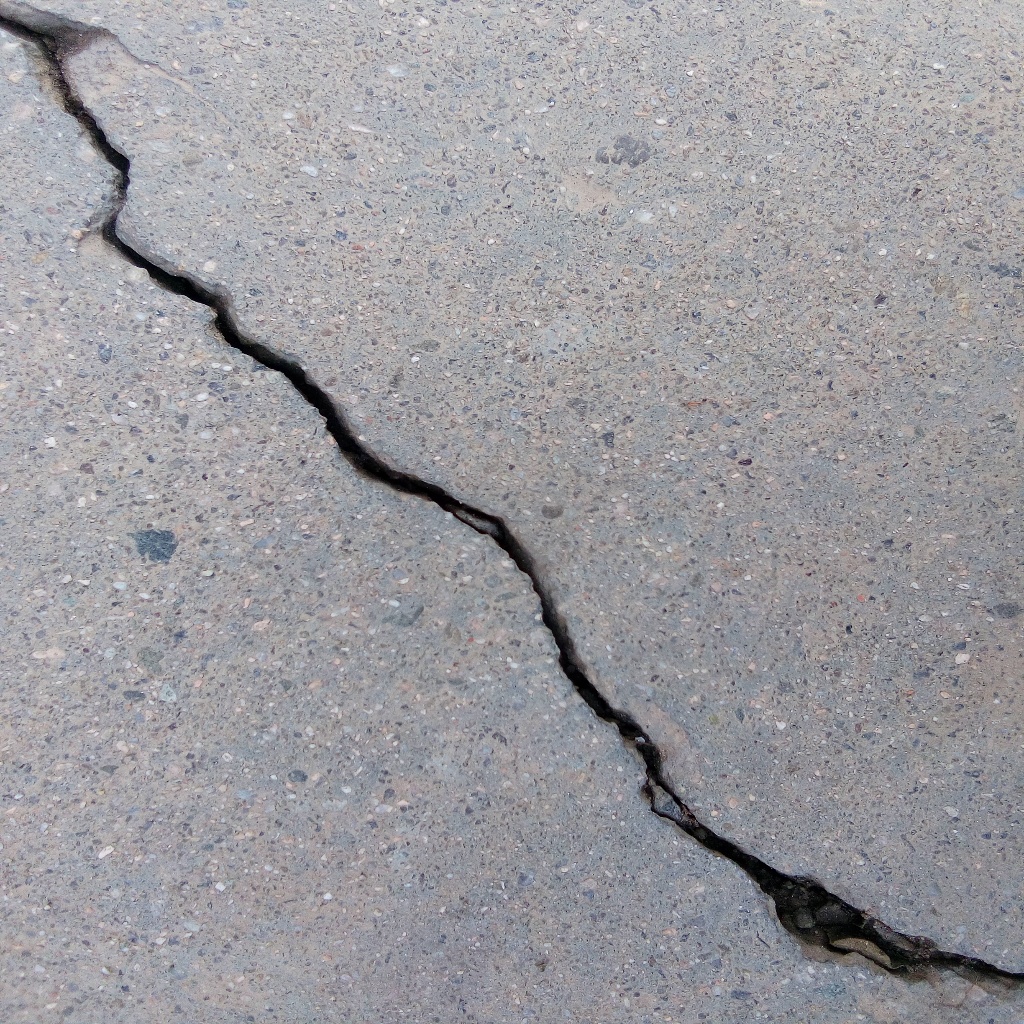

Supplement: S2 File — (ZIP) [file pone.0330218.s002.zip › 1 (297).jpg]

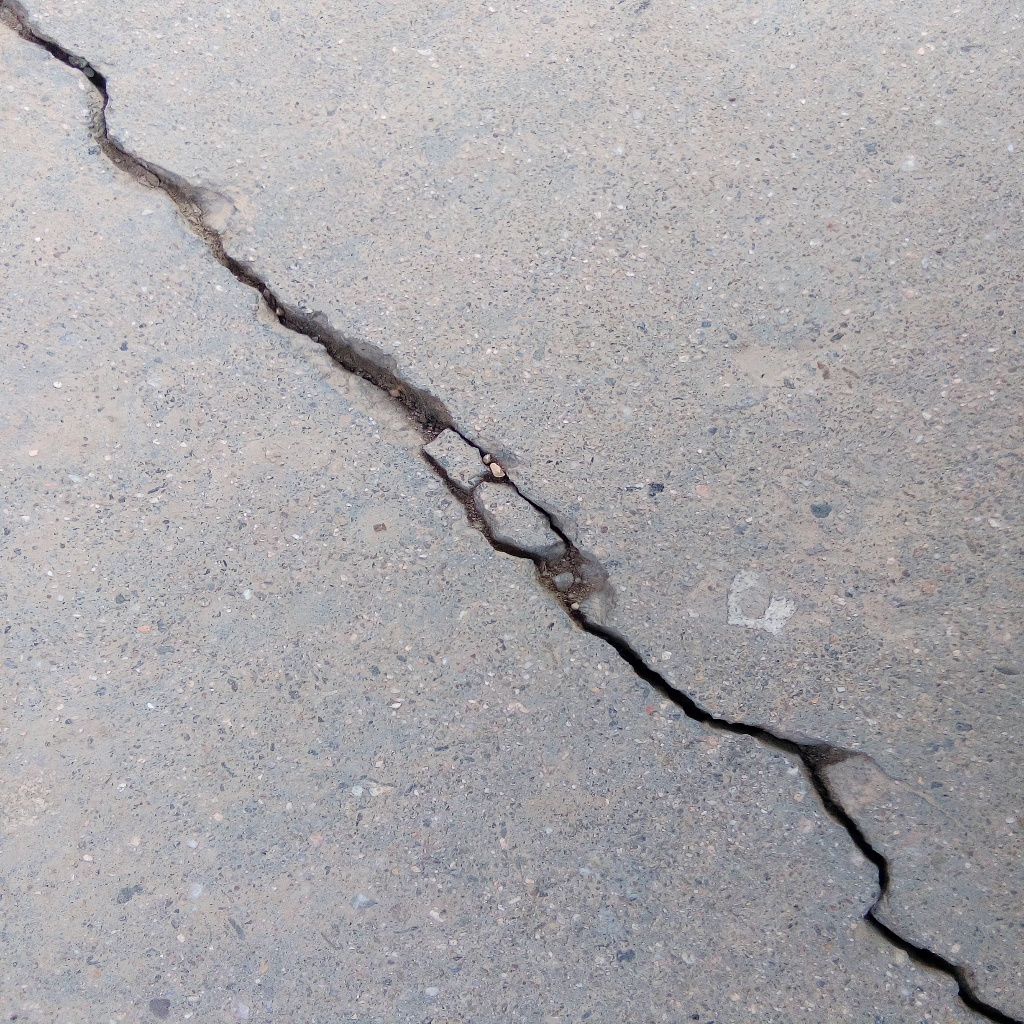

Supplement: S2 File — (ZIP) [file pone.0330218.s002.zip › 1 (298).jpg]
